# World Health Organization–Recommended Periodic Presumptive Treatment Versus Doxycycline Post-Exposure Prophylaxis for Sexually Transmitted Infection Control Among Men Who Have Sex With Men in Kenya: Protocol for a Randomized Controlled Trial

**DOI:** 10.2196/81113

**Published:** 2026-01-06

**Authors:** Susan M Graham, Fredrick O Otieno, Joshua Kimani, Alé Barrientos, Olusegun O Soge, Monisha Sharma, Deven T Hamilton, Connie Celum, R. Scott McClelland, Eduard J Sanders

**Affiliations:** 1 Department of Global Health School of Medicine University of Washington Seattle, WA United States; 2 Department of Medicine School of Medicine University of Washington Seattle, WA United States; 3 Nyanza Reproductive Health Society Kisumu Kenya; 4 Partners for Health and Development in Africa Nairobi Kenya; 5 Department of Global Health School of Public Health University of Washington Seattle, WA United States; 6 Center for Studies in Demography & Ecology University of Washington Seattle, WA United States; 7 Department of Epidemiology School of Public Health University of Washington Seattle, WA United States; 8 Aurum Institute Johannesburg South Africa

**Keywords:** chlamydia, gonorrhea, men who have sex with men, MSM, sexually transmitted infections, STI, syphilis

## Abstract

**Background:**

Men who have sex with men (MSM) are at high risk for bacterial sexually transmitted infections (STIs), including gonorrhea, chlamydia, and syphilis, in Kenya. Because nucleic acid amplification testing (NAAT) is not widely accessible, most gonorrhea and chlamydia infections go undiagnosed and are treated only if symptomatic. World Health Organization (WHO)–recommended periodic presumptive treatment (PPT) and doxycycline post-exposure prophylaxis (doxyPEP) are both potential interventions to reduce the burden of bacterial STIs in this population. Neither has been rigorously tested among MSM in Africa.

**Objective:**

This study aims to evaluate the effectiveness of WHO-recommended PPT versus doxyPEP, compared with standard syndromic treatment, in reducing the prevalence of bacterial STIs, including gonorrhea, chlamydia, and syphilis, among MSM in Kenya.

**Methods:**

We are conducting an open-label randomized controlled trial with 2900 participants assigned in a 2:2:1 ratio to WHO-recommended PPT given every 3 months, doxyPEP taken 24-72 hours after condomless sex, or standard treatment. Sociodemographic, psychosocial, and behavioral data are collected by audio computer-assisted self-interview. Syphilis testing and treatment are provided as part of standard care. Throat and rectal swabs and urine are collected, pooled, and batch tested for gonorrhea and chlamydia by NAAT; these results are not used to guide treatment. The primary trial outcome is the combined prevalence of laboratory-diagnosed gonorrhea, chlamydia, and early syphilis after baseline; secondary outcomes include the prevalence of each pathogen individually and antimicrobial resistance in *Neisseria gonorrhoeae*. Primary and secondary outcomes will be compared between each intervention and the common control arm by estimating relative risks over follow-up (months 3-18) using a modified Poisson model fitted with generalized estimating equations. We will also assess implementation outcomes, including acceptability, feasibility, and safety of each intervention compared to standard care among providers and patients using a mixed methods approach. Finally, we will evaluate the potential health and economic impact of scaling up WHO-recommended PPT and doxyPEP compared to standard of care on STI control among MSM and their partners in Kenya using a stochastic, network-based model and cost-effectiveness analysis on trial data.

**Results:**

Enrollment commenced on October 29, 2025. As of November 25, 2025, a total of 357 participants (12.3% of target) have been enrolled, including 133 in Kisumu, 122 in Nairobi, and 102 in Mombasa. Full enrollment is expected to take 6 months, with follow-up occurring over 18 months per participant. Results will be published in 2028.

**Conclusions:**

Results of this trial will provide critical data needed to inform guidelines to improve STI control among MSM in sub-Saharan Africa and other resource-limited settings where NAAT is not routinely available. Modeled estimates of the health and economic impact of scaling up these two interventions on STI control among MSM and their partners in Kenya will provide critical information to guide policymakers considering adoption of either intervention.

**Trial Registration:**

ClinicalTrials.gov NCT06468462; https://clinicaltrials.gov/ct2/show/NCT06468462

**International Registered Report Identifier (IRRID):**

PRR1-10.2196/81113

## Introduction

Gay and bisexual men and other men who have sex with men (MSM) face disproportionate risk for sexually transmitted infections (STIs), especially in resource-limited settings. The burden of STIs is greatest in low- and middle-income countries, and overlapping, synergistic epidemics of HIV and bacterial STIs in Africa have been recognized since the earliest days of the HIV epidemic [1-3]. Among MSM, STIs caused by *Neisseria gonorrhoeae* (NG) and *Chlamydia trachomatis* (CT) are significant causes of morbidity, ranging from urethritis, epididymitis, and proctitis to disseminated gonococcal infection [4]. Bacterial STIs can also lead to reproductive health problems among female partners of MSM, including pelvic inflammatory disease, chronic pelvic pain, tubal infertility, pregnancy complications, and fetal and neonatal death [1,5-10]. In sub-Saharan Africa, published prevalence estimates among MSM have ranged from 3%-11.5% for NG, 10%-14.5% for CT, and 1.1%-5.7% for early syphilis [11-17]. NG and CT increase the risk of HIV-1 transmission [18], with estimates from one study in the United States suggesting that 10.2% of all HIV infections among MSM are attributable to NG and CT [19]. Effective interventions to reduce the disproportionate burden of STIs among MSM in resource-limited settings are urgently needed.

Syndromic treatment is the standard of care for STI management in most of sub-Saharan Africa; it is less costly than etiologic testing [3], has relatively good sensitivity for male urethritis (87%-99%), and high cure rates when administered [20]. Unfortunately, syndromic treatment misses over 90% of STIs among MSM because they are asymptomatic, especially rectal and pharyngeal infections [12,21]. While laboratory-based or point-of-care nucleic acid amplification testing (NAAT) for NG and CT is widely used in well-resourced settings [22,23], NAAT for NG and CT is prohibitively expensive in African settings, at approximately US $19 per test conducted and US $97 per infection diagnosed, even if urethral, rectal, and pharyngeal samples are pooled [11]. In contrast, the estimated health expenditure per capita in Kenya in 2018 was US $88.39 [24]. The challenges with funding STI diagnostic tests are explicitly acknowledged in the 2022 World Health Organization (WHO) *Implementation Tool for PrEP for HIV Infection*, which recommends a stepwise approach to integration of STI services with HIV pre-exposure prophylaxis (PrEP) services, starting with syndromic management and integrating rapid diagnostic tests and eventually molecular tests depending on resource availability [25]. While the development of low-cost point-of-care diagnostics is an urgent priority [21,26], low-cost and feasible interventions to control STIs among MSM are needed now, as we await technology advances.

One intervention that holds promise to reduce STIs among MSM globally is doxycycline post-exposure prophylaxis (doxyPEP). In 2018, the IPERGAY substudy, a randomized open-label clinical trial of doxycycline 200 mg within 24-72 hours after condomless sex (doxyPEP) among MSM in France, reported a good safety profile, high levels of adherence, and a 47% relative reduction in new bacterial STIs among HIV PrEP users who also took 200 mg of doxycycline following every sexual encounter [27]. This reduction was driven by decreases in incident CT (70% reduction) and syphilis infections (73% reduction); there was no significant impact on NG [27]. Since that time, the DoxyPEP study, an open-label trial among MSM and transgender women living with HIV or taking PrEP in Seattle and San Francisco, found a 66% significant reduction in bacterial STIs in the PrEP cohort and a 62% reduction in the HIV cohort [28]. Reductions for specific infections in these cohorts were lower for NG than for CT and syphilis, at 55% and 57% for NG versus 88% and 74% for CT and 87% and 77% for syphilis, respectively [28]. More recently, the ANRS 174 DOXYVAC study in France, an open-label randomized trial of doxyPEP and meningococcal group B vaccine with a 2 × 2 factorial design, reported a significant reduction in all 3 STIs (ie, NG, CT, and syphilis) in the doxyPEP group with early termination of the control arm. The adjusted hazard ratio in the doxyPEP arm was 0.17 (95% CI 0.12-0.26; *P*<.0001) for CT or syphilis (or both) but only 0.78 (95% CI 0.60-1.01; *P=*.06) for NG; there was no evidence that the vaccine was effective [29]. DoxyPEP as a patient-controlled intervention to reduce STIs could be effective in resource-limited settings, although the lack of quarterly NAAT to identify and treat “breakthrough” NG infections could limit effectiveness of this strategy. Because NG is the only STI that has been associated with increased HIV acquisition among MSM in Kenya [30], its effective control should be a priority. Research to evaluate the overall risk-benefit ratio of doxyPEP, in terms of efficacy, long-term safety, impact on antimicrobial resistance (AMR), and cost-effectiveness among MSM in resource-limited settings is needed.

One additional STI control strategy has been proposed. In 2011, the WHO recommended periodic presumptive treatment (PPT) to eradicate asymptomatic NG and CT infections for MSM and transgender women who report condomless receptive anal intercourse and either multiple sex partners or a sex partner with an STI in the past 6 months [4]. No specific frequency of this intervention was recommended, and readers were referred to syndromic treatment guidelines for regimen details [4]. Although targeting criteria for this recommendation have been evaluated by our research in Kenya [31,32], evidence of the efficacy of PPT among MSM is lacking, as is an evaluation of its potential downsides. Several studies on single-dose or monthly antibiotics among female sex workers in Asia and Africa demonstrated reduced disease burden using various PPT strategies [33-38]. Moreover, a meta-analysis of PPT intervention effects showed a statistically significant impact on the incidence of curable STIs in 13 of 14 studies included [39]. The premise that PPT could help reduce STI burden among MSM in resource-limited settings is supported by this evidence, yet an investigation of the potential impact, risks, and benefits of PPT for MSM in a randomized controlled trial has not been conducted. As a provider-delivered strategy that can be directly observed, PPT using cefixime and azithromycin, the currently recommended syndromic regimen for CT and NG [40], could prove more effective than doxyPEP if doxyPEP adherence is suboptimal or if AMR limits the impact of doxyPEP on NG control.

The potential emergence of AMR is an important concern for both doxyPEP and PPT. Following early studies of empiric single-dose antibiotics to reduce NG burden among female sex workers in the Philippines, some women (12%) reported self-prescribing antibiotics for empiric treatment, and high rates of penicillin-resistant NG were associated with self-prescription [41]. Today, multidrug resistant NG is a growing international public health issue [42-47]. In sub-Saharan Africa, reports of cephalosporin resistance in NG have been relatively rare (<1%) to date; however, high levels of tetracycline and quinolone resistance have been reported, with detection of plasmid-mediated tetracycline-resistant NG estimated at 73%-97% [42,43,48-50]. In Kenya specifically, no confirmed cephalosporin resistance has been reported [42,51,52], although one study reported increasing minimum inhibitory concentrations for ceftriaxone in isolates collected between 2002 and 2009 [48]. Of note, doxycycline resistance in NG is estimated at 56% in France [53,54], where doxyPEP had unclear efficacy against NG in the IPERGAY study, compared to 20%-30% in the United States, where the doxyPEP study demonstrated a significant decrease in NG incidence [28], but has led to an increase in tetracycline resistance in NG in the community [55]. Rigorous studies are needed to quantify the scope of potential resistance that might accompany doxyPEP or PPT in resource-limited settings with high levels of tetracycline resistance, to mitigate this critical problem.

In this study, we will use a hybrid type 1 implementation-effectiveness trial to address three objectives: (1) to evaluate the effectiveness of two interventions: WHO-recommended PPT given every 3 months and doxyPEP taken 24-72 hours after condomless anal sex, compared to standard syndromic treatment, for reducing the combined prevalence of laboratory-diagnosed gonorrhea, chlamydia, and early syphilis (primary trial outcome) among Kenyan MSM; (2) to assess implementation outcomes including the acceptability, feasibility, and safety of each intervention compared to standard care among providers and patients, using a mixed methods approach; and (3) to evaluate the potential health and economic impact of scaling up WHO-recommended PPT and doxyPEP compared to standard of care on STI control among MSM and their partners in Kenya using a stochastic, network-based model and cost-effectiveness analysis based on trial data.

## Methods

### Study Design

We will conduct an open-label randomized controlled trial with a hybrid type 1 implementation-effectiveness component, enrolling 2900 participants for 18 months of follow-up. The trial will evaluate 2 interventions (ie, PPT vs doxyPEP) versus the standard of care assigned in a 2:2:1 ratio.

### Setting

The trial will be conducted at 3 research clinics in Kenya. In Nairobi, participants will be enrolled and followed at the Sex Workers Outreach Programme TRANSFORM Clinic, which is managed by Partners for Health and Development in Africa. In Kisumu, participants will be enrolled and followed at the Anza Mapema Clinic, which is managed by the Nyanza Reproductive Health Society. In Mombasa, participants will be enrolled and followed at Hapa-Kenya, a community-based organization serving the LGBTQ+ community, in partnership with the Pwani Research Center, University of Washington, University of Nairobi, and the Aurum Institute.

### Population

To participate, individuals must be aged 18-29 years, be assigned male sex at birth, identify as male, report condomless anal intercourse and either multiple male sex partners or a male sex partner with a syndromic (urethritis, proctitis, or genital ulcer disease) or laboratory-diagnosed STI (NG, CT, or syphilis) in the past 6 months, and be willing and able to give written informed consent. Exclusion criteria include: allergy to cephalosporin, macrolide, or tetracycline class antibiotics; recent use of prolonged antibiotics (≥14-day course) in the month before enrollment; use of medications that impact cefixime, azithromycin, or doxycycline metabolism; ongoing participation in another clinical trial; or active, clinically significant medical or psychiatric conditions that would interfere with study participation, at the discretion of the site investigator.

### Recruitment and Retention

At each site, recruitment will be conducted by fliers, posters, information sessions, word of mouth, and social media apps (Facebook, WhatsApp) to increase awareness of the study at each research clinic and at local partner organizations in each site’s catchment area. In addition, we will recruit participants through experienced peer outreach workers at previously mapped MSM hot spots, including bars, discos, restaurants, and social halls. Retention efforts will be supported by ensuring that the trial sites offer high-quality MSM-friendly services, offer flexible hours, monitor clinic flow and wait times, and provide HIV and STI treatment free of charge. In addition, we will use text or WhatsApp reminders 3 days and 1 day before a scheduled visit to promote timeline attendance. Tracing of participants who miss visits will be the responsibility of the outreach teams at each research site.

### Procedures

**Figure 1:**
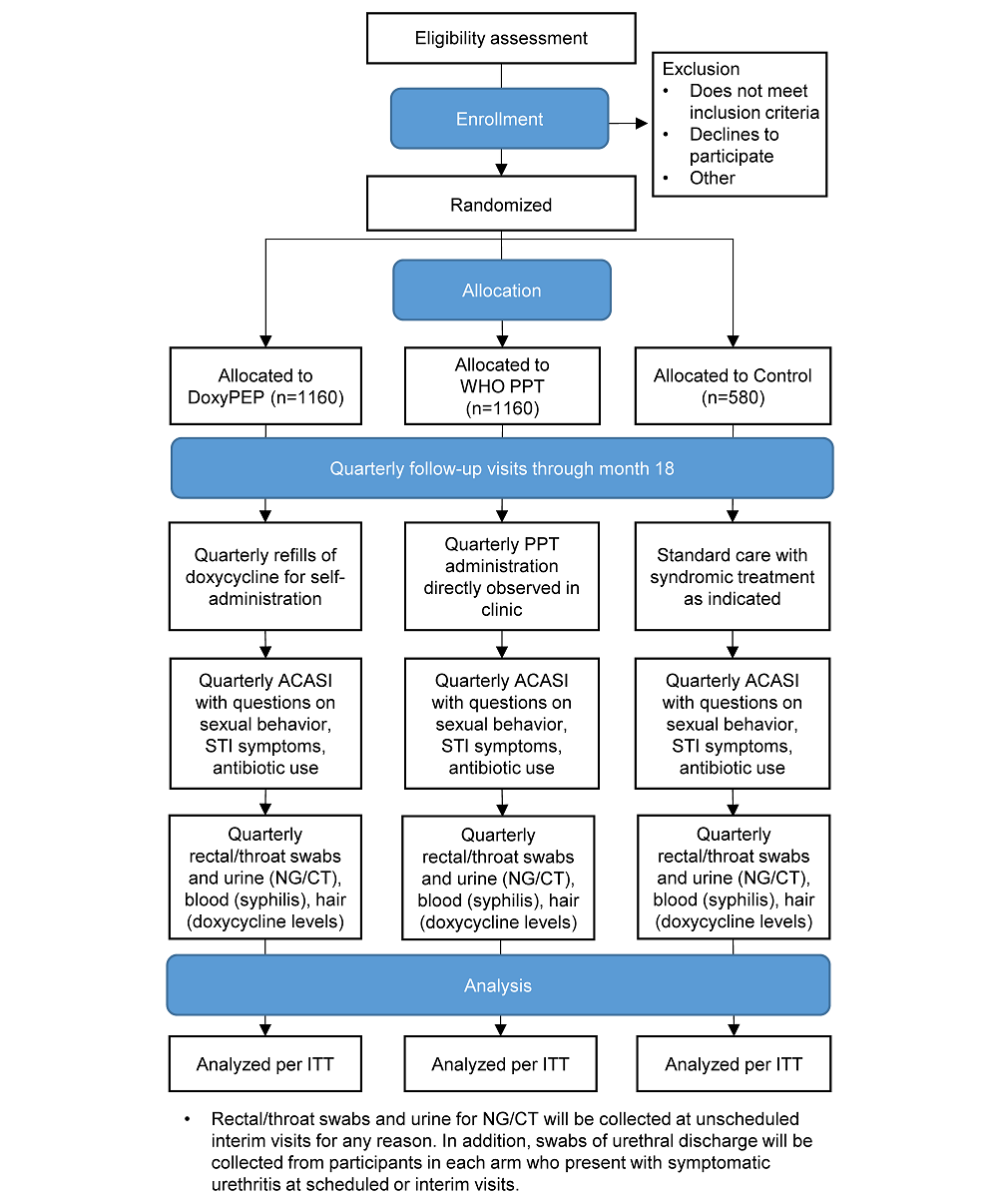
CONSORT (Consolidated Standards of Reporting Trials) flow diagram. ACASI: audio computer-assisted self-interview; CT: *Chlamydia trachomatis*; doxyPEP: doxycycline post-exposure prophylaxis; ITT: intention-to-treat; NG: *Neisseria gonorrhoeae*; PPT: periodic presumptive treatment; STI: sexually transmitted infection; WHO: World Health Organization.

Interested individuals will receive information on the study interventions and randomization and randomization process, then screened for eligibility. Sociodemographic data will be collected for all individuals screened, whether or not they are determined eligible or choose to enroll. Those who are eligible and choose to enroll will provide written informed consent. Participants will then be randomized in variable-sized blocks stratified by site, using REDCap (Research Electronic Data Capture; Vanderbilt University), a secure web application for building and managing databases for research studies [56]. Participants will attend quarterly visits, which are standard of care for HIV prevention and care services, over 18 months of follow-up. At each visit, we will collect participant data via REDCap using an audio computer-assisted self-interview (ACASI), with audio recordings available in English, Swahili, and Dholuo, depending on participant preference. Clinical data on symptoms, exam findings, specimen collection, and intervention delivery will be entered in REDCap data forms. Figure 1 presents a CONSORT (Consolidated Standards of Reporting Trials) flow diagram of the study, from enrollment and allocation through quarterly follow-up. Multimedia Appendix 1 provides the REDCap codebook for the different forms used, including the ACASI survey and clinical data forms; of note, many fields in the ACASI relate to the audio recordings in English, Swahili, and Dholuo, which follow each question and are made active using skip rules.

### Study Arms

The study comprises three arms: PPT intervention, doxyPEP intervention, and standard care.

#### Periodic Presumptive Treatment Intervention

Participants assigned to the PPT arm will be evaluated at baseline and every 3 months thereafter for PPT eligibility based on having had condomless anal sex and either multiple sex partners or a sex partner with an STI in the past 6 months (at baseline) [32] or in the past 3 months (at follow-up visits). If eligible at a given visit, they will be offered 400 mg by mouth (po) cefixime plus 1 gram azithromycin by mouth under direct observation, using the same regimen as for syndromic treatment per the latest WHO and Kenyan recommendations [57].

#### Doxycycline Post-Exposure Prophylaxis Intervention

Participants assigned to the doxyPEP arm will be provided with a 30-dose supply (ie, 60 capsules) of doxycycline hyclate at each quarterly visit and given 1:1 counseling on the self-administration of 200 mg of doxycycline by mouth within 24-72 hours after condomless anal or vaginal sex as frequently as daily if indicated but not more than once daily, in accordance with the DoxyPEP trial in the United States [28]. Counseling to promote doxyPEP adherence will be repeated at each follow-up visit and any interim visits, and refills will be provided by the study team upon request by telephone, WhatsApp, or in person by participants in this arm. Of note, participants in the DoxyPEP trial used a median of 15 doses per quarter (IQR 4-30), with a similar number of doses used (17 doses per quarter, IQR 7-32) in the open-label extension [58].

#### Standard Care

Participants assigned to the standard care arm will receive no STI control intervention (ie, neither PPT nor doxyPEP). Standard care provided at every scheduled visit to all participants (regardless of study arm) includes syphilis testing, with treatment as indicated in accordance with Kenyan guidelines [59], and screening for symptoms of urethritis or proctitis. Participants with symptomatic urethritis or proctitis will receive syndromic treatment with 400 mg of cefixime and 1 gram of azithromycin by mouth under direct observation, in accordance with Kenyan guidelines [59]. This regimen will be updated if Kenyan treatment recommendations change.

### Operational Components

Textbox 1 summarizes the operational components of the randomized controlled trial. Participants may return to the study clinic for interim visits at any time STI symptoms are present, regardless of study arm. All treatment will be documented, whether provided at a scheduled quarterly or an interim visit. Fidelity of delivery of each intervention will be closely monitored and documented, using a detailed standard operating procedure with a checklist of steps completed.

**Operational components of the randomized controlled trial.**

**Inclusion criteria**

- Aged 18-29 years; assigned male sex at birth; identifies as male; reports condomless anal intercourse and either multiple male sex partners or a male sex partner with a syndromic (urethritis, proctitis, or genital ulcer disease) or laboratory-diagnosed sexually transmitted infection (STI, such as *Neisseria gonorrhoeae* [NG], *Chlamydia trachomatis* [CT], or syphilis) in the past 6 months; willing and able to give written informed consent.

**Exclusion criteria**

- Allergy to cephalosporin, macrolide, or tetracycline class antibiotics; recent use of prolonged antibiotics (≥14-day course) in the month before enrollment; use of medications that impact cefixime, azithromycin, or doxycycline metabolism; ongoing participation in another clinical trial; or active, clinically significant medical or psychiatric conditions that would interfere with study participation at the discretion of the site investigator.

**Sample size**

- A total of 2900 men who have sex with men (MSM); randomized 2:2:1 to directly observed periodic presumptive treatment (PPT) or doxycycline post-exposure prophylaxis (doxyPEP) versus standard care (syndromic STI treatment only).

**Visit schedule**

- Screening and enrollment, then quarterly for 18 months; reminders and tracing in case of missed visits to ensure high retention.

**Standard of care package**

- All participants: syphilis testing and treatment, syndromic STI treatment, partner notification per current guidelines as indicated, risk reduction counseling, condoms, lubricants, and standard HIV prevention or care services according to HIV status.

All study sites include programmatic HIV prevention and care services. During the informed consent process, participants will be asked permission to link their programmatic data to the trial records. Within these programs, HIV testing and counseling will be conducted for previously HIV-negative participants at each quarterly visit, per Kenyan guidelines for key populations. Antiretroviral therapy (ART) will be provided to participants with HIV, and PrEP will be provided to those who test HIV negative and are willing to take daily or intermittent tenofovir disoproxil fumarate-based oral PrEP.

### Specimen Collection, Testing, and Storage

At each quarterly follow-up visit, participants will undergo hair collection for testing of doxycycline levels, blood collection for syphilis serology, and 3-site screening for NG and CT, with provider-collected or participant-collected (if provider collection is refused) pharyngeal and rectal swabs (2 of each) and a clean-catch urine. Participants who return to the study clinic for interim STI symptoms will undergo collection of the same pharyngeal, rectal, and urine samples, but no blood sample collection unless acute syphilis is suspected. We will not diagnose and treat infections other than syphilis in real time. Syphilis serology including rapid plasma reagin (Becton Dickinson) and, if reactive, *Treponema pallidum* hemagglutination assay (Biotec) will be tested at quarterly visits, with treatment provided to participants with a new syphilis diagnosis as soon as results are available. Participants who have symptomatic urethritis at any study visit (estimated at 5% of visits or ≈1015 visits) will undergo swab collection of urethral discharge for NG culture using modified Thayer-Martin Agar, a selective medium supplemented with nystatin, colistin, vancomycin, and trimethoprim that favors growth of NG while suppressing genital and extragenital commensal microbial flora growth. Antimicrobial susceptibility testing will be performed using disk diffusion for penicillin and tetracycline and E-test for cefixime, azithromycin, and doxycycline, using WHO control strains for quality assurance of antimicrobial susceptibility testing at each of the site’s microbiology laboratories [60].

All samples for NAAT will be transferred to and stored in the University of Washington–University of Nairobi East Africa STI Laboratory in Mombasa, which has operated with external quality assurance for >10 years. Aptima Combo 2 testing for NG and CT will be conducted in batches on a Hologic Panther platform by experienced laboratory staff blinded to randomization assignment. To reduce testing costs, we will pool the 3 samples from each participant for initial testing following published protocols [61,62]. All NG-positive pools will be broken down and individually tested to identify the infected site or sites [61]. All NG, CT, and early syphilis diagnoses will be reviewed by an Endpoint Adjudication Committee consisting of 2 STI experts on the University of Washington faculty who will be blinded to treatment arm. A random sample of 10% of all hair samples collected (≈812 samples) from the month 6 and month 18 visits in the doxyPEP arm will be shipped to the Hair Analytical Laboratory at the University of California at San Francisco for testing for doxycycline levels [63].

### NG Antimicrobial Resistance Testing

The potential for selection of AMR in NG by PPT or doxyPEP is one of the greatest risks to the success of these strategies; however, the magnitude of this risk cannot be known without empiric data. Cephalosporin resistance in NG is happily still rare in Kenya. Azithromycin resistance in NG is uncommon in Kenya (0%-8.3% in recent studies) [42,51], and PPT may not increase it substantially given co-coverage with cefixime. Resistance to azithromycin in CT has not been seen, although it is known to be less effective at eradicating rectal chlamydia than doxycycline [64]. Plasma-mediated doxycycline resistance in NG is common and may impact the efficacy of doxyPEP [50].

Given the importance of AMR in NG as a public health problem, we will focus on this organism. We will perform NG culture and susceptibility testing of urethral swabs from symptomatic urethritis cases, as described above, due to the high yield of these specimens on culture [65], but will supplement this testing with rigorous molecular methods using residual NAAT samples and noncultured swab or urine specimens to identify well-characterized genetic AMR determinants conferring cefixime, azithromycin, or tetracycline resistance in NG. Of note, we have validated and successfully used these methods for strain typing and characterization of resistance determinants in NG [50,66,67].

We will select a random sample of 25% of all NG-positive specimens (≈761 samples) from each intervention arm (50% at baseline and 50% at follow-up) for shipment to the University of Washington for molecular testing. DNA will be purified and extracted from residual NAAT samples, swabs, or urine in the Soge Laboratory using the Total Nucleic Acid Isolation Kit on the Roche MagNA Pure 24 System (Roche Diagnostics), an automated nucleic acid purification system that has been validated to yield high-quality DNA from NAAT and clinical specimens [68]. Molecular characterization of NG will include strain typing using the internationally used *N. gonorrhoeae* multiantigen sequencing typing system [69]; detection of plasmid-mediated *tetM*, which confers high-level tetracycline resistance, and the chromosomally mediated mutation rpsJ V57M, which confers low-level tetracycline resistance [70]; detection of single-nucleotide polymorphisms associated with reduced cephalosporin (cefixime or ceftriaxone) susceptibility and resistance (mosaic penA alleles, *penA* A311V, *penA* N513Y, ponA L421P, mtrR −35delA, and porB G120K/A121D/N) [71-74]; and detection of azithromycin resistance (23S rRNA A2059G, C2611T) [72,75] by polymerase chain reaction and sequencing. Well-characterized positive control strains and negative controls will be included in all polymerase chain reaction assays [76].

### Primary and Secondary Trial Outcomes

The primary trial outcome will be the combined prevalence of NG, CT, and early syphilis ascertained by laboratory diagnosis (eg, positive NAAT for NG or CT or new syphilis diagnosis, based on first positive rapid plasma reagin or a fourfold increase in nontreponemal titers). Secondary trial outcomes will be the prevalence of each pathogen individually and the prevalence of AMR to the different antibiotics assessed. Outcomes will be assessed at baseline and every 3 months for 18 months. We have selected prevalence instead of incidence as our outcome of interest because a major drawback of syndromic treatment is its failure to identify and treat asymptomatic infections that can therefore persist as a reservoir for transmission, despite spontaneous clearance of some infections [77].

### Power and Sample Size

The trial will follow a standard randomized superiority design, with comparison of each intervention separately to the standard of care arm. To detect at 33% reduction in STI prevalence (from 16% to 10.8%) for each intervention compared to standard care, we will need 2465 participants total: 986 participants in each intervention group and 493 in the control group (α=0.05, β=0.20). Accounting for 15% attrition after randomization, we will need 2900 participants total: 1160 participants in each intervention group and 580 in the control group. We will target enrollment of ≈967 participants at each of our three research sites to attain the required sample size. For analysis of rates of AMR in NG, our power will be limited for very rare outcomes (eg, cefixime resistance), but we will have 80% power to detect an increase in tetracycline resistance from 80% to 86.7% or from 85% to 90.8% in each intervention arm compared to the control arm.

### Statistical Analysis

The main trial analyses will follow intention-to-treat principles, including participants whether or not they accept or continue PPT or doxyPEP. Of note, in a small pilot program of ≈80 MSM cohort participants at a research clinic on the Kenyan coast in 2018-2019, acceptability of PPT at quarterly visits was over 90%. Bacterial STI diagnoses (NG, CT, and early syphilis) will be reported at baseline and each follow-up visit by arm and by study site. The combined STI outcome will be compared between each intervention and the common control arm by estimating the relative risks of any STI over follow-up (months 3-18) using a modified Poisson model fitted with generalized estimating equations (GEE) methods to account for repeated observations for each participant, assuming an independent covariance structure, with site and study arm as the only covariates [78]. The test for significance will use a 2-sided α=.05, and 95% CIs will be calculated using robust standard errors. The same approach will be used for each STI individually.

Appropriate missing data methods (eg, imputation) or causal estimation of direct effects will be conducted to assess the potential influence of differential missingness by arm. Syndromic infections diagnosed and treated at interim visits after 28 days will be defined as infections for the current quarter and handled in the analysis as if they were present at the next scheduled visit. Per-protocol analyses will also be conducted, as well as analyses controlling for potential confounders such as HIV status if they are unbalanced across arms. Resistance to each antibiotic assessed (ie, penicillin, tetracycline, cefixime, azithromycin, and doxycycline) among NG cases will be tabulated by study arm, research site, site of infection, and AMR assessment method. The odds of resistance among NG cases with resistance results will be compared between each intervention and the common control arm using logistic regression, with repeated measures GEE if repeat infections occur. We will also analyze nonparametric trends over time in the proportion of NG isolates demonstrating resistance to each antibiotic in the study overall and by study arm. Additional analyses with adjustment for baseline STI, sexual risk behavior, and HIV status will also be performed.

### Implementation Outcomes

**Figure 2:**
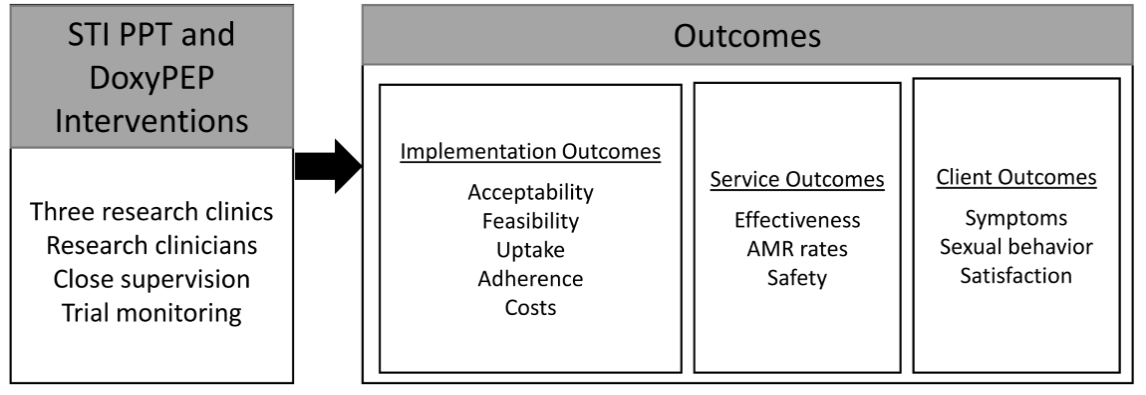
Conceptual model based on Proctor’s Implementation Science Framework. The conceptual model for assessment of each intervention tested includes assessment of implementation outcomes (acceptability, feasibility, uptake, adherence, and cost), service outcomes (effectiveness, antimicrobial resistance [AMR] rates, and safety), and client outcomes (symptoms, sexual behavior, and satisfaction). doxyPEP: doxycycline post-exposure prophylaxis; PPT: periodic presumptive treatment; STI: sexually transmitted infection.

Considering the unique needs of Kenyan MSM with respect to sexual health will be essential to developing effective strategies to address their STI burden. In addition, any STI control intervention must be acceptable, feasible, and safe for the patient. We will use a mixed methods approach to measure acceptability, feasibility, and safety of these interventions, using a conceptual model based on Proctor’s Implementation Science Framework [79,80]. Figure 2 presents the conceptual model for this work, which includes assessment of implementation outcomes (acceptability, feasibility, uptake, adherence, and cost), service outcomes (effectiveness, AMR rates, and safety), and client outcomes (symptoms, sexual behavior, and satisfaction). While the main trial analysis will focus on effectiveness and AMR rates, we will also collect acceptability, feasibility, and safety data from providers and from participants in all arms. Secondary outcomes related to the PPT and doxyPEP interventions that will be assessed include uptake and adherence (related to acceptability and sustainability of the interventions) and client outcomes including satisfaction, symptoms, and sexual behavior. The quantitative and qualitative measures we will use to assess these outcomes are presented in Table 1 [79]. Participants will be asked questions about acceptability (the 4-item Acceptability of Intervention Measure), feasibility (the 4-item Feasibility of Intervention Measure), and doxyPEP uptake and adherence in the ACASI instrument (Multimedia Appendix 1). In addition, their eligibility for and receipt of PPT (PPT arm participants) and acceptance of doxyPEP refills (doxyPEP arm participants), as well as symptoms reflecting potential side effects, will be captured in a clinical data form. To capture staff views and any challenges that arise, minutes will be taken at monthly staff meetings at each site. At the end of the study, acceptability and feasibility for providers will also be assessed using the Acceptability of Intervention Measure and the Feasibility of Intervention Measure in a questionnaire administered to research staff [81].

**Table 1:** Conceptual model with implementation, service, and client outcome measures.

Outcome type and specific outcome		Approach	Result
**Implementation**			
	Acceptability	Brief questionnaire for eligible individuals who decline to enroll; ACASI^a^ questions to participants; brief exit interviews; staff meeting minutes and questionnaire	Understanding how well PPT^b^ and doxyPEP^c^ are received by and are perceived to meet the needs of the target population
	Feasibility	ACASI questions to participants; staff meeting minutes and questionnaire	Understanding how easily PPT and doxyPEP can be carried out
	Uptake	Acceptance of directly observed PPT or of doxycycline tablets at study visits	Estimated PPT and doxyPEP acceptance at baseline documented in clinic records
	Adherence	Administration records for PPT participants; ACASI questions on doxyPEP use and adherence; doxycycline levels in hair for doxyPEP participants	Estimated PPT and doxyPEP acceptance over time documented in clinic records
	Costs	Microcosting; time-motion surveys (described below)	Cost to system, cost to patient
**Service**			
	Effectiveness	Impact on STI^d^ prevalence and on AMR^e^ (trial results)	Reduction in STI prevalence relative to increased AMR
	Safety	Rigorous monitoring of safety, including social harms	Adverse events attributed to PPT or doxyPEP; discontinuation due to adverse events
**Client**			
	Symptoms	Data on symptoms of potential side effects	Understanding how symptoms differ by arm
	Sexual behavior	ACASI questions on sexual behavior and antiretroviral use; brief exit interviews	Understanding of any behavioral disinhibition with PPT or doxyPEP
	Satisfaction	Brief exit interviews	Understanding how well PPT and doxyPEP met the needs of the target population

^a^ACASI: audio computer-assisted self-interview.

^b^PPT: periodic presumptive treatment.

^c^doxyPEP: doxycycline post-exposure prophylaxis.

^d^STI: sexually transmitted infection.

^e^AMR: antimicrobial resistance.

### Quantitative Data Collection

Due to its enhanced privacy, ACASI will be the main method of data collection, in English, Dholuo, or Kiswahili. We have successfully used ACASI to capture data in multiple projects at our research sites [30,82-85]. Because participants occasionally have difficulties using ACASI, a research assistant will be available to assist participants if needed. Measures that will be collected are based on our conceptual model, extensive experience conducting research among Kenyan MSM, and published literature on correlates of sexual behavior, HIV and STI incidence, and medication adherence. Table 2 presents an overview of data elements, content, tools for assessment, frequency of assessment, and applicable study arm.

**Table 2:** Quantitative data elements collected from participants: content, collection method, frequency, and arm.

Category and data element		Method and content	Frequency and arm
Primary trial outcome variable: NG^a^, CT^b^, or early infection		Aptima NAAT^c^: pooled specimen (throat, rectal, and urine) positive for NG or CT; Syphilis testing: first positive rapid plasma reagin or a fourfold increase in nontreponemal titers) for early syphilis	All arms: quarterly and at interim visits for STI^d^ symptoms
**Secondary trial outcome variables**			
	Each infection individually	Same as above	All arms: quarterly and at interim visits for STI symptoms
	AMR^e^	Resistance to penicillin, tetracycline, cefixime, azithromycin, or doxycycline by culture of urethral discharge (symptomatic urethritis cases) or molecular testing (25% of all NG-positive samples)	All arms: quarterly and at interim visits for STI symptoms
**Implementation outcome variables**			
	Acceptability	ACASI^f^: Acceptability of Intervention Measure [81]	All arms: quarterly
	Feasibility	ACASI: Feasibility of Intervention Measure [81]	All arms: quarterly
	Uptake	Direct observation: administration of cefixime and azithromycin in PPT arm; acceptance of doxycycline in doxyPEP^g^ arm	Intervention arms: quarterly
	Adherence	ACASI: self-report questions on adherence to doxyPEP; direct observation of PPT as above; hair doxycycline levels.	Intervention arms: quarterly
	Safety including social harms	Study monitoring: adverse events and social harms reported by participants or study staff	All arms: quarterly
	Symptoms	ACASI: urethral or rectal symptoms since past visit; whether STI treatment was sought outside the study; side effects of medications	All arms: quarterly
	Sexual risk behavior	ACASI: anal intercourse, vaginal intercourse, condom use, partner numbers, partner HIV status, concurrency, sexual networks, transactional sex, intimate partner violence	All arms: quarterly
	PrEP or ART use and adherence	ACASI: self-reported use of and adherence to PrEP (if HIV-negative) or ART^h^ (if HIV-positive); program records: chart data to confirm use if consents to linkage	All arms: quarterly
	Study retention	Study records: scheduled visits completed within 1-month window	All arms: quarterly
**Potential correlates of study outcomes**			
	Demographics	ACASI: age, education, religion, marital status, sexual orientation, employment	All arms: baseline
	Clinic access	ACASI: transportation costs, wait times	All arms: quarterly
	STI risk perception	ACASI: “What do you think is your risk of getting a sexually transmitted infection?” (4 item Likert scale)	All arms: quarterly
	HIV status	ACASI: self-reported HIV status; Program Records: programmatic test results if consents to linkage	All arms: quarterly
	Antibiotic use	ACASI: antibiotic use outside the study in the past 3 months	All arms: quarterly
	Substance use	ACASI: Alcohol Use Disorders Identification Test [86,87], Drug Abuse Screening Test [88]	All arms: quarterly
	Childhood abuse	ACASI: Childhood Experience of Care and Abuse [89]	All arms: baseline
	Sexual stigma	ACASI: sexual stigma, as in our previous work [90,91]	All arms: every 6 months
	Depressive symptoms	ACASI: Patient Health Questionnaire-9 [92]	All arms: quarterly

^a^NG: *Neisseria gonorrhoeae.*

^b^CT: *Chlamydia trachomatis.*

^c^NAAT: nucleic acid amplification testing.

^d^STI: sexually transmitted infection.

^e^AMR: antimicrobial resistance.

^f^ACASI: audio computer-assisted self-interview.

^g^doxyPEP: doxycycline post-exposure prophylaxis.

^h^ART: antiretroviral therapy.

### Qualitative Data Collection

During study screening, a brief questionnaire with 2 open-ended questions will be used to capture reasons for declining to enroll, including poor individual-level acceptability of PPT, doxyPEP, or randomization. During the trial, qualitative data collection will include brief exit interviews with a subset of 30 participants in each intervention arm (10 at each site), to understand participant-level acceptability and satisfaction with the interventions. Interviews will be conducted using participants’ preferred language (English, Dholuo, or Kiswahili). Participants will be sampled for these interviews to include a range of ages and reported risk behaviors (eg, transactional sex) using a stratified-purposive sampling approach. At any visit on which a participant withdraws, a similar exit interview will be conducted to obtain feedback on the intervention received. Interviews will be recorded if permission is granted; detailed notes will also be taken to document participant feedback. The topic guide for these interviews is included in Multimedia Appendix 2.

### Quantitative Analysis of Implementation Outcomes

GEE with appropriate links and robust standard errors will be used to test retention across all study arms and to evaluate associations between study arm and the quantitative implementation outcomes collected for both interventions (eg, acceptability and feasibility measures, uptake, adverse events, symptoms, and sexual risk behavior). Factors associated with adherence to doxyPEP will be analyzed in the population for whom hair samples are tested, based on semiquantitative assessment of high versus low doxycycline concentrations [63]. For all quantitative analyses, patterns of missing data will be evaluated, and multiple imputation will be used where appropriate.

### Qualitative Analysis of Implementation Outcomes

Exit interview notes and transcripts and staff meeting minutes will be translated into English if necessary and imported into Dedoose (SocioCultural Research Consultants) qualitative analysis software. These documents will be reviewed separately by 2 investigators for completeness and initial theme generation. Coding and analysis will focus on participant-level acceptability and satisfaction and on staff-level acceptability and feasibility, starting with a deductive approach informed by the theoretical framework of acceptability, a recently developed approach to acceptability research of medical interventions [93]. The components of the theoretical framework of acceptability are affective attitude, burden, ethicality, intervention coherence, opportunity costs, perceived effectiveness, and self-efficacy. Additional codes emerging from an inductive process will be added as they arise. Codes will be reviewed for consistency and intercoder agreement with consensus code generation by themes. Descriptive thematic analyses will be done separately for individuals who refuse participation, trial participants (including those who withdraw), and staff. Within these groupings, results will be triangulated with relevant quantitative data using joint displays.

### Cost Analysis

During the field work, we will conduct microcosting, semistructured interviews or focus group discussions with staff regarding time spent on different work activities, and time and motion observation of the intervention and standard of care treatments within the randomized controlled trial. Costs will be divided into direct costs of diagnostic evaluation or testing, direct costs of treatment, and indirect costs to patients. Research time (eg, informed consent) will be removed from programmatic costs. We will estimate the incremental cost per participant as the total annual program costs divided by the number of participants. The topic guide for staff is included in Multimedia Appendix 3.

### Modeling Impact

To assess impact, we will develop a stochastic, network-based model of NG, CT, and HIV transmission in Kenya. Because syphilis is relatively uncommon among Kenyan MSM and its transmission has not yet been extensively modeled, our model will not include syphilis. The general modeling approach will follow that developed by Jenness et al [94] for modeling NG, CT, and HIV transmission among MSM in the United States. The model will simulate a population of 100,000 individuals distinguished by the following attributes: age; partner preference; sexual role preference (insertive, receptive, or versatile); relationship status; NG, CT and HIV status; diagnosis status; time since infection; treatment status; and HIV viral load, among possible relevant others revealed by our fieldwork. Individuals can undergo any or all of the following transitions: enter the sexually active population; age; form or dissolve relationships of different types; become infected with NG, CT, or HIV; experience STI or HIV symptoms; test; become diagnosed; undergo treatment; discontinue ART treatment; reinitiate ART treatment; age out of the population; or die due to background age-specific mortality or HIV-related mortality. Within each partnership, individuals will engage in anal intercourse with coital frequency based on a Poisson model fit to study participant data, use condoms based on a binomial model also fit to study participant data, choose a sexual role (insertive or receptive) for each act of anal intercourse based on individual preferences, and disclose STI or HIV status. Network dynamics will be modeled using the *statnet* package in R to estimate exponential-family random graph models and their dynamic extension temporal exponential-family random graph models, which provide a foundation for statistically principled simulation of local and global network structure given a set of target statistics from empirical data [95]. Data sources and parameters will be based on the latest available literature at the time of model building. Sensitivity analyses will be conducted, especially around key parameters of the individual-level intervention efficacy. Comparison will also be made to NAAT-guided treatment as done in well-resourced settings. Model outcomes will include life-years lived with untreated NG or CT, incident NG and CT cases averted, and disability-adjusted life years averted.

### Cost-Effectiveness Analysis

After the trial is completed, mathematical models will be used to simulate long-term health and cost outcomes based on study data and to consider clinical outcomes beyond the scope and time horizon of the trial. Using cost and effectiveness outcomes estimated by the model, we will calculate the cost-effectiveness of PPT and of doxyPEP as interventions. The cost-effectiveness analysis will follow WHO guidelines and Cost of Implementing New Strategies recommendations [96,97], and report summary estimates that allow comparison to standard-care syndromic management. In addition, a comparison to NAAT-guided treatment (as done in well-resourced settings) will be made. Results of the cost-effectiveness analysis will provide important information to decision makers charged with defining priorities and allocating resources.

We will combine effectiveness estimates from the updated stochastic model with the cost estimates collected from each intervention to calculate cost per NG or CT case identified and treated and the incremental cost-effectiveness ratio measured as cost per incident NG or CT case averted and cost per disability-adjusted life year averted. Disability weights for calculation of disability-adjusted life years will be obtained from the latest global burden of disease study [98]. We will calculate the incremental cost-effectiveness ratio associated with PPT and with doxyPEP as the ratio of difference in costs divided by difference in disability-adjusted life years compared to the next most costly strategy over a 10-year horizon. Following updated guidelines recommending use of a supply-side threshold to reflect the opportunity cost of additional health investment, PPT and doxyPEP will be considered cost-effective if the incremental cost-effectiveness ratio is ≤US $500 per disability-adjusted life year averted [99]. We will use both the governmental and societal perspectives. Univariate and probabilistic sensitivity analyses will be performed to determine model robustness and the impact of varying different parameters through their plausible ranges on the estimate of cost-effectiveness.

### Data Safety and Monitoring

Adverse events will be recorded in study documentation, and serious adverse events will be reviewed for relatedness to study products or procedures by the study leadership team (the principal investigator of record and a lead investigator at each study site). Social impacts and any harms of study participation will also be assessed. An independent data safety and monitoring board composed of an experienced trial biostatistician and 2 internationally regarded clinical researchers, one Kenyan and the other Canadian, has been established to review study progress, including participant accrual, visit attendance, retention, and collection of primary and secondary endpoint data. This board will also review safety and outcome data in a closed report on a biannual basis to ensure safety and equipoise. The data safety and monitoring board will make recommendations based on their review of safety and efficacy data at biannual meetings. The trial will be terminated early if the incidence of serious adverse events related to either intervention exceeds 2% and is statistically significantly higher than the rate observed in the standard care arm (*P*<.01) in 2 consecutive interim (ie, every 6 months) safety analyses.

### Ethical Considerations

The protocol, consent forms, participant education and recruitment materials, and any subsequent modifications were reviewed and approved by the Kenyan Medical Research Institute’s Ethical Review Committee (Protocol 4962) and the Human Subjects Division of the University of Washington (STUDY00018588). The trial protocol (currently version 1.1, dated June 10, 2024) and any approved modifications will be posted at ClinicalTrials.gov. Trained research staff obtain written informed consent from all trial participants, for all exit interviews, and from staff who participate in feedback on acceptability and feasibility of the study interventions, following standardized protocols. Confidentiality of personal information is strictly maintained. Participant identifiers are not recorded in the study database but are included in a link log at each site. The link log is used to record participant names, contact information, and study ID numbers and will be maintained until participant enrollment and follow-up are complete for the purposes of avoiding duplicate enrollments, assisting with retention, and tracking participants with positive syphilis results. Participants are reimbursed 1000 KSh (approximately US $7.72) per visit. Clinical trial insurance and protocols to address social harms are in place to mitigate any adverse events resulting from trial participation.

## Results

During community engagement activities in preparation for the trial, the study was named the “*Mambo Matatu*” (Swahili for “three ways”) study by the gay, bisexual, and other MSM communities served by our research clinics. Regulatory approval was obtained from the Kenya Pharmacy and Poisons Board in December 2024, and a research permit was obtained from the Kenyan National Commission for Science, Technology, and Innovation in March 2025. After a delay due to late receipt of year 2 funding from the US National Institutes of Health, enrollment commenced on October 29, 2025. As of November 25, 2025, a total of 357 participants (12.3% of target) have been enrolled, including 133 in Kisumu, 122 in Nairobi, and 102 in Mombasa. Full enrollment is expected to take 6 months, with follow-up occurring over 18 months per participant. Results will be published in 2028. Table 3 presents the participant timeline as a schedule of enrollment, interventions, and assessments.

**Table 3:** Participant timeline: schedule of enrollment, interventions, and assessments.

Category		Trial period timepoints^a^								
		Enrollment		Postrandomization						Close-out
		-t_i_ to 0^b^	Month 0	Month 3	Month 6	Month 9	Month 12	Month 15	Month 18	
**Enrollment**										
	Eligibility screen	✓								
	Informed consent	✓								
	Locator information	✓								
	Randomization		✓							
**Intervention or comparator**										
	doxyPEP^c^ provision^d^		✓	✓	✓	✓	✓	✓	✓	
	PPT^e^		✓	✓	✓	✓	✓	✓	✓	
	Standard care^f^		✓	✓	✓	✓	✓	✓	✓	
**Assessments**										
	ACASI Survey		✓	✓	✓	✓	✓	✓	✓	
	Symptom review and clinical exam		✓	✓	✓	✓	✓	✓	✓	
	Specimen collection		✓	✓	✓	✓	✓	✓	✓	
	Syndromic treatment if indicated		✓	✓	✓	✓	✓	✓	✓	
	Syphilis treatment if indicated		✓	✓	✓	✓	✓	✓	✓	
	Adverse event monitoring		✓	✓	✓	✓	✓	✓	✓	
	Exit interview								✓	

^a^Acceptable visit window ±30 days.

^b^Pre-enrollment.

^c^doxyPEP: doxycycline post-exposure prophylaxis.

^d^Supply of 60 capsules of doxycycline are provided at each scheduled visit, with refills provided upon request between visits.

^e^Periodic presumptive treatment; provided when participant meets eligibility criteria (ie, having had condomless anal sex and either multiple sex partners or a sex partner with a sexually transmitted infection (STI) in the past 6 months at baseline [32] or in the past 3 months at follow-up visits).

^f^Limited to syndromic treatment when symptoms of an STI are present. Note that syndromic treatment is provided to participants in each study arm per Kenyan STI treatment guidelines when indicated.

## Discussion

### Overview

This study of two interventions to control bacterial STIs including CT, NG, and syphilis among MSM in African settings will yield important insights into how to improve STI control in resource-limited settings while balancing benefits, risks, and costs. The potential benefits of implementing either doxyPEP or PPT for the control of CT, NG, and syphilis among MSM in African settings are substantial; at the same time, these strategies are untested, and potential risks—including adverse events, increased AMR, risk compensation impacting sexual behavior, and increased costs—could counteract these benefits. Only a well-designed randomized trial comparing these two interventions to a standard care arm can help address these questions and determine whether the benefits outweigh risks of adopting either strategy. While we will be able to compare the two interventions to each other in addition to the primary analysis comparing each intervention to standard care, it is the comparison to standard care that is of greatest interest for policymakers, who will need to consider not only each intervention’s effectiveness, but also its acceptability, feasibility, and disadvantages in terms of safety, AMR, and costs relative to the current standard of care in determining the overall value added if either intervention is adopted.

We have carefully considered our study design and research approach to optimize feasibility while maintaining high rigor and ensuring that results will be reproducible. Kenya is an ideal setting for evaluating PPT and doxyPEP among MSM, as the Government of Kenya’s visionary 20-year plan for evidence-based HIV and STI prevention, the *Kenya HIV Prevention Revolution Road Map: Count Down to 2030*, aims to drive new HIV infections toward zero between 2013 and 2030 using effective HIV and STI prevention initiatives [100]. As HIV transmission decreases, risk has become more concentrated in key populations such as MSM, who also experience high rates of bacterial STIs. The Kenyan Ministry of Health endorses STI control in key populations, has worked to address health disparities among MSM, and supports this proposal, ensuring that our trial results will be linked to policy. In addition, each research site has collaborative relationships with MSM-led community-based organizations with large membership rosters, laying the groundwork for community feedback on the proposed work and its implementation and for successful outreach, recruitment, and retention.

We are using an oral regimen of cefixime and azithromycin instead of the Centers for Disease Control and Prevention’s currently recommended STI treatment regimen consisting of ceftriaxone by intramuscular injection and doxycycline by mouth [64]. We decided against the use of this regimen due to concerns about low acceptability of regular injections for presumptive treatment and because most low- and middle-income countries still using cefixime and azithromycin have not seen the same levels of AMR as in high-income countries, likely due the limitation of treatment to syndromic infections [101,102]. Azithromycin provides both CT coverage and dual coverage of most NG. Although azithromycin resistance can emerge in NG due to the long half-life of this drug [103,104], data from the NG surveillance programs at the WHO and Centers for Disease Control and Prevention suggest that cefixime resistance decreased after the addition of azithromycin to the NG treatment regimen [105,106]. In contrast, a recent study of our isolates in Kenya suggested that doxycycline can select for plasmid-mediated penicillin resistance only or both plasmid-mediated penicillin resistance and high-level plasma-mediated tetracycline resistance and therefore could potentially increase the risk of cephalosporin resistance [42]. Therefore, it is critical to evaluate NG AMR to all 3 antibiotics in all study arms.

Of note, the WHO did not specify a frequency for PPT assessment and delivery in their recommendations [4]. We debated offering this intervention on a 6-month basis, since a 6-month recall period is used for eligibility, but felt this might weaken the intervention’s efficacy. We will therefore provide PPT at each quarterly visit on which participants in the PPT arm are eligible and assess eligibility for PPT based on sexual behavior in the past 3 months. We anticipate being able to explore the potential efficacy of different frequencies of PPT administration in our modeling work. A consideration for both PPT and doxyPEP is the treatment of uninfected individuals. In our prior work, we have estimated a number needed to treat one infection with PPT ranging from 4-12 [31,32]. In the DoxyPEP study in US settings with similar STI incidence as in Kenya, the number needed to treat was estimated at 5 [28]. Treatment of uninfected individuals occurs in the setting of epidemiologic treatment of partners; for example, a recent study estimated that 65% of individuals receiving expedited partner treatment (often with cefixime by mouth) are asymptomatic and NAAT negative [107]. An important aspect of the proposed research is delivery of PPT or doxyPEP in a setting in which the vast majority of bacterial STIs are currently undiagnosed and therefore left untreated.

This study is particularly timely given recent evidence related to doxyPEP in particular and to STI control in general that has emerged. Recent publications have documented that presumptive treatment is common even in settings with NAAT and that both overtreatment and undertreatment occur at varying rates across studies and care settings [108,109]. In settings where NAAT is possible and follow-up is high, evidence supports testing first to avoid unnecessary ceftriaxone use for STI symptoms in MSM [110] and to avoid unnecessary antibiotic treatment in asymptomatic partners exposed to CT or NG [111]. Rising concerns about frequent NAAT and high levels of antibiotic consumption among MSM led to a randomized trial in Belgium that compared quarterly 3-site screening to not screening of asymptomatic MSM for NG and CT at all [112]. This “Gonoscreen” study demonstrated that frequent screening lowered CT rates but led to higher antibiotic consumption and had no effect on NG incidence [112]. Available evidence suggests that mass treatment of CT, NG, and syphilis in high prevalence populations such as MSM has only resulted in temporary reductions in the prevalence of these infections while likely increasing AMR [113]. DoxyPEP has, not unexpectedly, been associated with increases in antimicrobial resistance in NG as well as in other organisms present in the microbiome [55,114-116], leading to concern about the long-term benefits and harms of widespread use [117-119]. For example, the estimated percentage of NG isolates carrying the plasmid-borne gene *tetM*, which confers high-level tetracycline resistance, increased from less than 10% in 2020 to more than 30% in the first quarter of 2024 [120]. While modeling has shown that doxyPEP could avert a substantial portion of all STI diagnoses among at-risk MSM [121], a study that modeled the impact of doxyPEP on NG found that after initial decreases in NG prevalence and incidence, resistance would spread quickly, resulting in the total loss of doxyPEP’s clinical efficacy against NG within 10-20 years [122]. While continued impact of doxyPEP on CT and syphilis [123] will likely provide benefit for this strategy, evaluating other possible interventions, such as PPT, that are less costly and would lead to less overall antibiotic consumption is an important consideration.

While there are many important strengths of this study, it is not without limitations. First, selection bias is likely given that we will enroll MSM who accept research screening and participation. To mitigate this bias, we will collect data on characteristics of those who decline trial participation. Second, our results may not be generalizable to all geographic areas of Kenya, although data from earlier studies among MSM in Kenya suggest that the behavior risk profiles, sexual networks, and social interactions of MSM across the 3 sites are similar. Third, contamination can be a problem, so we will collect data on receipt of antibiotics in the past 30 days to monitor for this, especially in the standard care arm. Fourth, providing PPT or doxyPEP to intervention arm participants may reduce STI rates in the control arms by reducing NG and CT prevalence within sexual networks. We have data on STI prevalence at these sites prior to the study, which we can use to identify temporal decreases in STI rates [124]. Fifth, data we collect on stigmatized behaviors, such as condomless receptive anal intercourse and substance use, may be underreported due to social desirability bias. Our experience with ACASI in prior studies suggests that this method of data collection may yield more frank responses to questions on sensitive topics [82]. Sixth, there may be differential retention given the open-label design. We will try to address attrition bias by minimizing the burden of study visits and of each intervention on participants, keeping procedures within a reasonable timeframe, and monitoring clinic flow. Finally, we have not included testing for resistance in other organisms such as *Staphylococcus aureus* or *Streptococcus pneumoniae* due to a ceiling on our allowed budget. We will seek additional funding for additional testing of stored swabs and hair samples to explore the relationship between PPT and doxyPEP and AMR in off-target bacteria.

### Conclusions

The prevalence and incidence of bacterial STIs, including syphilis, NG, and CT infections, among MSM remain high in resource-limited settings such as Kenya. Results of this trial will provide critical data needed to inform guidelines to improve STI control among MSM in sub-Saharan Africa and other resource-limited settings where NAAT is not routinely available. Trial data will be used to model the health and economic impact of scaling up WHO-recommended PPT and doxyPEP compared to standard care on STI control among MSM and their partners in Kenya using a stochastic, agent-based model and standard cost-effectiveness evaluation. Modeled estimates of the health and economic impact of scaling up these two interventions on STI control among MSM and their partners in Kenya will provide critical information to guide policymakers.

## Data Availability

All data generated or analyzed during this study are included in this published article.

## Appendix

Multimedia Appendix 1 REDCap project with all data forms.

Multimedia Appendix 2 Exit interview topic guide.

Multimedia Appendix 3 Staff feedback guide.

Multimedia Appendix 4 Peer-review report from HSQE Health Services: Quality and Effectiveness Study Section, Healthcare Delivery and Methodologies Integrated Review Group (National Institutes of Health, USA).

## Abbreviations

ACASI: audio computer-assisted self-interview
AMR: antimicrobial resistance
ART: antiretroviral therapy
CONSORT: Consolidated Standards of Reporting Trials
CT: Chlamydia trachomatis
doxyPEP: doxycycline post-exposure prophylaxis
GEE: generalized estimating equations
MSM: men who have sex with men
NAAT: nucleic acid amplification testing
NG: Neisseria gonorrhoeae
PPT: periodic presumptive treatment
PrEP: pre-exposure prophylaxis
REDCap: Research Electronic Data Capture
STI: sexually transmitted infection
WHO: World Health Organization
