# Supplementary material for: World Health Organization–Recommended Periodic Presumptive Treatment Versus Doxycycline Post-Exposure Prophylaxis for Sexually Transmitted Infection Control Among Men Who Have Sex With Men in Kenya: Protocol for a Randomized Controlled Trial
Source: JMIR Res Protoc. 2026 Jan 6;15:e81113. doi: 10.2196/81113 (PMC12820545; doi:10.2196/81113)
Supplement: Multimedia Appendix 1 [file resprot_v15i1e81113_app1.pdf]

Data Dictionary Codebook

Mambo Matatu (PID: 104786)

11/25/2025 9:27am

Instruments

Instrument	Form Name	Events
Eligibility Form	eligibility_form	screening_and_cons_arm_1
Consent Form	consent_form	screening_and_cons_arm_1
Randomization Form	randomization_form	randomization_arm_1
Participant ACASI Form	participant_acasi_form	baseline_visit_arm_1<br>3_month_visit_arm_1<br>6_month_visit_arm_1<br>9_month_visit_arm_1<br>12_month_visit_arm_1<br>15_month_visit_arm_1<br>18_month_visit_arm_1
Clinical Case Report Form	clinical_case_report_form	baseline_visit_arm_1<br>3_month_visit_arm_1<br>6_month_visit_arm_1<br>9_month_visit_arm_1<br>12_month_visit_arm_1<br>15_month_visit_arm_1<br>18_month_visit_arm_1
Interim Visit Form	interim_visit_form	interim_visit_arm_1

Events

Event Name	Unique event name	Event ID
Screening and Consent	screening_and_cons_arm_1	937074
Randomization	randomization_arm_1	941660
Baseline Visit	baseline_visit_arm_1	941662
3 Month Visit	3_month_visit_arm_1	941664
6 Month Visit	6_month_visit_arm_1	941666
9 Month Visit	9_month_visit_arm_1	941668
12 Month Visit	12_month_visit_arm_1	941670
15 Month Visit	15_month_visit_arm_1	941672
18 Month Visit	18_month_visit_arm_1	941674
Interim visit	interim_visit_arm_1	944270

Languages

ID	Display Name
DH	☐ Dholuo
EN	☑ English **(default)**
SW	☐ Swahili

#	Variable / Field Name	Field Label<br>*Field Note*	Field Attributes (Field Type, Validation, Choices, Calculations, etc.)
Instrument: **Eligibility Form** (eligibility_form)<br>Active languages: SW			
1	[study_id]	Study ID	text
2	[id_check_elig]	Please enter participant ID again carefully. This must match the study ID of the record you created for this participant.	text, Required
3	[id_confirm_elig]	Confirmation that record and entered ID match	calc<br>Calculation: if([study_id]=[id_check_elig], 1, 0)<br>Field Annotation: @HIDDEN
4	[id_problem_elig]<br>Show the field ONLY if:<br>[id_confirm_elig]=0	WARNING: Entered study ID does not match REDCap Record Number. Check study ID carefully and reenter if you made a typo.If the study ID you entered is correct, please return to the Record Status Dashboard and open the correct record.	descriptive
5	[screen_date]	Date of eligibility screening	text (date_dmy), Required
6	[staff_id]	Initials of staff conducting eligibility screen	text, Required
7	[repeat_screen]	Has the participant been screened previously?	yesno, Required<br>1 Yes<br>0 No
8	[rescreen_id]<br>Show the field ONLY if:	Please enter the previous study ID under which the participant was screened.	text

	[repeat_screen]=1		
9	[eligibility_language]	What is the patient's preferred language?	radio, Required<br>EN English<br>SW Kiswahili<br>DH Dholuo
10	[sw_consent_prompt]<br><br>Show the field ONLY if:<br>[eligibility_language]='SW'	Swahili verbal consent for screening (please read to participant)<br><br>Asante kwa nia yako katika utafiti tunaofanya kuhusu kupunguza mzigo wa magonjwa ya ngono miongoni mwa wanaume wanaofanya ngono na wanaume wenzao na wanaofanya ngono na wanaume na wanawake nchini Kenya. Hatuhitaji jina lako au maelezo ya mawasiliano ili kuona kama umehitimu kushiriki. Je, ni sawa nikiuliza maswali machache ili kuona kama unahitimu kushiriki? Hii itachukua kama dakika moja.	descriptive
11	[dh_consent_prompt]<br><br>Show the field ONLY if:<br>[eligibility_language]='DH'	Dholuo verbal consent for screening (please read to participant)<br><br>Ero kamano kuom chiwori mar donjo e nonro,ma watimo kuom duoko piny pek mag tuoche nyeye kuom chuo materore gi chuo wetegi, to gi chuo materore kod chuo wetegi gi mine, to gi chuo mamoko e piny kenya. Ok wadwar nyingi kata yore ma inyalo yudi godo mondo eka wang'e ka bed ni inyalo donjo e nonro. Bende iyie mondo mi wapenji penjo matin mondo okonywa ng'eyo ka inyalo donjo e nonro? Penjo gi biro kawo madirom dakika achiel.	descriptive
12	[en_consent_prompt]<br><br>Show the field ONLY if:<br>[eligibility_language]='EN'	English verbal consent for screening (please read to participant)<br><br>Thank you for your interest in the study we are conducting on reducing the burden of sexually transmitted infections among gay and bisexual men and other men who have sex with men in Kenya. We don't need your name or contact information in order to see if you're eligible. Is it OK if I ask a few questions to see if you are eligible? This will take about one minute.	descriptive
13	[staff_permission]	Staff initials documenting verbal consent	text, Required
14	[dob]	Date of birth	text (date_dmy), Required, Identifier
15	[site]	Site	radio, Required<br>1 Kisumu<br>2 Nairobi<br>3 Mombasa
16	[calcage]	Calculated age	calc, Required<br>Calculation: rounddown(datediff([dob], 'today', 'y'))
17	[male_at_birth]	Assigned male sex at birth?	yesno, Required<br>1 Yes<br>0 No

18	[cisgender]	Identifies as male (cis-gender)?	yesno, Required<br>1 Yes<br>0 No
19	[cai_6_months]	Reports condomless anal intercourse with a man in the past 6 months?	yesno, Required<br>1 Yes<br>0 No
20	[mult_male]	More than one male sex partner in the past 6 months?	yesno, Required<br>1 Yes<br>0 No
21	[partner_sti]	Male sex partner with syndromic (urethritis, proctitis, or genital ulcer disease) or laboratory-diagnosed STI in the past 6 months?	yesno, Required<br>1 Yes<br>0 No
22	[willing_consent]	Willing and able to provide written informed consent and participate in all study procedures?	yesno, Required<br>1 Yes<br>0 No
23	[remain_18_months]	Planning to remain in the study area for 18 months?	yesno, Required<br>1 Yes<br>0 No
24	[age_ok]	Eligible based on age?	calc<br>Calculation: if(([calcage]>=18 and [calcage] <=29), 1, 0)
25	[include]	Eligible based on inclusion criteria?	calc<br>Calculation: if([age_ok]='1' and [male_at_birth]='1' and [cisgender]='1' and [cai_6_months]='1' and ([mult_male]='1' or [partner_sti]='1') and [willing_consent]='1' and [remain_18_months]='1', 1, 0)
26	[lack_understanding]<br>Show the field ONLY if: [include] = '1'	Unable to understand study procedures?	yesno, Required<br>1 Yes<br>0 No
27	[allergy]<br>Show the field ONLY if: [include] = '1'	Allergy to cephalosporin (cefixime), macrolide (erythromycin or azithromycin), or tetracycline (doxycycline) class antibiotics?	yesno, Required<br>1 Yes<br>0 No
28	[recent_abx]<br>Show the field ONLY if: [include] = '1'	Recent use of prolonged antibiotics (14 or more days in the month before enrollment)?	yesno, Required<br>1 Yes<br>0 No
29	[drug_interactions]<br>Show the field ONLY if: [include]='1'	Carefully review any medications the patient takes regularly and the list of drugs that interact with the Mambo Matatu study drugs.	descriptive<br>(Attachment: interaction table v2.docx, Display format: Link)
30	[drug_interaction]<br>Show the field ONLY if: [include] = '1'	Use of medications that impact cefixime, azithromycin, or doxycycline metabolism (check versus list in screening SOP)?	yesno, Required<br>1 Yes<br>0 No
31	[eligible]	Eligible for study	calc<br>Calculation: if([include]='1' and

			[lack_understanding]='0' and [allergy]='0' and [recent_abx]='0' and [drug_interaction]=0, 1, 0)
32	[staff_eligible]<br><br>Show the field ONLY if:<br>[eligible] = '1'	Individual is eligible for Mambo Matatu.<br><br>Please initial here and mark the form as "Complete" then proceed to administer informed consent.	text, Required
33	[not_eligible]<br><br>Show the field ONLY if:<br>[eligible] = '0'	Individual is not eligible for Mambo Matatu based on current responses. Please initial here and refer or counsel accordingly. If the reason for ineligibility is temporary, invite the participant to return in the future for repeat screening.<br><br>Please mark the form as "unverified" then click "Save and Exit Form."	text, Required
34	[eligibility_form_complete]	Section Header: *Form Status*<br>Complete?	dropdown<br>0 Incomplete<br>1 Unverified<br>2 Complete
**Instrument: Consent Form (consent_form)**<br>Active languages: None			
35	[id_check_consent]	Please enter participant ID again carefully. This must match the study ID of the record you have opened in order to proceed.	text, Required
36	[id_confirm_consent]	Confirmation that record and entered ID match	calc<br>Calculation: if([study_id]=[id_check_consent], 1, 0)<br>Field Annotation: @HIDDEN
37	[id_problem_consent]<br><br>Show the field ONLY if:<br>[id_confirm_consent]=0	WARNING: Entered study ID does not match REDCap Record Number. Check study ID carefully and reenter if you made a typo.If the study ID you entered is correct, please return to the Record Status Dashboard and open the correct record.	descriptive
38	[consent_language]<br><br>Show the field ONLY if:<br>[eligible] = '1'	In what language will you administer consent?	radio, Required<br>1 English<br>2 Kiswahili<br>3 Dholuo
39	[consent_eng]<br><br>Show the field ONLY if:<br>[eligible] = '1' and [consent_language] = '1'	English Consent	descriptive<br>(Attachment: Trial Informed Consent English.pdf, Display format: Link)
40	[consent_kiswahili]<br><br>Show the field ONLY if:<br>[eligible] = '1' and [consent_language] = '2'	Kiswahili consent	descriptive<br>(Attachment: Trial Informed Consent Kiswahili.pdf, Display format: Link)
41	[consent_dholuo]<br><br>Show the field ONLY if:<br>[eligible] = '1' and [consent_language] = '3'	Dholuo consent	descriptive<br>(Attachment: Trial Informed Consent Dholuo.pdf, Display format: Link)
42	[consent_given]	Do you agree to participate in this research study?	yesno, Required

	Show the field ONLY if: [eligible] = '1'		1 Yes<br>0 No
43	[contact_consent]<br><br>Show the field ONLY if: [eligible] = '1' and [consent_given] = '1'	Do you agree to provide contact information for follow-up?	yesno, Required<br>1 Yes<br>0 No
44	[fingerprint_consent]<br><br>Show the field ONLY if: [eligible] = '1' and [consent_given] = '1'	Do you agree to have your fingerprint scanned to help identify you in the research?	yesno, Required<br>1 Yes<br>0 No
45	[samples_data_consent]<br><br>Show the field ONLY if: [eligible] = '1' and [consent_given] = '1'	Do you agree to have your samples and data preserved for later study?	yesno, Required<br>1 Yes<br>0 No
46	[program_data_consent]<br><br>Show the field ONLY if: [eligible] = '1' and [consent_given] = '1'	Do you agree to link your program clinical records to this study?	yesno, Required<br>1 Yes<br>0 No
47	[program_id]<br><br>Show the field ONLY if: [eligible] = '1' and [consent_given] = '1' and [program_data_consent] = '1'	What is your program ID at the study site?<br><br>Note that this number is the Anza Mapema ID in Kisumu, the Hapa-Kenya ID in Mombasa, and the SWOP Clinic ID in Nairobi.	text, Identifier
48	[ship_consent]<br><br>Show the field ONLY if: [eligible] = '1' and [consent_given] = '1'	Do you agree to have your samples shipped to Seattle, USA for analysis?	yesno, Required<br>1 Yes<br>0 No
49	[end_enroll]<br><br>Show the field ONLY if: [eligible] = '1' and [consent_given] = '1'	That completes the enrollment process. Please mark the form as "complete" if done or as "unverified" if anything remains to be confirmed, then click "Save and Exit Form."	descriptive
50	[reason_not]<br><br>Show the field ONLY if: [consent_given] = '0'	Why did the participant choose not to enroll?	notes, Required
51	[feel_randomize]<br><br>Show the field ONLY if: [consent_given] = '0'	Some individuals may choose not to enroll because of concerns about being randomized. How does this individual feel about randomization to one of the three Mambo Matatu arms?	notes
52	[change_mind]<br><br>Show the field ONLY if: [consent_given] = '0'	Let the individual know that if they change their mind, they are welcome to come back and be screened again.<br><br>Please initial here, then mark the form as "unverified" then click "Save and Exit Form."	text
53	[consent_form_complete]	Section Header: *Form Status*<br>Complete?	dropdown<br>0 Incomplete<br>1 Unverified<br>2 Complete

Instrument: Randomization Form (randomization_form)

Active languages: None

54	[id_check_random]	Section Header: *Randomization*<br>Please enter participant ID again carefully. This must match the study ID of the record you have opened in order to proceed.	text, Required
55	[id_confirm_random]	Confirmation that record and entered ID match	calc<br>Calculation: if ([study_id]=[id_check_random], 1, 0)<br>Field Annotation: @HIDDEN
56	[id_problem_random]<br>Show the field ONLY if: [id_confirm_random]=0	WARNING: Entered study ID does not match REDCap Record Number. Check study ID carefully and reenter if you made a typo. If the study ID you entered is correct, please return to the Record Status Dashboard and open the correct record.	descriptive
57	[randomization_group]	Randomization Group	dropdown, Required<br>0 periodic presumptive treatment<br>1 doxyPEP<br>2 standard care
58	[randomization_form_complete]	Section Header: *Form Status*<br>Complete?	dropdown<br>0 Incomplete<br>1 Unverified<br>2 Complete

Instrument: Participant ACASI Form (participant_acasi_form) Enabled as survey

Active languages - Data Entry: DH, EN, SW \| Survey: DH, EN, SW

59	[id_check]	Section Header: *Participant Identification - STAFF TO COMPLETE THIS SECTIONATTENTION: For language selection, it is important to (1) click on the language desired in the upper right-hand corner of the screen and (2) select the same language in response to question 3. THESE MUST MATCH.*<br>Please enter participant ID carefully - this must match the study ID of the record you have opened in order to proceed.	text, Required
60	[id_confirm]	Confirmation that record and entered ID match	calc<br>Calculation: if([study_id]=[id_check], 1, 0)<br>Field Annotation: @HIDDEN
61	[instruct_id_problem]<br>Show the field ONLY if: [id_confirm]=0	Section Header:<br>WARNING: Entered study ID does not match REDCap Record Number. Check study ID carefully and reenter if you made a typo. If the study ID you entered is correct, please return to the Record Status Dashboard and open the correct record.	descriptive<br>(Attachment: Stop signs.jpg, Display format: Inline image/PDF)
62	[visit_date]	Section Header:<br>Visit Date	text (date_dmy), Required
63	[site_enrolled]	Study site	radio, Required<br>1 Kisumu<br>2 Nairobi<br>3 Mombasa
64	[acasi_language]	What language will the participant use?	radio, Required

			EN, English<br>SW, Kiswahili<br>DH, Dholuo
65	[nationality_eng]<br>Show the field ONLY if:<br>[event-name]='baseline_visit_arm_1' AND [acasi_language]='EN'	Section Header: *Sociodemographics and Sexual History (enrollment only)*	descriptive<br>(Attachment: Q1.mp3, Display format: Audio file (play in embedded player on page))
66	[nationality_sw]<br>Show the field ONLY if:<br>[event-name]='baseline_visit_arm_1' AND [acasi_language]='SW'		descriptive<br>(Attachment: SW-Q1.mp3, Display format: Audio file (play in embedded player on page))
67	[nationality_dh]<br>Show the field ONLY if:<br>[event-name]='baseline_visit_arm_1' AND [acasi_language]='DH'		descriptive<br>(Attachment: DH-Q1.mp3, Display format: Audio file (play in embedded player on page))
68	[nationality]<br>Show the field ONLY if:<br>[event-name]='baseline_visit_arm_1'	What is your nationality?<br>(Check all that apply)	checkbox, Required<br>1, nationality___1, Kenya<br>2, nationality___2, Uganda<br>3, nationality___3, Tanzania<br>4, nationality___4, Somalia<br>5, nationality___5, Ethiopia<br>6, nationality___6, South Sudan<br>7, nationality___7, Other (Specify)<br>8, nationality___8, Refuse to answer
69	[other_nationality]<br>Show the field ONLY if:<br>[nationality(7)]='1' AND [event-name]='baseline_visit_arm_1'	Other nationality (Specify)	text, Required
70	[residence_kisumu_eng]<br>Show the field ONLY if:<br>[event-name]='baseline_visit_arm_1' AND [site_enrolled] = 1 AND [acasi_language]='EN'		descriptive<br>(Attachment: Q2 Kisumu E.mp3, Display format: Audio file (play in embedded player on page))
71	[residence_kisumu_sw]<br>Show the field ONLY if:<br>[event-name]='baseline_visit_arm_1' AND [site_enrolled] = 1 AND [acasi_language]='SW'		descriptive<br>(Attachment: Q2 Kisumu K.mp3, Display format: Audio file (play in embedded player on page))
72	[residence_kisumu_dh]<br>Show the field ONLY if:<br>[event-name]='baseline_visit_arm_1' AND [site_en		descriptive<br>(Attachment: Q2 Kisumu L.mp3, Display format: Audio file (play in embedded player on page))

	rolled] = 1 AND [acasi_language]='DH'		
73	[residence_mombasa_eng]<br><br>Show the field ONLY if: [event-name]='baseline_visit_arm_1' AND [site_enrolled] = 3 AND [acasi_language]='EN'		descriptive (Attachment: Q2 Mombasa E.mp3, Display format: Audio file (play in embedded player on page))
74	[residence_mombasa_sw]<br><br>Show the field ONLY if: [event-name]='baseline_visit_arm_1' AND [site_enrolled] = 3 AND [acasi_language]='SW'		descriptive (Attachment: Q2 Mombasa K.mp3, Display format: Audio file (play in embedded player on page))
75	[residence_mombasa_dh]<br><br>Show the field ONLY if: [event-name]='baseline_visit_arm_1' AND [site_enrolled] = 3 AND [acasi_language]='DH'		descriptive (Attachment: Q2 Mombasa L.mp3, Display format: Audio file (play in embedded player on page))
76	[residence_nairobi_eng]<br><br>Show the field ONLY if: [event-name]='baseline_visit_arm_1' AND [site_enrolled] = 2 AND [acasi_language]='EN'		descriptive (Attachment: Q2 Nairobi E.mp3, Display format: Audio file (play in embedded player on page))
77	[residence_nairobi_sw]<br><br>Show the field ONLY if: [event-name]='baseline_visit_arm_1' AND [site_enrolled] = 2 AND [acasi_language]='SW'		descriptive (Attachment: Q2 Nairobi K.mp3, Display format: Audio file (play in embedded player on page))
78	[residence_nairobi_dh]<br><br>Show the field ONLY if: [event-name]='baseline_visit_arm_1' AND [site_enrolled] = 2 AND [acasi_language]='DH'		descriptive (Attachment: Q2 Nairobi L.mp3, Display format: Audio file (play in embedded player on page))
79	[residence]<br><br>Show the field ONLY if: [event-name]='baseline_visit_arm_1'	For how many years have you lived in [site_enrolled]? (Please state in years)	text (number, Min: 0, Max: 50), Required
80	[religion_eng]<br><br>Show the field ONLY if: [event-name]='baseline_visit_arm_1' AND [acasi_language]='EN'		descriptive (Attachment: Q3.mp3, Display format: Audio file (play in embedded player on page))

81	[religion_sw]<br><br>Show the field ONLY if:<br>[event-name]='baseline_visit_arm_1' AND [acasi_language]='SW'		descriptive<br>(Attachment: SW-Q3.mp3, Display format: Audio file (play in embedded player on page))
82	[religion_dh]<br><br>Show the field ONLY if:<br>[event-name]='baseline_visit_arm_1' AND [acasi_language]='DH'		descriptive<br>(Attachment: DH-Q3.mp3, Display format: Audio file (play in embedded player on page))
83	[religion]<br><br>Show the field ONLY if:<br>[event-name]='baseline_visit_arm_1'	What is your religion?	radio, Required<br>1 Muslim<br>2 Catholic<br>3 Protestant<br>4 Hindu<br>5 Indigenous African Church<br>6 No religion<br>7 Other (Specify)<br>8 Refuse to answer
84	[other_religion]<br><br>Show the field ONLY if:<br>[event-name]='baseline_visit_arm_1' and [religion] = '7'	Other religion (Specify)	text, Required
85	[education_eng]<br><br>Show the field ONLY if:<br>[event-name]='baseline_visit_arm_1' AND [acasi_language]='EN'	Section Header:	descriptive<br>(Attachment: Q4.mp3, Display format: Audio file (play in embedded player on page))
86	[education_sw]<br><br>Show the field ONLY if:<br>[event-name]='baseline_visit_arm_1' AND [acasi_language]='SW'		descriptive<br>(Attachment: SW-Q4.mp3, Display format: Audio file (play in embedded player on page))
87	[education_dh]<br><br>Show the field ONLY if:<br>[event-name]='baseline_visit_arm_1' AND [acasi_language]='DH'		descriptive<br>(Attachment: DH-Q4.mp3, Display format: Audio file (play in embedded player on page))
88	[education]<br><br>Show the field ONLY if:<br>[event-name]='baseline_visit_arm_1'	What is the highest grade level that you completed in school?	dropdown, Required<br>1 No education<br>2 Standard 1<br>3 Standard 2<br>4 Standard 3<br>5 Standard 4<br>6 Standard 5<br>7 Standard 6

			8 Standard 7<br>9 Standard 8<br>10 Form 1<br>11 Form 2<br>12 Form 3<br>13 Form 4<br>14 Form 5<br>15 Form 6<br>16 Incomplete college<br>17 Completed college<br>18 Incomplete University<br>19 Completed University<br>20 Refuse to answer
89	[student_eng]<br>Show the field ONLY if:<br>[event-name]='baseline_visit_arm_1' AND [acasi_language]='EN'		descriptive<br>(Attachment: Q5.mp3, Display format: Audio file (play in embedded player on page))
90	[student_sw]<br>Show the field ONLY if:<br>[event-name]='baseline_visit_arm_1' AND [acasi_language]='SW'		descriptive<br>(Attachment: SW-Q5.mp3, Display format: Audio file (play in embedded player on page))
91	[student_dh]<br>Show the field ONLY if:<br>[event-name]='baseline_visit_arm_1' AND [acasi_language]='DH'		descriptive<br>(Attachment: DH-Q5.mp3, Display format: Audio file (play in embedded player on page))
92	[student]<br>Show the field ONLY if:<br>[event-name]='baseline_visit_arm_1'	Are you currently a student enrolled in a school or an academic institution?	checkbox, Required<br>1 student___1 No<br>2 student___2 Yes<br>3 student___3 Refuse to answer
93	[employment_eng]<br>Show the field ONLY if:<br>[event-name]='baseline_visit_arm_1' AND [acasi_language]='EN'		descriptive<br>(Attachment: Q6.mp3, Display format: Audio file (play in embedded player on page))
94	[employment_sw]<br>Show the field ONLY if:<br>[event-name]='baseline_visit_arm_1' AND [acasi_language]='SW'		descriptive<br>(Attachment: SW-Q6.mp3, Display format: Audio file (play in embedded player on page))

95	[employment_dh]<br><br>Show the field ONLY if:<br>[event-name]='baseline_visit_arm_1' AND [acasi_language]='DH'		descriptive<br>(Attachment: DH-Q6.mp3, Display format: Audio file (play in embedded player on page))
96	[employment]<br><br>Show the field ONLY if:<br>[event-name]='baseline_visit_arm_1'	What best describes your current employment status?	radio, Required<br>1 Unemployed<br>2 Employed full time<br>3 Employed part time<br>4 Self-employed<br>5 Between jobs (casual)<br>6 Other (Specify)<br>7 Refuse to answer
97	[other_employment]<br><br>Show the field ONLY if:<br>[event-name]='baseline_visit_arm_1' and [employment]= '6'	Other employment (Specify)	text, Required
98	[sex_orientation_eng]<br><br>Show the field ONLY if:<br>[event-name]='baseline_visit_arm_1' AND [acasi_language]='EN'	Section Header:	descriptive<br>(Attachment: Q7.mp3, Display format: Audio file (play in embedded player on page))
99	[sex_orientation_sw]<br><br>Show the field ONLY if:<br>[event-name]='baseline_visit_arm_1' AND [acasi_language]='SW'		descriptive<br>(Attachment: Q7 K.mp3, Display format: Audio file (play in embedded player on page))
100	[sex_orientation_dh]<br><br>Show the field ONLY if:<br>[event-name]='baseline_visit_arm_1' AND [acasi_language]='DH'		descriptive<br>(Attachment: DH-Q7.mp3, Display format: Audio file (play in embedded player on page))
101	[sex_orientation]<br><br>Show the field ONLY if:<br>[event-name]='baseline_visit_arm_1'	How do you identify in terms of your sexual orientation	radio, Required<br>1 Bisexual<br>2 Gay/homosexual<br>3 Queer<br>4 Straight/heterosexual<br>5 MSM (man who has sex with men)<br>6 Other (Specify)<br>7 Refuse to answer
102	[other_orientation]<br><br>Show the field ONLY if:<br>[event-name]='baseline_visit_arm_1' and [sex_orientation] = '6'	Other orientation (Specify)	text, Required

103	[sex_attraction_eng]<br><br>Show the field ONLY if:<br>[event-name]='baseline_visit_arm_1' AND [acasi_language]='EN'		descriptive<br>(Attachment: Q8.mp3, Display format: Audio file (play in embedded player on page))
104	[sex_attraction_sw]<br><br>Show the field ONLY if:<br>[event-name]='baseline_visit_arm_1' AND [acasi_language]='SW'		descriptive<br>(Attachment: SW-Q8.mp3, Display format: Audio file (play in embedded player on page))
105	[sex_attraction_dh]<br><br>Show the field ONLY if:<br>[event-name]='baseline_visit_arm_1' AND [acasi_language]='DH'		descriptive<br>(Attachment: DH-Q8.mp3, Display format: Audio file (play in embedded player on page))
106	[sex_attraction]<br><br>Show the field ONLY if:<br>[event-name]='baseline_visit_arm_1'	Do you currently feel more sexually attracted to men or to women?	radio, Required<br>1 Only to women<br>2 More to women than to men<br>3 To women and men equally<br>4 More to men than to women<br>5 Only to men<br>6 Refuse to answer
107	[orientation_hidden_fam_eng]<br><br>Show the field ONLY if:<br>[event-name]='baseline_visit_arm_1' AND [acasi_language]='EN'		descriptive<br>(Attachment: Q9.mp3, Display format: Audio file (play in embedded player on page))
108	[orientation_hidden_fam_sw]<br><br>Show the field ONLY if:<br>[event-name]='baseline_visit_arm_1' AND [acasi_language]='SW'		descriptive<br>(Attachment: SW-Q9.mp3, Display format: Audio file (play in embedded player on page))
109	[orientation_hidden_fam_dh]<br><br>Show the field ONLY if:<br>[event-name]='baseline_visit_arm_1' AND [acasi_language]='DH'		descriptive<br>(Attachment: DH-Q9.mp3, Display format: Audio file (play in embedded player on page))
110	[orientation_hidden_fam]<br><br>Show the field ONLY if:<br>[event-name]='baseline_visit_arm_1'	In general, how hard do you try to keep your sexual orientation hidden from your family?	radio, Required<br>1 Try very hard<br>2 Try somewhat hard<br>3 Don't try but don't talk about it<br>4 I openly talk about it<br>5 Refuse to answer
111	[orientation_hidden_friends_eng]	Section Header:	descriptive<br>(Attachment: Q10.mp3, Display format:

	Show the field ONLY if: [event-name]='baseline_visit_arm_1' AND [acasi_language]='EN'		Audio file (play in embedded player on page))
112	[orientation_hidden_friends_sw]<br><br>Show the field ONLY if: [event-name]='baseline_visit_arm_1' AND [acasi_language]='SW'		descriptive<br>(Attachment: SW-Q10.mp3, Display format: Audio file (play in embedded player on page))
113	[orientation_hidden_friends_dh]<br><br>Show the field ONLY if: [event-name]='baseline_visit_arm_1' AND [acasi_language]='DH'		descriptive<br>(Attachment: DH-Q10.mp3, Display format: Audio file (play in embedded player on page))
114	[orientation_hidden_friends]<br><br>Show the field ONLY if: [event-name]='baseline_visit_arm_1'	In general, how hard do you try to keep your sexual orientation hidden from your friends?	radio, Required<br>1 Try very hard<br>2 Try somewhat hard<br>3 Don't try but don't talk about it<br>4 I openly talk about it<br>5 Refuse to answer
115	[orientation_hidden_work_eng]<br><br>Show the field ONLY if: [event-name]='baseline_visit_arm_1' AND [acasi_language]='EN'		descriptive<br>(Attachment: Q11.mp3, Display format: Audio file (play in embedded player on page))
116	[orientation_hidden_work_sw]<br><br>Show the field ONLY if: [event-name]='baseline_visit_arm_1' AND [acasi_language]='SW'		descriptive<br>(Attachment: Q11 K.mp3, Display format: Audio file (play in embedded player on page))
117	[orientation_hidden_work_dh]<br><br>Show the field ONLY if: [event-name]='baseline_visit_arm_1' AND [acasi_language]='DH'		descriptive<br>(Attachment: DH-Q11.mp3, Display format: Audio file (play in embedded player on page))
118	[orientation_hidden_work]<br><br>Show the field ONLY if: [event-name]='baseline_visit_arm_1'	In general, how hard do you try to keep your sexual orientation hidden at work or at school?	radio, Required<br>1 Try very hard<br>2 Try somewhat hard<br>3 Don't try but don't talk about it<br>4 I openly talk about it<br>5 Refuse to answer

119	[orientation_hidden_public_eng]<br><br>Show the field ONLY if:<br>[event-name]='baseline_visit_arm_1' AND [acasi_language]='EN'		descriptive<br>(Attachment: Q12.mp3, Display format: Audio file (play in embedded player on page))
120	[orientation_hidden_public_sw]<br><br>Show the field ONLY if:<br>[event-name]='baseline_visit_arm_1' AND [acasi_language]='SW'		descriptive<br>(Attachment: SW-Q12.mp3, Display format: Audio file (play in embedded player on page))
121	[orientation_hidden_public_dh]<br><br>Show the field ONLY if:<br>[event-name]='baseline_visit_arm_1' AND [acasi_language]='DH'		descriptive<br>(Attachment: DH-Q12.mp3, Display format: Audio file (play in embedded player on page))
122	[orientation_hidden_public]<br><br>Show the field ONLY if:<br>[event-name]='baseline_visit_arm_1'	In general, how hard do you try to keep your sexual orientation hidden when you are in public spaces?	radio, Required<br>1 Try very hard<br>2 Try somewhat hard<br>3 Don't try but don't talk about it<br>4 I openly talk about it<br>5 Refuse to answer
123	[ever_married_eng]<br><br>Show the field ONLY if:<br>[event-name]='baseline_visit_arm_1' AND [acasi_language]='EN'	Section Header:	descriptive<br>(Attachment: Q13.mp3, Display format: Audio file (play in embedded player on page))
124	[ever_married_sw]<br><br>Show the field ONLY if:<br>[event-name]='baseline_visit_arm_1' AND [acasi_language]='SW'		descriptive<br>(Attachment: SW-Q13.mp3, Display format: Audio file (play in embedded player on page))
125	[ever_married_dh]<br><br>Show the field ONLY if:<br>[event-name]='baseline_visit_arm_1' AND [acasi_language]='DH'		descriptive<br>(Attachment: DH-Q13.mp3, Display format: Audio file (play in embedded player on page))
126	[ever_married]<br><br>Show the field ONLY if:<br>[event-name]='baseline_visit_arm_1'	Have you ever been married to a female?	radio, Required<br>1 No<br>2 Yes<br>3 Refuse to answer
127	[living_female_eng]<br><br>Show the field ONLY if:<br>[event-name]='baseline_visit_arm_1' AND [acasi_language]='EN'		descriptive<br>(Attachment: Q14.mp3, Display format: Audio file (play in embedded player on page))

128	[living_female_sw]<br><br>Show the field ONLY if: [event-name]='baseline_visit_arm_1' AND [acasi_language]='SW'		descriptive<br>(Attachment: SW-Q14.mp3, Display format: Audio file (play in embedded player on page))
129	[living_female_dh]<br><br>Show the field ONLY if: [event-name]='baseline_visit_arm_1' AND [acasi_language]='DH'		descriptive<br>(Attachment: DH-Q14.mp3, Display format: Audio file (play in embedded player on page))
130	[living_female]<br><br>Show the field ONLY if: [event-name]='baseline_visit_arm_1'	Are you currently living with a female wife or female sexual partner?	radio, Required<br>1 No<br>2 Yes<br>3 Refuse to answer
131	[living_male_eng]<br><br>Show the field ONLY if: [event-name]='baseline_visit_arm_1' AND [acasi_language]='EN'		descriptive<br>(Attachment: Q15.mp3, Display format: Audio file (play in embedded player on page))
132	[living_male_sw]<br><br>Show the field ONLY if: [event-name]='baseline_visit_arm_1' AND [acasi_language]='SW'		descriptive<br>(Attachment: SW-Q15.mp3, Display format: Audio file (play in embedded player on page))
133	[living_male_dh]<br><br>Show the field ONLY if: [event-name]='baseline_visit_arm_1' AND [acasi_language]='DH'		descriptive<br>(Attachment: DH-Q15.mp3, Display format: Audio file (play in embedded player on page))
134	[living_male]<br><br>Show the field ONLY if: [event-name]='baseline_visit_arm_1'	Are you currently living with a male sexual partner?	radio, Required<br>1 No<br>2 Yes<br>3 Refuse to answer
135	[costeffective_prompt_eng]<br><br>Show the field ONLY if: [event-name]='3_month_visit_arm_1' AND [acasi_language]='EN'	Section Header: *Cost-Effectiveness (3 months, not repeated) Now we want to understand more about the time and effort it takes for you to attend your clinic visits along with the costs of attending. The next few questions will ask about your time and travel costs.*	descriptive<br>(Attachment: INST-Q16.mp3, Display format: Audio file (play in embedded player on page))
136	[costeffective_prompt_sw]<br><br>Show the field ONLY if: [event-name]='3_month_visit_arm_1' AND [acasi_language]='SW'		descriptive<br>(Attachment: SW-INST-Q16.mp3, Display format: Audio file (play in embedded player on page))
137	[costeffective_prompt_dh]<br><br>Show the field ONLY if:		descriptive<br>(Attachment: DH-INST-Q16.mp3, Display format: Audio file (play in embedded player on page))

	[event-name]='3_month_visit_arm_1' AND [acasi_language]='DH'		
138	[long_clinic_eng]<br><br>Show the field ONLY if:<br>[event-name]='3_month_visit_arm_1' AND [acasi_language]='EN'		descriptive<br>(Attachment: Q16.mp3, Display format: Audio file (play in embedded player on page))
139	[long_clinic_sw]<br><br>Show the field ONLY if:<br>[event-name]='3_month_visit_arm_1' AND [acasi_language]='SW'		descriptive<br>(Attachment: SW-Q16.mp3, Display format: Audio file (play in embedded player on page))
140	[long_clinic_dh]<br><br>Show the field ONLY if:<br>[event-name]='3_month_visit_arm_1' AND [acasi_language]='DH'		descriptive<br>(Attachment: DH-Q16.mp3, Display format: Audio file (play in embedded player on page))
141	[long_clinic]<br><br>Show the field ONLY if:<br>[event-name]='3_month_visit_arm_1'	Approximately how long did it take you to get to the clinic today?	dropdown, Required<br>1 Less than 15 minutes<br>2 15-30 minutes<br>3 30-45 minutes<br>4 45-60 minutes<br>5 60-90 minutes (1 to 1.5 hours)<br>6 90-180 minutes (1.5 to 2 hours)<br>7 More than 2 hours<br>8 Refuse to answer
142	[transport_clinic_eng]<br><br>Show the field ONLY if:<br>[event-name]='3_month_visit_arm_1' AND [acasi_language]='EN'		descriptive<br>(Attachment: Q17.mp3, Display format: Audio file (play in embedded player on page))
143	[transport_clinic_sw]<br><br>Show the field ONLY if:<br>[event-name]='3_month_visit_arm_1' AND [acasi_language]='SW'		descriptive<br>(Attachment: SW-Q17.mp3, Display format: Audio file (play in embedded player on page))
144	[transport_clinic_dh]<br><br>Show the field ONLY if:<br>[event-name]='3_month_visit_arm_1' AND [acasi_language]='DH'		descriptive<br>(Attachment: DH-Q17.mp3, Display format: Audio file (play in embedded player on page))
145	[transport_clinic]<br><br>Show the field ONLY if:<br>[event-name]='3_month_visit_arm_1'	How did you get to the clinic today?	radio, Required<br>1 Walk<br>2 Bicycle<br>3 Motorcycle<br>4 Matatu or bus<br>5 Car

			6 Other (Specify)<br>7 Refuse to answer
146	[transport_other]<br><br>Show the field ONLY if:<br>[event-name]='3_month_visit_arm_1' and [transport_clinic] = '6'	Other transport (Specify)	text, Required
147	[transport_cost_eng]<br><br>Show the field ONLY if:<br>[event-name]='3_month_visit_arm_1' AND ([transport_clinic]='3' or [transport_clinic]='4' or [transport_clinic]='5' or [transport_clinic]='6') AND [acasi_language]='EN'		descriptive<br>(Attachment: Q18.mp3, Display format: Audio file (play in embedded player on page))
148	[transport_cost_sw]<br><br>Show the field ONLY if:<br>[event-name]='3_month_visit_arm_1' AND ([transport_clinic]='3' or [transport_clinic]='4' or [transport_clinic]='5' or [transport_clinic]='6') AND [acasi_language]='SW'		descriptive<br>(Attachment: SW-Q18.mp3, Display format: Audio file (play in embedded player on page))
149	[transport_cost_dh]<br><br>Show the field ONLY if:<br>[event-name]='3_month_visit_arm_1' AND ([transport_clinic]='3' or [transport_clinic]='4' or [transport_clinic]='5' or [transport_clinic]='6') AND [acasi_language]='DH'		descriptive<br>(Attachment: DH-Q18.mp3, Display format: Audio file (play in embedded player on page))
150	[transport_cost]<br><br>Show the field ONLY if:<br>[event-name]='3_month_visit_arm_1'	If you paid a fare for your transport, how much did it cost you (and anyone you brought) to come to the clinic?<br><br>Please enter the cost in Kenyan shillings. Enter 0 if you did not pay.	text (number), Required
151	[kilometers_eng]<br><br>Show the field ONLY if:<br>[event-name]='3_month_visit_arm_1' AND [acasi_language]='EN'	Section Header:	descriptive<br>(Attachment: Q19.mp3, Display format: Audio file (play in embedded player on page))
152	[kilometers_sw]<br><br>Show the field ONLY if:<br>[event-name]='3_month_visit_arm_1' AND [acasi_language]='SW'		descriptive<br>(Attachment: SW-Q19.mp3, Display format: Audio file (play in embedded player on page))
153	[kilometers_dh]<br><br>Show the field ONLY if:		descriptive<br>(Attachment: DH-Q19.mp3, Display format:

	[event-name]='3_month_visit_arm_1' AND [acasi_language]='DH'		Audio file (play in embedded player on page))
154	[kilometers]<br><br>Show the field ONLY if:<br>[event-name]='3_month_visit_arm_1'	If you came in a motorcycle or car for which you buy fuel, how many kilometers did you come (one way)?<br><br>Please enter the distance in kilometers. Enter 0 if you did not buy fuel.	text (number, Min: 0), Required
155	[time_spent_eng]<br><br>Show the field ONLY if:<br>[event-name]='3_month_visit_arm_1' AND [acasi_language]='EN'		descriptive<br>(Attachment: Q20.mp3, Display format: Audio file (play in embedded player on page))
156	[time_spent_sw]<br><br>Show the field ONLY if:<br>[event-name]='3_month_visit_arm_1' AND [acasi_language]='SW'		descriptive<br>(Attachment: SW-Q20.mp3, Display format: Audio file (play in embedded player on page))
157	[time_spent_dh]<br><br>Show the field ONLY if:<br>[event-name]='3_month_visit_arm_1' AND [acasi_language]='DH'		descriptive<br>(Attachment: DH-Q20.mp3, Display format: Audio file (play in embedded player on page))
158	[time_spent]<br><br>Show the field ONLY if:<br>[event-name]='3_month_visit_arm_1'	How many minutes did you spend in the clinic area waiting to be seen?<br><br>Please answer in minutes.	text (number), Required
159	[watch_child_eng]<br><br>Show the field ONLY if:<br>[event-name]='3_month_visit_arm_1' AND [acasi_language]='EN'		descriptive<br>(Attachment: Q21.mp3, Display format: Audio file (play in embedded player on page))
160	[watch_child_sw]<br><br>Show the field ONLY if:<br>[event-name]='3_month_visit_arm_1' AND [acasi_language]='SW'		descriptive<br>(Attachment: SW-Q21.mp3, Display format: Audio file (play in embedded player on page))
161	[watch_child_dh]<br><br>Show the field ONLY if:<br>[event-name]='3_month_visit_arm_1' AND [acasi_language]='DH'		descriptive<br>(Attachment: DH-Q21.mp3, Display format: Audio file (play in embedded player on page))
162	[watch_child]<br><br>Show the field ONLY if:<br>[event-name]='3_month_visit_arm_1'	Did you pay someone to watch your child (or other dependent) so you could come to the clinic today?	radio, Required<br>1 No<br>2 Yes<br>3 Refuse to answer
163	[watch_child_cost_eng]<br><br>Show the field ONLY if:<br>[event-name]='3_month_visit_arm_1' AND [watch_		descriptive<br>(Attachment: Q22.mp3, Display format: Audio file (play in embedded player on page))

	child]='2' AND [acasi_language]='EN'		
164	[watch_child_cost_sw]<br><br>Show the field ONLY if:<br>[event-name]='3_month_visit_arm_1' AND [watch_child]='2' AND [acasi_language]='SW'		descriptive<br>(Attachment: SW-Q22.mp3, Display format: Audio file (play in embedded player on page))
165	[watch_child_cost_dh]<br><br>Show the field ONLY if:<br>[event-name]='3_month_visit_arm_1' AND [watch_child]='2' AND [acasi_language]='DH'		descriptive<br>(Attachment: DH-Q22.mp3, Display format: Audio file (play in embedded player on page))
166	[watch_child_cost]<br><br>Show the field ONLY if:<br>[event-name]='3_month_visit_arm_1' AND [watch_child]='2'	How much did you pay them?<br><br>Please enter the cost in Kenyan shillings. Enter 0 if you did not pay.	text (number, Min: 0)
167	[other_costs_eng]<br><br>Show the field ONLY if:<br>[event-name]='3_month_visit_arm_1' AND [acasi_language]='EN'		descriptive<br>(Attachment: Q23.mp3, Display format: Audio file (play in embedded player on page))
168	[other_costs_sw]<br><br>Show the field ONLY if:<br>[event-name]='3_month_visit_arm_1' AND [acasi_language]='SW'		descriptive<br>(Attachment: SW-Q23.mp3, Display format: Audio file (play in embedded player on page))
169	[other_costs_dh]<br><br>Show the field ONLY if:<br>[event-name]='3_month_visit_arm_1' AND [acasi_language]='DH'		descriptive<br>(Attachment: DH-Q23.mp3, Display format: Audio file (play in embedded player on page))
170	[other_costs]<br><br>Show the field ONLY if:<br>[event-name]='3_month_visit_arm_1'	Were there any other costs associated with coming to the clinic today?	radio, Required<br>1 No<br>2 Yes<br>3 Refuse to answer
171	[cost_details_eng]<br><br>Show the field ONLY if:<br>[event-name]='3_month_visit_arm_1' and [other_costs]='2' AND [acasi_language]='EN'		descriptive<br>(Attachment: Q24.mp3, Display format: Audio file (play in embedded player on page))
172	[cost_details_sw]<br><br>Show the field ONLY if:<br>[event-name]='3_month_visit_arm_1' and [other_costs]='2' AND [acasi_language]='SW'		descriptive<br>(Attachment: SW-Q24.mp3, Display format: Audio file (play in embedded player on page))

173	[cost_details_dh]<br><br>Show the field ONLY if:<br>[event-name]='3_month_visit_arm_1' and [other_costs]='2' AND [acasi_language]='DH'		descriptive<br>(Attachment: DH-Q24.mp3, Display format: Audio file (play in embedded player on page))
174	[cost_details]<br><br>Show the field ONLY if:<br>[event-name]='3_month_visit_arm_1' and [other_costs]='2'	Please let us know the costs in Kenyan shillings and what this was for (e.g., meals for you and anyone you brought).	notes, Required
175	[sexualhx_prompt_eng]<br><br>Show the field ONLY if:<br>[event-name]='baseline_visit_arm_1' AND [acasi_language]='EN'	Section Header: *Sexual History (enrollment only) Now I will ask some questions about your sexual history. When we use the word sex: we mean inserting your penis into the anus or vagina of another person (going top) or receiving another person's penis in your anus (going bottom). When we ask about your male partners, this includes any man or boy. When we ask about your female partners, this includes any woman or girl.*	descriptive<br>(Attachment: INST-Q25.mp3, Display format: Audio file (play in embedded player on page))
176	[sexualhx_prompt_sw]<br><br>Show the field ONLY if:<br>[event-name]='baseline_visit_arm_1' AND [acasi_language]='SW'		descriptive<br>(Attachment: SW-INST-Q25.mp3, Display format: Audio file (play in embedded player on page))
177	[sexualhx_prompt_dh]<br><br>Show the field ONLY if:<br>[event-name]='baseline_visit_arm_1' AND [acasi_language]='DH'		descriptive<br>(Attachment: DH-INST-Q25.mp3, Display format: Audio file (play in embedded player on page))
178	[ever_bottom_eng]<br><br>Show the field ONLY if:<br>[event-name]='baseline_visit_arm_1' AND [acasi_language]='EN'		descriptive<br>(Attachment: Q25.mp3, Display format: Audio file (play in embedded player on page))
179	[ever_bottom_sw]<br><br>Show the field ONLY if:<br>[event-name]='baseline_visit_arm_1' AND [acasi_language]='SW'		descriptive<br>(Attachment: SW-Q25.mp3, Display format: Audio file (play in embedded player on page))
180	[ever_bottom_dh]<br><br>Show the field ONLY if:<br>[event-name]='baseline_visit_arm_1' AND [acasi_language]='DH'		descriptive<br>(Attachment: DH-Q25.mp3, Display format: Audio file (play in embedded player on page))
181	[ever_bottom]<br><br>Show the field ONLY if:<br>[event-name]='baseline_visit_arm_1'	Have you ever had receptive (bottom) anal sex with a male partner?	radio, Required<br>1 No<br>2 Yes<br>3 Refuse to answer
182	[ever_top_eng]<br><br>Show the field ONLY if:<br>[event-name]='baseline_visit_arm_1' AND [acasi_l		descriptive<br>(Attachment: Q26.mp3, Display format: Audio file (play in embedded player on page))

	anguage]='EN'		
183	[ever_top_sw]<br><br>Show the field ONLY if:<br>[event-name]='baseline_visit_arm_1' AND [acasi_language]='SW'		descriptive<br>(Attachment: SW-Q26.mp3, Display format: Audio file (play in embedded player on page))
184	[ever_top_dh]<br><br>Show the field ONLY if:<br>[event-name]='baseline_visit_arm_1' AND [acasi_language]='DH'		descriptive<br>(Attachment: DH-Q26.mp3, Display format: Audio file (play in embedded player on page))
185	[ever_top]<br><br>Show the field ONLY if:<br>[event-name]='baseline_visit_arm_1'	Have you ever had insertive (top) anal sex with a male partner?	radio, Required<br>1 No<br>2 Yes<br>3 Refuse to answer
186	[lifetime_males_eng]<br><br>Show the field ONLY if:<br>[event-name]='baseline_visit_arm_1' AND [acasi_language]='EN'		descriptive<br>(Attachment: Q27.mp3, Display format: Audio file (play in embedded player on page))
187	[lifetime_males_sw]<br><br>Show the field ONLY if:<br>[event-name]='baseline_visit_arm_1' AND [acasi_language]='SW'		descriptive<br>(Attachment: SW-Q27.mp3, Display format: Audio file (play in embedded player on page))
188	[lifetime_males_dh]<br><br>Show the field ONLY if:<br>[event-name]='baseline_visit_arm_1' AND [acasi_language]='DH'		descriptive<br>(Attachment: DH-Q27.mp3, Display format: Audio file (play in embedded player on page))
189	[lifetime_males]<br><br>Show the field ONLY if:<br>[event-name]='baseline_visit_arm_1'	Please estimate: how many different males have you had anal sex with in your lifetime?	text (number, Min: 1)
190	[ever_cisfemale_eng]<br><br>Show the field ONLY if:<br>[event-name]='baseline_visit_arm_1' AND [acasi_language]='EN'	Section Header:	descriptive<br>(Attachment: Q28.mp3, Display format: Audio file (play in embedded player on page))
191	[ever_cisfemale_sw]<br><br>Show the field ONLY if:<br>[event-name]='baseline_visit_arm_1' AND [acasi_language]='SW'		descriptive<br>(Attachment: SW-Q28.mp3, Display format: Audio file (play in embedded player on page))
192	[ever_cisfemale_dh]<br><br>Show the field ONLY if:<br>[event-name]='baseline_visit_arm_1' AND [acasi_language]='DH'		descriptive<br>(Attachment: DH-Q28.mp3, Display format: Audio file (play in embedded player on page))

193	[ever_cisfemale]<br><br>Show the field ONLY if:<br>[event-name]='baseline_visit_arm_1'	Have you ever had vaginal or anal sex with a cisgender female?<br><br>(By cisgender female, we mean someone who identifies as a female and was born female.)	radio, Required<br>1 No<br>2 Yes<br>3 Don't know<br>4 Refuse to answer
194	[ever_transfemale_eng]<br><br>Show the field ONLY if:<br>[event-name]='baseline_visit_arm_1' AND [acasi_language]='EN'		descriptive<br>(Attachment: Q29.mp3, Display format: Audio file (play in embedded player on page))
195	[ever_transfemale_sw]<br><br>Show the field ONLY if:<br>[event-name]='baseline_visit_arm_1' AND [acasi_language]='SW'		descriptive<br>(Attachment: SW-Q29.mp3, Display format: Audio file (play in embedded player on page))
196	[ever_transfemale_dh]<br><br>Show the field ONLY if:<br>[event-name]='baseline_visit_arm_1' AND [acasi_language]='DH'		descriptive<br>(Attachment: DH-Q29.mp3, Display format: Audio file (play in embedded player on page))
197	[ever_transfemale]<br><br>Show the field ONLY if:<br>[event-name]='baseline_visit_arm_1'	Have you ever had anal sex with a transgender female?<br><br>(By transgender female, we mean someone who identifies as a female and was born male.)	radio, Required<br>1 No<br>2 Yes<br>3 Don't know<br>4 Refuse to answer
198	[last_partner_eng]<br><br>Show the field ONLY if:<br>[acasi_language]='EN'	Section Header: *Sexual Behavior Assessment (enrollment and quarterly)*	descriptive<br>(Attachment: Q30.mp3, Display format: Audio file (play in embedded player on page))
199	[last_partner_sw]<br><br>Show the field ONLY if:<br>[acasi_language]='SW'		descriptive<br>(Attachment: SW-Q30.mp3, Display format: Audio file (play in embedded player on page))
200	[last_partner_dh]<br><br>Show the field ONLY if:<br>[acasi_language]='DH'		descriptive<br>(Attachment: DH-Q30.mp3, Display format: Audio file (play in embedded player on page))
201	[last_partner]	The last time you had sex, was your partner male, female, or transgender female?	radio, Required<br>1 Male<br>2 Female<br>3 Transgender female<br>4 Don't know<br>5 Refuse to answer
202	[lastsex_condom_eng]<br><br>Show the field ONLY if:<br>[acasi_language]='EN'		descriptive<br>(Attachment: Q31.mp3, Display format: Audio file (play in embedded player on page))

203	[lastsex_condom_sw]<br><br>Show the field ONLY if:<br>[acasi_language]='SW'		descriptive<br>(Attachment: SW-Q31.mp3, Display format: Audio file (play in embedded player on page))
204	[lastsex_condom_dh]<br><br>Show the field ONLY if:<br>[acasi_language]='DH'		descriptive<br>(Attachment: DH-Q31.mp3, Display format: Audio file (play in embedded player on page))
205	[lastsex_condom]	The last time you had sex, did you or your partner use a condom?	radio, Required<br>1 No<br>2 Yes<br>3 Refuse to answer
206	[last_sex_drugs_eng]<br><br>Show the field ONLY if:<br>[acasi_language]='EN'		descriptive<br>(Attachment: Q32.mp3, Display format: Audio file (play in embedded player on page))
207	[last_sex_drugs_sw]<br><br>Show the field ONLY if:<br>[acasi_language]='SW'		descriptive<br>(Attachment: SW-Q32.mp3, Display format: Audio file (play in embedded player on page))
208	[last_sex_drugs_dh]<br><br>Show the field ONLY if:<br>[acasi_language]='DH'		descriptive<br>(Attachment: DH-Q32.mp3, Display format: Audio file (play in embedded player on page))
209	[last_sex_drugs]	The last time you had sex, did you use alcohol or other drugs before or during sex?	radio, Required<br>1 Alcohol<br>2 Other drugs<br>3 Both alcohol and other drugs<br>4 No alcohol or other drugs<br>5 Refuse to answer
210	[trans_paid_eng]<br><br>Show the field ONLY if:<br>[acasi_language]='EN'		descriptive<br>(Attachment: Q33.mp3, Display format: Audio file (play in embedded player on page))
211	[trans_paid_sw]<br><br>Show the field ONLY if:<br>[acasi_language]='SW'		descriptive<br>(Attachment: SW-Q33.mp3, Display format: Audio file (play in embedded player on page))
212	[trans_paid_dh]<br><br>Show the field ONLY if:<br>[acasi_language]='DH'		descriptive<br>(Attachment: DH-Q33.mp3, Display format: Audio file (play in embedded player on page))
213	[trans_paid]	In the past 3 months, have you been paid for sex with cash, living expenses, or goods?	radio, Required<br>1 No<br>2 Yes<br>3 Refuse to answer
214	[trans_paying_eng]<br><br>Show the field ONLY if:<br>[acasi_language]='EN'	Section Header:	descriptive<br>(Attachment: Q34.mp3, Display format: Audio file (play in embedded player on page))

215	[trans_paying_sw]<br><br>Show the field ONLY if:<br>[acasi_language]='SW'		descriptive<br>(Attachment: SW-Q34.mp3, Display format: Audio file (play in embedded player on page))
216	[trans_paying_dh]<br><br>Show the field ONLY if:<br>[acasi_language]='DH'		descriptive<br>(Attachment: DH-Q34.mp3, Display format: Audio file (play in embedded player on page))
217	[trans_paying]	In the past 3 months, have you paid for sex with cash, living expenses, or goods?	radio, Required<br>1 No<br>2 Yes<br>3 Refuse to answer
218	[group_sex_eng]<br><br>Show the field ONLY if:<br>[acasi_language]='EN'		descriptive<br>(Attachment: Q35.mp3, Display format: Audio file (play in embedded player on page))
219	[group_sex_sw]<br><br>Show the field ONLY if:<br>[acasi_language]='SW'		descriptive<br>(Attachment: SW-Q35.mp3, Display format: Audio file (play in embedded player on page))
220	[group_sex_dh]<br><br>Show the field ONLY if:<br>[acasi_language]='DH'		descriptive<br>(Attachment: DH-Q35.mp3, Display format: Audio file (play in embedded player on page))
221	[group_sex]	In the past 3 months, have you participated in group sex?<br><br>(By group sex, we mean sex with more than one person at the same time.)	radio, Required<br>1 No<br>2 Yes<br>3 Refuse to answer
222	[femalesex_partners_eng]<br><br>Show the field ONLY if:<br>[acasi_language]='EN'		descriptive<br>(Attachment: Q36.mp3, Display format: Audio file (play in embedded player on page))
223	[femalesex_partners_sw]<br><br>Show the field ONLY if:<br>[acasi_language]='SW'		descriptive<br>(Attachment: SW-Q36.mp3, Display format: Audio file (play in embedded player on page))
224	[femalesex_partners_dh]<br><br>Show the field ONLY if:<br>[acasi_language]='DH'		descriptive<br>(Attachment: DH-Q36.mp3, Display format: Audio file (play in embedded player on page))
225	[femalesex_partners]	With how many women have you had anal sex in the last 3 months? If you are not sure of the exact number, take your best guess.	text (number, Min: 0), Required
226	[intro_en]<br><br>Show the field ONLY if:<br>[acasi_language]='EN'	Section Header: *There are many kinds of sex. For the next several questions, we are interested in sex when a man's penis goes inside a man's anus (anal sex).*	descriptive<br>(Attachment: INST-Q37.mp3, Display format: Audio file (play in embedded player on page))
227	[intro_sw]<br><br>Show the field ONLY if:<br>[acasi_language]='SW'		descriptive<br>(Attachment: SW-INST-Q37.mp3, Display

			format: Audio file (play in embedded player on page))
228	[intro_dh]<br><br>Show the field ONLY if:<br>[acasi_language]='DH'		descriptive<br>(Attachment: DH-INST-Q37.mp3, Display format: Audio file (play in embedded player on page))
229	[last_anal_male_eng]<br><br>Show the field ONLY if:<br>[acasi_language]='EN'		descriptive<br>(Attachment: Q37.mp3, Display format: Audio file (play in embedded player on page))
230	[last_anal_male_sw]<br><br>Show the field ONLY if:<br>[acasi_language]='SW'		descriptive<br>(Attachment: SW-Q37.mp3, Display format: Audio file (play in embedded player on page))
231	[last_anal_male_dh]<br><br>Show the field ONLY if:<br>[acasi_language]='DH'		descriptive<br>(Attachment: DH-Q37.mp3, Display format: Audio file (play in embedded player on page))
232	[last_anal_male]	When did you last have anal sex?	radio, Required<br>1 In the past 2 days<br>2 In the past week<br>3 In the past 2 weeks<br>4 In the past 3 months<br>5 Before the past 3 months<br>6 Never had anal sex<br>7 Refuse to answer
233	[analsex_partners_eng]<br><br>Show the field ONLY if:<br>([last_anal_male] = '1' or [last_anal_male] = '2' or [last_anal_male] = '3' or [last_anal_male] = '4') AN D [acasi_language]='EN'		descriptive<br>(Attachment: Q38.mp3, Display format: Audio file (play in embedded player on page))
234	[analsex_partners_sw]<br><br>Show the field ONLY if:<br>([last_anal_male] = '1' or [last_anal_male] = '2' or [last_anal_male] = '3' or [last_anal_male] = '4') AN D [acasi_language]='SW'		descriptive<br>(Attachment: SW-Q38.mp3, Display format: Audio file (play in embedded player on page))
235	[analsex_partners_dh]<br><br>Show the field ONLY if:<br>([last_anal_male] = '1' or [last_anal_male] = '2' or [last_anal_male] = '3' or [last_anal_male] = '4') AN D [acasi_language]='DH'		descriptive<br>(Attachment: DH-Q38.mp3, Display format: Audio file (play in embedded player on page))
236	[analsex_partners]<br><br>Show the field ONLY if:<br>[last_anal_male] = '1' or [last_anal_male] = '2' or	With how many men have you had anal sex in the last 3 months? If you are not sure of the exact number, take your best guess.	text (number, Min: 0), Required

	[last_anal_male] = '3' or [last_anal_male] = '4'		
237	[regular_partners_eng]<br><br>Show the field ONLY if: ([last_anal_male] = '1' or [last_anal_male] = '2' or [last_anal_male] = '3' or [last_anal_male] = '4') AN D [acasi_language]='EN'		descriptive<br>(Attachment: Q39.mp3, Display format: Audio file (play in embedded player on page))
238	[regular_partners_sw]<br><br>Show the field ONLY if: ([last_anal_male] = '1' or [last_anal_male] = '2' or [last_anal_male] = '3' or [last_anal_male] = '4') AN D [acasi_language]='SW'		descriptive<br>(Attachment: SW-Q39.mp3, Display format: Audio file (play in embedded player on page))
239	[regular_partners_dh]<br><br>Show the field ONLY if: ([last_anal_male] = '1' or [last_anal_male] = '2' or [last_anal_male] = '3' or [last_anal_male] = '4') AN D [acasi_language]='DH'		descriptive<br>(Attachment: DH-Q39.mp3, Display format: Audio file (play in embedded player on page))
240	[regular_partners]<br><br>Show the field ONLY if: [last_anal_male] = '1' or [last_anal_male] = '2' or [last_anal_male] = '3' or [last_anal_male] = '4'	Of the men you had anal sex with in the last 3 months, how many were regular (long-term) partners? If you are not sure of the exact number, take your best guess.	text (number, Min: 0, Max: [analsex_partners]), Required
241	[nonregular_partners]<br><br>Show the field ONLY if: [analsex_partners] >1	nonregular_partners	calc<br>Calculation: [analsex_partners] - [regular_partners]<br>Field Annotation: @HIDDEN
242	[casual_partners_eng]<br><br>Show the field ONLY if: ([last_anal_male] = '1' or [last_anal_male] = '2' or [last_anal_male] = '3' or [last_anal_male] = '4') AN D [acasi_language]='EN'	Section Header:	descriptive<br>(Attachment: Q40.mp3, Display format: Audio file (play in embedded player on page))
243	[casual_partners_sw]<br><br>Show the field ONLY if: ([last_anal_male] = '1' or [last_anal_male] = '2' or [last_anal_male] = '3' or [last_anal_male] = '4') AN D [acasi_language]='SW'		descriptive<br>(Attachment: SW-Q40.mp3, Display format: Audio file (play in embedded player on page))

244	[casual_partners_dh]<br><br>Show the field ONLY if:<br>([last_anal_male] = '1' or [last_anal_male] = '2' or [last_anal_male] = '3' or [last_anal_male] = '4') AND [acasi_language]='DH'		descriptive<br>(Attachment: DH-Q40.mp3, Display format: Audio file (play in embedded player on page))
245	[casual_partners]<br><br>Show the field ONLY if:<br>[last_anal_male] = '1' or [last_anal_male] = '2' or [last_anal_male] = '3' or [last_anal_male] = '4'	Of the men you had anal sex with in the last 3 months, how many were casual (short-term) partners? If you are not sure of the exact number, take your best guess.	text (number, Min: 0, Max: [nonregular_partners]), Required
246	[oneoff_partners_eng]<br><br>Show the field ONLY if:<br>([last_anal_male] = '1' or [last_anal_male] = '2' or [last_anal_male] = '3' or [last_anal_male] = '4') AND [acasi_language]='EN'		descriptive<br>(Attachment: Q41.mp3, Display format: Audio file (play in embedded player on page))
247	[oneoff_partners_sw]<br><br>Show the field ONLY if:<br>([last_anal_male] = '1' or [last_anal_male] = '2' or [last_anal_male] = '3' or [last_anal_male] = '4') AND [acasi_language]='SW'		descriptive<br>(Attachment: SW-Q41.mp3, Display format: Audio file (play in embedded player on page))
248	[oneoff_partners_dh]<br><br>Show the field ONLY if:<br>([last_anal_male] = '1' or [last_anal_male] = '2' or [last_anal_male] = '3' or [last_anal_male] = '4') AND [acasi_language]='DH'		descriptive<br>(Attachment: DH-Q41.mp3, Display format: Audio file (play in embedded player on page))
249	[oneoff_partners]<br><br>Show the field ONLY if:<br>[last_anal_male] = '1' or [last_anal_male] = '2' or [last_anal_male] = '3' or [last_anal_male] = '4'	Of the men you had anal sex with in the last 3 months, how many were one-time partners? If you are not sure of the exact number, take your best guess.	text (number), Required
250	[client_partners_eng]<br><br>Show the field ONLY if:<br>([last_anal_male] = '1' or [last_anal_male] = '2' or [last_anal_male] = '3' or [last_anal_male] = '4') AND [acasi_language]='EN'		descriptive<br>(Attachment: Q42.mp3, Display format: Audio file (play in embedded player on page))
251	[client_partners_sw]<br><br>Show the field ONLY if:<br>([last_anal_male] = '1' or [last_anal_male] = '2' or [last_anal_male] = '3' or		descriptive<br>(Attachment: SW-Q42.mp3, Display format: Audio file (play in embedded player on page))

	[last_anal_male] = '4') AND [acasi_language]='SW'		
252	[client_partners_dh]<br><br>Show the field ONLY if:<br>([last_anal_male] = '1' or [last_anal_male] = '2' or [last_anal_male] = '3' or [last_anal_male] = '4') AND [acasi_language]='DH'		descriptive<br>(Attachment: DH-Q42.mp3, Display format: Audio file (play in embedded player on page))
253	[client_partners]<br><br>Show the field ONLY if:<br>[last_anal_male] = '1' or [last_anal_male] = '2' or [last_anal_male] = '3' or [last_anal_male] = '4'	Of the men you had anal sex with in the last 3 months, how many paid you money, gifts, or living expenses for sex? If you are not sure of the exact number, take your best guess.	text (number), Required
254	[paid_sex_partners_eng]<br><br>Show the field ONLY if:<br>([last_anal_male] = '1' or [last_anal_male] = '2' or [last_anal_male] = '3' or [last_anal_male] = '4') AND [acasi_language]='EN'	Section Header:	descriptive<br>(Attachment: Q43.mp3, Display format: Audio file (play in embedded player on page))
255	[paid_sex_partners_sw]<br><br>Show the field ONLY if:<br>([last_anal_male] = '1' or [last_anal_male] = '2' or [last_anal_male] = '3' or [last_anal_male] = '4') AND [acasi_language]='SW'		descriptive<br>(Attachment: SW-Q43.mp3, Display format: Audio file (play in embedded player on page))
256	[paid_sex_partners_dh]<br><br>Show the field ONLY if:<br>([last_anal_male] = '1' or [last_anal_male] = '2' or [last_anal_male] = '3' or [last_anal_male] = '4') AND [acasi_language]='DH'		descriptive<br>(Attachment: DH-Q43.mp3, Display format: Audio file (play in embedded player on page))
257	[paid_sex_partners]<br><br>Show the field ONLY if:<br>[last_anal_male] = '1' or [last_anal_male] = '2' or [last_anal_male] = '3' or [last_anal_male] = '4'	Of the men you had anal sex with in the last 3 months, how many did you pay with money, gifts, or living expenses for sex? If you are not sure of the exact number, take your best guess.	text (number), Required
258	[partner_3mo_sti_eng]<br><br>Show the field ONLY if:<br>([last_anal_male] = '1' or [last_anal_male] = '2' or [last_anal_male] = '3' or [last_anal_male] = '4') AND [acasi_language]='EN'		descriptive<br>(Attachment: Q44.mp3, Display format: Audio file (play in embedded player on page))

259	[partner_3mo_sti_sw]<br><br>Show the field ONLY if: ([last_anal_male] = '1' or [last_anal_male] = '2' or [last_anal_male] = '3' or [last_anal_male] = '4') AND [acasi_language]='SW'		descriptive<br>(Attachment: SW-Q44.mp3, Display format: Audio file (play in embedded player on page))
260	[partner_3mo_sti_dh]<br><br>Show the field ONLY if: ([last_anal_male] = '1' or [last_anal_male] = '2' or [last_anal_male] = '3' or [last_anal_male] = '4') AND [acasi_language]='DH'		descriptive<br>(Attachment: DH-Q44.mp3, Display format: Audio file (play in embedded player on page))
261	[partner_3mo_sti]<br><br>Show the field ONLY if: [last_anal_male] = '1' or [last_anal_male] = '2' or [last_anal_male] = '3' or [last_anal_male] = '4'	Of the men you had anal sex with in the last 3 months, did any of them tell you they had a sexually transmitted infection?	radio, Required<br>1 No<br>2 Yes<br>3 Don't remember<br>4 Refuse to answer
262	[sex_network_prompt_eng]<br><br>Show the field ONLY if: ([last_anal_male] = '1' or [last_anal_male] = '2' or [last_anal_male] = '3' or [last_anal_male] = '4') AND ([event-name]='baseline_visit_arm_1' or [event-name]='6_month_visit_arm_1' or [event-name]='12_month_visit_arm_1' or [event-name]='18_month_visit_arm_1') AND [acasi_language]='EN'	Section Header: *Sexual Network Characteristics (enrollment, every 6 months) We are now going to ask about the male partner you had anal sex with most recently in the past 3 months.*	descriptive<br>(Attachment: INST-Q45.mp3, Display format: Audio file (play in embedded player on page))
263	[sex_network_prompt_sw]<br><br>Show the field ONLY if: ([last_anal_male] = '1' or [last_anal_male] = '2' or [last_anal_male] = '3' or [last_anal_male] = '4') AND ([event-name]='baseline_visit_arm_1' or [event-name]='6_month_visit_arm_1' or [event-name]='12_month_visit_arm_1' or [event-name]='18_month_visit_arm_1') AND [acasi_language]='SW'		descriptive<br>(Attachment: SW-INST-Q45.mp3, Display format: Audio file (play in embedded player on page))
264	[sex_network_prompt_dh]<br><br>Show the field ONLY if: ([last_anal_male] = '1' or [last_anal_male] = '2' or		descriptive<br>(Attachment: DH-INST-Q45.mp3, Display format: Audio file (play in embedded player on page))

	[last_anal_male] = '3' or [last_anal_male] = '4') AND ([event-name]='baseline_visit_arm_1' or [event-name]='6_month_visit_arm_1' or [event-name]='12_month_visit_arm_1' or [event-name]='18_month_visit_arm_1') AND [acasi_language]='DH'		
265	[alter_age1_en]<br><br>Show the field ONLY if:<br>([last_anal_male] = '1' or [last_anal_male] = '2' or [last_anal_male] = '3' or [last_anal_male] = '4') AND ([event-name]='baseline_visit_arm_1' or [event-name]='6_month_visit_arm_1' or [event-name]='12_month_visit_arm_1' or [event-name]='18_month_visit_arm_1') AND [acasi_language]='EN'		descriptive<br>(Attachment: Q45.mp3, Display format: Audio file (play in embedded player on page))
266	[alter_age1_sw]<br><br>Show the field ONLY if:<br>([last_anal_male] = '1' or [last_anal_male] = '2' or [last_anal_male] = '3' or [last_anal_male] = '4') AND ([event-name]='baseline_visit_arm_1' or [event-name]='6_month_visit_arm_1' or [event-name]='12_month_visit_arm_1' or [event-name]='18_month_visit_arm_1') AND [acasi_language]='SW'		descriptive<br>(Attachment: SW-Q45.mp3, Display format: Audio file (play in embedded player on page))
267	[alter_age1_dh]<br><br>Show the field ONLY if:<br>([last_anal_male] = '1' or [last_anal_male] = '2' or [last_anal_male] = '3' or [last_anal_male] = '4') AND ([event-name]='baseline_visit_arm_1' or [event-name]='6_month_visit_arm_1' or [event-name]='12_month_visit_arm_1' or [event-name]='18_month_visit_arm_1') AND [acasi_language]='DH'		descriptive<br>(Attachment: DH-Q45.mp3, Display format: Audio file (play in embedded player on page))
268	[alter_age1]<br><br>Show the field ONLY if:<br>([last_anal_male] = '1' or [last_anal_male] = '2' or [last_anal_male] = '3' or	What is the age of this person?	radio, Required<br>1 About the same age as me<br>2 More than 5 years older than me<br>3 More than 5 years younger than me

	[last_anal_male] = '4') AND ([event-name]='baseline_visit_arm_1' or [event-name]='6_month_visit_arm_1' or [event-name]='12_month_visit_arm_1' or [event-name]='18_month_visit_arm_1')		4 Refuse to answer
269	[alter_condom1_eng]<br><br>Show the field ONLY if:<br>([last_anal_male] = '1' or [last_anal_male] = '2' or [last_anal_male] = '3' or [last_anal_male] = '4') AND ([event-name]='baseline_visit_arm_1' or [event-name]='6_month_visit_arm_1' or [event-name]='12_month_visit_arm_1' or [event-name]='18_month_visit_arm_1') AND [acasi_language]='EN'		descriptive<br>(Attachment: Q46.mp3, Display format: Audio file (play in embedded player on page))
270	[alter_condom1_sw]<br><br>Show the field ONLY if:<br>([last_anal_male] = '1' or [last_anal_male] = '2' or [last_anal_male] = '3' or [last_anal_male] = '4') AND ([event-name]='baseline_visit_arm_1' or [event-name]='6_month_visit_arm_1' or [event-name]='12_month_visit_arm_1' or [event-name]='18_month_visit_arm_1') AND [acasi_language]='SW'		descriptive<br>(Attachment: SW-Q46.mp3, Display format: Audio file (play in embedded player on page))
271	[alter_condom1_dh]<br><br>Show the field ONLY if:<br>([last_anal_male] = '1' or [last_anal_male] = '2' or [last_anal_male] = '3' or [last_anal_male] = '4') AND ([event-name]='baseline_visit_arm_1' or [event-name]='6_month_visit_arm_1' or [event-name]='12_month_visit_arm_1' or [event-name]='18_month_visit_arm_1') AND [acasi_language]='DH'		descriptive<br>(Attachment: DH-Q46.mp3, Display format: Audio file (play in embedded player on page))
272	[alter_condom1]<br><br>Show the field ONLY if:<br>([last_anal_male] = '1' or [last_anal_male] = '2' or [last_anal_male] = '3' or [last_anal_male] = '4') AND ([event-name]='baselin	When you last had anal sex with this person, did you or your partner use a condom?	radio, Required<br>1 No<br>2 Yes<br>3 Don't remember<br>4 Refuse to answer

	e_visit_arm_1' or [event-name]='6_month_visit_arm_1' or [event-name]='12_month_visit_arm_1' or [event-name]='18_month_visit_arm_1')		
273	[alter_doxypep1_eng]<br><br>Show the field ONLY if:<br>([last_anal_male] = '1' or [last_anal_male] = '2' or [last_anal_male] = '3' or [last_anal_male] = '4') and [randomization_arm_1][randomization_group]='1' and ([event-name]='6_month_visit_arm_1' or [event-name]='12_month_visit_arm_1' or [event-name]='18_month_visit_arm_1') AND [acasi_language]='EN'		descriptive<br>(Attachment: Q47.mp3, Display format: Audio file (play in embedded player on page))
274	[alter_doxypep1_sw]<br><br>Show the field ONLY if:<br>([last_anal_male] = '1' or [last_anal_male] = '2' or [last_anal_male] = '3' or [last_anal_male] = '4') and [randomization_arm_1][randomization_group]='1' and ([event-name]='6_month_visit_arm_1' or [event-name]='12_month_visit_arm_1' or [event-name]='18_month_visit_arm_1') AND [acasi_language]='SW'		descriptive<br>(Attachment: SW-Q47.mp3, Display format: Audio file (play in embedded player on page))
275	[alter_doxypep1_dh]<br><br>Show the field ONLY if:<br>([last_anal_male] = '1' or [last_anal_male] = '2' or [last_anal_male] = '3' or [last_anal_male] = '4') and [randomization_arm_1][randomization_group]='1' and ([event-name]='6_month_visit_arm_1' or [event-name]='12_month_visit_arm_1' or [event-name]='18_month_visit_arm_1') AND [acasi_language]='DH'		descriptive<br>(Attachment: DH-Q47.mp3, Display format: Audio file (play in embedded player on page))

276	[alter_doxypep1]<br><br>Show the field ONLY if:<br>([last_anal_male] = '1' or [last_anal_male] = '2' or [last_anal_male] = '3' or [last_anal_male] = '4') and [randomization_arm_1][randomization_group] ='1' and ([event-name] ='6_month_visit_arm_1' or [event-name]='12_month_visit_arm_1' or [event-name]='18_month_visit_arm_1')	After you last had anal sex with this person, did you take doxyPEP?	radio, Required<br>1 No<br>2 Yes<br>3 Don't remember<br>4 Refuse to answer
277	[alter_relationship1_eng]<br><br>Show the field ONLY if:<br>([last_anal_male] = '1' or [last_anal_male] = '2' or [last_anal_male] = '3' or [last_anal_male] = '4') AND ([event-name]='baseline_visit_arm_1' or [event-name]='6_month_visit_arm_1' or [event-name]='12_month_visit_arm_1' or [event-name]='18_month_visit_arm_1') AND [acasi_language]='EN'	Section Header:	descriptive<br>(Attachment: Q48.mp3, Display format: Audio file (play in embedded player on page))
278	[alter_relationship1_sw]<br><br>Show the field ONLY if:<br>([last_anal_male] = '1' or [last_anal_male] = '2' or [last_anal_male] = '3' or [last_anal_male] = '4') AND ([event-name]='baseline_visit_arm_1' or [event-name]='6_month_visit_arm_1' or [event-name]='12_month_visit_arm_1' or [event-name]='18_month_visit_arm_1') AND [acasi_language]='SW'		descriptive<br>(Attachment: SW-Q48.mp3, Display format: Audio file (play in embedded player on page))
279	[alter_relationship1_dh]<br><br>Show the field ONLY if:<br>([last_anal_male] = '1' or [last_anal_male] = '2' or [last_anal_male] = '3' or [last_anal_male] = '4') AND ([event-name]='baseline_visit_arm_1' or [event-name]='6_month_visit_arm_1' or [event-name]='12_month_visit_arm_1' or [event-name]='18_mont		descriptive<br>(Attachment: Q48 L.mp3, Display format: Audio file (play in embedded player on page))

	h_visit_arm_1') AND [acasi_language]='DH'		
280	[alter_relationship1]<br><br>Show the field ONLY if:<br>([last_anal_male] = '1' or [last_anal_male] = '2' or [last_anal_male] = '3' or [last_anal_male] = '4') AND ([event-name]='baseline_visit_arm_1' or [event-name]='6_month_visit_arm_1' or [event-name]='12_month_visit_arm_1' or [event-name]='18_month_visit_arm_1')	How would you describe your relationship with this person?	radio, Required<br>1 Regular (long-term) partner<br>2 Casual (short-term) partner<br>3 One-time encounter<br>4 Other (Specify)<br>5 Refuse to answer
281	[other_alter_relationship1]<br><br>Show the field ONLY if:<br>[alter_relationship1] = '4'	Other relationship (Specify)	text, Required
282	[alter_role1_eng]<br><br>Show the field ONLY if:<br>([last_anal_male] = '1' or [last_anal_male] = '2' or [last_anal_male] = '3' or [last_anal_male] = '4') AND ([event-name]='baseline_visit_arm_1' or [event-name]='6_month_visit_arm_1' or [event-name]='12_month_visit_arm_1' or [event-name]='18_month_visit_arm_1') AND [acasi_language]='EN'		descriptive<br>(Attachment: Q49.mp3, Display format: Audio file (play in embedded player on page))
283	[alter_role1_sw]<br><br>Show the field ONLY if:<br>([last_anal_male] = '1' or [last_anal_male] = '2' or [last_anal_male] = '3' or [last_anal_male] = '4') AND ([event-name]='baseline_visit_arm_1' or [event-name]='6_month_visit_arm_1' or [event-name]='12_month_visit_arm_1' or [event-name]='18_month_visit_arm_1') AND [acasi_language]='SW'		descriptive<br>(Attachment: SW-Q49.mp3, Display format: Audio file (play in embedded player on page))
284	[alter_role1_dh]<br><br>Show the field ONLY if:<br>([last_anal_male] = '1' or [last_anal_male] = '2' or [last_anal_male] = '3' or [last_anal_male] = '4') AND ([event-name]='baseline_visit_arm_1' or [event-name]='6_month_visit_ar		descriptive<br>(Attachment: DH-Q49.mp3, Display format: Audio file (play in embedded player on page))

	m_1' or [event-name]='12_month_visit_arm_1' or [event-name]='18_month_visit_arm_1') AND [acasi_language]='DH'		
285	[alter_role1]<br><br>Show the field ONLY if: ([last_anal_male] = '1' or [last_anal_male] = '2' or [last_anal_male] = '3' or [last_anal_male] = '4') AND ([event-name]='baseline_visit_arm_1' or [event-name]='6_month_visit_arm_1' or [event-name]='12_month_visit_arm_1' or [event-name]='18_month_visit_arm_1')	When you had anal sex with this partner, what role did you take?	radio, Required<br>1 Insertive (top) only<br>2 Mostly insertive (top)<br>3 About 50/50 insertive (top) and receptive (bottom)<br>4 Mostly receptive (bottom)<br>5 Receptive (bottom) only<br>6 Refuse to answer
286	[date_lastanalsex1_eng]<br><br>Show the field ONLY if: ([last_anal_male] = '1' or [last_anal_male] = '2' or [last_anal_male] = '3' or [last_anal_male] = '4') and ([alter_relationship1] = '1' or [alter_relationship1] = '2' or [alter_relationship1] = '4') AND ([event-name]='baseline_visit_arm_1' or [event-name]='6_month_visit_arm_1' or [event-name]='12_month_visit_arm_1' or [event-name]='18_month_visit_arm_1') AND [acasi_language]='EN'		descriptive<br>(Attachment: Q50.mp3, Display format: Audio file (play in embedded player on page))
287	[date_lastanalsex1_sw]<br><br>Show the field ONLY if: ([last_anal_male] = '1' or [last_anal_male] = '2' or [last_anal_male] = '3' or [last_anal_male] = '4') and ([alter_relationship1] = '1' or [alter_relationship1] = '2' or [alter_relationship1] = '4') AND ([event-name]='baseline_visit_arm_1' or [event-name]='6_month_visit_arm_1' or [event-name]='12_month_visit_arm_1' or [event-name]='18_month_visit_arm_1') AND [acasi_language]='SW'		descriptive<br>(Attachment: SW-Q50.mp3, Display format: Audio file (play in embedded player on page))
288	[date_lastanalsex1_dh]<br><br>Show the field ONLY if:		descriptive<br>(Attachment: DH-Q50.mp3, Display format:

	([last_anal_male] = '1' or [last_anal_male] = '2' or [last_anal_male] = '3' or [last_anal_male] = '4') and ([alter_relationship1] = '1' or [alter_relationship1] = '2' or [alter_relationship1] = '4') AND ([event-name]='baseline_visit_arm_1' or [event-name]='6_month_visit_arm_1' or [event-name]='12_month_visit_arm_1' or [event-name]='18_month_visit_arm_1') AND [acasi_language]='DH'		Audio file (play in embedded player on page))
289	[date_lastanalsex1]<br>Show the field ONLY if: ([last_anal_male] = '1' or [last_anal_male] = '2' or [last_anal_male] = '3' or [last_anal_male] = '4') and ([alter_relationship1] = '1' or [alter_relationship1] = '2' or [alter_relationship1] = '4') AND ([event-name]='baseline_visit_arm_1' or [event-name]='6_month_visit_arm_1' or [event-name]='12_month_visit_arm_1' or [event-name]='18_month_visit_arm_1')	When did you last have anal sex with this partner? If you do not know the exact date, please make your best guess.	text (date_dmy), Required
290	[date_firstanalsex1_eng]<br>Show the field ONLY if: ([last_anal_male] = '1' or [last_anal_male] = '2' or [last_anal_male] = '3' or [last_anal_male] = '4') and ([alter_relationship1] = '1' or [alter_relationship1] = '2' or [alter_relationship1] = '4') AND ([event-name]='baseline_visit_arm_1' or [event-name]='6_month_visit_arm_1' or [event-name]='12_month_visit_arm_1' or [event-name]='18_month_visit_arm_1') AND [acasi_language]='EN'	Section Header:	descriptive<br>(Attachment: Q51.mp3, Display format: Audio file (play in embedded player on page))
291	[date_firstanalsex1_sw]<br>Show the field ONLY if: ([last_anal_male] = '1' or [last_anal_male] = '2' or [last_anal_male] = '3' or		descriptive<br>(Attachment: SW-Q51.mp3, Display format: Audio file (play in embedded player on page))

	[last_anal_male] = '4') and ([alter_relationship1] = '1' or [alter_relationship1] = '2' or [alter_relationship1] = '4') AND ([event-name]='baseline_visit_arm_1' or [event-name]='6_month_visit_arm_1' or [event-name]='12_month_visit_arm_1' or [event-name]='18_month_visit_arm_1') AND [acasi_language]='SW'		
292	[date_firstanalsex1_dh]<br><br>Show the field ONLY if: ([last_anal_male] = '1' or [last_anal_male] = '2' or [last_anal_male] = '3' or [last_anal_male] = '4') and ([alter_relationship1] = '1' or [alter_relationship1] = '2' or [alter_relationship1] = '4') AND ([event-name]='baseline_visit_arm_1' or [event-name]='6_month_visit_arm_1' or [event-name]='12_month_visit_arm_1' or [event-name]='18_month_visit_arm_1') AND [acasi_language]='DH'		descriptive<br>(Attachment: DH-Q51.mp3, Display format: Audio file (play in embedded player on page))
293	[date_firstanalsex1]<br><br>Show the field ONLY if: ([last_anal_male] = '1' or [last_anal_male] = '2' or [last_anal_male] = '3' or [last_anal_male] = '4') and ([alter_relationship1] = '1' or [alter_relationship1] = '2' or [alter_relationship1] = '4') AND ([event-name]='baseline_visit_arm_1' or [event-name]='6_month_visit_arm_1' or [event-name]='12_month_visit_arm_1' or [event-name]='18_month_visit_arm_1')	When did you have anal sex with this partner for the very first time? If you do not know the exact date, please make your best guess.	text (date_dmy), Required
294	[partner1_ongoing_eng]<br><br>Show the field ONLY if: ([last_anal_male] = '1' or [last_anal_male] = '2' or [last_anal_male] = '3' or [last_anal_male] = '4') and ([alter_relationship1] = '1' or [alter_relationship1] = '2' or [alter_relations		descriptive<br>(Attachment: Q52.mp3, Display format: Audio file (play in embedded player on page))

	hip1] = '4') AND ([event-name]='baseline_visit_arm_1' or [event-name]='6_month_visit_arm_1' or [event-name]='12_month_visit_arm_1' or [event-name]='18_month_visit_arm_1') AND [acasi_language]='EN'		
295	[partner1_ongoing_sw]<br><br>Show the field ONLY if: ([last_anal_male] = '1' or [last_anal_male] = '2' or [last_anal_male] = '3' or [last_anal_male] = '4') and ([alter_relationship1] = '1' or [alter_relationship1] = '2' or [alter_relationship1] = '4') AND ([event-name]='baseline_visit_arm_1' or [event-name]='6_month_visit_arm_1' or [event-name]='12_month_visit_arm_1' or [event-name]='18_month_visit_arm_1') AND [acasi_language]='SW'		descriptive<br>(Attachment: SW-Q52.mp3, Display format: Audio file (play in embedded player on page))
296	[partner1_ongoing_dh]<br><br>Show the field ONLY if: ([last_anal_male] = '1' or [last_anal_male] = '2' or [last_anal_male] = '3' or [last_anal_male] = '4') and ([alter_relationship1] = '1' or [alter_relationship1] = '2' or [alter_relationship1] = '4') AND ([event-name]='baseline_visit_arm_1' or [event-name]='6_month_visit_arm_1' or [event-name]='12_month_visit_arm_1' or [event-name]='18_month_visit_arm_1') AND [acasi_language]='DH'		descriptive<br>(Attachment: DH-Q52.mp3, Display format: Audio file (play in embedded player on page))
297	[partner1_ongoing]<br><br>Show the field ONLY if: ([last_anal_male] = '1' or [last_anal_male] = '2' or [last_anal_male] = '3' or [last_anal_male] = '4') and ([alter_relationship1] = '1' or [alter_relationship1] = '2' or [alter_relationship1] = '4') AND ([event-name]='baseline_visit_arm_1' or [event-name]='6_month_visit_arm_1' or	Do you expect to have anal sex with this partner again?	radio<br>1 No<br>2 Yes<br>3 Don't know

	[event-name]='12_month_visit_arm_1' or [event-name]='18_month_visit_arm_1')		
298	[freq_analsex1_eng]<br><br>Show the field ONLY if: ([last_anal_male] = '1' or [last_anal_male] = '2' or [last_anal_male] = '3' or [last_anal_male] = '4') and ([alter_relationship1] = '1' or [alter_relationship1] = '2' or [alter_relationship1] = '4') AND [partner1_ongoing]='2' AND ([event-name]='baseline_visit_arm_1' or [event-name]='6_month_visit_arm_1' or [event-name]='12_month_visit_arm_1' or [event-name]='18_month_visit_arm_1') AND [acasi_language]='EN'		descriptive<br>(Attachment: Q53.mp3, Display format: Audio file (play in embedded player on page))
299	[freq_analsex1_sw]<br><br>Show the field ONLY if: ([last_anal_male] = '1' or [last_anal_male] = '2' or [last_anal_male] = '3' or [last_anal_male] = '4') and ([alter_relationship1] = '1' or [alter_relationship1] = '2' or [alter_relationship1] = '4') AND [partner1_ongoing]='2' AND ([event-name]='baseline_visit_arm_1' or [event-name]='6_month_visit_arm_1' or [event-name]='12_month_visit_arm_1' or [event-name]='18_month_visit_arm_1') AND [acasi_language]='SW'		descriptive<br>(Attachment: Q53 K.mp3, Display format: Audio file (play in embedded player on page))
300	[freq_analsex1_dh]<br><br>Show the field ONLY if: ([last_anal_male] = '1' or [last_anal_male] = '2' or [last_anal_male] = '3' or [last_anal_male] = '4') and ([alter_relationship1] = '1' or [alter_relationship1] = '2' or [alter_relationship1] = '4') AND [partner1_ongoing]='2' AND ([event-name]='baseline_visit_arm_1' or [event-name]='6_month_visit_arm_1' or [event-name]='12_month_visit_arm_1' or [even		descriptive<br>(Attachment: DH-Q53.mp3, Display format: Audio file (play in embedded player on page))

	t-name]='18_month_visit_arm_1') AND [acasi_language]='DH'		
301	[freq_analsex1]<br><br>Show the field ONLY if:<br>([last_anal_male] = '1' or [last_anal_male] = '2' or [last_anal_male] = '3' or [last_anal_male] = '4') and ([alter_relationship1] = '1' or [alter_relationship1] = '2' or [alter_relationship1] = '4') AND [partner1_ongoing]='2' AND ([event-name]='baseline_visit_arm_1' or [event-name]='6_month_visit_arm_1' or [event-name]='12_month_visit_arm_1' or [event-name]='18_month_visit_arm_1')	How often have you had anal sex with this partner in an average week?	radio, Required<br>1 Daily<br>2 Almost every day<br>3 Once a week<br>4 Less than once a week<br>5 Refuse to answer
302	[condom_analsex1_eng]<br><br>Show the field ONLY if:<br>([last_anal_male] = '1' or [last_anal_male] = '2' or [last_anal_male] = '3' or [last_anal_male] = '4') and ([alter_relationship1] = '1' or [alter_relationship1] = '2' or [alter_relationship1] = '4') AND ([event-name]='baseline_visit_arm_1' or [event-name]='6_month_visit_arm_1' or [event-name]='12_month_visit_arm_1' or [event-name]='18_month_visit_arm_1') AND [acasi_language]='EN'	Section Header:	descriptive<br>(Attachment: Q54.mp3, Display format: Audio file (play in embedded player on page))
303	[condom_analsex1_sw]<br><br>Show the field ONLY if:<br>([last_anal_male] = '1' or [last_anal_male] = '2' or [last_anal_male] = '3' or [last_anal_male] = '4') and ([alter_relationship1] = '1' or [alter_relationship1] = '2' or [alter_relationship1] = '4') AND ([event-name]='baseline_visit_arm_1' or [event-name]='6_month_visit_arm_1' or [event-name]='12_month_visit_arm_1' or [event-name]='18_month_visit_arm_1') AND [acasi_language]='SW'		descriptive<br>(Attachment: SW-Q54.mp3, Display format: Audio file (play in embedded player on page))

304	[condom_analsex1_dh]<br><br>Show the field ONLY if:<br>([last_anal_male] = '1' or [last_anal_male] = '2' or [last_anal_male] = '3' or [last_anal_male] = '4') and ([alter_relationship1] = '1' or [alter_relationship1] = '2' or [alter_relationship1] = '4') AND ([event-name]='baseline_visit_arm_1' or [event-name]='6_month_visit_arm_1' or [event-name]='12_month_visit_arm_1' or [event-name]='18_month_visit_arm_1') AND [acasi_language]='DH'		descriptive<br>(Attachment: DH-Q54.mp3, Display format: Audio file (play in embedded player on page))
305	[condom_analsex1]<br><br>Show the field ONLY if:<br>([last_anal_male] = '1' or [last_anal_male] = '2' or [last_anal_male] = '3' or [last_anal_male] = '4') and ([alter_relationship1] = '1' or [alter_relationship1] = '2' or [alter_relationship1] = '4') AND ([event-name]='baseline_visit_arm_1' or [event-name]='6_month_visit_arm_1' or [event-name]='12_month_visit_arm_1' or [event-name]='18_month_visit_arm_1')	When you have had anal sex with this partner, how often did you or your partner use a condom?	radio, Required<br>1 Always<br>2 Most of the time<br>3 About half the time<br>4 Rarely<br>5 Never<br>6 Refuse to answer
306	[hiv_analsex1_eng]<br><br>Show the field ONLY if:<br>([last_anal_male] = '1' or [last_anal_male] = '2' or [last_anal_male] = '3' or [last_anal_male] = '4') and ([alter_relationship1] = '1' or [alter_relationship1] = '2' or [alter_relationship1] = '4') AND ([event-name]='baseline_visit_arm_1' or [event-name]='6_month_visit_arm_1' or [event-name]='12_month_visit_arm_1' or [event-name]='18_month_visit_arm_1') AND [acasi_language]='EN'		descriptive<br>(Attachment: Q55.mp3, Display format: Audio file (play in embedded player on page))
307	[hiv_analsex1_sw]<br><br>Show the field ONLY if:<br>([last_anal_male] = '1' or [last_anal_male] = '2' or		descriptive<br>(Attachment: SW-Q55.mp3, Display format: Audio file (play in embedded player on page))

	[last_anal_male] = '3' or [last_anal_male] = '4') and ([alter_relationship1] = '1' or [alter_relationship1] = '2' or [alter_relationship1] = '4') AND ([event-name]='baseline_visit_arm_1' or [event-name]='6_month_visit_arm_1' or [event-name]='12_month_visit_arm_1' or [event-name]='18_month_visit_arm_1') AND [acasi_language]='SW'		
308	[hiv_analsex1_dh]<br>Show the field ONLY if:<br>([last_anal_male] = '1' or [last_anal_male] = '2' or [last_anal_male] = '3' or [last_anal_male] = '4') and ([alter_relationship1] = '1' or [alter_relationship1] = '2' or [alter_relationship1] = '4') AND ([event-name]='baseline_visit_arm_1' or [event-name]='6_month_visit_arm_1' or [event-name]='12_month_visit_arm_1' or [event-name]='18_month_visit_arm_1') AND [acasi_language]='DH'		descriptive<br>(Attachment: DH-Q55.mp3, Display format: Audio file (play in embedded player on page))
309	[hiv_analsex1]<br>Show the field ONLY if:<br>([last_anal_male] = '1' or [last_anal_male] = '2' or [last_anal_male] = '3' or [last_anal_male] = '4') and ([alter_relationship1] = '1' or [alter_relationship1] = '2' or [alter_relationship1] = '4') AND ([event-name]='baseline_visit_arm_1' or [event-name]='6_month_visit_arm_1' or [event-name]='12_month_visit_arm_1' or [event-name]='18_month_visit_arm_1')	What do you think this partner's HIV status is?	radio, Required<br>1 HIV positive<br>2 Probably HIV positive<br>3 I have no idea<br>4 Probably HIV negative<br>5 HIV negative<br>6 Refuse to answer
310	[hiv_reason_analsex1_eng]<br>Show the field ONLY if:<br>([last_anal_male] = '1' or [last_anal_male] = '2' or [last_anal_male] = '3' or [last_anal_male] = '4') and ([alter_relationship1] = '1' or [alter_relationship		descriptive<br>(Attachment: Q56.mp3, Display format: Audio file (play in embedded player on page))

	1] = '2' or [alter_relationship1] = '4') and ([hiv_analsex1] = '1' or [hiv_analsex1] = '2' or [hiv_analsex1] = '4' or [hiv_analsex1]= '5') and ([event-name]='baseline_visit_arm_1' or [event-name]='6_month_visit_arm_1' or [event-name]='12_month_visit_arm_1' or [event-name]='18_month_visit_arm_1') AND [acasi_language]='EN'		
311	[hiv_reason_analsex1_sw]<br><br>Show the field ONLY if: ([last_anal_male] = '1' or [last_anal_male] = '2' or [last_anal_male] = '3' or [last_anal_male] = '4') and ([alter_relationship1] = '1' or [alter_relationship1] = '2' or [alter_relationship1] = '4') and ([hiv_analsex1] = '1' or [hiv_analsex1] = '2' or [hiv_analsex1] = '4' or [hiv_analsex1]= '5') and ([event-name]='baseline_visit_arm_1' or [event-name]='6_month_visit_arm_1' or [event-name]='12_month_visit_arm_1' or [event-name]='18_month_visit_arm_1') AND [acasi_language]='SW'		descriptive<br>(Attachment: SW-Q56.mp3, Display format: Audio file (play in embedded player on page))
312	[hiv_reason_analsex1_dh]<br><br>Show the field ONLY if: ([last_anal_male] = '1' or [last_anal_male] = '2' or [last_anal_male] = '3' or [last_anal_male] = '4') and ([alter_relationship1] = '1' or [alter_relationship1] = '2' or [alter_relationship1] = '4') and ([hiv_analsex1] = '1' or [hiv_analsex1] = '2' or [hiv_analsex1] = '4' or [hiv_analsex1]= '5') and ([event-name]='baseline_visit_arm_1' or [event-name]='6_month_visit_arm_1' or [event-name]='12_month_visit_arm_1' or [event-name]='18_month_visit_arm_1') AND [acasi_language]='DH'		descriptive<br>(Attachment: DH-Q56.mp3, Display format: Audio file (play in embedded player on page))
313	[hiv_reason_analsex1]	Please indicate all the reasons you have for thinking this. (Check all that apply.)	checkbox, Required

	Show the field ONLY if: ([last_anal_male] = '1' or [last_anal_male] = '2' or [last_anal_male] = '3' or [last_anal_male] = '4') and ([alter_relationship1] = '1' or [alter_relationship1] = '2' or [alter_relationship1] = '4') and ([hiv_analsex1] = '1' or [hiv_analsex1] = '2' or [hiv_analsex1] = '4' or [hiv_analsex1]= '5') and ([event-name]='baseline_visit_arm_1' or [event-name]='6_month_visit_arm_1' or [event-name]='12_month_visit_arm_1' or [event-name]='18_month_visit_arm_1')		1 hiv_reason_analsex1___1 We discussed our HIV status<br>2 hiv_reason_analsex1___2 We did couple counseling together<br>3 hiv_reason_analsex1___3 They seemed healthy<br>4 hiv_reason_analsex1___4 They seemed unhealthy<br>5 hiv_reason_analsex1___5 I heard rumours<br>6 hiv_reason_analsex1___6 They told me they are taking antiretroviral treatment<br>7 hiv_reason_analsex1___7 I have seen them taking pills<br>8 hiv_reason_analsex1___8 Don't know<br>9 hiv_reason_analsex1___9 Refuse to answer
314	[intro_q57_eng]<br>Show the field ONLY if: ([last_anal_male] = '1' or [last_anal_male] = '2' or [last_anal_male] = '3' or [last_anal_male] = '4') and [analsex_partners]>1 AND ([event-name]='baseline_visit_arm_1' or [event-name]='6_month_visit_arm_1' or [event-name]='12_month_visit_arm_1' or [event-name]='18_month_visit_arm_1') AND [acasi_language]='EN'	Section Header: *We are now going to ask about the male partner you had anal sex with second most recently in the past 3 months.*	descriptive (Attachment: INST-Q57.mp3, Display format: Audio file (play in embedded player on page))
315	[intro_q57_sw]<br>Show the field ONLY if: ([last_anal_male] = '1' or [last_anal_male] = '2' or [last_anal_male] = '3' or [last_anal_male] = '4') and [analsex_partners]>1 AND ([event-name]='baseline_visit_arm_1' or [event-name]='6_month_visit_arm_1' or [event-name]='12_month_visit_arm_1' or [event-name]='18_month_visit_arm_1') AND [acasi_language]='SW'		descriptive (Attachment: INST-Q57.mp3, Display format: Audio file (play in embedded player on page))

316	[intro_q57_dh]<br><br>Show the field ONLY if:<br>([last_anal_male] = '1' or [last_anal_male] = '2' or [last_anal_male] = '3' or [last_anal_male] = '4') and [analsex_partners]>1 AND ([event-name]='baseline_visit_arm_1' or [event-name]='6_month_visit_arm_1' or [event-name]='12_month_visit_arm_1' or [event-name]='18_month_visit_arm_1') AND [acasi_language]='DH'		descriptive<br>(Attachment: DH-INST-Q57.mp3, Display format: Audio file (play in embedded player on page))
317	[alter_age2_eng]<br><br>Show the field ONLY if:<br>([last_anal_male] = '1' or [last_anal_male] = '2' or [last_anal_male] = '3' or [last_anal_male] = '4') and [analsex_partners]>1 AND ([event-name]='baseline_visit_arm_1' or [event-name]='6_month_visit_arm_1' or [event-name]='12_month_visit_arm_1' or [event-name]='18_month_visit_arm_1') AND [acasi_language]='EN'		descriptive<br>(Attachment: Q57.mp3, Display format: Audio file (play in embedded player on page))
318	[alter_age2_sw]<br><br>Show the field ONLY if:<br>([last_anal_male] = '1' or [last_anal_male] = '2' or [last_anal_male] = '3' or [last_anal_male] = '4') and [analsex_partners]>1 AND ([event-name]='baseline_visit_arm_1' or [event-name]='6_month_visit_arm_1' or [event-name]='12_month_visit_arm_1' or [event-name]='18_month_visit_arm_1') AND [acasi_language]='SW'		descriptive<br>(Attachment: SW-Q57.mp3, Display format: Audio file (play in embedded player on page))
319	[alter_age2_dh]<br><br>Show the field ONLY if:<br>([last_anal_male] = '1' or [last_anal_male] = '2' or [last_anal_male] = '3' or [last_anal_male] = '4') and [analsex_partners]>1 AND ([event-name]='baseline_visit_arm_1' or [event-name]='6_month_visit_arm_1' or [event-name]='12_month_visit_arm_1'		descriptive<br>(Attachment: DH-Q57.mp3, Display format: Audio file (play in embedded player on page))

	or [event-name]='18_month_visit_arm_1') AND [acasi_language]='DH'		
320	[alter_age2]<br><br>Show the field ONLY if: ([last_anal_male] = '1' or [last_anal_male] = '2' or [last_anal_male] = '3' or [last_anal_male] = '4') and [analsex_partners]>1 AND ([event-name]='baseline_visit_arm_1' or [event-name]='6_month_visit_arm_1' or [event-name]='12_month_visit_arm_1' or [event-name]='18_month_visit_arm_1')	What is the age of this person?	radio, Required<br>1 About the same age as me<br>2 More than 5 years older than me<br>3 More than 5 years younger than me<br>4 Refuse to answer
321	[alter_condom2_eng]<br><br>Show the field ONLY if: ([last_anal_male] = '1' or [last_anal_male] = '2' or [last_anal_male] = '3' or [last_anal_male] = '4') and [analsex_partners]>1 AND ([event-name]='baseline_visit_arm_1' or [event-name]='6_month_visit_arm_1' or [event-name]='12_month_visit_arm_1' or [event-name]='18_month_visit_arm_1') AND [acasi_language]='EN'		descriptive<br>(Attachment: Q58.mp3, Display format: Audio file (play in embedded player on page))
322	[alter_condom2_sw]<br><br>Show the field ONLY if: ([last_anal_male] = '1' or [last_anal_male] = '2' or [last_anal_male] = '3' or [last_anal_male] = '4') and [analsex_partners]>1 AND ([event-name]='baseline_visit_arm_1' or [event-name]='6_month_visit_arm_1' or [event-name]='12_month_visit_arm_1' or [event-name]='18_month_visit_arm_1') AND [acasi_language]='SW'		descriptive<br>(Attachment: SW-Q58.mp3, Display format: Audio file (play in embedded player on page))
323	[alter_condom2_dh]<br><br>Show the field ONLY if: ([last_anal_male] = '1' or [last_anal_male] = '2' or [last_anal_male] = '3' or [last_anal_male] = '4') and [analsex_partners]>1 AND ([event-name]='baseline_visit_arm_1' or [event-name]='6_month_visit_		descriptive<br>(Attachment: DH-Q58.mp3, Display format: Audio file (play in embedded player on page))

	arm_1' or [event-name] ='12_month_visit_arm_1' or [event-name]='18_month_visit_arm_1') AND [acasi_language]='DH'		
324	[alter_condom2]<br><br>Show the field ONLY if: ([last_anal_male] = '1' or [last_anal_male] = '2' or [last_anal_male] = '3' or [last_anal_male] = '4') and [analsex_partners]>1 AND ([event-name]='baseline_visit_arm_1' or [event-name]='6_month_visit_arm_1' or [event-name] ='12_month_visit_arm_1' or [event-name]='18_month_visit_arm_1')	When you last had anal sex with this person, did you or your partner use a condom?	radio, Required<br>1 No<br>2 Yes<br>3 Don't remember<br>4 Refuse to answer
325	[alter_doxypep2_eng]<br><br>Show the field ONLY if: ([last_anal_male] = '1' or [last_anal_male] = '2' or [last_anal_male] = '3' or [last_anal_male] = '4') and [analsex_partners]>1 and [randomization_arm_1][randomization_group] ='1' AND ([event-name] ='6_month_visit_arm_1' or [event-name]='12_month_visit_arm_1' or [event-name]='18_month_visit_arm_1') AND [acasi_language]='EN'		descriptive<br>(Attachment: Q59.mp3, Display format: Audio file (play in embedded player on page))
326	[alter_doxypep2_sw]<br><br>Show the field ONLY if: ([last_anal_male] = '1' or [last_anal_male] = '2' or [last_anal_male] = '3' or [last_anal_male] = '4') and [analsex_partners]>1 and [randomization_arm_1][randomization_group] ='1' AND ([event-name] ='6_month_visit_arm_1' or [event-name]='12_month_visit_arm_1' or [event-name]='18_month_visit_arm_1') AND [acasi_language]='SW'		descriptive<br>(Attachment: SW-Q59.mp3, Display format: Audio file (play in embedded player on page))
327	[alter_doxypep2_dh]<br><br>Show the field ONLY if: ([last_anal_male] = '1' or [last_anal_male] = '2' or [last_anal_male] = '3' or [last_anal_male] = '4') an		descriptive<br>(Attachment: DH-Q59.mp3, Display format: Audio file (play in embedded player on page))

	d [analsex_partners]>1 a nd [randomization_arm_ 1][randomization_group] ='1' AND ([event-name] ='6_month_visit_arm_1' or [event-name]='12_mo nth_visit_arm_1' or [even t-name]='18_month_visit _arm_1') AND [acasi_lang uage]='DH'		
328	[alter_doxypep2]<br><br>Show the field ONLY if: ([last_anal_male] = '1' or [last_anal_male] = '2' or [last_anal_male] = '3' or [last_anal_male] = '4') an d [analsex_partners]>1 a nd [randomization_arm_ 1][randomization_group] ='1' AND ([event-name] ='6_month_visit_arm_1' or [event-name]='12_mo nth_visit_arm_1' or [even t-name]='18_month_visit _arm_1')	After you last had anal sex with this person, did you take doxyPEP?	radio, Required<br>1 No<br>2 Yes<br>3 Don't remember<br>4 Refuse to answer
329	[alter_relationship2_e ng]<br><br>Show the field ONLY if: ([last_anal_male] = '1' or [last_anal_male] = '2' or [last_anal_male] = '3' or [last_anal_male] = '4') an d [analsex_partners]>1 A ND ([event-name]='basel ine_visit_arm_1' or [even t-name]='6_month_visit_ arm_1' or [event-name] ='12_month_visit_arm_1' or [event-name]='18_mo nth_visit_arm_1') AND [a casi_language]='EN'	Section Header:	descriptive<br>(Attachment: Q60.mp3, Display format: Audio file (play in embedded player on page))
330	[alter_relationship2_s w]<br><br>Show the field ONLY if: ([last_anal_male] = '1' or [last_anal_male] = '2' or [last_anal_male] = '3' or [last_anal_male] = '4') an d [analsex_partners]>1 A ND ([event-name]='basel ine_visit_arm_1' or [even t-name]='6_month_visit_ arm_1' or [event-name] ='12_month_visit_arm_1' or [event-name]='18_mo nth_visit_arm_1') AND [a casi_language]='SW'		descriptive<br>(Attachment: SW-Q60.mp3, Display format: Audio file (play in embedded player on page))

331	[alter_relationship2_dh]<br><br>Show the field ONLY if:<br>([last_anal_male] = '1' or [last_anal_male] = '2' or [last_anal_male] = '3' or [last_anal_male] = '4') and [analsex_partners]>1 AND ([event-name]='baseline_visit_arm_1' or [event-name]='6_month_visit_arm_1' or [event-name]='12_month_visit_arm_1' or [event-name]='18_month_visit_arm_1') AND [acasi_language]='DH'		descriptive<br>(Attachment: DH-Q60.mp3, Display format: Audio file (play in embedded player on page))
332	[alter_relationship2]<br><br>Show the field ONLY if:<br>([last_anal_male] = '1' or [last_anal_male] = '2' or [last_anal_male] = '3' or [last_anal_male] = '4') and [analsex_partners]>1 AND ([event-name]='baseline_visit_arm_1' or [event-name]='6_month_visit_arm_1' or [event-name]='12_month_visit_arm_1' or [event-name]='18_month_visit_arm_1')	How would you describe your relationship with this person?	radio, Required<br>1 Regular (long-term) partner<br>2 Casual (short-term) partner<br>3 One-time encounter<br>4 Other<br>5 Refuse to answer
333	[other_alter_relationship2]<br><br>Show the field ONLY if:<br>[alter_relationship2] = '4'	Other alter relationship (specify)	text, Required
334	[alter_role2_eng]<br><br>Show the field ONLY if:<br>([last_anal_male] = '1' or [last_anal_male] = '2' or [last_anal_male] = '3' or [last_anal_male] = '4') and [analsex_partners]>1 AND ([event-name]='baseline_visit_arm_1' or [event-name]='6_month_visit_arm_1' or [event-name]='12_month_visit_arm_1' or [event-name]='18_month_visit_arm_1') AND [acasi_language]='EN'		descriptive<br>(Attachment: Q61.mp3, Display format: Audio file (play in embedded player on page))
335	[alter_role2_sw]<br><br>Show the field ONLY if:<br>([last_anal_male] = '1' or [last_anal_male] = '2' or [last_anal_male] = '3' or [last_anal_male] = '4') and [analsex_partners]>1 A		descriptive<br>(Attachment: SW-Q61.mp3, Display format: Audio file (play in embedded player on page))

	ND ([event-name]='baseline_visit_arm_1' or [event-name]='6_month_visit_arm_1' or [event-name]='12_month_visit_arm_1' or [event-name]='18_month_visit_arm_1') AND [acasi_language]='SW'		
336	[alter_role2_dh]<br><br>Show the field ONLY if: ([last_anal_male] = '1' or [last_anal_male] = '2' or [last_anal_male] = '3' or [last_anal_male] = '4') and [analsex_partners]>1 AND ([event-name]='baseline_visit_arm_1' or [event-name]='6_month_visit_arm_1' or [event-name]='12_month_visit_arm_1' or [event-name]='18_month_visit_arm_1') AND [acasi_language]='DH'		descriptive<br>(Attachment: DH-Q61.mp3, Display format: Audio file (play in embedded player on page))
337	[alter_role2]<br><br>Show the field ONLY if: ([last_anal_male] = '1' or [last_anal_male] = '2' or [last_anal_male] = '3' or [last_anal_male] = '4') and [analsex_partners]>1 AND ([event-name]='baseline_visit_arm_1' or [event-name]='6_month_visit_arm_1' or [event-name]='12_month_visit_arm_1' or [event-name]='18_month_visit_arm_1')	When you had anal sex with this partner, what role did you take?	radio, Required<br>1 Insertive (top) only<br>2 Mostly insertive (top)<br>3 About 50/50 insertive (top) and receptive (bottom)<br>4 Mostly receptive (bottom)<br>5 Receptive (bottom) only<br>6 Refuse to answer
338	[date_lastanalsex2_eng]<br><br>Show the field ONLY if: ([last_anal_male] = '1' or [last_anal_male] = '2' or [last_anal_male] = '3' or [last_anal_male] = '4') and [analsex_partners]>1 and ([alter_relationship2] = '1' or [alter_relationship2] = '2' or [alter_relationship2] = '4') AND ([event-name]='baseline_visit_arm_1' or [event-name]='6_month_visit_arm_1' or [event-name]='12_month_visit_arm_1' or [event-name]='18_month_visit_arm_1') AND [acasi_language]='EN'		descriptive<br>(Attachment: Q62.mp3, Display format: Audio file (play in embedded player on page))

339	[date_lastanalsex2_sw]<br><br>Show the field ONLY if: ([last_anal_male] = '1' or [last_anal_male] = '2' or [last_anal_male] = '3' or [last_anal_male] = '4') and [analsex_partners]>1 and ([alter_relationship2] = '1' or [alter_relationship2] = '2' or [alter_relationship2] = '4') AND ([event-name]='baseline_visit_arm_1' or [event-name]='6_month_visit_arm_1' or [event-name]='12_month_visit_arm_1' or [event-name]='18_month_visit_arm_1') AND [acasi_language]='SW'		descriptive<br>(Attachment: SW-Q62.mp3, Display format: Audio file (play in embedded player on page))
340	[date_lastanalsex2_dh]<br><br>Show the field ONLY if: ([last_anal_male] = '1' or [last_anal_male] = '2' or [last_anal_male] = '3' or [last_anal_male] = '4') and [analsex_partners]>1 and ([alter_relationship2] = '1' or [alter_relationship2] = '2' or [alter_relationship2] = '4') AND ([event-name]='baseline_visit_arm_1' or [event-name]='6_month_visit_arm_1' or [event-name]='12_month_visit_arm_1' or [event-name]='18_month_visit_arm_1') AND [acasi_language]='DH'		descriptive<br>(Attachment: DH-Q62.mp3, Display format: Audio file (play in embedded player on page))
341	[date_lastanalsex2]<br><br>Show the field ONLY if: ([last_anal_male] = '1' or [last_anal_male] = '2' or [last_anal_male] = '3' or [last_anal_male] = '4') and [analsex_partners]>1 and ([alter_relationship2] = '1' or [alter_relationship2] = '2' or [alter_relationship2] = '4') AND ([event-name]='baseline_visit_arm_1' or [event-name]='6_month_visit_arm_1' or [event-name]='12_month_visit_arm_1' or [event-name]='18_month_visit_arm_1')	When did you last have anal sex with this partner? If you do not know the exact date, please make your best guess.	text (date_dmy), Required
342	[date_firstanalsex2_eng]	Section Header:	descriptive<br>(Attachment: Q63.mp3, Display format:

	Show the field ONLY if: ([last_anal_male] = '1' or [last_anal_male] = '2' or [last_anal_male] = '3' or [last_anal_male] = '4') and [analsex_partners]>1 and ([alter_relationship2] = '1' or [alter_relationship2] = '2' or [alter_relationship2] = '4') AND ([event-name]='baseline_visit_arm_1' or [event-name]='6_month_visit_arm_1' or [event-name]='12_month_visit_arm_1' or [event-name]='18_month_visit_arm_1') AND [acasi_language]='EN'		Audio file (play in embedded player on page))
343	[date_firstanalsex2_sw]<br><br>Show the field ONLY if: ([last_anal_male] = '1' or [last_anal_male] = '2' or [last_anal_male] = '3' or [last_anal_male] = '4') and [analsex_partners]>1 and ([alter_relationship2] = '1' or [alter_relationship2] = '2' or [alter_relationship2] = '4') AND ([event-name]='baseline_visit_arm_1' or [event-name]='6_month_visit_arm_1' or [event-name]='12_month_visit_arm_1' or [event-name]='18_month_visit_arm_1') AND [acasi_language]='SW'		descriptive<br>(Attachment: SW-Q63.mp3, Display format: Audio file (play in embedded player on page))
344	[date_firstanalsex2_dh]<br><br>Show the field ONLY if: ([last_anal_male] = '1' or [last_anal_male] = '2' or [last_anal_male] = '3' or [last_anal_male] = '4') and [analsex_partners]>1 and ([alter_relationship2] = '1' or [alter_relationship2] = '2' or [alter_relationship2] = '4') AND ([event-name]='baseline_visit_arm_1' or [event-name]='6_month_visit_arm_1' or [event-name]='12_month_visit_arm_1' or [event-name]='18_month_visit_arm_1') AND [acasi_language]='DH'		descriptive<br>(Attachment: DH-Q63.mp3, Display format: Audio file (play in embedded player on page))

345	[date_firstanalsex2]<br><br>Show the field ONLY if: ([last_anal_male] = '1' or [last_anal_male] = '2' or [last_anal_male] = '3' or [last_anal_male] = '4') and [analsex_partners]>1 and ([alter_relationship2] = '1' or [alter_relationship2] = '2' or [alter_relationship2] = '4') AND ([event-name]='baseline_visit_arm_1' or [event-name]='6_month_visit_arm_1' or [event-name]='12_month_visit_arm_1' or [event-name]='18_month_visit_arm_1')	When did you have anal sex with this partner for the very first time? If you do not know the exact date, please make your best guess.	text (date_dmy), Required
346	[partner2_ongoing_eng]<br><br>Show the field ONLY if: ([last_anal_male] = '1' or [last_anal_male] = '2' or [last_anal_male] = '3' or [last_anal_male] = '4') and [analsex_partners]>1 and ([alter_relationship2] = '1' or [alter_relationship2] = '2' or [alter_relationship2] = '4') AND ([event-name]='baseline_visit_arm_1' or [event-name]='6_month_visit_arm_1' or [event-name]='12_month_visit_arm_1' or [event-name]='18_month_visit_arm_1') AND [acasi_language]='EN'		descriptive<br>(Attachment: Q64.mp3, Display format: Audio file (play in embedded player on page))
347	[partner2_ongoing_sw]<br><br>Show the field ONLY if: ([last_anal_male] = '1' or [last_anal_male] = '2' or [last_anal_male] = '3' or [last_anal_male] = '4') and [analsex_partners]>1 and ([alter_relationship2] = '1' or [alter_relationship2] = '2' or [alter_relationship2] = '4') AND ([event-name]='baseline_visit_arm_1' or [event-name]='6_month_visit_arm_1' or [event-name]='12_month_visit_arm_1' or [event-name]='18_month_visit_arm_1') AND [acasi_language]='SW'		descriptive<br>(Attachment: SW-Q64.mp3, Display format: Audio file (play in embedded player on page))
348	[partner2_ongoing_dh]		descriptive<br>(Attachment: DH-Q64.mp3, Display format:

	Show the field ONLY if: ([last_anal_male] = '1' or [last_anal_male] = '2' or [last_anal_male] = '3' or [last_anal_male] = '4') and [analsex_partners]>1 and ([alter_relationship2] = '1' or [alter_relationship2] = '2' or [alter_relationship2] = '4') AND ([event-name]='baseline_visit_arm_1' or [event-name]='6_month_visit_arm_1' or [event-name]='12_month_visit_arm_1' or [event-name]='18_month_visit_arm_1') AND [acasi_language]='DH'		Audio file (play in embedded player on page))
349	[partner2_ongoing]<br><br>Show the field ONLY if: ([last_anal_male] = '1' or [last_anal_male] = '2' or [last_anal_male] = '3' or [last_anal_male] = '4') and [analsex_partners]>1 and ([alter_relationship2] = '1' or [alter_relationship2] = '2' or [alter_relationship2] = '4') AND ([event-name]='baseline_visit_arm_1' or [event-name]='6_month_visit_arm_1' or [event-name]='12_month_visit_arm_1' or [event-name]='18_month_visit_arm_1')	Do you expect to have anal sex with this partner again?	radio<br>1 No<br>2 Yes<br>3 Don't Know
350	[freq_analsex2_eng]<br><br>Show the field ONLY if: ([last_anal_male] = '1' or [last_anal_male] = '2' or [last_anal_male] = '3' or [last_anal_male] = '4') and [analsex_partners]>1 and ([alter_relationship2] = '1' or [alter_relationship2] = '2' or [alter_relationship2] = '4') AND [partner2_ongoing]='2' AND ([event-name]='baseline_visit_arm_1' or [event-name]='6_month_visit_arm_1' or [event-name]='12_month_visit_arm_1' or [event-name]='18_month_visit_arm_1') AND [acasi_language]='EN'		descriptive<br>(Attachment: Q65.mp3, Display format: Audio file (play in embedded player on page))
351	[freq_analsex2_sw]<br><br>Show the field ONLY if:		descriptive<br>(Attachment: Q65 K.mp3, Display format:

	([last_anal_male] = '1' or [last_anal_male] = '2' or [last_anal_male] = '3' or [last_anal_male] = '4') and [analsex_partners]>1 and ([alter_relationship2] = '1' or [alter_relationship2] = '2' or [alter_relationship2] = '4') AND [partner2_ongoing]='2' AND ([event-name]='baseline_visit_arm_1' or [event-name]='6_month_visit_arm_1' or [event-name]='12_month_visit_arm_1' or [event-name]='18_month_visit_arm_1') AND [acasi_language]='SW'		Audio file (play in embedded player on page))
352	[freq_analsex2_dh]<br><br>Show the field ONLY if:<br>([last_anal_male] = '1' or [last_anal_male] = '2' or [last_anal_male] = '3' or [last_anal_male] = '4') and [analsex_partners]>1 and ([alter_relationship2] = '1' or [alter_relationship2] = '2' or [alter_relationship2] = '4') AND [partner2_ongoing]='2' AND ([event-name]='baseline_visit_arm_1' or [event-name]='6_month_visit_arm_1' or [event-name]='12_month_visit_arm_1' or [event-name]='18_month_visit_arm_1') AND [acasi_language]='DH'		descriptive<br>(Attachment: DH-Q65.mp3, Display format: Audio file (play in embedded player on page))
353	[freq_analsex2]<br><br>Show the field ONLY if:<br>([last_anal_male] = '1' or [last_anal_male] = '2' or [last_anal_male] = '3' or [last_anal_male] = '4') and [analsex_partners]>1 and ([alter_relationship2] = '1' or [alter_relationship2] = '2' or [alter_relationship2] = '4') AND [partner2_ongoing]='2' AND ([event-name]='baseline_visit_arm_1' or [event-name]='6_month_visit_arm_1' or [event-name]='12_month_visit_arm_1' or [event-name]='18_month_visit_arm_1')	How often have you had anal sex with this partner in an average week?	radio, Required<br>1 Daily<br>2 Almost every day<br>3 Once a week<br>4 Less than once a week<br>5 Refuse to answer
354	[condom_analsex2_eng]	Section Header:	descriptive<br>(Attachment: Q66.mp3, Display format:

	Show the field ONLY if: ([last_anal_male] = '1' or [last_anal_male] = '2' or [last_anal_male] = '3' or [last_anal_male] = '4') and [analsex_partners]>1 and ([alter_relationship2] = '1' or [alter_relationship2] = '2' or [alter_relationship2] = '4') AND ([event-name]='baseline_visit_arm_1' or [event-name]='6_month_visit_arm_1' or [event-name]='12_month_visit_arm_1' or [event-name]='18_month_visit_arm_1') AND [acasi_language]='EN'		Audio file (play in embedded player on page))
355	[condom_analsex2_sw]<br><br>Show the field ONLY if: ([last_anal_male] = '1' or [last_anal_male] = '2' or [last_anal_male] = '3' or [last_anal_male] = '4') and [analsex_partners]>1 and ([alter_relationship2] = '1' or [alter_relationship2] = '2' or [alter_relationship2] = '4') AND ([event-name]='baseline_visit_arm_1' or [event-name]='6_month_visit_arm_1' or [event-name]='12_month_visit_arm_1' or [event-name]='18_month_visit_arm_1') AND [acasi_language]='SW'		descriptive<br>(Attachment: SW-Q66.mp3, Display format: Audio file (play in embedded player on page))
356	[condom_analsex2_dh]<br><br>Show the field ONLY if: ([last_anal_male] = '1' or [last_anal_male] = '2' or [last_anal_male] = '3' or [last_anal_male] = '4') and [analsex_partners]>1 and ([alter_relationship2] = '1' or [alter_relationship2] = '2' or [alter_relationship2] = '4') AND ([event-name]='baseline_visit_arm_1' or [event-name]='6_month_visit_arm_1' or [event-name]='12_month_visit_arm_1' or [event-name]='18_month_visit_arm_1') AND [acasi_language]='DH'		descriptive<br>(Attachment: DH-Q66.mp3, Display format: Audio file (play in embedded player on page))
357	[condom_analsex2]<br><br>Show the field ONLY if:	When you have had anal sex with this partner, how often did you or your partner use a condom?	radio, Required<br>1 Always

	([last_anal_male] = '1' or [last_anal_male] = '2' or [last_anal_male] = '3' or [last_anal_male] = '4') and [analsex_partners]>1 and ([alter_relationship2] = '1' or [alter_relationship2] = '2' or [alter_relationship2] = '4') AND ([event-name]='baseline_visit_arm_1' or [event-name]='6_month_visit_arm_1' or [event-name]='12_month_visit_arm_1' or [event-name]='18_month_visit_arm_1')		2 Most of the time<br>3 About half the time<br>4 Rarely<br>5 Never<br>6 Refuse to answer
358	[hiv_analsex2_eng]<br>Show the field ONLY if: ([last_anal_male] = '1' or [last_anal_male] = '2' or [last_anal_male] = '3' or [last_anal_male] = '4') and [analsex_partners]>1 and ([alter_relationship2] = '1' or [alter_relationship2] = '2' or [alter_relationship2] = '4') AND ([event-name]='baseline_visit_arm_1' or [event-name]='6_month_visit_arm_1' or [event-name]='12_month_visit_arm_1' or [event-name]='18_month_visit_arm_1') AND [acasi_language]='EN'		descriptive<br>(Attachment: Q67.mp3, Display format: Audio file (play in embedded player on page))
359	[hiv_analsex2_sw]<br>Show the field ONLY if: ([last_anal_male] = '1' or [last_anal_male] = '2' or [last_anal_male] = '3' or [last_anal_male] = '4') and [analsex_partners]>1 and ([alter_relationship2] = '1' or [alter_relationship2] = '2' or [alter_relationship2] = '4') AND ([event-name]='baseline_visit_arm_1' or [event-name]='6_month_visit_arm_1' or [event-name]='12_month_visit_arm_1' or [event-name]='18_month_visit_arm_1') AND [acasi_language]='SW'		descriptive<br>(Attachment: SW-Q67.mp3, Display format: Audio file (play in embedded player on page))
360	[hiv_analsex2_dh]<br>Show the field ONLY if: ([last_anal_male] = '1' or [last_anal_male] = '2' or		descriptive<br>(Attachment: DH-Q67.mp3, Display format: Audio file (play in embedded player on page))

	[last_anal_male] = '3' or [last_anal_male] = '4') and [analsex_partners]>1 and ([alter_relationship2] = '1' or [alter_relationship2] = '2' or [alter_relationship2] = '4') AND ([event-name]='baseline_visit_arm_1' or [event-name]='6_month_visit_arm_1' or [event-name]='12_month_visit_arm_1' or [event-name]='18_month_visit_arm_1') AND [acasi_language]='DH'		
361	[hiv_analsex2]<br><br>Show the field ONLY if: ([last_anal_male] = '1' or [last_anal_male] = '2' or [last_anal_male] = '3' or [last_anal_male] = '4') and [analsex_partners]>1 and ([alter_relationship2] = '1' or [alter_relationship2] = '2' or [alter_relationship2] = '4') AND ([event-name]='baseline_visit_arm_1' or [event-name]='6_month_visit_arm_1' or [event-name]='12_month_visit_arm_1' or [event-name]='18_month_visit_arm_1')	What do you think this partner's HIV status is?	radio, Required<br>1 HIV positive<br>2 Probably HIV positive<br>3 I have no idea<br>4 Probably HIV negative<br>5 HIV negative<br>6 Refuse to answer
362	[hiv_reason_analsex2_eng]<br><br>Show the field ONLY if: ([hiv_analsex2]='1' or [hiv_analsex2] = '2' or [hiv_analsex2] = '4' or [hiv_analsex2]= '5') and ([event-name]='baseline_visit_arm_1' or [event-name]='6_month_visit_arm_1' or [event-name]='12_month_visit_arm_1' or [event-name]='18_month_visit_arm_1') AND [acasi_language]='EN'		descriptive<br>(Attachment: Q68.mp3, Display format: Audio file (play in embedded player on page))
363	[hiv_reason_analsex2_sw]<br><br>Show the field ONLY if: ([hiv_analsex2]='1' or [hiv_analsex2] = '2' or [hiv_analsex2] = '4' or [hiv_analsex2]= '5') and ([event-name]='baseline_visit_arm_1' or [event-name]='6_month_visit_arm_1' or		descriptive<br>(Attachment: SW-Q68.mp3, Display format: Audio file (play in embedded player on page))

	[event-name]='12_month_visit_arm_1' or [event-name]='18_month_visit_arm_1') AND [acasi_language]='SW'		
364	[hiv_reason_analsex2_dh]<br><br>Show the field ONLY if: ([hiv_analsex2]='1' or [hiv_analsex2] = '2' or [hiv_analsex2] = '4' or [hiv_analsex2]= '5') and ([event-name]='baseline_visit_arm_1' or [event-name]='6_month_visit_arm_1' or [event-name]='12_month_visit_arm_1' or [event-name]='18_month_visit_arm_1') AND [acasi_language]='DH'		descriptive<br>(Attachment: DH-Q68.mp3, Display format: Audio file (play in embedded player on page))
365	[hiv_reason_analsex2]<br><br>Show the field ONLY if: ([hiv_analsex2]='1' or [hiv_analsex2] = '2' or [hiv_analsex2] = '4' or [hiv_analsex2]= '5') and ([event-name]='baseline_visit_arm_1' or [event-name]='6_month_visit_arm_1' or [event-name]='12_month_visit_arm_1' or [event-name]='18_month_visit_arm_1')	Please indicate all the reasons you have for thinking this. (Check all that apply.)	checkbox, Required<br>1 | hiv_reason_analsex2___1 | We discussed our HIV status<br>2 | hiv_reason_analsex2___2 | We did couple counseling together<br>3 | hiv_reason_analsex2___3 | They seemed healthy<br>4 | hiv_reason_analsex2___4 | They seemed unhealthy<br>5 | hiv_reason_analsex2___5 | I heard rumours<br>6 | hiv_reason_analsex2___6 | They told me they are taking antiretroviral treatment<br>7 | hiv_reason_analsex2___7 | I have seen them taking pills<br>8 | hiv_reason_analsex2___8 | Don't know<br>9 | hiv_reason_analsex2___9 | Refuse to answer
366	[int_q69_en]<br><br>Show the field ONLY if: ([last_anal_male] = '1' or [last_anal_male] = '2' or [last_anal_male] = '3' or [last_anal_male] = '4') and [analsex_partners]>2 AND ([event-name]='baseline_visit_arm_1' or [even	Section Header: *We are now going to ask about the male partner you had anal sex with third most recently in the past 3 months.*	descriptive<br>(Attachment: INST-Q69.mp3, Display format: Audio file (play in embedded player on page))

	t-name]='6_month_visit_arm_1' or [event-name]='12_month_visit_arm_1' or [event-name]='18_month_visit_arm_1') AND [acasi_language]='EN'		
367	[int_q69_sw]<br><br>Show the field ONLY if:<br>([last_anal_male] = '1' or [last_anal_male] = '2' or [last_anal_male] = '3' or [last_anal_male] = '4') and [analsex_partners]>2 AND ([event-name]='baseline_visit_arm_1' or [event-name]='6_month_visit_arm_1' or [event-name]='12_month_visit_arm_1' or [event-name]='18_month_visit_arm_1') AND [acasi_language]='SW'		descriptive<br>(Attachment: SW-INST-Q69.mp3, Display format: Audio file (play in embedded player on page))
368	[int_q69_dh]<br><br>Show the field ONLY if:<br>([last_anal_male] = '1' or [last_anal_male] = '2' or [last_anal_male] = '3' or [last_anal_male] = '4') and [analsex_partners]>2 AND ([event-name]='baseline_visit_arm_1' or [event-name]='6_month_visit_arm_1' or [event-name]='12_month_visit_arm_1' or [event-name]='18_month_visit_arm_1') AND [acasi_language]='DH'		descriptive<br>(Attachment: DH-INST-Q69.mp3, Display format: Audio file (play in embedded player on page))
369	[alter_age3_eng]<br><br>Show the field ONLY if:<br>([last_anal_male] = '1' or [last_anal_male] = '2' or [last_anal_male] = '3' or [last_anal_male] = '4') and [analsex_partners]>2 AND ([event-name]='baseline_visit_arm_1' or [event-name]='6_month_visit_arm_1' or [event-name]='12_month_visit_arm_1' or [event-name]='18_month_visit_arm_1') AND [acasi_language]='EN'		descriptive<br>(Attachment: Q69.mp3, Display format: Audio file (play in embedded player on page))
370	[alter_age3_sw]<br><br>Show the field ONLY if:<br>([last_anal_male] = '1' or [last_anal_male] = '2' or [last_anal_male] = '3' or [last_anal_male] = '4') an		descriptive<br>(Attachment: SW-Q69.mp3, Display format: Audio file (play in embedded player on page))

	d [analsex_partners]>2 AND ([event-name]='baseline_visit_arm_1' or [event-name]='6_month_visit_arm_1' or [event-name]='12_month_visit_arm_1' or [event-name]='18_month_visit_arm_1') AND [acasi_language]='SW'		
371	[alter_age3_dh]<br><br>Show the field ONLY if:<br>([last_anal_male] = '1' or [last_anal_male] = '2' or [last_anal_male] = '3' or [last_anal_male] = '4') and [analsex_partners]>2 AND ([event-name]='baseline_visit_arm_1' or [event-name]='6_month_visit_arm_1' or [event-name]='12_month_visit_arm_1' or [event-name]='18_month_visit_arm_1') AND [acasi_language]='DH'		descriptive<br>(Attachment: DH-Q69.mp3, Display format: Audio file (play in embedded player on page))
372	[alter_age3]<br><br>Show the field ONLY if:<br>([last_anal_male] = '1' or [last_anal_male] = '2' or [last_anal_male] = '3' or [last_anal_male] = '4') and [analsex_partners]>2 AND ([event-name]='baseline_visit_arm_1' or [event-name]='6_month_visit_arm_1' or [event-name]='12_month_visit_arm_1' or [event-name]='18_month_visit_arm_1')	What is the age of this person?	radio, Required<br>1 About the same age as me<br>2 More than 5 years older than me<br>3 More than 5 years younger than me<br>4 Refuse to answer
373	[alter_condom3_eng]<br><br>Show the field ONLY if:<br>([last_anal_male] = '1' or [last_anal_male] = '2' or [last_anal_male] = '3' or [last_anal_male] = '4') and [analsex_partners]>2 AND ([event-name]='baseline_visit_arm_1' or [event-name]='6_month_visit_arm_1' or [event-name]='12_month_visit_arm_1' or [event-name]='18_month_visit_arm_1') AND [acasi_language]='EN'		descriptive<br>(Attachment: Q70.mp3, Display format: Audio file (play in embedded player on page))
374	[alter_condom3_sw]<br><br>Show the field ONLY if:<br>([last_anal_male] = '1' or [last_anal_male] = '2' or		descriptive<br>(Attachment: SW-Q70.mp3, Display format: Audio file (play in embedded player on page))

	[last_anal_male] = '3' or [last_anal_male] = '4') and [analsex_partners]>2 AND ([event-name]='baseline_visit_arm_1' or [event-name]='6_month_visit_arm_1' or [event-name]='12_month_visit_arm_1' or [event-name]='18_month_visit_arm_1') AND [acasi_language]='SW'		
375	[alter_condom3_dh]<br><br>Show the field ONLY if:<br>([last_anal_male] = '1' or [last_anal_male] = '2' or [last_anal_male] = '3' or [last_anal_male] = '4') and [analsex_partners]>2 AND ([event-name]='baseline_visit_arm_1' or [event-name]='6_month_visit_arm_1' or [event-name]='12_month_visit_arm_1' or [event-name]='18_month_visit_arm_1') AND [acasi_language]='DH'		descriptive<br>(Attachment: DH-Q70.mp3, Display format: Audio file (play in embedded player on page))
376	[alter_condom3]<br><br>Show the field ONLY if:<br>([last_anal_male] = '1' or [last_anal_male] = '2' or [last_anal_male] = '3' or [last_anal_male] = '4') and [analsex_partners]>2 AND ([event-name]='baseline_visit_arm_1' or [event-name]='6_month_visit_arm_1' or [event-name]='12_month_visit_arm_1' or [event-name]='18_month_visit_arm_1')	When you last had anal sex with this person, did you or your partner use a condom?	radio, Required<br>1 No<br>2 Yes<br>3 Don't remember<br>4 Refuse to answer
377	[alter_doxypep3_eng]<br><br>Show the field ONLY if:<br>([last_anal_male] = '1' or [last_anal_male] = '2' or [last_anal_male] = '3' or [last_anal_male] = '4') and [analsex_partners]>2 and [randomization_arm_1][randomization_group] = '1' and ([event-name]='6_month_visit_arm_1' or [event-name]='12_month_visit_arm_1' or [event-name]='18_month_visit_arm_1') AND [acasi_language]='EN'		descriptive<br>(Attachment: Q71.mp3, Display format: Audio file (play in embedded player on page))
378	[alter_doxypep3_sw]		descriptive<br>(Attachment: SW-Q71.mp3, Display format:

	Show the field ONLY if: ([last_anal_male] = '1' or [last_anal_male] = '2' or [last_anal_male] = '3' or [last_anal_male] = '4') and [analsex_partners]>2 and [randomization_arm_1][randomization_group] = '1' and ([event-name] ='6_month_visit_arm_1' or [event-name]='12_month_visit_arm_1' or [event-name]='18_month_visit_arm_1') AND [acasi_language]='SW'		Audio file (play in embedded player on page))
379	[alter_doxypep3_dh]<br><br>Show the field ONLY if: ([last_anal_male] = '1' or [last_anal_male] = '2' or [last_anal_male] = '3' or [last_anal_male] = '4') and [analsex_partners]>2 and [randomization_arm_1][randomization_group] = '1' and ([event-name] ='6_month_visit_arm_1' or [event-name]='12_month_visit_arm_1' or [event-name]='18_month_visit_arm_1') AND [acasi_language]='DH'		descriptive<br>(Attachment: DH-Q71.mp3, Display format: Audio file (play in embedded player on page))
380	[alter_doxypep3]<br><br>Show the field ONLY if: ([last_anal_male] = '1' or [last_anal_male] = '2' or [last_anal_male] = '3' or [last_anal_male] = '4') and [analsex_partners]>2 and [randomization_arm_1][randomization_group] = '1' and ([event-name] ='6_month_visit_arm_1' or [event-name]='12_month_visit_arm_1' or [event-name]='18_month_visit_arm_1')	After you last had anal sex with this person, did you take doxyPEP?	radio, Required<br>1 No<br>2 Yes<br>3 Don't remember<br>4 Refuse to answer
381	[alter_relationship3_eng]<br><br>Show the field ONLY if: ([last_anal_male] = '1' or [last_anal_male] = '2' or [last_anal_male] = '3' or [last_anal_male] = '4') and [analsex_partners]>2 and ([event-name]='baseline_visit_arm_1' or [event-name]='6_month_visit_arm_1' or [event-name]	Section Header:	descriptive<br>(Attachment: Q72.mp3, Display format: Audio file (play in embedded player on page))

	='12_month_visit_arm_1' or [event-name]='18_month_visit_arm_1') AND [acasi_language]='EN'		
382	[alter_relationship3_sw]<br><br>Show the field ONLY if:<br>([last_anal_male] = '1' or [last_anal_male] = '2' or [last_anal_male] = '3' or [last_anal_male] = '4') and [analsex_partners]>2 and ([event-name]='baseline_visit_arm_1' or [event-name]='6_month_visit_arm_1' or [event-name]='12_month_visit_arm_1' or [event-name]='18_month_visit_arm_1') AND [acasi_language]='SW'		descriptive<br>(Attachment: SW-Q72.mp3, Display format: Audio file (play in embedded player on page))
383	[alter_relationship3_dh]<br><br>Show the field ONLY if:<br>([last_anal_male] = '1' or [last_anal_male] = '2' or [last_anal_male] = '3' or [last_anal_male] = '4') and [analsex_partners]>2 and ([event-name]='baseline_visit_arm_1' or [event-name]='6_month_visit_arm_1' or [event-name]='12_month_visit_arm_1' or [event-name]='18_month_visit_arm_1') AND [acasi_language]='DH'		descriptive<br>(Attachment: DH-Q72.mp3, Display format: Audio file (play in embedded player on page))
384	[alter_relationship3]<br><br>Show the field ONLY if:<br>([last_anal_male] = '1' or [last_anal_male] = '2' or [last_anal_male] = '3' or [last_anal_male] = '4') and [analsex_partners]>2 and ([event-name]='baseline_visit_arm_1' or [event-name]='6_month_visit_arm_1' or [event-name]='12_month_visit_arm_1' or [event-name]='18_month_visit_arm_1')	How would you describe your relationship with this person?	radio, Required<br>1 Regular (long-term) partner<br>2 Casual (short-term) partner<br>3 One-time encounter<br>4 Other (Specify)<br>5 Refuse to answer
385	[other_alter_relationship3]<br><br>Show the field ONLY if:<br>[alter_relationship3] = '4'	Other relationship (specify)	text, Required
386	[alter_role3_eng]<br><br>Show the field ONLY if:		descriptive<br>(Attachment: Q73.mp3, Display format:

	([last_anal_male] = '1' or [last_anal_male] = '2' or [last_anal_male] = '3' or [last_anal_male] = '4') and [analsex_partners]>2 and ([event-name]='baseline_visit_arm_1' or [event-name]='6_month_visit_arm_1' or [event-name]='12_month_visit_arm_1' or [event-name]='18_month_visit_arm_1') AND [acasi_language]='EN'		Audio file (play in embedded player on page))
387	[alter_role3_sw]<br><br>Show the field ONLY if:<br>([last_anal_male] = '1' or [last_anal_male] = '2' or [last_anal_male] = '3' or [last_anal_male] = '4') and [analsex_partners]>2 and ([event-name]='baseline_visit_arm_1' or [event-name]='6_month_visit_arm_1' or [event-name]='12_month_visit_arm_1' or [event-name]='18_month_visit_arm_1') AND [acasi_language]='SW'		descriptive<br>(Attachment: SW-Q73.mp3, Display format: Audio file (play in embedded player on page))
388	[alter_role3_dh]<br><br>Show the field ONLY if:<br>([last_anal_male] = '1' or [last_anal_male] = '2' or [last_anal_male] = '3' or [last_anal_male] = '4') and [analsex_partners]>2 and ([event-name]='baseline_visit_arm_1' or [event-name]='6_month_visit_arm_1' or [event-name]='12_month_visit_arm_1' or [event-name]='18_month_visit_arm_1') AND [acasi_language]='DH'		descriptive<br>(Attachment: DH-Q73.mp3, Display format: Audio file (play in embedded player on page))
389	[alter_role3]<br><br>Show the field ONLY if:<br>([last_anal_male] = '1' or [last_anal_male] = '2' or [last_anal_male] = '3' or [last_anal_male] = '4') and [analsex_partners]>2 and ([event-name]='baseline_visit_arm_1' or [event-name]='6_month_visit_arm_1' or [event-name]='12_month_visit_arm_1' or [event-name]='18_month_visit_arm_1')	When you had anal sex with this partner, what role did you take?	radio, Required<br>1 Insertive (top) only<br>2 Mostly insertive (top)<br>3 About 50/50 insertive (top) and receptive (bottom)<br>4 Mostly receptive (bottom)<br>5 Receptive (bottom) only<br>6 Refuse to answer

390	[date_lastanalsex3_eng]<br><br>Show the field ONLY if: ([last_anal_male] = '1' or [last_anal_male] = '2' or [last_anal_male] = '3' or [last_anal_male] = '4') and [analsex_partners]>2 and ([alter_relationship3] = '1' or [alter_relationship3] = '2' or [alter_relationship3] = '4') and ([event-name]='baseline_visit_arm_1' or [event-name]='6_month_visit_arm_1' or [event-name]='12_month_visit_arm_1' or [event-name]='18_month_visit_arm_1') AND [acasi_language]='EN'		descriptive<br>(Attachment: Q74.mp3, Display format: Audio file (play in embedded player on page))
391	[date_lastanalsex3_sw]<br><br>Show the field ONLY if: ([last_anal_male] = '1' or [last_anal_male] = '2' or [last_anal_male] = '3' or [last_anal_male] = '4') and [analsex_partners]>2 and ([alter_relationship3] = '1' or [alter_relationship3] = '2' or [alter_relationship3] = '4') and ([event-name]='baseline_visit_arm_1' or [event-name]='6_month_visit_arm_1' or [event-name]='12_month_visit_arm_1' or [event-name]='18_month_visit_arm_1') AND [acasi_language]='SW'		descriptive<br>(Attachment: SW-Q74.mp3, Display format: Audio file (play in embedded player on page))
392	[date_lastanalsex3_dh]<br><br>Show the field ONLY if: ([last_anal_male] = '1' or [last_anal_male] = '2' or [last_anal_male] = '3' or [last_anal_male] = '4') and [analsex_partners]>2 and ([alter_relationship3] = '1' or [alter_relationship3] = '2' or [alter_relationship3] = '4') and ([event-name]='baseline_visit_arm_1' or [event-name]='6_month_visit_arm_1' or [event-name]='12_month_visit_arm_1' or [event-name]='18_month_visit_arm_1') AND [acasi_language]='DH'		descriptive<br>(Attachment: DH-Q74.mp3, Display format: Audio file (play in embedded player on page))

393	[date_lastanalsex3]<br>Show the field ONLY if: ([last_anal_male] = '1' or [last_anal_male] = '2' or [last_anal_male] = '3' or [last_anal_male] = '4') and [analsex_partners]>2 and ([alter_relationship3] = '1' or [alter_relationship3] = '2' or [alter_relationship3] = '4') and ([event-name]='baseline_visit_arm_1' or [event-name]='6_month_visit_arm_1' or [event-name]='12_month_visit_arm_1' or [event-name]='18_month_visit_arm_1')	When did you last have anal sex with this partner? If you do not know the exact date, please make your best guess.	text (date_dmy), Required
394	[date_firstanalsex3_eng]<br>Show the field ONLY if: ([last_anal_male] = '1' or [last_anal_male] = '2' or [last_anal_male] = '3' or [last_anal_male] = '4') and [analsex_partners]>2 and ([alter_relationship3] = '1' or [alter_relationship3] = '2' or [alter_relationship3] = '4') and ([event-name]='baseline_visit_arm_1' or [event-name]='6_month_visit_arm_1' or [event-name]='12_month_visit_arm_1' or [event-name]='18_month_visit_arm_1') AND [acasi_language]='EN'	Section Header:	descriptive<br>(Attachment: Q75.mp3, Display format: Audio file (play in embedded player on page))
395	[date_firstanalsex3_sw]<br>Show the field ONLY if: ([last_anal_male] = '1' or [last_anal_male] = '2' or [last_anal_male] = '3' or [last_anal_male] = '4') and [analsex_partners]>2 and ([alter_relationship3] = '1' or [alter_relationship3] = '2' or [alter_relationship3] = '4') and ([event-name]='baseline_visit_arm_1' or [event-name]='6_month_visit_arm_1' or [event-name]='12_month_visit_arm_1' or [event-name]='18_month_visit_arm_1') AND [acasi_language]='SW'		descriptive<br>(Attachment: SW-Q75.mp3, Display format: Audio file (play in embedded player on page))

396	[date_firstanalsex3_dh]<br><br>Show the field ONLY if: ([last_anal_male] = '1' or [last_anal_male] = '2' or [last_anal_male] = '3' or [last_anal_male] = '4') and [analsex_partners]>2 and ([alter_relationship3] = '1' or [alter_relationship3] = '2' or [alter_relationship3] = '4') and ([event-name]='baseline_visit_arm_1' or [event-name]='6_month_visit_arm_1' or [event-name]='12_month_visit_arm_1' or [event-name]='18_month_visit_arm_1') AND [acasi_language]='DH'		descriptive<br>(Attachment: DH-Q75.mp3, Display format: Audio file (play in embedded player on page))
397	[date_firstanalsex3]<br><br>Show the field ONLY if: ([last_anal_male] = '1' or [last_anal_male] = '2' or [last_anal_male] = '3' or [last_anal_male] = '4') and [analsex_partners]>2 and ([alter_relationship3] = '1' or [alter_relationship3] = '2' or [alter_relationship3] = '4') and ([event-name]='baseline_visit_arm_1' or [event-name]='6_month_visit_arm_1' or [event-name]='12_month_visit_arm_1' or [event-name]='18_month_visit_arm_1')	When did you have anal sex with this partner for the very first time? If you do not know the exact date, please make your best guess.	text (date_dmy), Required
398	[partner3_ongoing_eng]<br><br>Show the field ONLY if: ([last_anal_male] = '1' or [last_anal_male] = '2' or [last_anal_male] = '3' or [last_anal_male] = '4') and [analsex_partners]>2 and ([alter_relationship3] = '1' or [alter_relationship3] = '2' or [alter_relationship3] = '4') AND ([event-name]='baseline_visit_arm_1' or [event-name]='6_month_visit_arm_1' or [event-name]='12_month_visit_arm_1' or [event-name]='18_month_visit_arm_1') AND [acasi_language]='EN'		descriptive<br>(Attachment: Q76.mp3, Display format: Audio file (play in embedded player on page))

399	[partner3_ongoing_sw]<br><br>Show the field ONLY if: ([last_anal_male] = '1' or [last_anal_male] = '2' or [last_anal_male] = '3' or [last_anal_male] = '4') and [analsex_partners]>2 and ([alter_relationship3] = '1' or [alter_relationship3] = '2' or [alter_relationship3] = '4') AND ([event-name]='baseline_visit_arm_1' or [event-name]='6_month_visit_arm_1' or [event-name]='12_month_visit_arm_1' or [event-name]='18_month_visit_arm_1') AND [acasi_language]='SW'		descriptive<br>(Attachment: SW-Q76.mp3, Display format: Audio file (play in embedded player on page))
400	[partner3_ongoing_dh]<br><br>Show the field ONLY if: ([last_anal_male] = '1' or [last_anal_male] = '2' or [last_anal_male] = '3' or [last_anal_male] = '4') and [analsex_partners]>2 and ([alter_relationship3] = '1' or [alter_relationship3] = '2' or [alter_relationship3] = '4') AND ([event-name]='baseline_visit_arm_1' or [event-name]='6_month_visit_arm_1' or [event-name]='12_month_visit_arm_1' or [event-name]='18_month_visit_arm_1') AND [acasi_language]='DH'		descriptive<br>(Attachment: DH-Q76.mp3, Display format: Audio file (play in embedded player on page))
401	[partner3_ongoing]<br><br>Show the field ONLY if: ([last_anal_male] = '1' or [last_anal_male] = '2' or [last_anal_male] = '3' or [last_anal_male] = '4') and [analsex_partners]>2 and ([alter_relationship3] = '1' or [alter_relationship3] = '2' or [alter_relationship3] = '4') AND ([event-name]='baseline_visit_arm_1' or [event-name]='6_month_visit_arm_1' or [event-name]='12_month_visit_arm_1' or [event-name]='18_month_visit_arm_1')	Do you expect to have anal sex with this partner again?	radio<br>1 No<br>2 Yes<br>3 Don't know
402	[freq_analsex3_eng]		descriptive<br>(Attachment: Q77.mp3, Display format:

	Show the field ONLY if: ([last_anal_male] = '1' or [last_anal_male] = '2' or [last_anal_male] = '3' or [last_anal_male] = '4') and [analsex_partners]>2 and ([alter_relationship3] = '1' or [alter_relationship3] = '2' or [alter_relationship3] = '4') AND [partner3_ongoing]='2' and ([event-name]='baseline_visit_arm_1' or [event-name]='6_month_visit_arm_1' or [event-name]='12_month_visit_arm_1' or [event-name]='18_month_visit_arm_1') AND [acasi_language]='EN'		Audio file (play in embedded player on page))
403	[freq_analsex3_sw]<br><br>Show the field ONLY if: ([last_anal_male] = '1' or [last_anal_male] = '2' or [last_anal_male] = '3' or [last_anal_male] = '4') and [analsex_partners]>2 and ([alter_relationship3] = '1' or [alter_relationship3] = '2' or [alter_relationship3] = '4') AND [partner3_ongoing]='2' and ([event-name]='baseline_visit_arm_1' or [event-name]='6_month_visit_arm_1' or [event-name]='12_month_visit_arm_1' or [event-name]='18_month_visit_arm_1') AND [acasi_language]='SW'		descriptive<br>(Attachment: SW-Q77.mp3, Display format: Audio file (play in embedded player on page))
404	[freq_analsex3_dh]<br><br>Show the field ONLY if: ([last_anal_male] = '1' or [last_anal_male] = '2' or [last_anal_male] = '3' or [last_anal_male] = '4') and [analsex_partners]>2 and ([alter_relationship3] = '1' or [alter_relationship3] = '2' or [alter_relationship3] = '4') AND [partner3_ongoing]='2' and ([event-name]='baseline_visit_arm_1' or [event-name]='6_month_visit_arm_1' or [event-name]='12_month_visit_arm_1' or [event-name]='18_month_visit_arm_1') AND [acasi_language]='DH'		descriptive<br>(Attachment: DH-Q77.mp3, Display format: Audio file (play in embedded player on page))

405	[freq_analsex3]<br><br>Show the field ONLY if: ([last_anal_male] = '1' or [last_anal_male] = '2' or [last_anal_male] = '3' or [last_anal_male] = '4') and [analsex_partners]>2 and ([alter_relationship3] = '1' or [alter_relationship3] = '2' or [alter_relationship3] = '4') AND [partner3_ongoing]='2' and ([event-name]='baseline_visit_arm_1' or [event-name]='6_month_visit_arm_1' or [event-name]='12_month_visit_arm_1' or [event-name]='18_month_visit_arm_1')	How often have you had anal sex with this partner in an average week?	radio, Required<br>1 Daily<br>2 Almost every day<br>3 Once a week<br>4 Less than once a week<br>5 Refuse to answer
406	[condom_analsex3_eng]<br><br>Show the field ONLY if: ([last_anal_male] = '1' or [last_anal_male] = '2' or [last_anal_male] = '3' or [last_anal_male] = '4') and [analsex_partners]>2 and ([alter_relationship3] = '1' or [alter_relationship3] = '2' or [alter_relationship3] = '4') AND ([event-name]='baseline_visit_arm_1' or [event-name]='6_month_visit_arm_1' or [event-name]='12_month_visit_arm_1' or [event-name]='18_month_visit_arm_1') AND [acasi_language]='EN'	Section Header:	descriptive<br>(Attachment: Q78.mp3, Display format: Audio file (play in embedded player on page))
407	[condom_analsex3_sw]<br><br>Show the field ONLY if: ([last_anal_male] = '1' or [last_anal_male] = '2' or [last_anal_male] = '3' or [last_anal_male] = '4') and [analsex_partners]>2 and ([alter_relationship3] = '1' or [alter_relationship3] = '2' or [alter_relationship3] = '4') AND ([event-name]='baseline_visit_arm_1' or [event-name]='6_month_visit_arm_1' or [event-name]='12_month_visit_arm_1' or [event-name]='18_month_visit_arm_1') AND [acasi_language]='SW'		descriptive<br>(Attachment: SW-Q78.mp3, Display format: Audio file (play in embedded player on page))

408	[condom_analsex3_dh]<br><br>Show the field ONLY if: ([last_anal_male] = '1' or [last_anal_male] = '2' or [last_anal_male] = '3' or [last_anal_male] = '4') and [analsex_partners]>2 and ([alter_relationship3] = '1' or [alter_relationship3] = '2' or [alter_relationship3] = '4') AND ([event-name]='baseline_visit_arm_1' or [event-name]='6_month_visit_arm_1' or [event-name]='12_month_visit_arm_1' or [event-name]='18_month_visit_arm_1') AND [acasi_language]='DH'		descriptive<br>(Attachment: DH-Q78.mp3, Display format: Audio file (play in embedded player on page))
409	[condom_analsex3]<br><br>Show the field ONLY if: ([last_anal_male] = '1' or [last_anal_male] = '2' or [last_anal_male] = '3' or [last_anal_male] = '4') and [analsex_partners]>2 and ([alter_relationship3] = '1' or [alter_relationship3] = '2' or [alter_relationship3] = '4') AND ([event-name]='baseline_visit_arm_1' or [event-name]='6_month_visit_arm_1' or [event-name]='12_month_visit_arm_1' or [event-name]='18_month_visit_arm_1')	When you have had anal sex with this partner, how often did you or your partner use a condom?	radio, Required<br>1 Always<br>2 Most of the time<br>3 About half the time<br>4 Rarely<br>5 Never<br>6 Refuse to answer
410	[hiv_analsex3_eng]<br><br>Show the field ONLY if: ([last_anal_male] = '1' or [last_anal_male] = '2' or [last_anal_male] = '3' or [last_anal_male] = '4') and [analsex_partners]>2 and ([alter_relationship3] = '1' or [alter_relationship3] = '2' or [alter_relationship3] = '4') and ([event-name]='baseline_visit_arm_1' or [event-name]='6_month_visit_arm_1' or [event-name]='12_month_visit_arm_1' or [event-name]='18_month_visit_arm_1') AND [acasi_language]='EN'		descriptive<br>(Attachment: Q79.mp3, Display format: Audio file (play in embedded player on page))
411	[hiv_analsex3_sw]		descriptive<br>(Attachment: SW-Q79.mp3, Display format:

	Show the field ONLY if: ([last_anal_male] = '1' or [last_anal_male] = '2' or [last_anal_male] = '3' or [last_anal_male] = '4') and [analsex_partners]>2 and ([alter_relationship3] = '1' or [alter_relationship3] = '2' or [alter_relationship3] = '4') and ([event-name]='baseline_visit_arm_1' or [event-name]='6_month_visit_arm_1' or [event-name]='12_month_visit_arm_1' or [event-name]='18_month_visit_arm_1') AND [acasi_language]='SW'		Audio file (play in embedded player on page))
412	[hiv_analsex3_dh]<br><br>Show the field ONLY if: ([last_anal_male] = '1' or [last_anal_male] = '2' or [last_anal_male] = '3' or [last_anal_male] = '4') and [analsex_partners]>2 and ([alter_relationship3] = '1' or [alter_relationship3] = '2' or [alter_relationship3] = '4') and ([event-name]='baseline_visit_arm_1' or [event-name]='6_month_visit_arm_1' or [event-name]='12_month_visit_arm_1' or [event-name]='18_month_visit_arm_1') AND [acasi_language]='DH'		descriptive<br>(Attachment: DH-Q79.mp3, Display format: Audio file (play in embedded player on page))
413	[hiv_analsex3]<br><br>Show the field ONLY if: ([last_anal_male] = '1' or [last_anal_male] = '2' or [last_anal_male] = '3' or [last_anal_male] = '4') and [analsex_partners]>2 and ([alter_relationship3] = '1' or [alter_relationship3] = '2' or [alter_relationship3] = '4') and ([event-name]='baseline_visit_arm_1' or [event-name]='6_month_visit_arm_1' or [event-name]='12_month_visit_arm_1' or [event-name]='18_month_visit_arm_1')	What do you think this partner's HIV status is?	radio, Required<br>1 HIV positive<br>2 Probably HIV positive<br>3 I have no idea<br>4 Probably HIV negative<br>5 HIV negative<br>6 Refuse to answer
414	[hiv_reason_analsex3_eng]<br><br>Show the field ONLY if:		descriptive<br>(Attachment: Q80.mp3, Display format: Audio file (play in embedded player on page))

	([hiv_analsex3] = '1' or [hiv_analsex3] = '2' or [hiv_analsex3] = '4' or [hiv_analsex3]= '5') and ([event-name]='baseline_visit_arm_1' or [event-name]='6_month_visit_arm_1' or [event-name]='12_month_visit_arm_1' or [event-name]='18_month_visit_arm_1') AND [acasi_language]='EN'		
415	[hiv_reason_analsex3_sw]<br><br>Show the field ONLY if:<br>([hiv_analsex3] = '1' or [hiv_analsex3] = '2' or [hiv_analsex3] = '4' or [hiv_analsex3]= '5') and ([event-name]='baseline_visit_arm_1' or [event-name]='6_month_visit_arm_1' or [event-name]='12_month_visit_arm_1' or [event-name]='18_month_visit_arm_1') AND [acasi_language]='SW'		descriptive<br>(Attachment: SW-Q80.mp3, Display format: Audio file (play in embedded player on page))
416	[hiv_reason_analsex3_dh]<br><br>Show the field ONLY if:<br>([hiv_analsex3] = '1' or [hiv_analsex3] = '2' or [hiv_analsex3] = '4' or [hiv_analsex3]= '5') and ([event-name]='baseline_visit_arm_1' or [event-name]='6_month_visit_arm_1' or [event-name]='12_month_visit_arm_1' or [event-name]='18_month_visit_arm_1') AND [acasi_language]='DH'		descriptive<br>(Attachment: DH-Q80.mp3, Display format: Audio file (play in embedded player on page))
417	[hiv_reason_analsex3]<br><br>Show the field ONLY if:<br>([hiv_analsex3] = '1' or [hiv_analsex3] = '2' or [hiv_analsex3] = '4' or [hiv_analsex3]= '5') and ([event-name]='baseline_visit_arm_1' or [event-name]='6_month_visit_arm_1' or [event-name]='12_month_visit_arm_1' or [event-name]='18_month_visit_arm_1')	Please indicate all the reasons you have for thinking this. (Check all that apply.)	checkbox, Required<br>1 | hiv_reason_analsex3___1 | We discussed our HIV status<br>2 | hiv_reason_analsex3___2 | We did couple counseling together<br>3 | hiv_reason_analsex3___3 | They seemed healthy<br>4 | hiv_reason_analsex3___4 | They seemed unhealthy<br>5 | hiv_reason_analsex3___5 | I heard rumours

			6 | hiv_reason_analsex3___6 | They told me they are taking antiretroviral treatment<br>7 | hiv_reason_analsex3___7 | I have seen them taking pills<br>8 | hiv_reason_analsex3___8 | Don't know<br>9 | hiv_reason_analsex3___9 | Refuse to answer
418	[emotional_ipv_eng]<br><br>Show the field ONLY if:<br>[acasi_language]='EN'	Section Header: *Intimate Partner Violence (enrollment, quarterly)*	descriptive<br>(Attachment: Q81.mp3, Display format: Audio file (play in embedded player on page))
419	[emotional_ipv_sw]<br><br>Show the field ONLY if:<br>[acasi_language]='SW'		descriptive<br>(Attachment: SW-Q81.mp3, Display format: Audio file (play in embedded player on page))
420	[emotional_ipv_dh]<br><br>Show the field ONLY if:<br>[acasi_language]='DH'		descriptive<br>(Attachment: DH-Q81.mp3, Display format: Audio file (play in embedded player on page))
421	[emotional_ipv]	During the past 3 months, have you been in a relationship with a person who threatens, frightens, or insults you, or treats you badly?	radio, Required<br>1 | No<br>2 | Yes<br>3 | Refuse to answer
422	[physical_ipv_eng]<br><br>Show the field ONLY if:<br>[acasi_language]='EN'		descriptive<br>(Attachment: Q82.mp3, Display format: Audio file (play in embedded player on page))
423	[physical_ipv_sw]<br><br>Show the field ONLY if:<br>[acasi_language]='SW'		descriptive<br>(Attachment: SW-Q82.mp3, Display format: Audio file (play in embedded player on page))
424	[physical_ipv_dh]<br><br>Show the field ONLY if:<br>[acasi_language]='DH'		descriptive<br>(Attachment: DH-Q82.mp3, Display format: Audio file (play in embedded player on page))
425	[physical_ipv]	During the past 3 months, have you been in a relationship with a person who has physically hurt you?	radio, Required<br>1 | No<br>2 | Yes<br>3 | Refuse to answer
426	[sexual_ipv_eng]<br><br>Show the field ONLY if:<br>[acasi_language]='EN'		descriptive<br>(Attachment: Q83.mp3, Display format: Audio file (play in embedded player on page))
427	[sexual_ipv_sw]<br><br>Show the field ONLY if:<br>[acasi_language]='SW'		descriptive<br>(Attachment: SW-Q83.mp3, Display format: Audio file (play in embedded player on page))

428	[sexual_ipv_dh]<br><br>Show the field ONLY if:<br>[acasi_language]='DH'		descriptive<br>(Attachment: DH-Q83.mp3, Display format: Audio file (play in embedded player on page))
429	[sexual_ipv]	During the past 3 months, have you been in a relationship with a person who forces you to participate in sexual activities that make you feel uncomfortable?	radio, Required<br>1 No<br>2 Yes<br>3 Refuse to answer
430	[physical_violence_en g]<br><br>Show the field ONLY if:<br>[acasi_language]='EN'	Section Header: *Other Social Harms (enrollment, quarterly)*	descriptive<br>(Attachment: Q84.mp3, Display format: Audio file (play in embedded player on page))
431	[physical_violence_sw]<br><br>Show the field ONLY if:<br>[acasi_language]='SW'		descriptive<br>(Attachment: SW-Q84.mp3, Display format: Audio file (play in embedded player on page))
432	[physical_violence_dh]<br><br>Show the field ONLY if:<br>[acasi_language]='DH'		descriptive<br>(Attachment: DH-Q84.mp3, Display format: Audio file (play in embedded player on page))
433	[physical_violence]	During the past 3 months, have you been physically assaulted (e.g. beaten up, attacked)?	radio, Required<br>1 No<br>2 Yes<br>3 Refuse to answer
434	[sexual_violence_eng]<br><br>Show the field ONLY if:<br>[acasi_language]='EN'		descriptive<br>(Attachment: Q85.mp3, Display format: Audio file (play in embedded player on page))
435	[sexual_violence_sw]<br><br>Show the field ONLY if:<br>[acasi_language]='SW'		descriptive<br>(Attachment: SW-Q85.mp3, Display format: Audio file (play in embedded player on page))
436	[sexual_violence_dh]<br><br>Show the field ONLY if:<br>[acasi_language]='DH'		descriptive<br>(Attachment: DH-Q85.mp3, Display format: Audio file (play in embedded player on page))
437	[sexual_violence]	During the past 3 months, have you been raped or forced to have sex?	radio, Required<br>1 No<br>2 Yes<br>3 Refuse to answer
438	[phq_prompt_eng]<br><br>Show the field ONLY if:<br>([event-name]='baseline_visit_arm_1' or [event-name]='6_month_visit_arm_1' or [event-name]='12_month_visit_arm_1' or [event-name]='18_month_visit_arm_1') AND [acasi_language]='EN'	Section Header: *PHQ-9 (enrollment, every 6 months) The following questions are about your mental health or well-being in the last two weeks.*	descriptive<br>(Attachment: INST-Q86.mp3, Display format: Audio file (play in embedded player on page))

439	[phq_prompt_sw]<br><br>Show the field ONLY if: ([event-name]='baseline_visit_arm_1' or [event-name]='6_month_visit_arm_1' or [event-name]='12_month_visit_arm_1' or [event-name]='18_month_visit_arm_1') AND [acasi_language]='SW'		descriptive<br>(Attachment: SW-INST-Q86.mp3, Display format: Audio file (play in embedded player on page))
440	[phq_prompt_dh]<br><br>Show the field ONLY if: ([event-name]='baseline_visit_arm_1' or [event-name]='6_month_visit_arm_1' or [event-name]='12_month_visit_arm_1' or [event-name]='18_month_visit_arm_1') AND [acasi_language]='DH'		descriptive<br>(Attachment: DH-INST-Q86.mp3, Display format: Audio file (play in embedded player on page))
441	[phq_q1_eng]<br><br>Show the field ONLY if: ([event-name]='baseline_visit_arm_1' or [event-name]='6_month_visit_arm_1' or [event-name]='12_month_visit_arm_1' or [event-name]='18_month_visit_arm_1') AND [acasi_language]='EN'		descriptive<br>(Attachment: Q86.mp3, Display format: Audio file (play in embedded player on page))
442	[phq_q1_sw]<br><br>Show the field ONLY if: ([event-name]='baseline_visit_arm_1' or [event-name]='6_month_visit_arm_1' or [event-name]='12_month_visit_arm_1' or [event-name]='18_month_visit_arm_1') AND [acasi_language]='SW'		descriptive<br>(Attachment: SW-Q86.mp3, Display format: Audio file (play in embedded player on page))
443	[phq_q1_dh]<br><br>Show the field ONLY if: ([event-name]='baseline_visit_arm_1' or [event-name]='6_month_visit_arm_1' or [event-name]='12_month_visit_arm_1' or [event-name]='18_month_visit_arm_1') AND [acasi_language]='DH'		descriptive<br>(Attachment: DH-Q86.mp3, Display format: Audio file (play in embedded player on page))
444	[phq_q1]<br><br>Show the field ONLY if: [event-name]='baseline_visit_arm_1' or [event-name]='6_month_visit_arm	Over the last two weeks, how often have you experienced little interest or pleasure in doing things?	radio, Required<br>1 Not at all<br>2 Several days<br>3 More than half the days

	_1' or [event-name]='12_month_visit_arm_1' or [event-name]='18_month_visit_arm_1'		4 Nearly every day<br>5 Refuse to answer
445	[phq_q2_eng]<br><br>Show the field ONLY if:<br>([event-name]='baseline_visit_arm_1' or [event-name]='6_month_visit_arm_1' or [event-name]='12_month_visit_arm_1' or [event-name]='18_month_visit_arm_1') AND [acasi_language]='EN'		descriptive<br>(Attachment: Q87.mp3, Display format: Audio file (play in embedded player on page))
446	[phq_q2_sw]<br><br>Show the field ONLY if:<br>([event-name]='baseline_visit_arm_1' or [event-name]='6_month_visit_arm_1' or [event-name]='12_month_visit_arm_1' or [event-name]='18_month_visit_arm_1') AND [acasi_language]='SW'		descriptive<br>(Attachment: SW-Q87.mp3, Display format: Audio file (play in embedded player on page))
447	[phq_q2_dh]<br><br>Show the field ONLY if:<br>([event-name]='baseline_visit_arm_1' or [event-name]='6_month_visit_arm_1' or [event-name]='12_month_visit_arm_1' or [event-name]='18_month_visit_arm_1') AND [acasi_language]='DH'		descriptive<br>(Attachment: DH-Q87.mp3, Display format: Audio file (play in embedded player on page))
448	[phq_q2]<br><br>Show the field ONLY if:<br>[event-name]='baseline_visit_arm_1' or [event-name]='6_month_visit_arm_1' or [event-name]='12_month_visit_arm_1' or [event-name]='18_month_visit_arm_1'	Over the last two weeks, how often have you felt down, depressed, or hopeless?	radio, Required<br>1 Not at all<br>2 Several days<br>3 More than half the days<br>4 Nearly every day<br>5 Refuse to answer
449	[phq_q3_eng]<br><br>Show the field ONLY if:<br>([event-name]='baseline_visit_arm_1' or [event-name]='6_month_visit_arm_1' or [event-name]='12_month_visit_arm_1' or [event-name]='18_month_visit_arm_1') AND [acasi_language]='EN'		descriptive<br>(Attachment: Q88.mp3, Display format: Audio file (play in embedded player on page))
450	[phq_q3_sw]<br><br>Show the field ONLY if:		descriptive<br>(Attachment: SW-Q88.mp3, Display format:

	([event-name]='baseline_visit_arm_1' or [event-name]='6_month_visit_arm_1' or [event-name]='12_month_visit_arm_1' or [event-name]='18_month_visit_arm_1') AND [acasi_language]='SW'		Audio file (play in embedded player on page))
451	[phq_q3_dh]<br><br>Show the field ONLY if:<br>([event-name]='baseline_visit_arm_1' or [event-name]='6_month_visit_arm_1' or [event-name]='12_month_visit_arm_1' or [event-name]='18_month_visit_arm_1') AND [acasi_language]='DH'		descriptive<br>(Attachment: DH-Q88.mp3, Display format: Audio file (play in embedded player on page))
452	[phq_q3]<br><br>Show the field ONLY if:<br>[event-name]='baseline_visit_arm_1' or [event-name]='6_month_visit_arm_1' or [event-name]='12_month_visit_arm_1' or [event-name]='18_month_visit_arm_1'	Over the last two weeks, how often have you had trouble falling or staying asleep, or sleeping too much?	radio, Required<br>1 Not at all<br>2 Several days<br>3 More than half the days<br>4 Nearly every day<br>5 Refuse to answer
453	[phq_q4_eng]<br><br>Show the field ONLY if:<br>([event-name]='baseline_visit_arm_1' or [event-name]='6_month_visit_arm_1' or [event-name]='12_month_visit_arm_1' or [event-name]='18_month_visit_arm_1') AND [acasi_language]='EN'	Section Header:	descriptive<br>(Attachment: Q89.mp3, Display format: Audio file (play in embedded player on page))
454	[phq_q4_sw]<br><br>Show the field ONLY if:<br>([event-name]='baseline_visit_arm_1' or [event-name]='6_month_visit_arm_1' or [event-name]='12_month_visit_arm_1' or [event-name]='18_month_visit_arm_1') AND [acasi_language]='SW'		descriptive<br>(Attachment: SW-Q89.mp3, Display format: Audio file (play in embedded player on page))
455	[phq_q4_dh]<br><br>Show the field ONLY if:<br>([event-name]='baseline_visit_arm_1' or [event-name]='6_month_visit_arm_1' or [event-name]='12_month_visit_arm_1' or [event-name]='18_month_		descriptive<br>(Attachment: DH-Q89.mp3, Display format: Audio file (play in embedded player on page))

	visit_arm_1') AND [acasi_language]='DH'		
456	[phq_q4]<br><br>Show the field ONLY if: [event-name]='baseline_visit_arm_1' or [event-name]='6_month_visit_arm_1' or [event-name]='12_month_visit_arm_1' or [event-name]='18_month_visit_arm_1'	Over the last two weeks, how often have you felt tired or had little energy?	radio, Required<br>1 Not at all<br>2 Several days<br>3 More than half the days<br>4 Nearly every day<br>5 Refuse to answer
457	[phq_q5_eng]<br><br>Show the field ONLY if: ([event-name]='baseline_visit_arm_1' or [event-name]='6_month_visit_arm_1' or [event-name]='12_month_visit_arm_1' or [event-name]='18_month_visit_arm_1') AND [acasi_language]='EN'		descriptive<br>(Attachment: Q90.mp3, Display format: Audio file (play in embedded player on page))
458	[phq_q5_sw]<br><br>Show the field ONLY if: ([event-name]='baseline_visit_arm_1' or [event-name]='6_month_visit_arm_1' or [event-name]='12_month_visit_arm_1' or [event-name]='18_month_visit_arm_1') AND [acasi_language]='SW'		descriptive<br>(Attachment: SW-Q90.mp3, Display format: Audio file (play in embedded player on page))
459	[phq_q5_dh]<br><br>Show the field ONLY if: ([event-name]='baseline_visit_arm_1' or [event-name]='6_month_visit_arm_1' or [event-name]='12_month_visit_arm_1' or [event-name]='18_month_visit_arm_1') AND [acasi_language]='DH'		descriptive<br>(Attachment: DH-Q90.mp3, Display format: Audio file (play in embedded player on page))
460	[phq_q5]<br><br>Show the field ONLY if: [event-name]='baseline_visit_arm_1' or [event-name]='6_month_visit_arm_1' or [event-name]='12_month_visit_arm_1' or [event-name]='18_month_visit_arm_1'	Over the last two weeks, how often have you had poor appetite or have overeaten (eaten too much)?	radio, Required<br>1 Not at all<br>2 Several days<br>3 More than half the days<br>4 Nearly every day<br>5 Refuse to answer
461	[phq_q6_eng]<br><br>Show the field ONLY if: ([event-name]='baseline_visit_arm_1' or [event-name]='6_month_visit_arm		descriptive<br>(Attachment: Q91.mp3, Display format: Audio file (play in embedded player on page))

	_1' or [event-name]='12_month_visit_arm_1' or [event-name]='18_month_visit_arm_1') AND [acasi_language]='EN'		
462	[phq_q6_sw]<br><br>Show the field ONLY if:<br>([event-name]='baseline_visit_arm_1' or [event-name]='6_month_visit_arm_1' or [event-name]='12_month_visit_arm_1' or [event-name]='18_month_visit_arm_1') AND [acasi_language]='SW'		descriptive<br>(Attachment: SW-Q91.mp3, Display format: Audio file (play in embedded player on page))
463	[phq_q6_dh]<br><br>Show the field ONLY if:<br>([event-name]='baseline_visit_arm_1' or [event-name]='6_month_visit_arm_1' or [event-name]='12_month_visit_arm_1' or [event-name]='18_month_visit_arm_1') AND [acasi_language]='DH'		descriptive<br>(Attachment: DH-Q91.mp3, Display format: Audio file (play in embedded player on page))
464	[phq_q6]<br><br>Show the field ONLY if:<br>[event-name]='baseline_visit_arm_1' or [event-name]='6_month_visit_arm_1' or [event-name]='12_month_visit_arm_1' or [event-name]='18_month_visit_arm_1'	Over the last two weeks, how often have you felt bad about yourself - or that you are a failure or have let yourself or your family down?	radio, Required<br>1 Not at all<br>2 Several days<br>3 More than half the days<br>4 Nearly every day<br>5 Refuse to answer
465	[phq_q7_eng]<br><br>Show the field ONLY if:<br>([event-name]='baseline_visit_arm_1' or [event-name]='6_month_visit_arm_1' or [event-name]='12_month_visit_arm_1' or [event-name]='18_month_visit_arm_1') AND [acasi_language]='EN'	Section Header:	descriptive<br>(Attachment: Q92.mp3, Display format: Audio file (play in embedded player on page))
466	[phq_q7_sw]<br><br>Show the field ONLY if:<br>([event-name]='baseline_visit_arm_1' or [event-name]='6_month_visit_arm_1' or [event-name]='12_month_visit_arm_1' or [event-name]='18_month_visit_arm_1') AND [acasi_language]='SW'		descriptive<br>(Attachment: SW-Q92.mp3, Display format: Audio file (play in embedded player on page))
467	[phq_q7_dh]		descriptive<br>(Attachment: DH-Q92.mp3, Display format:

	Show the field ONLY if: ([event-name]='baseline_visit_arm_1' or [event-name]='6_month_visit_arm_1' or [event-name]='12_month_visit_arm_1' or [event-name]='18_month_visit_arm_1') AND [acasi_language]='DH'		Audio file (play in embedded player on page))
468	[phq_q7]<br><br>Show the field ONLY if: [event-name]='baseline_visit_arm_1' or [event-name]='6_month_visit_arm_1' or [event-name]='12_month_visit_arm_1' or [event-name]='18_month_visit_arm_1'	Over the last two weeks, how often have you had trouble concentrating on things, such as reading the newspaper or watching television?	radio, Required<br>1 Not at all<br>2 Several days<br>3 More than half the days<br>4 Nearly every day<br>5 Refuse to answer
469	[phq_q8_eng]<br><br>Show the field ONLY if: ([event-name]='baseline_visit_arm_1' or [event-name]='6_month_visit_arm_1' or [event-name]='12_month_visit_arm_1' or [event-name]='18_month_visit_arm_1') AND [acasi_language]='EN'		descriptive<br>(Attachment: Q93.mp3, Display format: Audio file (play in embedded player on page))
470	[phq_q8_sw]<br><br>Show the field ONLY if: ([event-name]='baseline_visit_arm_1' or [event-name]='6_month_visit_arm_1' or [event-name]='12_month_visit_arm_1' or [event-name]='18_month_visit_arm_1') AND [acasi_language]='SW'		descriptive<br>(Attachment: SW-Q93.mp3, Display format: Audio file (play in embedded player on page))
471	[phq_q8_dh]<br><br>Show the field ONLY if: ([event-name]='baseline_visit_arm_1' or [event-name]='6_month_visit_arm_1' or [event-name]='12_month_visit_arm_1' or [event-name]='18_month_visit_arm_1') AND [acasi_language]='DH'		descriptive<br>(Attachment: DH-Q93.mp3, Display format: Audio file (play in embedded player on page))
472	[phq_q8]<br><br>Show the field ONLY if: [event-name]='baseline_visit_arm_1' or [event-name]='6_month_visit_arm_1' or [event-name]='12_month_visit_arm_1' or [e	Over the last two weeks, how often have you experienced moving or speaking so slowly that other people could have noticed? Or the opposite - being so fidgety or restless that you have been moving around a lot more than usual?	radio, Required<br>1 Not at all<br>2 Several days<br>3 More than half the days<br>4 Nearly every day<br>5 Refuse to answer

	vent-name]='18_month_visit_arm_1'		
473	[phq_q9_eng]<br><br>Show the field ONLY if: ([event-name]='baseline_visit_arm_1' or [event-name]='6_month_visit_arm_1' or [event-name]='12_month_visit_arm_1' or [event-name]='18_month_visit_arm_1') AND [acasi_language]='EN'		descriptive<br>(Attachment: Q94.mp3, Display format: Audio file (play in embedded player on page))
474	[phq_q9_sw]<br><br>Show the field ONLY if: ([event-name]='baseline_visit_arm_1' or [event-name]='6_month_visit_arm_1' or [event-name]='12_month_visit_arm_1' or [event-name]='18_month_visit_arm_1') AND [acasi_language]='SW'		descriptive<br>(Attachment: SW-Q94.mp3, Display format: Audio file (play in embedded player on page))
475	[phq_q9_dh]<br><br>Show the field ONLY if: ([event-name]='baseline_visit_arm_1' or [event-name]='6_month_visit_arm_1' or [event-name]='12_month_visit_arm_1' or [event-name]='18_month_visit_arm_1') AND [acasi_language]='DH'		descriptive<br>(Attachment: DH-Q94.mp3, Display format: Audio file (play in embedded player on page))
476	[phq_q9]<br><br>Show the field ONLY if: [event-name]='baseline_visit_arm_1' or [event-name]='6_month_visit_arm_1' or [event-name]='12_month_visit_arm_1' or [event-name]='18_month_visit_arm_1'	Over the last two weeks, how often have you thought that you would be better off dead or of hurting yourself in some way?	radio, Required<br>1 Not at all<br>2 Several days<br>3 More than half the days<br>4 Nearly every day<br>5 Refuse to answer
477	[phq_difficulty_eng]<br><br>Show the field ONLY if: ([phq_q1]>1 or [phq_q2]>1 or [phq_q3]>1 or [phq_q4]>1 or [phq_q5]>1 or [phq_q6]>1 or [phq_q7]>1 or [phq_q8]>1 or [phq_q9]>1) AND ([event-name]='baseline_visit_arm_1' or [event-name]='6_month_visit_arm_1' or [event-name]='12_month_visit_arm_1' or [event-name]='18_month_visit_arm_		descriptive<br>(Attachment: Q95.mp3, Display format: Audio file (play in embedded player on page))

	1') AND [acasi_language] ='EN'		
478	[phq_difficulty_sw]<br><br>Show the field ONLY if: ([phq_q1]>1 or [phq_q2] >1 or [phq_q3]>1 or [phq _q4]>1 or [phq_q5]>1 or [phq_q6]>1 or [phq_q7]> 1 or [phq_q8]>1 or [phq_ q9]>1) AND ([event-nam e]='baseline_visit_arm_1' or [event-name]='6_mon th_visit_arm_1' or [event-name]='12_month_visit_ arm_1' or [event-name] ='18_month_visit_arm_ 1') AND [acasi_language] ='SW'		descriptive<br>(Attachment: SW-Q95.mp3, Display format: Audio file (play in embedded player on page))
479	[phq_difficulty_dh]<br><br>Show the field ONLY if: ([phq_q1]>1 or [phq_q2] >1 or [phq_q3]>1 or [phq _q4]>1 or [phq_q5]>1 or [phq_q6]>1 or [phq_q7]> 1 or [phq_q8]>1 or [phq_ q9]>1) AND ([event-nam e]='baseline_visit_arm_1' or [event-name]='6_mon th_visit_arm_1' or [event-name]='12_month_visit_ arm_1' or [event-name] ='18_month_visit_arm_ 1') AND [acasi_language] ='DH'		descriptive<br>(Attachment: DH-Q95.mp3, Display format: Audio file (play in embedded player on page))
480	[phq_difficulty]<br><br>Show the field ONLY if: ([phq_q1]>1 or [phq_q2] >1 or [phq_q3]>1 or [phq _q4]>1 or [phq_q5]>1 or [phq_q6]>1 or [phq_q7]> 1 or [phq_q8]>1 or [phq_ q9]>1) AND ([event-nam e]='baseline_visit_arm_1' or [event-name]='6_mon th_visit_arm_1' or [event-name]='12_month_visit_ arm_1' or [event-name] ='18_month_visit_arm_ 1')	How difficult have these problems made it for you to do your work, take care of things at home, or get along with other people?	radio, Required<br>1 Not difficult at all<br>2 Somewhat difficult<br>3 Very difficult<br>4 Extremely difficult<br>5 Refuse to answer
481	[audit_prompt_en]<br><br>Show the field ONLY if: [acasi_language]='EN'	Section Header: *AUDIT (enrollment, quarterly) The following questions are about your use of alcoholic beverages during the past year. Because alcohol use can affect many areas of health (and may interfere with certain medications), it is important for us to know how much you usually drink and whether you have experienced any problems with your drinking. Please try to be as honest and as accurate as you can.*	descriptive<br>(Attachment: INST-Q96.mp3, Display format: Audio file (play in embedded player on page))
482	[audit_prompt_sw]<br><br>Show the field ONLY if:		descriptive<br>(Attachment: SW-INST-Q96.mp3, Display

	[acasi_language]='SW'		format: Audio file (play in embedded player on page))
483	[audit_prompt_dh]<br><br>Show the field ONLY if:<br>[acasi_language]='DH'		descriptive<br>(Attachment: DH-INST-Q96.mp3, Display format: Audio file (play in embedded player on page))
484	[audit_q1_eng]<br><br>Show the field ONLY if:<br>[acasi_language]='EN'		descriptive<br>(Attachment: Q96.mp3, Display format: Audio file (play in embedded player on page))
485	[audit_q1_sw]<br><br>Show the field ONLY if:<br>[acasi_language]='SW'		descriptive<br>(Attachment: SW-Q96.mp3, Display format: Audio file (play in embedded player on page))
486	[audit_q1_dh]<br><br>Show the field ONLY if:<br>[acasi_language]='DH'		descriptive<br>(Attachment: DH-Q96.mp3, Display format: Audio file (play in embedded player on page))
487	[audit_q1]	How often do you have a drink containing alcohol?	radio, Required<br>1 Never<br>2 Monthly or less<br>3 2 to 4 times a month<br>4 2 to 3 times per week<br>5 4 or more times a week<br>6 Refuse to answer
488	[audit_q2_eng]<br><br>Show the field ONLY if:<br>[audit_q1]>1 AND [acasi_language]='EN'		descriptive<br>(Attachment: Q97.mp3, Display format: Audio file (play in embedded player on page))
489	[audit_q2_sw]<br><br>Show the field ONLY if:<br>[audit_q1]>1 AND [acasi_language]='SW'		descriptive<br>(Attachment: SW-Q97.mp3, Display format: Audio file (play in embedded player on page))
490	[audit_q2_dh]<br><br>Show the field ONLY if:<br>[audit_q1]>1 AND [acasi_language]='DH'		descriptive<br>(Attachment: DH-Q97.mp3, Display format: Audio file (play in embedded player on page))
491	[audit_q2]<br><br>Show the field ONLY if:<br>[audit_q1]>1	How many drinks containing alcohol do you have on a typical day when you are drinking?	radio, Required<br>1 1 to 2<br>2 3 to 4<br>3 5 to 6<br>4 7 to 9<br>5 10 or more<br>6 Refuse to answer
492	[audit_q3_eng]<br><br>Show the field ONLY if:<br>[audit_q1]>1 AND [acasi_language]='EN'		descriptive<br>(Attachment: Q98.mp3, Display format: Audio file (play in embedded player on page))

493	[audit_q3_sw]<br><br>Show the field ONLY if:<br>[audit_q1]>1 AND [acasi_language]='SW'		descriptive<br>(Attachment: SW-Q98.mp3, Display format: Audio file (play in embedded player on page))
494	[audit_q3_dh]<br><br>Show the field ONLY if:<br>[audit_q1]>1 AND [acasi_language]='DH'		descriptive<br>(Attachment: DH-Q98.mp3, Display format: Audio file (play in embedded player on page))
495	[audit_q3]<br><br>Show the field ONLY if:<br>[audit_q1]>1	How often do you have six or more drinks on one occasion?	radio, Required<br>1 Never<br>2 Less than monthly<br>3 Monthly<br>4 Weekly<br>5 Daily or almost daily<br>6 Refuse to answer
496	[audit_q4_eng]<br><br>Show the field ONLY if:<br>[audit_q1]>1 AND [acasi_language]='EN'	Section Header:	descriptive<br>(Attachment: Q99.mp3, Display format: Audio file (play in embedded player on page))
497	[audit_q4_sw]<br><br>Show the field ONLY if:<br>[audit_q1]>1 AND [acasi_language]='SW'		descriptive<br>(Attachment: SW-Q99.mp3, Display format: Audio file (play in embedded player on page))
498	[audit_q4_dh]<br><br>Show the field ONLY if:<br>[audit_q1]>1 AND [acasi_language]='DH'		descriptive<br>(Attachment: DH-Q99.mp3, Display format: Audio file (play in embedded player on page))
499	[audit_q4]<br><br>Show the field ONLY if:<br>[audit_q1]>1	How often during the last year have you found that you were not able to stop drinking once you had started?	radio, Required<br>1 Never<br>2 A few days a year<br>3 Every month<br>4 Every week<br>5 Every day<br>6 Refuse to answer
500	[audit_q5_eng]<br><br>Show the field ONLY if:<br>[audit_q1]>1 AND [acasi_language]='EN'		descriptive<br>(Attachment: Q100.mp3, Display format: Audio file (play in embedded player on page))
501	[audit_q5_sw]<br><br>Show the field ONLY if:<br>[audit_q1]>1 AND [acasi_language]='SW'		descriptive<br>(Attachment: SW-Q100.mp3, Display format: Audio file (play in embedded player on page))
502	[audit_q5_dh]<br><br>Show the field ONLY if:<br>[audit_q1]>1 AND [acasi_language]='DH'		descriptive<br>(Attachment: DH-Q100.mp3, Display format: Audio file (play in embedded player on page))

503	[audit_q5]<br><br>Show the field ONLY if:<br>[audit_q1]>1	How often during the last year have you failed to do what was normally expected from you because of drinking?	radio, Required<br>1 Never<br>2 A few days a year<br>3 Every month<br>4 Every week<br>5 Every day<br>6 Refuse to answer
504	[audit_q6_eng]<br><br>Show the field ONLY if:<br>[audit_q1]>1 AND [acasi_language]='EN'		descriptive<br>(Attachment: Q101.mp3, Display format: Audio file (play in embedded player on page))
505	[audit_q6_sw]<br><br>Show the field ONLY if:<br>[audit_q1]>1 AND [acasi_language]='SW'		descriptive<br>(Attachment: SW-Q101.mp3, Display format: Audio file (play in embedded player on page))
506	[audit_q6_dh]<br><br>Show the field ONLY if:<br>[audit_q1]>1 AND [acasi_language]='DH'		descriptive<br>(Attachment: DH-Q101.mp3, Display format: Audio file (play in embedded player on page))
507	[audit_q6]<br><br>Show the field ONLY if:<br>[audit_q1]>1	How often during the last year have you needed a first drink in the morning to get yourself going after a heavy drinking session?	radio, Required<br>1 Never<br>2 A few days a year<br>3 Every month<br>4 Every week<br>5 Every day<br>6 Refuse to answer
508	[audit_q7_eng]<br><br>Show the field ONLY if:<br>[audit_q1]>1 AND [acasi_language]='EN'	Section Header:	descriptive<br>(Attachment: Q102.mp3, Display format: Audio file (play in embedded player on page))
509	[audit_q7_sw]<br><br>Show the field ONLY if:<br>[audit_q1]>1 AND [acasi_language]='SW'		descriptive<br>(Attachment: SW-Q102.mp3, Display format: Audio file (play in embedded player on page))
510	[audit_q7_dh]<br><br>Show the field ONLY if:<br>[audit_q1]>1 AND [acasi_language]='DH'		descriptive<br>(Attachment: DH-Q102.mp3, Display format: Audio file (play in embedded player on page))

511	[audit_q7]<br><br>Show the field ONLY if:<br>[audit_q1]>1	How often during the last year have you had a feeling of guilt or remorse after drinking?	radio, Required<br>1 Never<br>2 A few days a year<br>3 Every month<br>4 Every week<br>5 Every day<br>6 Refuse to answer
512	[audit_q8_eng]<br><br>Show the field ONLY if:<br>[audit_q1]>1 AND [acasi_language]='EN'		descriptive<br>(Attachment: Q103.mp3, Display format: Audio file (play in embedded player on page))
513	[audit_q8_sw]<br><br>Show the field ONLY if:<br>[audit_q1]>1 AND [acasi_language]='SW'		descriptive<br>(Attachment: SW-Q103.mp3, Display format: Audio file (play in embedded player on page))
514	[audit_q8_dh]<br><br>Show the field ONLY if:<br>[audit_q1]>1 AND [acasi_language]='DH'		descriptive<br>(Attachment: DH-Q103.mp3, Display format: Audio file (play in embedded player on page))
515	[audit_q8]<br><br>Show the field ONLY if:<br>[audit_q1]>1	How often during the last year have you been unable to remember what happened the night before because you had been drinking?	radio, Required<br>1 Never<br>2 A few days a year<br>3 Every month<br>4 Every week<br>5 Every day<br>6 Refuse to answer
516	[audit_q9_eng]<br><br>Show the field ONLY if:<br>[acasi_language]='EN'		descriptive<br>(Attachment: Q104.mp3, Display format: Audio file (play in embedded player on page))
517	[audit_q9_sw]<br><br>Show the field ONLY if:<br>[acasi_language]='SW'		descriptive<br>(Attachment: SW-Q104.mp3, Display format: Audio file (play in embedded player on page))
518	[audit_q9_dh]<br><br>Show the field ONLY if:<br>[acasi_language]='DH'		descriptive<br>(Attachment: DH-Q104.mp3, Display format: Audio file (play in embedded player on page))
519	[audit_q9]	Have you or someone else ever been injured as a result of your drinking, either in the past or more recently?	radio, Required<br>1 No<br>2 Yes but not in the last year<br>3 Yes during the last year<br>4 Refuse to answer
520	[audit_q10_eng]<br><br>Show the field ONLY if:<br>[acasi_language]='EN'		descriptive<br>(Attachment: Q105.mp3, Display format: Audio file (play in embedded player on page))

521	[audit_q10_sw]<br><br>Show the field ONLY if:<br>[acasi_language]='SW'		descriptive<br>(Attachment: SW-Q105.mp3, Display format: Audio file (play in embedded player on page))
522	[audit_q10_dh]<br><br>Show the field ONLY if:<br>[acasi_language]='DH'		descriptive<br>(Attachment: DH-Q105.mp3, Display format: Audio file (play in embedded player on page))
523	[audit_q10]	Has a relative or friend, or a doctor or other health worker ever been concerned about your drinking or suggested you cut down, either in the past or more recently?	radio, Required<br>1 No<br>2 Yes but not in the last year<br>3 Yes during the last year<br>4 Refuse to answer
524	[dast_prompt_eng]<br><br>Show the field ONLY if:<br>([event-name]='baseline_visit_arm_1' or [event-name]='6_month_visit_arm_1' or [event-name]='12_month_visit_arm_1' or [event-name]='18_month_visit_arm_1') AND [acasi_language]='EN'	Section Header: *DAST (enrollment, every 6 months) The following questions concern information about your potential involvement with drugs excluding alcohol and tobacco during the past 6 months. Some of the drugs we are asking about are miraa (khat), bhang (marijuana), hashish, inhalants (glue, paint), heroin, cocaine, pain medications, sleeping medications, and others. Remember that the questions do not include alcohol or tobacco.*	descriptive<br>(Attachment: INST-Q106.mp3, Display format: Audio file (play in embedded player on page))
525	[dast_prompt_sw]<br><br>Show the field ONLY if:<br>([event-name]='baseline_visit_arm_1' or [event-name]='6_month_visit_arm_1' or [event-name]='12_month_visit_arm_1' or [event-name]='18_month_visit_arm_1') AND [acasi_language]='SW'		descriptive<br>(Attachment: SW-INST-Q106.mp3, Display format: Audio file (play in embedded player on page))
526	[dast_prompt_dh]<br><br>Show the field ONLY if:<br>([event-name]='baseline_visit_arm_1' or [event-name]='6_month_visit_arm_1' or [event-name]='12_month_visit_arm_1' or [event-name]='18_month_visit_arm_1') AND [acasi_language]='DH'		descriptive<br>(Attachment: DH-INST-Q106.mp3, Display format: Audio file (play in embedded player on page))
527	[dast_q1_eng]<br><br>Show the field ONLY if:<br>([event-name]='baseline_visit_arm_1' or [event-name]='6_month_visit_arm_1' or [event-name]='12_month_visit_arm_1' or [event-name]='18_month_visit_arm_1') AND [acasi_language]='EN'		descriptive<br>(Attachment: Q106.mp3, Display format: Audio file (play in embedded player on page))

528	[dast_q1_sw]<br><br>Show the field ONLY if:<br>([event-name]='baseline_visit_arm_1' or [event-name]='6_month_visit_arm_1' or [event-name]='12_month_visit_arm_1' or [event-name]='18_month_visit_arm_1') AND [acasi_language]='SW'		descriptive<br>(Attachment: SW-Q106.mp3, Display format: Audio file (play in embedded player on page))
529	[dast_q1_dh]<br><br>Show the field ONLY if:<br>([event-name]='baseline_visit_arm_1' or [event-name]='6_month_visit_arm_1' or [event-name]='12_month_visit_arm_1' or [event-name]='18_month_visit_arm_1') AND [acasi_language]='DH'		descriptive<br>(Attachment: DH-Q106.mp3, Display format: Audio file (play in embedded player on page))
530	[dast_q1]<br><br>Show the field ONLY if:<br>[event-name]='baseline_visit_arm_1' or [event-name]='6_month_visit_arm_1' or [event-name]='12_month_visit_arm_1' or [event-name]='18_month_visit_arm_1'	In the past 6 months, have you used drugs other than those required for medical reasons?	radio, Required<br>1 No<br>2 Yes<br>3 Refuse to answer
531	[dast_q2_eng]<br><br>Show the field ONLY if:<br>[dast_q1] = '2' AND ([event-name]='baseline_visit_arm_1' or [event-name]='6_month_visit_arm_1' or [event-name]='12_month_visit_arm_1' or [event-name]='18_month_visit_arm_1') AND [acasi_language]='EN'		descriptive<br>(Attachment: Q107.mp3, Display format: Audio file (play in embedded player on page))
532	[dast_q2_sw]<br><br>Show the field ONLY if:<br>[dast_q1] = '2' AND ([event-name]='baseline_visit_arm_1' or [event-name]='6_month_visit_arm_1' or [event-name]='12_month_visit_arm_1' or [event-name]='18_month_visit_arm_1') AND [acasi_language]='SW'		descriptive<br>(Attachment: SW-Q107.mp3, Display format: Audio file (play in embedded player on page))
533	[dast_q2_dh]<br><br>Show the field ONLY if:<br>[dast_q1] = '2' AND ([event-name]='baseline_visit		descriptive<br>(Attachment: DH-Q107.mp3, Display format: Audio file (play in embedded player on page))

	_arm_1' or [event-name]='6_month_visit_arm_1' or [event-name]='12_month_visit_arm_1' or [event-name]='18_month_visit_arm_1') AND [acasi_language]='DH'		
534	[dast_q2]<br><br>Show the field ONLY if:<br>[dast_q1] = '2' AND ([event-name]='baseline_visit_arm_1' or [event-name]='6_month_visit_arm_1' or [event-name]='12_month_visit_arm_1' or [event-name]='18_month_visit_arm_1')	In the past 6 months, what type or types of drug have you used that are not medicines? Check all that apply.	checkbox, Required<br>4 | dast_q2___4 | Miraa (khat)<br>5 | dast_q2___5 | Bhang (marijuana)<br>6 | dast_q2___6 | Hashish<br>7 | dast_q2___7 | Inhalants (glue, paint)<br>8 | dast_q2___8 | Heroin<br>9 | dast_q2___9 | Cocaine<br>10 | dast_q2___10 | Pain medications<br>11 | dast_q2___11 | Sleeping medications<br>12 | dast_q2___12 | Other<br>13 | dast_q2___13 | Not applicable<br>14 | dast_q2___14 | Refuse to answer
535	[dast_q3]<br><br>Show the field ONLY if:<br>[dast_q2(12)] = '1'	Other substance (specify)	text, Required
536	[dast_q4_eng]<br><br>Show the field ONLY if:<br>[dast_q1] = '2' AND ([event-name]='baseline_visit_arm_1' or [event-name]='6_month_visit_arm_1' or [event-name]='12_month_visit_arm_1' or [event-name]='18_month_visit_arm_1') AND [acasi_language]='EN'		descriptive<br>(Attachment: Q108.mp3, Display format: Audio file (play in embedded player on page))
537	[dast_q4_sw]<br><br>Show the field ONLY if:<br>[dast_q1] = '2' AND ([event-name]='baseline_visit_arm_1' or [event-name]='6_month_visit_arm_1' or [event-name]='12_month_visit_arm_1' or [event-name]='18_month_visit_arm_1') AND [acasi_language]='SW'		descriptive<br>(Attachment: SW-Q108.mp3, Display format: Audio file (play in embedded player on page))
538	[dast_q4_dh]<br><br>Show the field ONLY if:<br>[dast_q1] = '2' AND ([event-name]='baseline_visit_arm_1' or [event-name]='6_month_visit_arm_1' or [event-name]='12_mo		descriptive<br>(Attachment: DH-Q108.mp3, Display format: Audio file (play in embedded player on page))

	nth_visit_arm_1' or [event-name]='18_month_visit_arm_1') AND [acasi_language]='DH'		
539	[dast_q4]<br><br>Show the field ONLY if: [dast_q1] = '2' AND ([event-name]='baseline_visit_arm_1' or [event-name]='6_month_visit_arm_1' or [event-name]='12_month_visit_arm_1' or [event-name]='18_month_visit_arm_1')	In the past 6 months, have you taken more than one drug at a time?	radio, Required<br>1 No<br>2 Yes<br>3 Refuse to answer
540	[dast_q5_eng]<br><br>Show the field ONLY if: [dast_q1] = '2' AND ([event-name]='baseline_visit_arm_1' or [event-name]='6_month_visit_arm_1' or [event-name]='12_month_visit_arm_1' or [event-name]='18_month_visit_arm_1') AND [acasi_language]='EN'	Section Header:	descriptive<br>(Attachment: Q109.mp3, Display format: Audio file (play in embedded player on page))
541	[dast_q5_sw]<br><br>Show the field ONLY if: [dast_q1] = '2' AND ([event-name]='baseline_visit_arm_1' or [event-name]='6_month_visit_arm_1' or [event-name]='12_month_visit_arm_1' or [event-name]='18_month_visit_arm_1') AND [acasi_language]='SW'		descriptive<br>(Attachment: SW-Q109.mp3, Display format: Audio file (play in embedded player on page))
542	[dast_q5_dh]<br><br>Show the field ONLY if: [dast_q1] = '2' AND ([event-name]='baseline_visit_arm_1' or [event-name]='6_month_visit_arm_1' or [event-name]='12_month_visit_arm_1' or [event-name]='18_month_visit_arm_1') AND [acasi_language]='DH'		descriptive<br>(Attachment: DH-Q109.mp3, Display format: Audio file (play in embedded player on page))
543	[dast_q5]<br><br>Show the field ONLY if: [dast_q1] = '2' AND ([event-name]='baseline_visit_arm_1' or [event-name]='6_month_visit_arm_1' or [event-name]='12_month_visit_arm_1' or [even	In the past 6 months, have you always been able to stop using drugs when you want to?	radio, Required<br>1 No<br>2 Yes<br>3 Refuse to answer

	t-name]='18_month_visit_arm_1')		
544	[dast_q6_eng]<br><br>Show the field ONLY if:<br>[dast_q1] = '2' AND ([event-name]='baseline_visit_arm_1' or [event-name]='6_month_visit_arm_1' or [event-name]='12_month_visit_arm_1' or [event-name]='18_month_visit_arm_1') AND [acasi_language]='EN'		descriptive<br>(Attachment: Q110.mp3, Display format: Audio file (play in embedded player on page))
545	[dast_q6_sw]<br><br>Show the field ONLY if:<br>[dast_q1] = '2' AND ([event-name]='baseline_visit_arm_1' or [event-name]='6_month_visit_arm_1' or [event-name]='12_month_visit_arm_1' or [event-name]='18_month_visit_arm_1') AND [acasi_language]='SW'		descriptive<br>(Attachment: SW-Q110.mp3, Display format: Audio file (play in embedded player on page))
546	[dast_q6_dh]<br><br>Show the field ONLY if:<br>[dast_q1] = '2' AND ([event-name]='baseline_visit_arm_1' or [event-name]='6_month_visit_arm_1' or [event-name]='12_month_visit_arm_1' or [event-name]='18_month_visit_arm_1') AND [acasi_language]='DH'		descriptive<br>(Attachment: DH-Q110.mp3, Display format: Audio file (play in embedded player on page))
547	[dast_q6]<br><br>Show the field ONLY if:<br>[dast_q1] = '2' AND ([event-name]='baseline_visit_arm_1' or [event-name]='6_month_visit_arm_1' or [event-name]='12_month_visit_arm_1' or [event-name]='18_month_visit_arm_1')	In the past 6 months, have you had "blackouts" or "flashbacks" as a result of drug use?	radio, Required<br>1 No<br>2 Yes<br>3 Refuse to answer
548	[dast_q7_eng]<br><br>Show the field ONLY if:<br>[dast_q1] = '2' AND ([event-name]='baseline_visit_arm_1' or [event-name]='6_month_visit_arm_1' or [event-name]='12_month_visit_arm_1' or [event-name]='18_month_visit_arm_1') AND [acasi_language]='EN'		descriptive<br>(Attachment: Q111.mp3, Display format: Audio file (play in embedded player on page))

549	[dast_q7_sw]<br><br>Show the field ONLY if:<br>[dast_q1] = '2' AND ([event-name]='baseline_visit_arm_1' or [event-name]='6_month_visit_arm_1' or [event-name]='12_month_visit_arm_1' or [event-name]='18_month_visit_arm_1') AND [acasi_language]='SW'		descriptive<br>(Attachment: SW-Q111.mp3, Display format: Audio file (play in embedded player on page))
550	[dast_q7_dh]<br><br>Show the field ONLY if:<br>[dast_q1] = '2' AND ([event-name]='baseline_visit_arm_1' or [event-name]='6_month_visit_arm_1' or [event-name]='12_month_visit_arm_1' or [event-name]='18_month_visit_arm_1') AND [acasi_language]='DH'		descriptive<br>(Attachment: DH-Q111.mp3, Display format: Audio file (play in embedded player on page))
551	[dast_q7]<br><br>Show the field ONLY if:<br>[dast_q1] = '2' AND ([event-name]='baseline_visit_arm_1' or [event-name]='6_month_visit_arm_1' or [event-name]='12_month_visit_arm_1' or [event-name]='18_month_visit_arm_1')	In the past 6 months, have you ever felt bad or guilty about your drug use?	radio, Required<br>1 No<br>2 Yes<br>3 Refuse to answer
552	[dast_q8_eng]<br><br>Show the field ONLY if:<br>[dast_q1] = '2' AND ([event-name]='baseline_visit_arm_1' or [event-name]='6_month_visit_arm_1' or [event-name]='12_month_visit_arm_1' or [event-name]='18_month_visit_arm_1') AND [acasi_language]='EN'	Section Header:	descriptive<br>(Attachment: Q112.mp3, Display format: Audio file (play in embedded player on page))
553	[dast_q8_sw]<br><br>Show the field ONLY if:<br>[dast_q1] = '2' AND ([event-name]='baseline_visit_arm_1' or [event-name]='6_month_visit_arm_1' or [event-name]='12_month_visit_arm_1' or [event-name]='18_month_visit_arm_1') AND [acasi_language]='SW'		descriptive<br>(Attachment: SW-Q112.mp3, Display format: Audio file (play in embedded player on page))
554	[dast_q8_dh]		descriptive<br>(Attachment: DH-Q112.mp3, Display format:

	Show the field ONLY if: [dast_q1] = '2' AND ([event-name]='baseline_visit_arm_1' or [event-name]='6_month_visit_arm_1' or [event-name]='12_month_visit_arm_1' or [event-name]='18_month_visit_arm_1') AND [acasi_language]='DH'		Audio file (play in embedded player on page))
555	[dast_q8]<br><br>Show the field ONLY if: [dast_q1] = '2' AND ([event-name]='baseline_visit_arm_1' or [event-name]='6_month_visit_arm_1' or [event-name]='12_month_visit_arm_1' or [event-name]='18_month_visit_arm_1')	In the past 6 months, has your spouse (or parent) ever complained about your involvement with drugs?	radio, Required<br>1 No<br>2 Yes<br>3 Refuse to answer
556	[dast_q9_eng]<br><br>Show the field ONLY if: [dast_q1] = '2' AND ([event-name]='baseline_visit_arm_1' or [event-name]='6_month_visit_arm_1' or [event-name]='12_month_visit_arm_1' or [event-name]='18_month_visit_arm_1') AND [acasi_language]='EN'		descriptive<br>(Attachment: Q113.mp3, Display format: Audio file (play in embedded player on page))
557	[dast_q9_sw]<br><br>Show the field ONLY if: [dast_q1] = '2' AND ([event-name]='baseline_visit_arm_1' or [event-name]='6_month_visit_arm_1' or [event-name]='12_month_visit_arm_1' or [event-name]='18_month_visit_arm_1') AND [acasi_language]='SW'		descriptive<br>(Attachment: SW-Q113.mp3, Display format: Audio file (play in embedded player on page))
558	[dast_q9_dh]<br><br>Show the field ONLY if: [dast_q1] = '2' AND ([event-name]='baseline_visit_arm_1' or [event-name]='6_month_visit_arm_1' or [event-name]='12_month_visit_arm_1' or [event-name]='18_month_visit_arm_1') AND [acasi_language]='DH'		descriptive<br>(Attachment: DH-Q113.mp3, Display format: Audio file (play in embedded player on page))
559	[dast_q9]<br><br>Show the field ONLY if:	In the past 6 months, have you neglected your family because of your drug use?	radio, Required<br>1 No<br>2 Yes

	[dast_q1] = '2' AND ([event-name]='baseline_visit_arm_1' or [event-name]='6_month_visit_arm_1' or [event-name]='12_month_visit_arm_1' or [event-name]='18_month_visit_arm_1')		3 Refuse to answer
560	[dast_q10_eng]<br><br>Show the field ONLY if:<br>[dast_q1] = '2' AND ([event-name]='baseline_visit_arm_1' or [event-name]='6_month_visit_arm_1' or [event-name]='12_month_visit_arm_1' or [event-name]='18_month_visit_arm_1') AND [acasi_language]='EN'		descriptive<br>(Attachment: Q114.mp3, Display format: Audio file (play in embedded player on page))
561	[dast_q10_sw]<br><br>Show the field ONLY if:<br>[dast_q1] = '2' AND ([event-name]='baseline_visit_arm_1' or [event-name]='6_month_visit_arm_1' or [event-name]='12_month_visit_arm_1' or [event-name]='18_month_visit_arm_1') AND [acasi_language]='SW'		descriptive<br>(Attachment: SW-Q114.mp3, Display format: Audio file (play in embedded player on page))
562	[dast_q10_dh]<br><br>Show the field ONLY if:<br>[dast_q1] = '2' AND ([event-name]='baseline_visit_arm_1' or [event-name]='6_month_visit_arm_1' or [event-name]='12_month_visit_arm_1' or [event-name]='18_month_visit_arm_1') AND [acasi_language]='DH'		descriptive<br>(Attachment: DH-Q114.mp3, Display format: Audio file (play in embedded player on page))
563	[dast_q10]<br><br>Show the field ONLY if:<br>[dast_q1] = '2' AND ([event-name]='baseline_visit_arm_1' or [event-name]='6_month_visit_arm_1' or [event-name]='12_month_visit_arm_1' or [event-name]='18_month_visit_arm_1')	In the past 6 months, have you engaged in illegal activities in order to obtain drugs?	radio, Required<br>1 No<br>2 Yes<br>3 Refuse to answer
564	[dast_q11_eng]<br><br>Show the field ONLY if:<br>[dast_q1] = '2' AND ([event-name]='baseline_visit_arm_1' or [event-name]	Section Header:	descriptive<br>(Attachment: Q115.mp3, Display format: Audio file (play in embedded player on page))

	='6_month_visit_arm_1' or [event-name]='12_month_visit_arm_1' or [event-name]='18_month_visit_arm_1') and [acasi_language]='EN'		
565	[dast_q11_sw]<br><br>Show the field ONLY if: [dast_q1] = '2' AND ([event-name]='baseline_visit_arm_1' or [event-name]='6_month_visit_arm_1' or [event-name]='12_month_visit_arm_1' or [event-name]='18_month_visit_arm_1') and [acasi_language]='SW'		descriptive<br>(Attachment: SW-Q115.mp3, Display format: Audio file (play in embedded player on page))
566	[dast_q11_dh]<br><br>Show the field ONLY if: [dast_q1] = '2' AND ([event-name]='baseline_visit_arm_1' or [event-name]='6_month_visit_arm_1' or [event-name]='12_month_visit_arm_1' or [event-name]='18_month_visit_arm_1') and [acasi_language]='DH'		descriptive<br>(Attachment: DH-Q115.mp3, Display format: Audio file (play in embedded player on page))
567	[dast_q11]<br><br>Show the field ONLY if: [dast_q1] = '2' AND ([event-name]='baseline_visit_arm_1' or [event-name]='6_month_visit_arm_1' or [event-name]='12_month_visit_arm_1' or [event-name]='18_month_visit_arm_1')	In the past 6 months, have you ever experienced withdrawal symptoms (felt sick) when you stopped taking drugs?	radio, Required<br>1 No<br>2 Yes<br>3 Refuse to answer
568	[dast_q12_eng]<br><br>Show the field ONLY if: [dast_q1] = '2' AND ([event-name]='baseline_visit_arm_1' or [event-name]='6_month_visit_arm_1' or [event-name]='12_month_visit_arm_1' or [event-name]='18_month_visit_arm_1') AND [acasi_language]='EN'		descriptive<br>(Attachment: Q116.mp3, Display format: Audio file (play in embedded player on page))
569	[dast_q12_sw]<br><br>Show the field ONLY if: [dast_q1] = '2' AND ([event-name]='baseline_visit_arm_1' or [event-name]='6_month_visit_arm_1' or [event-name]='12_mo		descriptive<br>(Attachment: SW-Q116.mp3, Display format: Audio file (play in embedded player on page))

	nth_visit_arm_1' or [event-name]='18_month_visit_arm_1') AND [acasi_language]='SW'		
570	[dast_q12_dh]<br><br>Show the field ONLY if:<br>[dast_q1] = '2' AND ([event-name]='baseline_visit_arm_1' or [event-name]='6_month_visit_arm_1' or [event-name]='12_month_visit_arm_1' or [event-name]='18_month_visit_arm_1') AND [acasi_language]='DH'		descriptive<br>(Attachment: DH-Q116.mp3, Display format: Audio file (play in embedded player on page))
571	[dast_q12]<br><br>Show the field ONLY if:<br>[dast_q1] = '2' AND ([event-name]='baseline_visit_arm_1' or [event-name]='6_month_visit_arm_1' or [event-name]='12_month_visit_arm_1' or [event-name]='18_month_visit_arm_1')	In the past 6 months, have you had medical problems as a result of your drug use (memory loss, hepatitis, convulsions, bleeding, etc)?	radio, Required<br>1 No<br>2 Yes<br>3 Refuse to answer
572	[dast_q13_eng]<br><br>Show the field ONLY if:<br>[dast_q1] = '2' AND ([event-name]='baseline_visit_arm_1' or [event-name]='6_month_visit_arm_1' or [event-name]='12_month_visit_arm_1' or [event-name]='18_month_visit_arm_1') AND [acasi_language]='EN'		descriptive<br>(Attachment: Q117.mp3, Display format: Audio file (play in embedded player on page))
573	[dast_q13_sw]<br><br>Show the field ONLY if:<br>[dast_q1] = '2' AND ([event-name]='baseline_visit_arm_1' or [event-name]='6_month_visit_arm_1' or [event-name]='12_month_visit_arm_1' or [event-name]='18_month_visit_arm_1') AND [acasi_language]='SW'		descriptive<br>(Attachment: Q117 K.mp3, Display format: Audio file (play in embedded player on page))
574	[dast_q13_dh]<br><br>Show the field ONLY if:<br>[dast_q1] = '2' AND ([event-name]='baseline_visit_arm_1' or [event-name]='6_month_visit_arm_1' or [event-name]='12_month_visit_arm_1' or [event-name]='18_month_visit		descriptive<br>(Attachment: Q117 L.mp3, Display format: Audio file (play in embedded player on page))

	_arm_1') AND [acasi_language]='DH'		
575	[dast_q13]<br><br>Show the field ONLY if: [dast_q1] = '2' AND ([event-name]='baseline_visit_arm_1' or [event-name]='6_month_visit_arm_1' or [event-name]='12_month_visit_arm_1' or [event-name]='18_month_visit_arm_1')	Some people have tried injecting drugs using a syringe or needle. Have you injected drugs in the last 6 months?	radio, Required<br>1 No<br>2 Yes<br>8 Don't know<br>9 No response
576	[dast_q14_eng]<br><br>Show the field ONLY if: [dast_q1] = '2' AND ([event-name]='baseline_visit_arm_1' or [event-name]='6_month_visit_arm_1' or [event-name]='12_month_visit_arm_1' or [event-name]='18_month_visit_arm_1') AND [acasi_language]='EN'		descriptive<br>(Attachment: Q118.mp3, Display format: Audio file (play in embedded player on page))
577	[dast_q14_sw]<br><br>Show the field ONLY if: [dast_q1] = '2' AND ([event-name]='baseline_visit_arm_1' or [event-name]='6_month_visit_arm_1' or [event-name]='12_month_visit_arm_1' or [event-name]='18_month_visit_arm_1') AND [acasi_language]='SW'		descriptive<br>(Attachment: SW-Q118.mp3, Display format: Audio file (play in embedded player on page))
578	[dast_q14_dh]<br><br>Show the field ONLY if: [dast_q1] = '2' AND ([event-name]='baseline_visit_arm_1' or [event-name]='6_month_visit_arm_1' or [event-name]='12_month_visit_arm_1' or [event-name]='18_month_visit_arm_1') AND [acasi_language]='DH'		descriptive<br>(Attachment: DH-Q118.mp3, Display format: Audio file (play in embedded player on page))
579	[dast_14]<br><br>Show the field ONLY if: [dast_q1] = '2' AND ([event-name]='baseline_visit_arm_1' or [event-name]='6_month_visit_arm_1' or [event-name]='12_month_visit_arm_1' or [event-name]='18_month_visit_arm_1')	Have you shared a syringe or needle with anyone else when injecting drugs in the last 6 months?	radio, Required<br>1 No<br>2 Yes<br>8 Don't know<br>9 No response

580	[child_abuse_prompt_eng]<br><br>Show the field ONLY if:<br>[event-name]='baseline_visit_arm_1' AND [acasi_language]='EN'	Section Header: *Childhood Abuse (enrollment only) Children are often the target of violence, mistreatment, or abuse, many times at the hands of parents, family friends, teachers, or other adults in positions of power. The following questions will ask about any violence or mistreatment you may have experienced during your childhood.*	descriptive<br>(Attachment: INST-Q119.mp3, Display format: Audio file (play in embedded player on page))
581	[child_abuse_prompt_sw]<br><br>Show the field ONLY if:<br>[event-name]='baseline_visit_arm_1' AND [acasi_language]='SW'		descriptive<br>(Attachment: SW-INST-Q119.mp3, Display format: Audio file (play in embedded player on page))
582	[child_abuse_prompt_dh]<br><br>Show the field ONLY if:<br>[event-name]='baseline_visit_arm_1' AND [acasi_language]='DH'		descriptive<br>(Attachment: DH-INST-Q119.mp3, Display format: Audio file (play in embedded player on page))
583	[ceca_q1_eng]<br><br>Show the field ONLY if:<br>[event-name]='baseline_visit_arm_1' AND [acasi_language]='EN'		descriptive<br>(Attachment: Q119.mp3, Display format: Audio file (play in embedded player on page))
584	[ceca_q1_sw]<br><br>Show the field ONLY if:<br>[event-name]='baseline_visit_arm_1' AND [acasi_language]='SW'		descriptive<br>(Attachment: SW-Q119.mp3, Display format: Audio file (play in embedded player on page))
585	[ceca_q1_dh]<br><br>Show the field ONLY if:<br>[event-name]='baseline_visit_arm_1' AND [acasi_language]='DH'		descriptive<br>(Attachment: DH-Q119.mp3, Display format: Audio file (play in embedded player on page))
586	[ceca_q1]<br><br>Show the field ONLY if:<br>[event-name]='baseline_visit_arm_1'	When you were a child or teenager were you ever hit repeatedly with something (such as a belt or stick) or punched, kicked, or burnt by someone in the household?	radio, Required<br>1 No<br>2 Yes<br>3 Refuse to answer
587	[ceca_q2_eng]<br><br>Show the field ONLY if:<br>[event-name]='baseline_visit_arm_1' AND [acasi_language]='EN'		descriptive<br>(Attachment: Q120.mp3, Display format: Audio file (play in embedded player on page))
588	[ceca_q2_sw]<br><br>Show the field ONLY if:<br>[event-name]='baseline_visit_arm_1' AND [acasi_language]='SW'		descriptive<br>(Attachment: SW-Q120.mp3, Display format: Audio file (play in embedded player on page))

589	[ceca_q2_dh]<br><br>Show the field ONLY if:<br>[event-name]='baseline_visit_arm_1' AND [acasi_language]='DH'		descriptive<br>(Attachment: DH-Q120.mp3, Display format: Audio file (play in embedded player on page))
590	[ceca_q2]<br><br>Show the field ONLY if:<br>[event-name]='baseline_visit_arm_1'	When you were a child or teenager did you ever have any unwanted sexual experiences?	radio, Required<br>1 No<br>2 Yes<br>3 Refuse to answer
591	[ceca_q3_eng]<br><br>Show the field ONLY if:<br>[event-name]='baseline_visit_arm_1' AND [acasi_language]='EN'		descriptive<br>(Attachment: Q121.mp3, Display format: Audio file (play in embedded player on page))
592	[ceca_q3_sw]<br><br>Show the field ONLY if:<br>[event-name]='baseline_visit_arm_1' AND [acasi_language]='SW'		descriptive<br>(Attachment: SW-Q121.mp3, Display format: Audio file (play in embedded player on page))
593	[ceca_q3_dh]<br><br>Show the field ONLY if:<br>[event-name]='baseline_visit_arm_1' AND [acasi_language]='DH'		descriptive<br>(Attachment: DH-Q121.mp3, Display format: Audio file (play in embedded player on page))
594	[ceca_q3]<br><br>Show the field ONLY if:<br>[event-name]='baseline_visit_arm_1'	Did anyone force you or persuade you to have sexual intercourse against your wishes before the age of 17?	radio, Required<br>1 No<br>2 Yes<br>3 Refuse to answer
595	[ceca_q4_eng]<br><br>Show the field ONLY if:<br>[event-name]='baseline_visit_arm_1' AND [acasi_language]='EN'		descriptive<br>(Attachment: Q122.mp3, Display format: Audio file (play in embedded player on page))
596	[ceca_q4_sw]<br><br>Show the field ONLY if:<br>[event-name]='baseline_visit_arm_1' AND [acasi_language]='SW'		descriptive<br>(Attachment: SW-Q122.mp3, Display format: Audio file (play in embedded player on page))
597	[ceca_q4_dh]<br><br>Show the field ONLY if:<br>[event-name]='baseline_visit_arm_1' AND [acasi_language]='DH'		descriptive<br>(Attachment: DH-Q122.mp3, Display format: Audio file (play in embedded player on page))
598	[ceca_q4]<br><br>Show the field ONLY if:<br>[event-name]='baseline_visit_arm_1'	Can you think of any upsetting sexual experiences before age 17 with a related adult or someone in authority (e.g. a teacher)?	radio, Required<br>1 No<br>2 Yes<br>3 Refuse to answer

599	[inst_stigma_eng]<br><br>Show the field ONLY if: ([event-name]='baseline_visit_arm_1' or [event-name]='6_month_visit_arm_1' or [event-name]='12_month_visit_arm_1' or [event-name]='18_month_visit_arm_1') AND [acasi_language]='EN'	Section Header: *Sexual Stigma (enrollment half-yearly) It is challenging to be a man who has sex with men in Kenya. We would like to ask a few questions about your experiences.*	descriptive<br>(Attachment: INST-123 E.mp3, Display format: Audio file (play in embedded player on page))
600	[inst_stigma_sw]<br><br>Show the field ONLY if: ([event-name]='baseline_visit_arm_1' or [event-name]='6_month_visit_arm_1' or [event-name]='12_month_visit_arm_1' or [event-name]='18_month_visit_arm_1') AND [acasi_language]='SW'		descriptive<br>(Attachment: INST-123 K.mp3, Display format: Audio file (play in embedded player on page))
601	[inst_stigma_dh]<br><br>Show the field ONLY if: ([event-name]='baseline_visit_arm_1' or [event-name]='6_month_visit_arm_1' or [event-name]='12_month_visit_arm_1' or [event-name]='18_month_visit_arm_1') AND [acasi_language]='DH'		descriptive<br>(Attachment: INST-123 L.mp3, Display format: Audio file (play in embedded player on page))
602	[stigma_q1_eng]<br><br>Show the field ONLY if: ([event-name]='baseline_visit_arm_1' or [event-name]='6_month_visit_arm_1' or [event-name]='12_month_visit_arm_1' or [event-name]='18_month_visit_arm_1') AND [acasi_language]='EN'		descriptive<br>(Attachment: Q123.mp3, Display format: Audio file (play in embedded player on page))
603	[stigma_q1_sw]<br><br>Show the field ONLY if: ([event-name]='baseline_visit_arm_1' or [event-name]='6_month_visit_arm_1' or [event-name]='12_month_visit_arm_1' or [event-name]='18_month_visit_arm_1') AND [acasi_language]='SW'		descriptive<br>(Attachment: SW-Q123.mp3, Display format: Audio file (play in embedded player on page))
604	[stigma_q1_dh]<br><br>Show the field ONLY if: ([event-name]='baseline_visit_arm_1' or [event-name]='6_month_visit_arm		descriptive<br>(Attachment: DH-Q123.mp3, Display format: Audio file (play in embedded player on page))

	_1' or [event-name]='12_month_visit_arm_1' or [event-name]='18_month_visit_arm_1') AND [acasi_language]='DH'		
605	[stigma_q1]<br><br>Show the field ONLY if:<br>[event-name]='baseline_visit_arm_1' or [event-name]='6_month_visit_arm_1' or [event-name]='12_month_visit_arm_1' or [event-name]='18_month_visit_arm_1'	How often have you heard that homosexuals are not normal?	radio, Required<br>1 Never<br>2 Once or twice<br>3 A few times<br>4 Many times<br>5 Refuse to answer
606	[sitgma_q2_eng]<br><br>Show the field ONLY if:<br>([event-name]='baseline_visit_arm_1' or [event-name]='6_month_visit_arm_1' or [event-name]='12_month_visit_arm_1' or [event-name]='18_month_visit_arm_1') AND [acasi_language]='EN'		descriptive<br>(Attachment: Q124.mp3, Display format: Audio file (play in embedded player on page))
607	[stigma_q2_sw]<br><br>Show the field ONLY if:<br>([event-name]='baseline_visit_arm_1' or [event-name]='6_month_visit_arm_1' or [event-name]='12_month_visit_arm_1' or [event-name]='18_month_visit_arm_1') AND [acasi_language]='SW'		descriptive<br>(Attachment: SW-Q124.mp3, Display format: Audio file (play in embedded player on page))
608	[stigma_q2_dh]<br><br>Show the field ONLY if:<br>([event-name]='baseline_visit_arm_1' or [event-name]='6_month_visit_arm_1' or [event-name]='12_month_visit_arm_1' or [event-name]='18_month_visit_arm_1') AND [acasi_language]='DH'		descriptive<br>(Attachment: DH-Q124.mp3, Display format: Audio file (play in embedded player on page))
609	[stigma_q2]<br><br>Show the field ONLY if:<br>[event-name]='baseline_visit_arm_1' or [event-name]='6_month_visit_arm_1' or [event-name]='12_month_visit_arm_1' or [event-name]='18_month_visit_arm_1'	How often have you felt that you hurt or embarrassed your family because you have sex with men?	radio, Required<br>1 Never<br>2 Once or twice<br>3 A few times<br>4 Many times<br>5 Refuse to answer
610	[sitgma_q3_eng]<br><br>Show the field ONLY if:		descriptive<br>(Attachment: Q125.mp3, Display format:

	([event-name]='baseline_visit_arm_1' or [event-name]='6_month_visit_arm_1' or [event-name]='12_month_visit_arm_1' or [event-name]='18_month_visit_arm_1') AND [acasi_language]='EN'		Audio file (play in embedded player on page))
611	[stigma_q3_sw]<br><br>Show the field ONLY if:<br>([event-name]='baseline_visit_arm_1' or [event-name]='6_month_visit_arm_1' or [event-name]='12_month_visit_arm_1' or [event-name]='18_month_visit_arm_1') AND [acasi_language]='SW'		descriptive<br>(Attachment: SW-Q125.mp3, Display format: Audio file (play in embedded player on page))
612	[stigma_q3_dh]<br><br>Show the field ONLY if:<br>([event-name]='baseline_visit_arm_1' or [event-name]='6_month_visit_arm_1' or [event-name]='12_month_visit_arm_1' or [event-name]='18_month_visit_arm_1') AND [acasi_language]='DH'		descriptive<br>(Attachment: DH-Q125.mp3, Display format: Audio file (play in embedded player on page))
613	[stigma_q3]<br><br>Show the field ONLY if:<br>[event-name]='baseline_visit_arm_1' or [event-name]='6_month_visit_arm_1' or [event-name]='12_month_visit_arm_1' or [event-name]='18_month_visit_arm_1'	How often have you been made fun of or called names because you have sex with men?	radio, Required<br>1 Never<br>2 Once or twice<br>3 A few times<br>4 Many times<br>5 Refuse to answer
614	[stigma_q4_eng]<br><br>Show the field ONLY if:<br>([event-name]='baseline_visit_arm_1' or [event-name]='6_month_visit_arm_1' or [event-name]='12_month_visit_arm_1' or [event-name]='18_month_visit_arm_1') AND [acasi_language]='EN'	Section Header:	descriptive<br>(Attachment: Q126.mp3, Display format: Audio file (play in embedded player on page))
615	[stigma_q4_sw]<br><br>Show the field ONLY if:<br>([event-name]='baseline_visit_arm_1' or [event-name]='6_month_visit_arm_1' or [event-name]='12_month_visit_arm_1' or [event-name]='18_month_		descriptive<br>(Attachment: SW-Q126.mp3, Display format: Audio file (play in embedded player on page))

	visit_arm_1') AND [acasi_language]='SW'		
616	[stigma_q4_dh]<br><br>Show the field ONLY if:<br>([event-name]='baseline_visit_arm_1' or [event-name]='6_month_visit_arm_1' or [event-name]='12_month_visit_arm_1' or [event-name]='18_month_visit_arm_1') AND [acasi_language]='DH'		descriptive<br>(Attachment: DH-Q126.mp3, Display format: Audio file (play in embedded player on page))
617	[stigma_q4]<br><br>Show the field ONLY if:<br>[event-name]='baseline_visit_arm_1' or [event-name]='6_month_visit_arm_1' or [event-name]='12_month_visit_arm_1' or [event-name]='18_month_visit_arm_1'	How often have you been hit or beaten up because you have sex with men?	radio, Required<br>1 Never<br>2 Once or twice<br>3 A few times<br>4 Many times<br>5 Refuse to answer
618	[stigma_q5_eng]<br><br>Show the field ONLY if:<br>([event-name]='baseline_visit_arm_1' or [event-name]='6_month_visit_arm_1' or [event-name]='12_month_visit_arm_1' or [event-name]='18_month_visit_arm_1') AND [acasi_language]='EN'		descriptive<br>(Attachment: Q127.mp3, Display format: Audio file (play in embedded player on page))
619	[stigma_q5_sw]<br><br>Show the field ONLY if:<br>([event-name]='baseline_visit_arm_1' or [event-name]='6_month_visit_arm_1' or [event-name]='12_month_visit_arm_1' or [event-name]='18_month_visit_arm_1') AND [acasi_language]='SW'		descriptive<br>(Attachment: SW-Q127.mp3, Display format: Audio file (play in embedded player on page))
620	[stigma_q5_dh]<br><br>Show the field ONLY if:<br>([event-name]='baseline_visit_arm_1' or [event-name]='6_month_visit_arm_1' or [event-name]='12_month_visit_arm_1' or [event-name]='18_month_visit_arm_1') AND [acasi_language]='DH'		descriptive<br>(Attachment: DH-Q127.mp3, Display format: Audio file (play in embedded player on page))
621	[stigma_q5]<br><br>Show the field ONLY if:<br>[event-name]='baseline_visit_arm_1' or [event-na	How often have you had to pretend that you are not a homosexual in order to be accepted?	radio, Required<br>1 Never<br>2 Once or twice

	me]='6_month_visit_arm_1' or [event-name]='12_month_visit_arm_1' or [event-name]='18_month_visit_arm_1'		3 A few times<br>4 Many times<br>5 Refuse to answer
622	[stigma_q6_eng]<br><br>Show the field ONLY if:<br>([event-name]='baseline_visit_arm_1' or [event-name]='6_month_visit_arm_1' or [event-name]='12_month_visit_arm_1' or [event-name]='18_month_visit_arm_1') AND [acasi_language]='EN'		descriptive<br>(Attachment: Q128.mp3, Display format: Audio file (play in embedded player on page))
623	[stigma_q6_sw]<br><br>Show the field ONLY if:<br>([event-name]='baseline_visit_arm_1' or [event-name]='6_month_visit_arm_1' or [event-name]='12_month_visit_arm_1' or [event-name]='18_month_visit_arm_1') AND [acasi_language]='SW'		descriptive<br>(Attachment: SW-Q128.mp3, Display format: Audio file (play in embedded player on page))
624	[stigma_q6_dh]<br><br>Show the field ONLY if:<br>([event-name]='baseline_visit_arm_1' or [event-name]='6_month_visit_arm_1' or [event-name]='12_month_visit_arm_1' or [event-name]='18_month_visit_arm_1') AND [acasi_language]='DH'		descriptive<br>(Attachment: DH-Q128.mp3, Display format: Audio file (play in embedded player on page))
625	[stigma_q6]<br><br>Show the field ONLY if:<br>[event-name]='baseline_visit_arm_1' or [event-name]='6_month_visit_arm_1' or [event-name]='12_month_visit_arm_1' or [event-name]='18_month_visit_arm_1'	How often has your family not accepted you because you have sex with men?	radio, Required<br>1 Never<br>2 Once or twice<br>3 A few times<br>4 Many times<br>5 Refuse to answer
626	[stigma_q7_eng]<br><br>Show the field ONLY if:<br>([event-name]='baseline_visit_arm_1' or [event-name]='6_month_visit_arm_1' or [event-name]='12_month_visit_arm_1' or [event-name]='18_month_visit_arm_1') AND [acasi_language]='EN'	Section Header:	descriptive<br>(Attachment: Q129.mp3, Display format: Audio file (play in embedded player on page))
627	[stigma_q7_sw]		descriptive<br>(Attachment: SW-Q129.mp3, Display format:

	Show the field ONLY if: ([event-name]='baseline_visit_arm_1' or [event-name]='6_month_visit_arm_1' or [event-name]='12_month_visit_arm_1' or [event-name]='18_month_visit_arm_1') AND [acasi_language]='SW'		Audio file (play in embedded player on page))
628	[stigma_q7_dh]<br><br>Show the field ONLY if: ([event-name]='baseline_visit_arm_1' or [event-name]='6_month_visit_arm_1' or [event-name]='12_month_visit_arm_1' or [event-name]='18_month_visit_arm_1') AND [acasi_language]='DH'		descriptive<br>(Attachment: DH-Q129.mp3, Display format: Audio file (play in embedded player on page))
629	[stigma_q7]<br><br>Show the field ONLY if: [event-name]='baseline_visit_arm_1' or [event-name]='6_month_visit_arm_1' or [event-name]='12_month_visit_arm_1' or [event-name]='18_month_visit_arm_1'	How often have you lost your friends because you have sex with men?	radio, Required<br>1 Never<br>2 Once or twice<br>3 A few times<br>4 Many times<br>5 Refuse to answer
630	[stigma_q8_eng]<br><br>Show the field ONLY if: ([event-name]='baseline_visit_arm_1' or [event-name]='6_month_visit_arm_1' or [event-name]='12_month_visit_arm_1' or [event-name]='18_month_visit_arm_1') AND [acasi_language]='EN'		descriptive<br>(Attachment: Q130.mp3, Display format: Audio file (play in embedded player on page))
631	[stigma_q8_sw]<br><br>Show the field ONLY if: ([event-name]='baseline_visit_arm_1' or [event-name]='6_month_visit_arm_1' or [event-name]='12_month_visit_arm_1' or [event-name]='18_month_visit_arm_1') AND [acasi_language]='SW'		descriptive<br>(Attachment: SW-Q130.mp3, Display format: Audio file (play in embedded player on page))
632	[stigma_q8_dh]<br><br>Show the field ONLY if: ([event-name]='baseline_visit_arm_1' or [event-name]='6_month_visit_arm_1' or [event-name]='12_month_visit_arm_1' or [event-name]='18_month_		descriptive<br>(Attachment: DH-Q130.mp3, Display format: Audio file (play in embedded player on page))

	visit_arm_1') AND [acasi_language]='DH'		
633	[stigma_q8]<br><br>Show the field ONLY if:<br>[event-name]='baseline_visit_arm_1' or [event-name]='6_month_visit_arm_1' or [event-name]='12_month_visit_arm_1' or [event-name]='18_month_visit_arm_1'	How often have you been kicked out of school because you have sex with men?	radio, Required<br>1 Never<br>2 Once or twice<br>3 A few times<br>4 Many times<br>5 Refuse to answer
634	[stigma_q9_eng]<br><br>Show the field ONLY if:<br>([event-name]='baseline_visit_arm_1' or [event-name]='6_month_visit_arm_1' or [event-name]='12_month_visit_arm_1' or [event-name]='18_month_visit_arm_1') AND [acasi_language]='EN'		descriptive<br>(Attachment: Q131.mp3, Display format: Audio file (play in embedded player on page))
635	[stigma_9_sw]<br><br>Show the field ONLY if:<br>([event-name]='baseline_visit_arm_1' or [event-name]='6_month_visit_arm_1' or [event-name]='12_month_visit_arm_1' or [event-name]='18_month_visit_arm_1') AND [acasi_language]='SW'		descriptive<br>(Attachment: SW-Q131.mp3, Display format: Audio file (play in embedded player on page))
636	[stigma_9_dh]<br><br>Show the field ONLY if:<br>([event-name]='baseline_visit_arm_1' or [event-name]='6_month_visit_arm_1' or [event-name]='12_month_visit_arm_1' or [event-name]='18_month_visit_arm_1') AND [acasi_language]='DH'		descriptive<br>(Attachment: DH-Q131.mp3, Display format: Audio file (play in embedded player on page))
637	[stigma_q9]<br><br>Show the field ONLY if:<br>[event-name]='baseline_visit_arm_1' or [event-name]='6_month_visit_arm_1' or [event-name]='12_month_visit_arm_1' or [event-name]='18_month_visit_arm_1'	How often have you lost a place to live because you have sex with men?	radio, Required<br>1 Never<br>2 Once or twice<br>3 A few times<br>4 Many times<br>5 Refuse to answer
638	[stigma_q10_eng]<br><br>Show the field ONLY if:<br>([event-name]='baseline_visit_arm_1' or [event-name]='6_month_visit_arm	Section Header:	descriptive<br>(Attachment: Q132.mp3, Display format: Audio file (play in embedded player on page))

	_1' or [event-name]='12_month_visit_arm_1' or [event-name]='18_month_visit_arm_1') AND [acasi_language]='EN'		
639	[stigma_q10_sw]<br><br>Show the field ONLY if:<br>([event-name]='baseline_visit_arm_1' or [event-name]='6_month_visit_arm_1' or [event-name]='12_month_visit_arm_1' or [event-name]='18_month_visit_arm_1') AND [acasi_language]='SW'		descriptive<br>(Attachment: SW-Q132.mp3, Display format: Audio file (play in embedded player on page))
640	[stigma_q10_dh]<br><br>Show the field ONLY if:<br>([event-name]='baseline_visit_arm_1' or [event-name]='6_month_visit_arm_1' or [event-name]='12_month_visit_arm_1' or [event-name]='18_month_visit_arm_1') AND [acasi_language]='DH'		descriptive<br>(Attachment: DH-Q132.mp3, Display format: Audio file (play in embedded player on page))
641	[stigma_q10]<br><br>Show the field ONLY if:<br>[event-name]='baseline_visit_arm_1' or [event-name]='6_month_visit_arm_1' or [event-name]='12_month_visit_arm_1' or [event-name]='18_month_visit_arm_1'	How often have you lost a job or career opportunity because you have sex with men?	radio, Required<br>1 Never<br>2 Once or twice<br>3 A few times<br>4 Many times<br>5 Refuse to answer
642	[stigma_q11_eng]<br><br>Show the field ONLY if:<br>([event-name]='baseline_visit_arm_1' or [event-name]='6_month_visit_arm_1' or [event-name]='12_month_visit_arm_1' or [event-name]='18_month_visit_arm_1') AND [acasi_language]='EN'		descriptive<br>(Attachment: Q133.mp3, Display format: Audio file (play in embedded player on page))
643	[stigma_q11_sw]<br><br>Show the field ONLY if:<br>([event-name]='baseline_visit_arm_1' or [event-name]='6_month_visit_arm_1' or [event-name]='12_month_visit_arm_1' or [event-name]='18_month_visit_arm_1') AND [acasi_language]='SW'		descriptive<br>(Attachment: SW-Q133.mp3, Display format: Audio file (play in embedded player on page))
644	[stigma_q11_dh]		descriptive<br>(Attachment: DH-Q133.mp3, Display format:

	Show the field ONLY if: ([event-name]='baseline_visit_arm_1' or [event-name]='6_month_visit_arm_1' or [event-name]='12_month_visit_arm_1' or [event-name]='18_month_visit_arm_1') AND [acasi_language]='DH'		Audio file (play in embedded player on page))
645	[stigma_q11]<br><br>Show the field ONLY if: [event-name]='baseline_visit_arm_1' or [event-name]='6_month_visit_arm_1' or [event-name]='12_month_visit_arm_1' or [event-name]='18_month_visit_arm_1'	How often have you experienced police harassment because you have sex with men?	radio, Required<br>1 Never<br>2 Once or twice<br>3 A few times<br>4 Many times<br>5 Refuse to answer
646	[incarceration_eng]<br><br>Show the field ONLY if: ([event-name]='baseline_visit_arm_1' or [event-name]='6_month_visit_arm_1' or [event-name]='12_month_visit_arm_1' or [event-name]='18_month_visit_arm_1') AND [acasi_language]='EN'	Section Header: *Social Harms (every 6 months)*	descriptive<br>(Attachment: Q134.mp3, Display format: Audio file (play in embedded player on page))
647	[incarceration_sw]<br><br>Show the field ONLY if: ([event-name]='baseline_visit_arm_1' or [event-name]='6_month_visit_arm_1' or [event-name]='12_month_visit_arm_1' or [event-name]='18_month_visit_arm_1') AND [acasi_language]='SW'		descriptive<br>(Attachment: SW-Q134.mp3, Display format: Audio file (play in embedded player on page))
648	[incarceration_dh]<br><br>Show the field ONLY if: ([event-name]='baseline_visit_arm_1' or [event-name]='6_month_visit_arm_1' or [event-name]='12_month_visit_arm_1' or [event-name]='18_month_visit_arm_1') AND [acasi_language]='DH'		descriptive<br>(Attachment: DH-Q134.mp3, Display format: Audio file (play in embedded player on page))
649	[incarceration]<br><br>Show the field ONLY if: [event-name]='baseline_visit_arm_1' or [event-name]='6_month_visit_arm_1' or [event-name]='12_month_visit_arm_1' or [e	In the past 3 months, have you been incarcerated (that is, spent one or more nights in jail, a detection center, or prison) for any reason?	radio, Required<br>1 No<br>2 Yes<br>3 Refuse to answer

	vent-name]='18_month_visit_arm_1'		
650	[risk_perception_eng]<br><br>Show the field ONLY if:<br>[acasi_language]='EN'	Section Header: *STI Risk Perception (enrollment, quarterly)*	descriptive<br>(Attachment: Q135.mp3, Display format: Audio file (play in embedded player on page))
651	[risk_perception_sw]<br><br>Show the field ONLY if:<br>[acasi_language]='SW'		descriptive<br>(Attachment: SW-Q135.mp3, Display format: Audio file (play in embedded player on page))
652	[risk_perception_dh]<br><br>Show the field ONLY if:<br>[acasi_language]='DH'		descriptive<br>(Attachment: DH-Q135.mp3, Display format: Audio file (play in embedded player on page))
653	[risk_perception]	What do you think are your chances of getting a sexually transmitted infection?	radio, Required<br>1 No chance at all<br>2 Small chance<br>3 Moderate chance<br>4 Great chance<br>5 Don't know<br>6 Refuse to answer
654	[ever_doxypep_eng]<br><br>Show the field ONLY if:<br>[event-name]='baseline_visit_arm_1' AND [acasi_language]='EN'		descriptive<br>(Attachment: Q136.mp3, Display format: Audio file (play in embedded player on page))
655	[ever_doxypep_sw]<br><br>Show the field ONLY if:<br>[event-name]='baseline_visit_arm_1' AND [acasi_language]='SW'		descriptive<br>(Attachment: Q136 K.mp3, Display format: Audio file (play in embedded player on page))
656	[ever_doxypep_dh]<br><br>Show the field ONLY if:<br>[event-name]='baseline_visit_arm_1' AND [acasi_language]='DH'		descriptive<br>(Attachment: Q136 L.mp3, Display format: Audio file (play in embedded player on page))
657	[ever_doxypep]<br><br>Show the field ONLY if:<br>[event-name]='baseline_visit_arm_1'	Before enrolling in this study, had you ever taken doxyPEP (i.e., taking two tablets of doxycycline within 24-72 hours after unprotected anal sex)?	radio<br>1 No<br>2 Yes<br>3 Don't know<br>4 No response
658	[ever_doxypep_times_eng]<br><br>Show the field ONLY if:<br>[event-name]='baseline_visit_arm_1' and [acasi_language]='EN' and [ever_doxypep]=2		descriptive<br>(Attachment: Q137.mp3, Display format: Audio file (play in embedded player on page))

659	[ever_doxypep_times_sw]<br><br>Show the field ONLY if:<br>[event-name]='baseline_visit_arm_1' and [acasi_language]='SW' and [ever_doxypep]=2		descriptive<br>(Attachment: SW-Q137.mp3, Display format: Audio file (play in embedded player on page))
660	[ever_doxypep_times_dh]<br><br>Show the field ONLY if:<br>[event-name]='baseline_visit_arm_1' and [acasi_language]='DH' and [ever_doxypep]=2		descriptive<br>(Attachment: DH-Q137.mp3, Display format: Audio file (play in embedded player on page))
661	[ever_doxypep_times]<br><br>Show the field ONLY if:<br>[event-name]='baseline_visit_arm_1' and [ever_doxypep]=2	How many times have you taken doxyPEP in the last 3 months?	text
662	[aim_prompt_eng]<br><br>Show the field ONLY if:<br>([event-name]='baseline_visit_arm_1' or [event-name]='6_month_visit_arm_1' or [event-name]='12_month_visit_arm_1' or [event-name]='18_month_visit_arm_1') and [acasi_language]='EN'	Section Header: *Intervention Acceptability and Feasibility (every 6 months) For the questions below, please consider your assignment in the Mambo Matatu trial and whether you agree or disagree with the statements below.*	descriptive<br>(Attachment: INST-Q138.mp3, Display format: Audio file (play in embedded player on page))
663	[aim_prompt_sw]<br><br>Show the field ONLY if:<br>([event-name]='baseline_visit_arm_1' or [event-name]='6_month_visit_arm_1' or [event-name]='12_month_visit_arm_1' or [event-name]='18_month_visit_arm_1') AND [acasi_language]='SW'		descriptive<br>(Attachment: SW-INST-Q138.mp3, Display format: Audio file (play in embedded player on page))
664	[aim_prompt_dh]<br><br>Show the field ONLY if:<br>([event-name]='baseline_visit_arm_1' or [event-name]='6_month_visit_arm_1' or [event-name]='12_month_visit_arm_1' or [event-name]='18_month_visit_arm_1') AND [acasi_language]='DH'		descriptive<br>(Attachment: DH-INST-Q138.mp3, Display format: Audio file (play in embedded player on page))
665	[aim1_st_eng]<br><br>Show the field ONLY if:<br>([event-name]='baseline_visit_arm_1' or [event-name]='6_month_visit_arm		descriptive<br>(Attachment: Q138.3E.mp3, Display format: Audio file (play in embedded player on page))

	_1' or [event-name]='12_month_visit_arm_1' or [event-name]='18_month_visit_arm_1') AND [acasi_language]='EN' AND [randomization_arm_1][randomization_group]='2'		
666	[aim1_st_sw]<br><br>Show the field ONLY if:<br>([event-name]='baseline_visit_arm_1' or [event-name]='6_month_visit_arm_1' or [event-name]='12_month_visit_arm_1' or [event-name]='18_month_visit_arm_1') AND [acasi_language]='SW' AND [randomization_arm_1][randomization_group]='2'		descriptive<br>(Attachment: SW-Q138.3.mp3, Display format: Audio file (play in embedded player on page))
667	[aim1_st_dh]<br><br>Show the field ONLY if:<br>([event-name]='baseline_visit_arm_1' or [event-name]='6_month_visit_arm_1' or [event-name]='12_month_visit_arm_1' or [event-name]='18_month_visit_arm_1') AND [acasi_language]='DH' AND [randomization_arm_1][randomization_group]='2'		descriptive<br>(Attachment: DH-Q138.3L.mp3, Display format: Audio file (play in embedded player on page))
668	[aim1_ppt_eng]<br><br>Show the field ONLY if:<br>([event-name]='baseline_visit_arm_1' or [event-name]='6_month_visit_arm_1' or [event-name]='12_month_visit_arm_1' or [event-name]='18_month_visit_arm_1') AND [acasi_language]='EN' AND [randomization_arm_1][randomization_group]='0'		descriptive<br>(Attachment: Q138.PPT.E.mp3, Display format: Audio file (play in embedded player on page))
669	[aim1_ppt_sw]<br><br>Show the field ONLY if:<br>([event-name]='baseline_visit_arm_1' or [event-name]='6_month_visit_arm_1' or [event-name]='12_month_visit_arm_1' or [event-name]='18_month_visit_arm_1') AND [acasi_language]='SW' AND [randomization_arm_1][randomization_group]='0'		descriptive<br>(Attachment: Q138.PPT.K.mp3, Display format: Audio file (play in embedded player on page))
670	[aim1_ppt_dh]		descriptive<br>(Attachment: Q138.PPT.L.mp3, Display

	Show the field ONLY if: ([event-name]='baseline_visit_arm_1' or [event-name]='6_month_visit_arm_1' or [event-name]='12_month_visit_arm_1' or [event-name]='18_month_visit_arm_1') AND [acasi_language]='DH' AND [randomization_arm_1][randomization_group]='0'		format: Audio file (play in embedded player on page))
671	[aim1_doxypep_eng]<br><br>Show the field ONLY if: ([event-name]='baseline_visit_arm_1' or [event-name]='6_month_visit_arm_1' or [event-name]='12_month_visit_arm_1' or [event-name]='18_month_visit_arm_1') AND [acasi_language]='EN' AND [randomization_arm_1][randomization_group]='1'		descriptive<br>(Attachment: Q138.1E.mp3, Display format: Audio file (play in embedded player on page))
672	[aim1_doxypep_sw]<br><br>Show the field ONLY if: ([event-name]='baseline_visit_arm_1' or [event-name]='6_month_visit_arm_1' or [event-name]='12_month_visit_arm_1' or [event-name]='18_month_visit_arm_1') AND [acasi_language]='SW' AND [randomization_arm_1][randomization_group]='1'		descriptive<br>(Attachment: SW-Q138.1.mp3, Display format: Audio file (play in embedded player on page))
673	[aim1_doxypep_dh]<br><br>Show the field ONLY if: ([event-name]='baseline_visit_arm_1' or [event-name]='6_month_visit_arm_1' or [event-name]='12_month_visit_arm_1' or [event-name]='18_month_visit_arm_1') AND [acasi_language]='DH' AND [randomization_arm_1][randomization_group]='1'		descriptive<br>(Attachment: DH-Q138.1L.mp3, Display format: Audio file (play in embedded player on page))
674	[aim1]<br><br>Show the field ONLY if: [event-name]='baseline_visit_arm_1' or [event-name]='6_month_visit_arm_1' or [event-name]='12_month_visit_arm_1' or [event-name]='18_month_visit_arm_1'	[randomization_arm_1][randomization_group] meets my approval.	radio, Required<br>1 Completely disagree<br>2 Disagree<br>3 Neither agree nor disagree<br>4 Agree<br>5 Completely agree<br>6 Refuse to answer

675	[aim2_doxypep_eng]<br><br>Show the field ONLY if: ([event-name]='baseline_visit_arm_1' or [event-name]='6_month_visit_arm_1' or [event-name]='12_month_visit_arm_1' or [event-name]='18_month_visit_arm_1') AND [acasi_language]='EN' AND [randomization_arm_1][randomization_group]='1'		descriptive<br>(Attachment: Q139.1E.mp3, Display format: Audio file (play in embedded player on page))
676	[aim2_doxypep_sw]<br><br>Show the field ONLY if: ([event-name]='baseline_visit_arm_1' or [event-name]='6_month_visit_arm_1' or [event-name]='12_month_visit_arm_1' or [event-name]='18_month_visit_arm_1') AND [acasi_language]='SW' AND [randomization_arm_1][randomization_group]='1'		descriptive<br>(Attachment: SW-Q139.1.mp3, Display format: Audio file (play in embedded player on page))
677	[aim2_doxypep_dh]<br><br>Show the field ONLY if: ([event-name]='baseline_visit_arm_1' or [event-name]='6_month_visit_arm_1' or [event-name]='12_month_visit_arm_1' or [event-name]='18_month_visit_arm_1') AND [acasi_language]='DH' AND [randomization_arm_1][randomization_group]='1'		descriptive<br>(Attachment: DH-Q139.1L.mp3, Display format: Audio file (play in embedded player on page))
678	[aim2_st_eng]<br><br>Show the field ONLY if: ([event-name]='baseline_visit_arm_1' or [event-name]='6_month_visit_arm_1' or [event-name]='12_month_visit_arm_1' or [event-name]='18_month_visit_arm_1') AND [acasi_language]='EN' AND [randomization_arm_1][randomization_group]='2'		descriptive<br>(Attachment: Q139.3E.mp3, Display format: Audio file (play in embedded player on page))
679	[aim2_st_sw]<br><br>Show the field ONLY if: ([event-name]='baseline_visit_arm_1' or [event-name]='6_month_visit_arm_1' or [event-name]='12_month_visit_arm_1' or [event-name]='18_month_		descriptive<br>(Attachment: SW-Q139.3.mp3, Display format: Audio file (play in embedded player on page))

	visit_arm_1') AND [acasi_language]='SW' AND [randomization_arm_1][randomization_group]='2'		
680	[aim2_st_dh]<br><br>Show the field ONLY if:<br>([event-name]='baseline_visit_arm_1' or [event-name]='6_month_visit_arm_1' or [event-name]='12_month_visit_arm_1' or [event-name]='18_month_visit_arm_1') AND [acasi_language]='DH' AND [randomization_arm_1][randomization_group]='2'		descriptive<br>(Attachment: DH-Q139.3L.mp3, Display format: Audio file (play in embedded player on page))
681	[aim2_ppt_eng]<br><br>Show the field ONLY if:<br>([event-name]='baseline_visit_arm_1' or [event-name]='6_month_visit_arm_1' or [event-name]='12_month_visit_arm_1' or [event-name]='18_month_visit_arm_1') AND [acasi_language]='EN' AND [randomization_arm_1][randomization_group]='0'		descriptive<br>(Attachment: Q139.PPT.E.mp3, Display format: Audio file (play in embedded player on page))
682	[aim2_ppt_sw]<br><br>Show the field ONLY if:<br>([event-name]='baseline_visit_arm_1' or [event-name]='6_month_visit_arm_1' or [event-name]='12_month_visit_arm_1' or [event-name]='18_month_visit_arm_1') AND [acasi_language]='SW' AND [randomization_arm_1][randomization_group]='0'		descriptive<br>(Attachment: Q139.PPT.K.mp3, Display format: Audio file (play in embedded player on page))
683	[aim2_ppt_dh]<br><br>Show the field ONLY if:<br>([event-name]='baseline_visit_arm_1' or [event-name]='6_month_visit_arm_1' or [event-name]='12_month_visit_arm_1' or [event-name]='18_month_visit_arm_1') AND [acasi_language]='DH' AND [randomization_arm_1][randomization_group]='0'		descriptive<br>(Attachment: Q139.PPT.L.mp3, Display format: Audio file (play in embedded player on page))
684	[aim2]<br><br>Show the field ONLY if:<br>[event-name]='baseline_visit_arm_1' or [event-na	[randomization_arm_1][randomization_group] is appealing to me.	radio, Required<br>1 Completely disagree<br>2 Disagree

	me]='6_month_visit_arm_1' or [event-name]='12_month_visit_arm_1' or [event-name]='18_month_visit_arm_1'		3 Neither agree nor disagree<br>4 Agree<br>5 Completely agree<br>6 Refuse to answer
685	[aim3_doxypep_eng]<br><br>Show the field ONLY if:<br>([event-name]='baseline_visit_arm_1' or [event-name]='6_month_visit_arm_1' or [event-name]='12_month_visit_arm_1' or [event-name]='18_month_visit_arm_1') AND [acasi_language]='EN' AND [randomization_arm_1][randomization_group]='1'		descriptive<br>(Attachment: Q140.1E.mp3, Display format: Audio file (play in embedded player on page))
686	[aim3_doxypep_sw]<br><br>Show the field ONLY if:<br>([event-name]='baseline_visit_arm_1' or [event-name]='6_month_visit_arm_1' or [event-name]='12_month_visit_arm_1' or [event-name]='18_month_visit_arm_1') AND [acasi_language]='SW' AND [randomization_arm_1][randomization_group]='1'		descriptive<br>(Attachment: SW-Q140.1.mp3, Display format: Audio file (play in embedded player on page))
687	[aim3_doxypep_dh]<br><br>Show the field ONLY if:<br>([event-name]='baseline_visit_arm_1' or [event-name]='6_month_visit_arm_1' or [event-name]='12_month_visit_arm_1' or [event-name]='18_month_visit_arm_1') AND [acasi_language]='DH' AND [randomization_arm_1][randomization_group]='1'		descriptive<br>(Attachment: DH-Q140.1L.mp3, Display format: Audio file (play in embedded player on page))
688	[aim3_ppt_eng]<br><br>Show the field ONLY if:<br>([event-name]='baseline_visit_arm_1' or [event-name]='6_month_visit_arm_1' or [event-name]='12_month_visit_arm_1' or [event-name]='18_month_visit_arm_1') AND [acasi_language]='EN' AND [randomization_arm_1][randomization_group]='0'		descriptive<br>(Attachment: Q140.PPT.E.mp3, Display format: Audio file (play in embedded player on page))
689	[aim3_ppt_sw]<br><br>Show the field ONLY if:		descriptive<br>(Attachment: Q140.PPT.K.mp3, Display

	([event-name]='baseline_visit_arm_1' or [event-name]='6_month_visit_arm_1' or [event-name]='12_month_visit_arm_1' or [event-name]='18_month_visit_arm_1') AND [acasi_language]='SW' AND [randomization_arm_1][randomization_group]='0'		format: Audio file (play in embedded player on page))
690	[aim3_ppt_dh]<br><br>Show the field ONLY if:<br>([event-name]='baseline_visit_arm_1' or [event-name]='6_month_visit_arm_1' or [event-name]='12_month_visit_arm_1' or [event-name]='18_month_visit_arm_1') AND [acasi_language]='DH' AND [randomization_arm_1][randomization_group]='0'		descriptive<br>(Attachment: Q140.PPT.L.mp3, Display format: Audio file (play in embedded player on page))
691	[aim3_st_eng]<br><br>Show the field ONLY if:<br>([event-name]='baseline_visit_arm_1' or [event-name]='6_month_visit_arm_1' or [event-name]='12_month_visit_arm_1' or [event-name]='18_month_visit_arm_1') AND [acasi_language]='EN' AND [randomization_arm_1][randomization_group]='2'		descriptive<br>(Attachment: Q140.3E.mp3, Display format: Audio file (play in embedded player on page))
692	[aim3_st_sw]<br><br>Show the field ONLY if:<br>([event-name]='baseline_visit_arm_1' or [event-name]='6_month_visit_arm_1' or [event-name]='12_month_visit_arm_1' or [event-name]='18_month_visit_arm_1') AND [acasi_language]='SW' AND [randomization_arm_1][randomization_group]='2'		descriptive<br>(Attachment: SW-Q140.3.mp3, Display format: Audio file (play in embedded player on page))
693	[aim3_st_dh]<br><br>Show the field ONLY if:<br>([event-name]='baseline_visit_arm_1' or [event-name]='6_month_visit_arm_1' or [event-name]='12_month_visit_arm_1' or [event-name]='18_month_visit_arm_1') AND [acasi_language]='DH' AND [ran		descriptive<br>(Attachment: DH-Q140.3L.mp3, Display format: Audio file (play in embedded player on page))

	domization_arm_1][randomization_group]='2'		
694	[aim3]<br><br>Show the field ONLY if:<br>[event-name]='baseline_visit_arm_1' or [event-name]='6_month_visit_arm_1' or [event-name]='12_month_visit_arm_1' or [event-name]='18_month_visit_arm_1'	I like [randomization_arm_1][randomization_group].	radio, Required<br>1 Completely disagree<br>2 Disagree<br>3 Neither agree nor disagree<br>4 Agree<br>5 Completely agree<br>6 Refuse to answer
695	[aim4_ppt_eng]<br><br>Show the field ONLY if:<br>([event-name]='baseline_visit_arm_1' or [event-name]='6_month_visit_arm_1' or [event-name]='12_month_visit_arm_1' or [event-name]='18_month_visit_arm_1') AND [acasi_language]='EN' AND [randomization_arm_1][randomization_group]='0'	Section Header:	descriptive<br>(Attachment: Q141.PPT.E.mp3, Display format: Audio file (play in embedded player on page))
696	[aim4_ppt_sw]<br><br>Show the field ONLY if:<br>([event-name]='baseline_visit_arm_1' or [event-name]='6_month_visit_arm_1' or [event-name]='12_month_visit_arm_1' or [event-name]='18_month_visit_arm_1') AND [acasi_language]='SW' AND [randomization_arm_1][randomization_group]='0'		descriptive<br>(Attachment: Q141.PPT.K.mp3, Display format: Audio file (play in embedded player on page))
697	[aim4_ppt_dh]<br><br>Show the field ONLY if:<br>([event-name]='baseline_visit_arm_1' or [event-name]='6_month_visit_arm_1' or [event-name]='12_month_visit_arm_1' or [event-name]='18_month_visit_arm_1') AND [acasi_language]='DH' AND [randomization_arm_1][randomization_group]='0'		descriptive<br>(Attachment: Q141.PPT.L.mp3, Display format: Audio file (play in embedded player on page))
698	[aim4_st_eng]<br><br>Show the field ONLY if:<br>([event-name]='baseline_visit_arm_1' or [event-name]='6_month_visit_arm_1' or [event-name]='12_month_visit_arm_1' or [event-name]='18_month_visit_arm_1') AND [acasi_		descriptive<br>(Attachment: Q141.3E.mp3, Display format: Audio file (play in embedded player on page))

	language]='EN' AND [randomization_arm_1][randomization_group]='2'		
699	[aim4_st_sw]<br><br>Show the field ONLY if: ([event-name]='baseline_visit_arm_1' or [event-name]='6_month_visit_arm_1' or [event-name]='12_month_visit_arm_1' or [event-name]='18_month_visit_arm_1') AND [acasi_language]='SW' AND [randomization_arm_1][randomization_group]='2'		descriptive<br>(Attachment: SW-Q141.3.mp3, Display format: Audio file (play in embedded player on page))
700	[aim4_st_dh]<br><br>Show the field ONLY if: ([event-name]='baseline_visit_arm_1' or [event-name]='6_month_visit_arm_1' or [event-name]='12_month_visit_arm_1' or [event-name]='18_month_visit_arm_1') AND [acasi_language]='DH' AND [randomization_arm_1][randomization_group]='2'		descriptive<br>(Attachment: DH-Q141.3L.mp3, Display format: Audio file (play in embedded player on page))
701	[aim4_doxypep_eng]<br><br>Show the field ONLY if: ([event-name]='baseline_visit_arm_1' or [event-name]='6_month_visit_arm_1' or [event-name]='12_month_visit_arm_1' or [event-name]='18_month_visit_arm_1') AND [acasi_language]='EN' AND [randomization_arm_1][randomization_group]='1'		descriptive<br>(Attachment: Q141.1E.mp3, Display format: Audio file (play in embedded player on page))
702	[aim4_doxypep_sw]<br><br>Show the field ONLY if: ([event-name]='baseline_visit_arm_1' or [event-name]='6_month_visit_arm_1' or [event-name]='12_month_visit_arm_1' or [event-name]='18_month_visit_arm_1') AND [acasi_language]='SW' AND [randomization_arm_1][randomization_group]='1'		descriptive<br>(Attachment: SW-Q141.1.mp3, Display format: Audio file (play in embedded player on page))
703	[aim4_doxypep_dh]<br><br>Show the field ONLY if: ([event-name]='baseline_visit_arm_1' or [event-name]='6_month_visit_arm		descriptive<br>(Attachment: DH-Q141.1L.mp3, Display format: Audio file (play in embedded player on page))

	_1' or [event-name]='12_month_visit_arm_1' or [event-name]='18_month_visit_arm_1') AND [acasi_language]='DH' AND [randomization_arm_1][randomization_group]='1'		
704	[aim4]<br><br>Show the field ONLY if:<br>[event-name]='baseline_visit_arm_1' or [event-name]='6_month_visit_arm_1' or [event-name]='12_month_visit_arm_1' or [event-name]='18_month_visit_arm_1'	I welcome [randomization_arm_1][randomization_group].	radio, Required<br>1 Completely disagree<br>2 Disagree<br>3 Neither agree nor disagree<br>4 Agree<br>5 Completely agree<br>6 Refuse to answer
705	[fim1_doxypep_eng]<br><br>Show the field ONLY if:<br>([event-name]='baseline_visit_arm_1' or [event-name]='6_month_visit_arm_1' or [event-name]='12_month_visit_arm_1' or [event-name]='18_month_visit_arm_1') AND [acasi_language]='EN' AND [randomization_arm_1][randomization_group]='1'		descriptive<br>(Attachment: Q142.1E.mp3, Display format: Audio file (play in embedded player on page))
706	[fim1_doxypep_sw]<br><br>Show the field ONLY if:<br>([event-name]='baseline_visit_arm_1' or [event-name]='6_month_visit_arm_1' or [event-name]='12_month_visit_arm_1' or [event-name]='18_month_visit_arm_1') AND [acasi_language]='SW' AND [randomization_arm_1][randomization_group]='1'		descriptive<br>(Attachment: SW-Q142.1.mp3, Display format: Audio file (play in embedded player on page))
707	[fim1_doxypep_dh]<br><br>Show the field ONLY if:<br>([event-name]='baseline_visit_arm_1' or [event-name]='6_month_visit_arm_1' or [event-name]='12_month_visit_arm_1' or [event-name]='18_month_visit_arm_1') AND [acasi_language]='DH' AND [randomization_arm_1][randomization_group]='1'		descriptive<br>(Attachment: DH-Q142.1L.mp3, Display format: Audio file (play in embedded player on page))
708	[fim1_st_eng]<br><br>Show the field ONLY if:<br>([event-name]='baseline_visit_arm_1' or [event-na		descriptive<br>(Attachment: Q142.3E.mp3, Display format: Audio file (play in embedded player on page))

	me]='6_month_visit_arm_1' or [event-name]='12_month_visit_arm_1' or [event-name]='18_month_visit_arm_1') AND [acasi_language]='EN' AND [randomization_arm_1][randomization_group]='2'		
709	[fim1_st_sw]<br><br>Show the field ONLY if:<br>([event-name]='baseline_visit_arm_1' or [event-name]='6_month_visit_arm_1' or [event-name]='12_month_visit_arm_1' or [event-name]='18_month_visit_arm_1') AND [acasi_language]='SW' AND [randomization_arm_1][randomization_group]='2'		descriptive<br>(Attachment: SW-Q142.3.mp3, Display format: Audio file (play in embedded player on page))
710	[fim1_st_dh]<br><br>Show the field ONLY if:<br>([event-name]='baseline_visit_arm_1' or [event-name]='6_month_visit_arm_1' or [event-name]='12_month_visit_arm_1' or [event-name]='18_month_visit_arm_1') AND [acasi_language]='DH' AND [randomization_arm_1][randomization_group]='2'		descriptive<br>(Attachment: DH-Q142.3L.mp3, Display format: Audio file (play in embedded player on page))
711	[fim1_ppt_eng]<br><br>Show the field ONLY if:<br>([event-name]='baseline_visit_arm_1' or [event-name]='6_month_visit_arm_1' or [event-name]='12_month_visit_arm_1' or [event-name]='18_month_visit_arm_1') AND [acasi_language]='EN' AND [randomization_arm_1][randomization_group]='0'		descriptive<br>(Attachment: Q142.PPT.E.mp3, Display format: Audio file (play in embedded player on page))
712	[fim1_ppt_sw]<br><br>Show the field ONLY if:<br>([event-name]='baseline_visit_arm_1' or [event-name]='6_month_visit_arm_1' or [event-name]='12_month_visit_arm_1' or [event-name]='18_month_visit_arm_1') AND [acasi_language]='SW' AND [randomization_arm_1][randomization_group]='0'		descriptive<br>(Attachment: Q142.PPT.K.mp3, Display format: Audio file (play in embedded player on page))

713	[fim1_ppt_dh]<br><br>Show the field ONLY if:<br>([event-name]='baseline_visit_arm_1' or [event-name]='6_month_visit_arm_1' or [event-name]='12_month_visit_arm_1' or [event-name]='18_month_visit_arm_1') AND [acasi_language]='DH' AND [randomization_arm_1][randomization_group]='0'		descriptive<br>(Attachment: Q142.PPT.L.mp3, Display format: Audio file (play in embedded player on page))
714	[fim1]<br><br>Show the field ONLY if:<br>[event-name]='baseline_visit_arm_1' or [event-name]='6_month_visit_arm_1' or [event-name]='12_month_visit_arm_1' or [event-name]='18_month_visit_arm_1'	[randomization_arm_1][randomization_group] seems implementable.	radio, Required<br>1 Completely disagree<br>2 Disagree<br>3 Neither agree nor disagree<br>4 Agree<br>5 Completely agree<br>6 Refuse to answer
715	[fim2_doxypep_eng]<br><br>Show the field ONLY if:<br>([event-name]='baseline_visit_arm_1' or [event-name]='6_month_visit_arm_1' or [event-name]='12_month_visit_arm_1' or [event-name]='18_month_visit_arm_1') AND [acasi_language]='EN' AND [randomization_arm_1][randomization_group]='1'		descriptive<br>(Attachment: Q143.1 E.mp3, Display format: Audio file (play in embedded player on page))
716	[fim2_doxypep_sw]<br><br>Show the field ONLY if:<br>([event-name]='baseline_visit_arm_1' or [event-name]='6_month_visit_arm_1' or [event-name]='12_month_visit_arm_1' or [event-name]='18_month_visit_arm_1') AND [acasi_language]='SW' AND [randomization_arm_1][randomization_group]='1'		descriptive<br>(Attachment: SW-Q143.1.mp3, Display format: Audio file (play in embedded player on page))
717	[fim2_doxypep_dh]<br><br>Show the field ONLY if:<br>([event-name]='baseline_visit_arm_1' or [event-name]='6_month_visit_arm_1' or [event-name]='12_month_visit_arm_1' or [event-name]='18_month_visit_arm_1') AND [acasi_language]='DH' AND [ran		descriptive<br>(Attachment: DH-Q143.1L.mp3, Display format: Audio file (play in embedded player on page))

	domization_arm_1][randomization_group]='1'		
718	[fim2_st_eng]<br><br>Show the field ONLY if: ([event-name]='baseline_visit_arm_1' or [event-name]='6_month_visit_arm_1' or [event-name]='12_month_visit_arm_1' or [event-name]='18_month_visit_arm_1') AND [acasi_language]='EN' AND [randomization_arm_1][randomization_group]='2'		descriptive<br>(Attachment: Q143.3E.mp3, Display format: Audio file (play in embedded player on page))
719	[fim2_st_sw]<br><br>Show the field ONLY if: ([event-name]='baseline_visit_arm_1' or [event-name]='6_month_visit_arm_1' or [event-name]='12_month_visit_arm_1' or [event-name]='18_month_visit_arm_1') AND [acasi_language]='SW' AND [randomization_arm_1][randomization_group]='2'		descriptive<br>(Attachment: SW-Q143.3.mp3, Display format: Audio file (play in embedded player on page))
720	[fim2_st_dh]<br><br>Show the field ONLY if: ([event-name]='baseline_visit_arm_1' or [event-name]='6_month_visit_arm_1' or [event-name]='12_month_visit_arm_1' or [event-name]='18_month_visit_arm_1') AND [acasi_language]='DH' AND [randomization_arm_1][randomization_group]='2'		descriptive<br>(Attachment: DH-Q143.3L.mp3, Display format: Audio file (play in embedded player on page))
721	[fim2_ppt_eng]<br><br>Show the field ONLY if: ([event-name]='baseline_visit_arm_1' or [event-name]='6_month_visit_arm_1' or [event-name]='12_month_visit_arm_1' or [event-name]='18_month_visit_arm_1') AND [acasi_language]='EN' AND [randomization_arm_1][randomization_group]='0'		descriptive<br>(Attachment: Q143.PPT.E.mp3, Display format: Audio file (play in embedded player on page))
722	[fim2_ppt_sw]<br><br>Show the field ONLY if: ([event-name]='baseline_visit_arm_1' or [event-name]='6_month_visit_arm_1' or [event-name]='12_		descriptive<br>(Attachment: Q143.PPT.K.mp3, Display format: Audio file (play in embedded player on page))

	month_visit_arm_1' or [event-name]='18_month_visit_arm_1') AND [acasi_language]='SW' AND [randomization_arm_1][randomization_group]='0'		
723	[fim2_ppt_dh]<br><br>Show the field ONLY if:<br>([event-name]='baseline_visit_arm_1' or [event-name]='6_month_visit_arm_1' or [event-name]='12_month_visit_arm_1' or [event-name]='18_month_visit_arm_1') AND [acasi_language]='DH' AND [randomization_arm_1][randomization_group]='0'		descriptive<br>(Attachment: Q143.PPT.L.mp3, Display format: Audio file (play in embedded player on page))
724	[fim2]<br><br>Show the field ONLY if:<br>[event-name]='baseline_visit_arm_1' or [event-name]='6_month_visit_arm_1' or [event-name]='12_month_visit_arm_1' or [event-name]='18_month_visit_arm_1'	[randomization_arm_1][randomization_group] seems possible.	radio, Required<br>1 Completely disagree<br>2 Disagree<br>3 Neither agree nor disagree<br>4 Agree<br>5 Completely agree<br>6 Refuse to answer
725	[fim3_doxypep_eng]<br><br>Show the field ONLY if:<br>([event-name]='baseline_visit_arm_1' or [event-name]='6_month_visit_arm_1' or [event-name]='12_month_visit_arm_1' or [event-name]='18_month_visit_arm_1') AND [acasi_language]='EN' AND [randomization_arm_1][randomization_group]='1'	Section Header:	descriptive<br>(Attachment: Q144.1E.mp3, Display format: Audio file (play in embedded player on page))
726	[fim3_doxypep_sw]<br><br>Show the field ONLY if:<br>([event-name]='baseline_visit_arm_1' or [event-name]='6_month_visit_arm_1' or [event-name]='12_month_visit_arm_1' or [event-name]='18_month_visit_arm_1') AND [acasi_language]='SW' AND [randomization_arm_1][randomization_group]='1'		descriptive<br>(Attachment: SW-Q144.1.mp3, Display format: Audio file (play in embedded player on page))
727	[fim3_doxypep_dh]<br><br>Show the field ONLY if:<br>([event-name]='baseline_visit_arm_1' or [event-name]='6_month_visit_arm		descriptive<br>(Attachment: DH-Q144.1L.mp3, Display format: Audio file (play in embedded player on page))

	_1' or [event-name]='12_month_visit_arm_1' or [event-name]='18_month_visit_arm_1') AND [acasi_language]='DH' AND [randomization_arm_1][randomization_group]='1'		
728	[fim3_st_eng]<br><br>Show the field ONLY if:<br>([event-name]='baseline_visit_arm_1' or [event-name]='6_month_visit_arm_1' or [event-name]='12_month_visit_arm_1' or [event-name]='18_month_visit_arm_1') AND [acasi_language]='EN' AND [randomization_arm_1][randomization_group]='2'		descriptive<br>(Attachment: Q144.3E.mp3, Display format: Audio file (play in embedded player on page))
729	[fim3_st_sw]<br><br>Show the field ONLY if:<br>([event-name]='baseline_visit_arm_1' or [event-name]='6_month_visit_arm_1' or [event-name]='12_month_visit_arm_1' or [event-name]='18_month_visit_arm_1') AND [acasi_language]='SW' AND [randomization_arm_1][randomization_group]='2'		descriptive<br>(Attachment: SW-Q144.3.mp3, Display format: Audio file (play in embedded player on page))
730	[fim3_st_dh]<br><br>Show the field ONLY if:<br>([event-name]='baseline_visit_arm_1' or [event-name]='6_month_visit_arm_1' or [event-name]='12_month_visit_arm_1' or [event-name]='18_month_visit_arm_1') AND [acasi_language]='DH' AND [randomization_arm_1][randomization_group]='2'		descriptive<br>(Attachment: DH-Q144.3L.mp3, Display format: Audio file (play in embedded player on page))
731	[fim3_ppt_eng]<br><br>Show the field ONLY if:<br>([event-name]='baseline_visit_arm_1' or [event-name]='6_month_visit_arm_1' or [event-name]='12_month_visit_arm_1' or [event-name]='18_month_visit_arm_1') AND [acasi_language]='EN' AND [randomization_arm_1][randomization_group]='0'		descriptive<br>(Attachment: Q144.PPT.E.mp3, Display format: Audio file (play in embedded player on page))
732	[fim3_ppt_sw]		descriptive<br>(Attachment: Q144.PPT.K.mp3, Display

	Show the field ONLY if:<br>([event-name]='baseline_visit_arm_1' or [event-name]='6_month_visit_arm_1' or [event-name]='12_month_visit_arm_1' or [event-name]='18_month_visit_arm_1') AND [acasi_language]='SW' AND [randomization_arm_1][randomization_group]='0'		format: Audio file (play in embedded player on page))
733	[fim3_ppt_dh]<br><br>Show the field ONLY if:<br>([event-name]='baseline_visit_arm_1' or [event-name]='6_month_visit_arm_1' or [event-name]='12_month_visit_arm_1' or [event-name]='18_month_visit_arm_1') AND [acasi_language]='DH' AND [randomization_arm_1][randomization_group]='0'		descriptive<br>(Attachment: Q144.PPT.L.mp3, Display format: Audio file (play in embedded player on page))
734	[fim3]<br><br>Show the field ONLY if:<br>[event-name]='baseline_visit_arm_1' or [event-name]='6_month_visit_arm_1' or [event-name]='12_month_visit_arm_1' or [event-name]='18_month_visit_arm_1'	[randomization_arm_1][randomization_group] seems doable.	radio, Required<br>1 Completely disagree<br>2 Disagree<br>3 Neither agree nor disagree<br>4 Agree<br>5 Completely agree<br>6 Refuse to answer
735	[fim4_doxypep_eng]<br><br>Show the field ONLY if:<br>([event-name]='baseline_visit_arm_1' or [event-name]='6_month_visit_arm_1' or [event-name]='12_month_visit_arm_1' or [event-name]='18_month_visit_arm_1') AND [acasi_language]='EN' AND [randomization_arm_1][randomization_group]='1'		descriptive<br>(Attachment: Q145.1E.mp3, Display format: Audio file (play in embedded player on page))
736	[fim4_doxypep_sw]<br><br>Show the field ONLY if:<br>([event-name]='baseline_visit_arm_1' or [event-name]='6_month_visit_arm_1' or [event-name]='12_month_visit_arm_1' or [event-name]='18_month_visit_arm_1') AND [acasi_language]='SW' AND [randomization_arm_1][randomization_group]='1'		descriptive<br>(Attachment: SW-Q145.1.mp3, Display format: Audio file (play in embedded player on page))

737	[fim4_doxypep_dh]<br><br>Show the field ONLY if:<br>([event-name]='baseline_visit_arm_1' or [event-name]='6_month_visit_arm_1' or [event-name]='12_month_visit_arm_1' or [event-name]='18_month_visit_arm_1') AND [acasi_language]='DH' AND [randomization_arm_1][randomization_group]='1'		descriptive<br>(Attachment: DH-Q145.1L.mp3, Display format: Audio file (play in embedded player on page))
738	[fim4_st_eng]<br><br>Show the field ONLY if:<br>([event-name]='baseline_visit_arm_1' or [event-name]='6_month_visit_arm_1' or [event-name]='12_month_visit_arm_1' or [event-name]='18_month_visit_arm_1') AND [acasi_language]='EN' AND [randomization_arm_1][randomization_group]='2'		descriptive<br>(Attachment: Q145.3E.mp3, Display format: Audio file (play in embedded player on page))
739	[fim4_st_sw]<br><br>Show the field ONLY if:<br>([event-name]='baseline_visit_arm_1' or [event-name]='6_month_visit_arm_1' or [event-name]='12_month_visit_arm_1' or [event-name]='18_month_visit_arm_1') AND [acasi_language]='SW' AND [randomization_arm_1][randomization_group]='2'		descriptive<br>(Attachment: SW-Q145.3.mp3, Display format: Audio file (play in embedded player on page))
740	[fim4_st_dh]<br><br>Show the field ONLY if:<br>([event-name]='baseline_visit_arm_1' or [event-name]='6_month_visit_arm_1' or [event-name]='12_month_visit_arm_1' or [event-name]='18_month_visit_arm_1') AND [acasi_language]='DH' AND [randomization_arm_1][randomization_group]='2'		descriptive<br>(Attachment: DH-Q145.3L.mp3, Display format: Audio file (play in embedded player on page))
741	[fim4_ppt_eng]<br><br>Show the field ONLY if:<br>([event-name]='baseline_visit_arm_1' or [event-name]='6_month_visit_arm_1' or [event-name]='12_month_visit_arm_1' or [event-name]='18_month_		descriptive<br>(Attachment: Q145.PPT.E.mp3, Display format: Audio file (play in embedded player on page))

	visit_arm_1') AND [acasi_language]='EN' AND [randomization_arm_1][randomization_group]='0'		
742	[fim4_ppt_sw]<br><br>Show the field ONLY if:<br>([event-name]='baseline_visit_arm_1' or [event-name]='6_month_visit_arm_1' or [event-name]='12_month_visit_arm_1' or [event-name]='18_month_visit_arm_1') AND [acasi_language]='SW' AND [randomization_arm_1][randomization_group]='0'		descriptive<br>(Attachment: Q145.PPT.K.mp3, Display format: Audio file (play in embedded player on page))
743	[fim4_ppt_dh]<br><br>Show the field ONLY if:<br>([event-name]='baseline_visit_arm_1' or [event-name]='6_month_visit_arm_1' or [event-name]='12_month_visit_arm_1' or [event-name]='18_month_visit_arm_1') AND [acasi_language]='DH' AND [randomization_arm_1][randomization_group]='0'		descriptive<br>(Attachment: Q145.PPT.L.mp3, Display format: Audio file (play in embedded player on page))
744	[fim4]<br><br>Show the field ONLY if:<br>[event-name]='baseline_visit_arm_1' or [event-name]='6_month_visit_arm_1' or [event-name]='12_month_visit_arm_1' or [event-name]='18_month_visit_arm_1'	[randomization_arm_1][randomization_group] seems easy to use.	radio, Required<br>1 Completely disagree<br>2 Disagree<br>3 Neither agree nor disagree<br>4 Agree<br>5 Completely agree<br>6 Refuse to answer
745	[forget_doxypep_eng]<br><br>Show the field ONLY if:<br>[randomization_arm_1][randomization_group]='1' and ([event-name]= '3_month_visit_arm_1' or [event-name]='6_month_visit_arm_1' or [event-name] = '9_month_visit_arm_1' or [event-name]='12_month_visit_arm_1' or [event-name]='15_month_visit_arm_1' or [event-name]='18_month_visit_arm_1') AND [acasi_language]='EN'	Section Header: *DoxyPEP Adherence (quarterly if in doxyPEP arm)*	descriptive<br>(Attachment: Q146.mp3, Display format: Audio file (play in embedded player on page))
746	[forget_doxypep_sw]<br><br>Show the field ONLY if:		descriptive<br>(Attachment: SW-Q146.mp3, Display format:

	[randomization_arm_1][randomization_group]='1' and ([event-name]= '3_month_visit_arm_1' or [event-name]='6_month_visit_arm_1' or [event-name] = '9_month_visit_arm_1' or [event-name]='12_month_visit_arm_1' or [event-name]='15_month_visit_arm_1' or [event-name] ='18_month_visit_arm_1') AND [acasi_language] ='SW'		Audio file (play in embedded player on page))
747	[forget_doxypep_dh]<br><br>Show the field ONLY if: [randomization_arm_1][randomization_group]='1' and ([event-name]= '3_month_visit_arm_1' or [event-name]='6_month_visit_arm_1' or [event-name] = '9_month_visit_arm_1' or [event-name]='12_month_visit_arm_1' or [event-name]='15_month_visit_arm_1' or [event-name] ='18_month_visit_arm_1') AND [acasi_language] ='DH'		descriptive<br>(Attachment: DH-Q146.mp3, Display format: Audio file (play in embedded player on page))
748	[forget_doxypep]<br><br>Show the field ONLY if: [randomization_arm_1][randomization_group]='1' and ([event-name]= '3_month_visit_arm_1' or [event-name]='6_month_visit_arm_1' or [event-name] = '9_month_visit_arm_1' or [event-name]='12_month_visit_arm_1' or [event-name]='15_month_visit_arm_1' or [event-name] ='18_month_visit_arm_1')	Sometimes people may have difficulty remembering to take their medicines as instructed. DoxyPEP (2 tablets of doxycycline) is taken 24-72 hours after you have condomless sex, as post-exposure prophylaxis (PEP) to prevent sexually transmitted infections. During the last 3 months, did you miss taking your doxyPEP as instructed?	radio, Required<br>1 No<br>2 Yes<br>3 Refuse to answer
749	[doxypep_adhere_eng]<br><br>Show the field ONLY if: [randomization_arm_1][randomization_group]='1' and ([event-name]= '3_month_visit_arm_1' or [event-name]='6_month_visit_arm_1' or [event-name] = '9_month_visit_arm_1' or [event-name]='12_month_visit_arm_1' or [event-name]='15_month_visit_arm_1' or [event-name]		descriptive<br>(Attachment: Q147.mp3, Display format: Audio file (play in embedded player on page))

	='18_month_visit_arm_1') AND [acasi_language] ='EN'		
750	[doxypep_adhere_sw]<br><br>Show the field ONLY if: [randomization_arm_1][randomization_group]='1' and ([event-name]= '3_month_visit_arm_1' or [event-name]='6_month_visit_arm_1' or [event-name] = '9_month_visit_arm_1' or [event-name]='12_month_visit_arm_1' or [event-name]='15_month_visit_arm_1' or [event-name] ='18_month_visit_arm_1') AND [acasi_language] ='SW'		descriptive<br>(Attachment: SW-Q147.mp3, Display format: Audio file (play in embedded player on page))
751	[doxypep_adhere_dh]<br><br>Show the field ONLY if: [randomization_arm_1][randomization_group]='1' and ([event-name]= '3_month_visit_arm_1' or [event-name]='6_month_visit_arm_1' or [event-name] = '9_month_visit_arm_1' or [event-name]='12_month_visit_arm_1' or [event-name]='15_month_visit_arm_1' or [event-name] ='18_month_visit_arm_1') AND [acasi_language] ='DH'		descriptive<br>(Attachment: DH-Q147.mp3, Display format: Audio file (play in embedded player on page))
752	[doxypep_adhere]<br><br>Show the field ONLY if: [randomization_arm_1][randomization_group]='1' and ([event-name]= '3_month_visit_arm_1' or [event-name]='6_month_visit_arm_1' or [event-name] = '9_month_visit_arm_1' or [event-name]='12_month_visit_arm_1' or [event-name]='15_month_visit_arm_1' or [event-name] ='18_month_visit_arm_1')	In the last 3 months, how good a job did you do at taking your doxyPEP after condomless sex?	radio, Required<br>1 Very poor<br>2 Poor<br>3 Fair<br>4 Good<br>5 Very good<br>6 Excellent<br>7 Don't know<br>8 Refuse to answer
753	[doxypep_times_eng]<br><br>Show the field ONLY if: [randomization_arm_1][randomization_group]='1' and ([event-name]= '3_month_visit_arm_1' or [event-name]='6_month_visit		descriptive<br>(Attachment: Q148.mp3, Display format: Audio file (play in embedded player on page))

	_arm_1' or [event-name] = '9_month_visit_arm_1' or [event-name]='12_month_visit_arm_1' or [event-name]='15_month_visit_arm_1' or [event-name] ='18_month_visit_arm_1') AND [acasi_language] ='EN'		
754	[doxypep_times_sw]<br><br>Show the field ONLY if: [randomization_arm_1][randomization_group]='1' and ([event-name]= '3_month_visit_arm_1' or [event-name]='6_month_visit_arm_1' or [event-name] = '9_month_visit_arm_1' or [event-name]='12_month_visit_arm_1' or [event-name]='15_month_visit_arm_1' or [event-name] ='18_month_visit_arm_1') AND [acasi_language] ='SW'		descriptive<br>(Attachment: SW-Q148.mp3, Display format: Audio file (play in embedded player on page))
755	[doxypep_times_dh]<br><br>Show the field ONLY if: [randomization_arm_1][randomization_group]='1' and ([event-name]= '3_month_visit_arm_1' or [event-name]='6_month_visit_arm_1' or [event-name] = '9_month_visit_arm_1' or [event-name]='12_month_visit_arm_1' or [event-name]='15_month_visit_arm_1' or [event-name] ='18_month_visit_arm_1') AND [acasi_language] ='DH'		descriptive<br>(Attachment: DH-Q148.mp3, Display format: Audio file (play in embedded player on page))
756	[doxypep_times]<br><br>Show the field ONLY if: [randomization_arm_1][randomization_group]='1' and ([event-name]= '3_month_visit_arm_1' or [event-name]='6_month_visit_arm_1' or [event-name] = '9_month_visit_arm_1' or [event-name]='12_month_visit_arm_1' or [event-name]='15_month_visit_arm_1' or [event-name] ='18_month_visit_arm_1')	Please think carefully about the last month. In the last 30 days, how many times did you take your doxyPEP after condomless sex?	text (number, Min: 0, Max: 31), Required
757	[doxypep_misscause_eng]	Section Header:	descriptive<br>(Attachment: Q149.mp3, Display format:

	Show the field ONLY if: [randomization_arm_1][randomization_group]='1' and ([event-name]= '3_month_visit_arm_1' or [event-name]='6_month_visit_arm_1' or [event-name] = '9_month_visit_arm_1' or [event-name]='12_month_visit_arm_1' or [event-name]='15_month_visit_arm_1' or [event-name] ='18_month_visit_arm_1') AND [acasi_language] ='EN'		Audio file (play in embedded player on page))
758	[doxypep_misscause_sw]<br><br>Show the field ONLY if: [randomization_arm_1][randomization_group]='1' and ([event-name]= '3_month_visit_arm_1' or [event-name]='6_month_visit_arm_1' or [event-name] = '9_month_visit_arm_1' or [event-name]='12_month_visit_arm_1' or [event-name]='15_month_visit_arm_1' or [event-name] ='18_month_visit_arm_1') AND [acasi_language] ='SW'		descriptive<br>(Attachment: SW-Q149.mp3, Display format: Audio file (play in embedded player on page))
759	[doxypep_misscause_dh]<br><br>Show the field ONLY if: [randomization_arm_1][randomization_group]='1' and ([event-name]= '3_month_visit_arm_1' or [event-name]='6_month_visit_arm_1' or [event-name] = '9_month_visit_arm_1' or [event-name]='12_month_visit_arm_1' or [event-name]='15_month_visit_arm_1' or [event-name] ='18_month_visit_arm_1') AND [acasi_language] ='DH'		descriptive<br>(Attachment: DH-Q149.mp3, Display format: Audio file (play in embedded player on page))
760	[doxypep_misscause]<br><br>Show the field ONLY if: [randomization_arm_1][randomization_group]='1' and ([event-name]= '3_month_visit_arm_1' or [event-name]='6_month_visit_arm_1' or [event-name] = '9_month_visit_arm_1' or [event-name]='12_month_visit_arm_1' or [even	During the last 30 days, what caused you to miss taking your doxyPEP pills after condomless sex?<br><br>Check all that apply.	checkbox, Required<br>1 doxypep_misscause___1 Busy<br>2 doxypep_misscause___2 Traveled or oth change in routi<br>3 doxypep_misscause___3 Forgot<br>4 doxypep_misscause___4 No condomless<br>5 doxypep_misscause___5 Problem with confidentiality/<br>6 doxypep_misscause___6 Too many pills

	t-name]='15_month_visit_arm_1' or [event-name]='18_month_visit_arm_1')		<table><tr><td>7</td><td>doxypep_misscause___7</td><td>Missed a refill/r</td></tr><tr><td>8</td><td>doxypep_misscause___8</td><td>Illness</td></tr><tr><td>9</td><td>doxypep_misscause___9</td><td>Lost or stolen p</td></tr><tr><td>10</td><td>doxypep_misscause___10</td><td>Side effects: dru or harmful</td></tr><tr><td>11</td><td>doxypep_misscause___11</td><td>Felt depressed/ove</td></tr><tr><td>12</td><td>doxypep_misscause___12</td><td>Shared pills wit someone else</td></tr><tr><td>13</td><td>doxypep_misscause___13</td><td>Alcohol use</td></tr><tr><td>14</td><td>doxypep_misscause___14</td><td>Other (Specify)</td></tr><tr><td>15</td><td>doxypep_misscause___15</td><td>Refuse to answ</td></tr></table>
761	[other_doxypepmiss]<br><br>Show the field ONLY if:<br>[doxypep_misscause(14)] = '1'	Other reason (specify)	text
762	[disclose_doxypep_eng]<br><br>Show the field ONLY if:<br>[randomization_arm_1][randomization_group]='1' and ([event-name]= '3_month_visit_arm_1' or [event-name]='6_month_visit_arm_1' or [event-name] = '9_month_visit_arm_1' or [event-name]='12_month_visit_arm_1' or [event-name]='15_month_visit_arm_1' or [event-name]='18_month_visit_arm_1') AND [acasi_language]='EN'		descriptive<br>(Attachment: Q150.mp3, Display format: Audio file (play in embedded player on page))
763	[disclose_doxypep_sw]<br><br>Show the field ONLY if:<br>[randomization_arm_1][randomization_group]='1' and ([event-name]= '3_month_visit_arm_1' or [event-name]='6_month_visit_arm_1' or [event-name] = '9_month_visit_arm_1' or [event-name]='12_month_visit_arm_1' or [event-name]='15_month_visit_arm_1' or [event-name]='18_month_visit_arm_1') AND [acasi_language]='SW'		descriptive<br>(Attachment: SW-Q150.mp3, Display format: Audio file (play in embedded player on page))
764	[disclose_doxypep_dh]<br><br>Show the field ONLY if:<br>[randomization_arm_1][randomization_group]='1' and ([event-name]= '3_m		descriptive<br>(Attachment: DH-Q150.mp3, Display format: Audio file (play in embedded player on page))

	onth_visit_arm_1' or [event-name]='6_month_visit_arm_1' or [event-name] = '9_month_visit_arm_1' or [event-name]='12_month_visit_arm_1' or [event-name]='15_month_visit_arm_1' or [event-name] ='18_month_visit_arm_1') AND [acasi_language] ='DH'		
765	[disclose_doxypep]<br><br>Show the field ONLY if:<br>[randomization_arm_1][randomization_group]='1' and ([event-name]= '3_month_visit_arm_1' or [event-name]='6_month_visit_arm_1' or [event-name] = '9_month_visit_arm_1' or [event-name]='12_month_visit_arm_1' or [event-name]='15_month_visit_arm_1' or [event-name] ='18_month_visit_arm_1')	To how many people have you disclosed that you are using doxyPEP?	text (number, Min: 0)
766	[reported_hiv_status_eng]<br><br>Show the field ONLY if:<br>[event-name]= 'baseline_visit_arm_1' AND [acasi_language]='EN'	Section Header: *HIV Status and Prevention or Care Questions*	descriptive<br>(Attachment: Q151.mp3, Display format: Audio file (play in embedded player on page))
767	[reported_hiv_status_sw]<br><br>Show the field ONLY if:<br>[event-name]= 'baseline_visit_arm_1' AND [acasi_language]='SW'		descriptive<br>(Attachment: SW-Q151.mp3, Display format: Audio file (play in embedded player on page))
768	[reported_hiv_status_dh]<br><br>Show the field ONLY if:<br>[event-name]= 'baseline_visit_arm_1' AND [acasi_language]='DH'		descriptive<br>(Attachment: DH-Q151.mp3, Display format: Audio file (play in embedded player on page))
769	[reported_hiv_status]<br><br>Show the field ONLY if:<br>[event-name]= 'baseline_visit_arm_1'	Have you ever tested positive for HIV?	radio, Required<br>1 No<br>2 Yes<br>3 Refuse to answer
770	[art_ever_eng]<br><br>Show the field ONLY if:<br>[event-name]= 'baseline_visit_arm_1' and [reported_hiv_status] = '2' AND [acasi_language]='EN'		descriptive<br>(Attachment: Q152.mp3, Display format: Audio file (play in embedded player on page))

771	[art_ever_sw]<br><br>Show the field ONLY if:<br>[event-name]= 'baseline_visit_arm_1' and [reported_hiv_status] = '2' AND [acasi_language]='SW'		descriptive<br>(Attachment: SW-Q152.mp3, Display format: Audio file (play in embedded player on page))
772	[art_ever_dh]<br><br>Show the field ONLY if:<br>[event-name]= 'baseline_visit_arm_1' and [reported_hiv_status] = '2' AND [acasi_language]='DH'		descriptive<br>(Attachment: DH-Q152.mp3, Display format: Audio file (play in embedded player on page))
773	[art_ever]<br><br>Show the field ONLY if:<br>[event-name]= 'baseline_visit_arm_1' and [reported_hiv_status] = '2'	Have you ever taken antiretroviral medication (called ART) to treat HIV?	radio, Required<br>1 No<br>2 Yes<br>3 Refuse to answer
774	[art_taking_eng]<br><br>Show the field ONLY if:<br>[baseline_visit_arm_1][reported_hiv_status] = '2' AND [acasi_language]='EN'		descriptive<br>(Attachment: Q153.mp3, Display format: Audio file (play in embedded player on page))
775	[art_taking_sw]<br><br>Show the field ONLY if:<br>[baseline_visit_arm_1][reported_hiv_status] = '2' AND [acasi_language]='SW'		descriptive<br>(Attachment: SW-Q153.mp3, Display format: Audio file (play in embedded player on page))
776	[art_taking_dh]<br><br>Show the field ONLY if:<br>[baseline_visit_arm_1][reported_hiv_status] = '2' AND [acasi_language]='DH'		descriptive<br>(Attachment: DH-Q153.mp3, Display format: Audio file (play in embedded player on page))
777	[art_taking]<br><br>Show the field ONLY if:<br>[baseline_visit_arm_1][reported_hiv_status] = '2'	Are you currently taking antiretroviral medication (called ART) to treat HIV?	radio, Required<br>1 No<br>2 Yes<br>3 Refuse to answer
778	[miss_art_eng]<br><br>Show the field ONLY if:<br>[baseline_visit_arm_1][reported_hiv_status] = '2' and [art_taking]='2' AND [acasi_language]='EN'	Section Header:	descriptive<br>(Attachment: Q154.mp3, Display format: Audio file (play in embedded player on page))
779	[miss_art_sw]<br><br>Show the field ONLY if:<br>[baseline_visit_arm_1][reported_hiv_status] = '2' and [art_taking]='2' AND [acasi_language]='SW'		descriptive<br>(Attachment: SW-Q154.mp3, Display format: Audio file (play in embedded player on page))

780	[miss_art_dh]<br><br>Show the field ONLY if:<br>[baseline_visit_arm_1][reported_hiv_status] = '2' and [art_taking]='2' AND [acasi_language]='DH'		descriptive<br>(Attachment: DH-Q154.mp3, Display format: Audio file (play in embedded player on page))
781	[miss_art]<br><br>Show the field ONLY if:<br>[baseline_visit_arm_1][reported_hiv_status] = '2' and [art_taking]='2'	Sometimes people may have difficulty remembering to take their medicines as instructed. During the last 30 days, did you miss taking your ART as instructed?	radio, Required<br>1 No<br>2 Yes<br>3 Refuse to answer
782	[times_miss_art_eng]<br><br>Show the field ONLY if:<br>[baseline_visit_arm_1][reported_hiv_status] = '2' and [miss_art] = '2' AND [acasi_language]='EN'		descriptive<br>(Attachment: Q155.mp3, Display format: Audio file (play in embedded player on page))
783	[times_miss_art_sw]<br><br>Show the field ONLY if:<br>[baseline_visit_arm_1][reported_hiv_status] = '2' and [miss_art] = '2' AND [acasi_language]='SW'		descriptive<br>(Attachment: SW-Q155.mp3, Display format: Audio file (play in embedded player on page))
784	[times_miss_art_dh]<br><br>Show the field ONLY if:<br>[baseline_visit_arm_1][reported_hiv_status] = '2' and [miss_art] = '2' AND [acasi_language]='DH'		descriptive<br>(Attachment: DH-Q155.mp3, Display format: Audio file (play in embedded player on page))
785	[times_miss_art]<br><br>Show the field ONLY if:<br>[baseline_visit_arm_1][reported_hiv_status] = '2' and [miss_art] = '2'	In the last 30 days, how many times did you miss your ART?	text, Required
786	[ever_prep_eng]<br><br>Show the field ONLY if:<br>[event-name]= 'baseline_visit_arm_1' and [reported_hiv_status] = '1' AND [acasi_language]='EN'		descriptive<br>(Attachment: Q156.mp3, Display format: Audio file (play in embedded player on page))
787	[ever_prep_sw]<br><br>Show the field ONLY if:<br>[event-name]= 'baseline_visit_arm_1' and [reported_hiv_status] = '1' AND [acasi_language]='SW'		descriptive<br>(Attachment: SW-Q156.mp3, Display format: Audio file (play in embedded player on page))
788	[ever_prep_dh]<br><br>Show the field ONLY if:<br>[event-name]= 'baseline_visit_arm_1' and [reported_hiv_status] = '1' AND [acasi_language]='DH'		descriptive<br>(Attachment: DH-Q156.mp3, Display format: Audio file (play in embedded player on page))

789	[ever_prep]<br><br>Show the field ONLY if:<br>[event-name]= 'baseline_visit_arm_1' and [reported_hiv_status] = '1'	Have you ever taken antiretroviral medication (called PrEP) to prevent HIV?	radio, Required<br>1 No<br>2 Yes<br>3 Refuse to answer
790	[current_prep_eng]<br><br>Show the field ONLY if:<br>[acasi_language]='EN' AND [baseline_visit_arm_1][reported_hiv_status] = '1'	Section Header:	descriptive<br>(Attachment: Q157.mp3, Display format: Audio file (play in embedded player on page))
791	[current_prep_sw]<br><br>Show the field ONLY if:<br>[acasi_language]='SW' AND [baseline_visit_arm_1][reported_hiv_status] = '1'		descriptive<br>(Attachment: SW-Q157.mp3, Display format: Audio file (play in embedded player on page))
792	[current_prep_dh]<br><br>Show the field ONLY if:<br>[acasi_language]='DH' AND [baseline_visit_arm_1][reported_hiv_status] = '1'		descriptive<br>(Attachment: DH-Q157.mp3, Display format: Audio file (play in embedded player on page))
793	[current_prep]<br><br>Show the field ONLY if:<br>[baseline_visit_arm_1][reported_hiv_status] = '1'	Are you still taking antiretroviral medication (called PrEP) to prevent HIV?	radio, Required<br>1 No<br>2 Yes<br>3 Refuse to answer
794	[miss_prep_eng]<br><br>Show the field ONLY if:<br>[baseline_visit_arm_1][reported_hiv_status] = '1' and [current_prep]= '2' AND [acasi_language]='EN'		descriptive<br>(Attachment: Q158.mp3, Display format: Audio file (play in embedded player on page))
795	[miss_prep_sw]<br><br>Show the field ONLY if:<br>[baseline_visit_arm_1][reported_hiv_status] = '1' and [current_prep]= '2' AND [acasi_language]='SW'		descriptive<br>(Attachment: SW-Q158.mp3, Display format: Audio file (play in embedded player on page))
796	[miss_prep_dh]<br><br>Show the field ONLY if:<br>[baseline_visit_arm_1][reported_hiv_status] = '1' and [current_prep]= '2' AND [acasi_language]='DH'		descriptive<br>(Attachment: DH-Q158.mp3, Display format: Audio file (play in embedded player on page))
797	[miss_prep]<br><br>Show the field ONLY if:	Sometimes people may have difficulty remembering to take their medicines as instructed. During the last 30 days, did you miss taking your PrEP as instructed?	radio, Required<br>1 No<br>2 Yes

	[baseline_visit_arm_1][reported_hiv_status] = '1' and [current_prep]= '2'		3 Refuse to answer
798	[times_miss_prep_eng]<br><br>Show the field ONLY if: [baseline_visit_arm_1][reported_hiv_status] = '1' and [miss_prep] = '2' AND [acasi_language]='EN'		descriptive (Attachment: Q159.mp3, Display format: Audio file (play in embedded player on page))
799	[times_miss_prep_sw]<br><br>Show the field ONLY if: [baseline_visit_arm_1][reported_hiv_status] = '1' and [miss_prep] = '2' AND [acasi_language]='SW'		descriptive (Attachment: SW-Q159.mp3, Display format: Audio file (play in embedded player on page))
800	[times_miss_prep_dh]<br><br>Show the field ONLY if: [baseline_visit_arm_1][reported_hiv_status] = '1' and [miss_prep] = '2' AND [acasi_language]='DH'		descriptive (Attachment: DH-Q159.mp3, Display format: Audio file (play in embedded player on page))
801	[times_miss_prep]<br><br>Show the field ONLY if: [baseline_visit_arm_1][reported_hiv_status] = '1' and [miss_prep] = '2'	In the last 30 days, how many times did you miss your PrEP?	text (number), Required
802	[inst_end_en]<br><br>Show the field ONLY if: [acasi_language]='EN'		descriptive (Attachment: INST-Q159.mp3, Display format: Audio file (play in embedded player on page))
803	[inst_end_sw]<br><br>Show the field ONLY if: [acasi_language]='SW'		descriptive (Attachment: SW-INST-Q159.mp3, Display format: Audio file (play in embedded player on page))
804	[inst_end_dh]<br><br>Show the field ONLY if: [acasi_language]='DH'		descriptive (Attachment: DH-INST-Q159L.mp3, Display format: Audio file (play in embedded player on page))
805	[the_end]	Please let the research staff know you are done, so they can initial that you have completed.<br><br>STAFF ONLY: Please enter your initials in this field.	text, Required
806	[participant_acasi_form_complete]	Section Header: *Form Status*<br>Complete?	dropdown<br>0 Incomplete<br>1 Unverified<br>2 Complete
**Instrument: Clinical Case Report Form (clinical_case_report_form)**<br>Active languages: None			
807	[id_check_clinical]	Section Header: *Participant Identification*<br>Please enter participant ID carefully - this must match the study ID of the record you have opened in	text, Required

		order to proceed.	
808	[id_confirm_clinical]	Confirmation that record and entered ID match	calc<br>Calculation: if([study_id]=[id_check_clinical], 1, 0)<br>Field Annotation: @HIDDEN
809	[id_problem_clinical]<br>Show the field ONLY if:<br>[id_confirm_clinical]=0	WARNING: Entered study ID does not match REDCap Record Number. Check study ID carefully and reenter if you made a typo.If the study ID you entered is correct, please return to the Record Study Dashboard and open the correct record.	descriptive
810	[exam_date]	Date of Clinical Review	text (date_dmy), Required
811	[exposure_intro]	Exposure History<br>The following questions are asked to determine which sites (penis, rectum, throat) may have been exposed to STI and understand prior treatment and antibiotic exposures.	descriptive, Required
812	[insertive_sex]	In the past 3 months, have you had sex where you inserted your penis into someone's anus or vagina?	yesno, Required<br>1 Yes<br>0 No
813	[receptive_sex]	In the past 3 months, have you had sex where a male partner inserted his penis into your anus?	yesno, Required<br>1 Yes<br>0 No
814	[oral_sex]	In the past 3 months, have you had sex where your partner's penis was inserted into your mouth?	yesno, Required<br>1 Yes<br>0 No
815	[sti_treatment]	In the past 3 months, have you been treated for an STI outside this clinic?	yesno, Required<br>1 Yes<br>0 No
816	[syndrome_treated]<br>Show the field ONLY if:<br>[sti_treatment] = '1'	If yes, what kind of symptoms did you have?	radio, Required<br>1 Urethritis (pain or burning with urination, urethral discharge)<br>2 Proctitis (rectal pain or discharge)<br>3 Both urethritis and proctitis<br>4 Other
817	[other_symptoms]<br>Show the field ONLY if:<br>[syndrome_treated] = '4'	Other symptoms (specify)	text, Required
818	[days_symptomatic]<br>Show the field ONLY if:<br>[sti_treatment] = '1'	For how many days did you have symptoms?	text (number, Min: 0), Required
819	[any_abx]	In the past 3 months, have you taken antibiotics for any reason?	yesno, Required<br>1 Yes<br>0 No
820	[comments]	Comments on exposure history and/or antibiotics taken:	notes
821	[burn_urine]	Section Header: *STI Symptoms*	yesno, Required

		Do you currently have pain or burning with urination?	1 Yes<br>0 No
822	[urethral_discharge]	Do you currently have urethral discharge?	yesno, Required<br>1 Yes<br>0 No
823	[rectal_pain]	Do you currently have rectal pain?	yesno, Required<br>1 Yes<br>0 No
824	[rectal_discharge]	Do you currently have rectal discharge?	yesno, Required<br>1 Yes<br>0 No
825	[ulcer_symptom]	Do you currently have a genital ulcer?	yesno, Required<br>1 Yes<br>0 No
826	[side_effects]	Section Header: *Symptom Review, including Potential Side Effects of Medication*<br>In the past 3 months, have you had any of the following symptoms?	checkbox, Required<br>1 side_effects___1 Skin rash or sensitivity<br>2 side_effects___2 Headache<br>3 side_effects___3 Changes in vision<br>4 side_effects___4 Sore throat or pain with swallowing<br>5 side_effects___5 Difficulty swallowing<br>6 side_effects___6 Diarrhea<br>7 side_effects___7 Nausea<br>8 side_effects___8 Vomiting<br>9 side_effects___9 Abdominal pain<br>10 side_effects___10 Other<br>11 side_effects___11 None of the above
827	[other_side_effect]<br>Show the field ONLY if: [side_effects(10)]='1'	What other symptom did you experience?	notes
828	[pharynx_eryth_exud]	Section Header: *Physical Exam Findings*<br>Pharyngeal erythema or exudate on exam?	radio, Required<br>1 Yes<br>2 No<br>3 Refused
829	[inguinal_adenop]	Inguinal adenopathy on exam?	radio, Required<br>1 Yes<br>2 No<br>3 Refused
830	[ulcer]	Genital or penile ulcer on exam?	radio, Required<br>1 Yes

			2 No<br>3 Refused
831	[warts]	Genital or penile warts on exam?	radio, Required<br>1 Yes<br>2 No<br>3 Refused
832	[exam_urethra_discharge]	Urethral discharge on exam?	radio, Required<br>1 Yes<br>2 No<br>3 Refused
833	[ulcer_anal]	Perianal ulcer on exam?	radio, Required<br>1 Yes<br>2 No<br>3 Refused
834	[warts_anal]	Perianal warts on exam?	radio, Required<br>1 Yes<br>2 No<br>3 Refused
835	[exam_rectal_discharge]	Rectal discharge on exam?	radio, Required<br>1 Yes<br>2 No<br>3 Refused
836	[clinician_swab]	Section Header: *Specimen Collection*<br>Will participant accept clinician collected swabs?	yesno, Required<br>1 Yes<br>0 No
837	[clinician_swab_type]<br>Show the field ONLY if:<br>[clinician_swab]=1	Which swab or swabs will the clinician collect? Check all that apply.	checkbox<br>1 clinician_swab_type___1 Throat swab<br>2 clinician_swab_type___2 Rectal swab
838	[self_collect]	Will patient self-collect swabs?	yesno, Required<br>1 Yes<br>0 No
839	[self_collect_type]<br>Show the field ONLY if:<br>[self_collect]=1	Which swab or swabs will the patient collect? Check all that apply.	checkbox<br>1 self_collect_type___1 Throat swab<br>2 self_collect_type___2 Rectal swab
840	[throat_swabs]	Two throat swabs collected?	radio, Required<br>1 Yes (collected)<br>2 No (refused)
841	[rectal_swabs]	Two rectal swabs collected?	radio, Required<br>1 Yes (collected)<br>2 No (refused)

842	[urethral_swab]<br><br>Show the field ONLY if:<br>if([burn_urine]='1' or [urethral_discharge]='1' or [exam_urethra_discharge]='1', 1, 0)	Urethral swab collected?	radio<br>1 Yes (collected)<br>2 No (refused)
843	[urine_collected]	Urine specimen collected?	radio, Required<br>1 Yes (collected)<br>2 No (refused)
844	[blood_syphilis]	Blood for syphilis testing collected?	radio, Required<br>1 Yes (collected)<br>2 No (refused)
845	[hair_collected]<br><br>Show the field ONLY if:<br>[event-name]='3_month_visit_arm_1' or [event-name]='6_month_visit_arm_1' or [event-name]='9_month_visit_arm_1' or [event-name]='12_month_visit_arm_1' or [event-name]='15_month_visit_arm_1' or [event-name]='18_month_visit_arm_1'	Hair collected?	radio, Required<br>1 Yes (collected)<br>2 No (refused)<br>3 Not possible
846	[hair_method]<br><br>Show the field ONLY if:<br>[hair_collected] = '1'	How was the hair collected?	radio<br>1 Scissors<br>2 Razor
847	[hair_reason]<br><br>Show the field ONLY if:<br>[hair_collected] = '2' or [hair_collected] = '3'	Reason for not collecting hair sample	checkbox<br>1 hair_reason___1 Participant refusal<br>2 hair_reason___2 Completely bald or shaved (no scalp hair)<br>3 hair_reason___3 Braids or hairstyle prohibit collection
848	[urethritis]	Section Header: *Syndromic Treatment*<br><br>Urethritis present?	calc<br>Calculation: if([burn_urine]='1' or [urethral_discharge]='1' or [exam_urethra_discharge]='1', 1, 0)
849	[proctitis]	Proctitis present?	calc<br>Calculation: if([rectal_pain]='1' or [rectal_discharge]='1' or [exam_rectal_discharge]='1', 1, 0)
850	[days_syndrome]<br><br>Show the field ONLY if:<br>[urethritis]='1' or [proctitis]='1'	For how many days have symptoms been present?	text, Required
851	[syndrome_treatment]<br><br>Show the field ONLY if:<br>[urethritis]='1' or [proctitis]='1'	Syndromic treatment administered?	radio, Required<br>1 Yes (administered)<br>2 No (refused)

852	[staff_syndromic_treat]<br><br>Show the field ONLY if: [syndrome_treatment] = '1'	Staff initials for syndromic treatment administration	text, Required
853	[ept_discussed]<br><br>Show the field ONLY if: [syndrome_treatment] = '1'	Did you discuss expedited partner treatment?	radio, Required<br>1 Yes<br>2 No
854	[ept_partners]<br><br>Show the field ONLY if: [syndrome_treatment] = '1' and [ept_discussed] = '1'	For how many partners did the participant accept to take expediated partner treatment?	text (integer, Min: 0, Max: 10), Required
855	[ppt_eligibility_intro]<br><br>Show the field ONLY if: [randomization_arm_1][randomization_group]='0'	Now, you are assigned to the periodic presumptive treatment, or PPT, study arm in Mambo Matatu. That means we will provide PPT at this visit if you are eligible.<br><br>I'd like to ask you a few questions to determine if you are eligible for PPT. Is that OK?	descriptive, Required
856	[condomless_sex3]<br><br>Show the field ONLY if: [randomization_arm_1][randomization_group]='0'	Have you had anal sex without a condom at any time in the past 6 months?	yesno, Required<br>1 Yes<br>0 No
857	[multiple_sex3]<br><br>Show the field ONLY if: [randomization_arm_1][randomization_group]='0'	Have you had sex with more than one partner in the past 6 months?	yesno, Required<br>1 Yes<br>0 No
858	[partner_sti3]<br><br>Show the field ONLY if: [randomization_arm_1][randomization_group]='0'	Have any of your sex partners had a sexually transmitted infection in the past 6 months?	yesno, Required<br>1 Yes<br>0 No
859	[ppt_risk_criteria]	Meets PPT risk criteria	calc<br>Calculation: if(([condomless_sex3]='1' and ([multiple_sex3]='1' or [partner_sti3]='1'))='1', 1, 0)
860	[ppt_eligible]	eligible for PPT	calc<br>Calculation: if([randomization_arm_1][randomization_group]='0' and [ppt_risk_criteria]='1' and [urethritis]='0' and [proctitis]='0', 1, 0)
861	[eligible_presumptive]<br><br>Show the field ONLY if: [randomization_arm_1][randomization_group]='0' and [ppt_eligible]='1'	Presumptive treatment administered?	yesno, Required<br>1 Yes<br>0 No
862	[staff_ppt]<br><br>Show the field ONLY if: [randomization_arm_1][randomization_group]='0' and [ppt_eligible]='1'	Staff initials for presumptive treatment administration	text, Required

863	[doxypep_intro]<br><br>Show the field ONLY if:<br>[randomization_arm_1][randomization_group]='1'	You are assigned to the doxyPEP, study arm in Mambo Matatu. I'd like to review with you now how to use doxyPEP and how to get refills.	descriptive, Required
864	[doxypep_info]<br><br>Show the field ONLY if:<br>[randomization_arm_1][randomization_group]='1'	Reviewed doxyPEP purpose and instructions with participant?	yesno, Required<br>1 Yes<br>0 No
865	[doxypep_brought]<br><br>Show the field ONLY if:<br>[randomization_arm_1][randomization_group]='1' and ([event-name]='3_month_visit_arm_1' or [event-name]='6_month_visit_arm_1' or [event-name]='9_month_visit_arm_1' or [event-name]='12_month_visit_arm_1' or [event-name]='15_month_visit_arm_1' or [event-name]='18_month_visit_arm_1')	Did participant bring their remaining doxyPEP to clinic?	yesno, Required<br>1 Yes<br>0 No
866	[doxypep_count]<br><br>Show the field ONLY if:<br>[randomization_arm_1][randomization_group]='1' and [doxypep_brought]='1'	Please count the number of doxycycline capsules remaining and enter here.	text
867	[doxypep_continue]<br><br>Show the field ONLY if:<br>[randomization_arm_1][randomization_group] = '1' and ([event-name]='3_month_visit_arm_1' or [event-name]='6_month_visit_arm_1' or [event-name]='9_month_visit_arm_1' or [event-name]='12_month_visit_arm_1' or [event-name]='15_month_visit_arm_1' or [event-name]='18_month_visit_arm_1')	Participant wants to continue doxyPEP?	yesno, Required<br>1 Yes<br>0 No
868	[doxypep_reason]<br><br>Show the field ONLY if:<br>[randomization_arm_1][randomization_group]='1' and [doxypep_continue]='0'	Why does the participant not want to continue doxyPEP?	notes, Required
869	[doxypep_refill]<br><br>Show the field ONLY if:<br>[randomization_arm_1][randomization_group]='1'	Provided doxyPEP?	yesno, Required<br>1 Yes<br>0 No

870	[staff_doxypep_refill]<br><br>Show the field ONLY if:<br>[randomization_arm_1][randomization_group]='1' and [doxypep_refill]='1'	Staff initials for doxyPEP provision	text, Required
871	[comments_doxypep]<br><br>Show the field ONLY if:<br>[randomization_arm_1][randomization_group]='1'	Comments on doxyPEP refill	notes
872	[treatment_given]	Was any treatment other than syndromic treatment or the assigned intervention given at this visit?<br><br>Please check all that apply.	checkbox<br>1	treatment_given___1	syphilis treatment<br>2	treatment_given___2	wart treatment<br>3	treatment_given___3	herpes treatment<br>4	treatment_given___4	other
873	[other_treatment]<br><br>Show the field ONLY if:<br>[treatment_given(4)]	What other treatment was given?	text
874	[program_intro]	Program Data<br><br>Questions will only appear if consent for chart linkage was provided at enrolment.	descriptive
875	[hiv_status]<br><br>Show the field ONLY if:<br>[screening_and_cons_arm_1][program_data_consent] = '1'	HIV status per clinic records for [screening_and_cons_arm_1][program_id].	radio<br>1	Known Positive<br>2	Prior Negative<br>3	Unknown
876	[hiv_testing]<br><br>Show the field ONLY if:<br>[screening_and_cons_arm_1][program_data_consent] = '1' and [hiv_status] >1	HIV testing this visit or since last visit for [screening_and_cons_arm_1][program_id].	radio<br>1	New Positive<br>2	Negative<br>3	Indeterminant<br>4	Not done
877	[art_status]<br><br>Show the field ONLY if:<br>[screening_and_cons_arm_1][program_data_consent] = '1' and ([hiv_status] = '1' or [hiv_testing] = '1')	ART provided this visit or since last visit for [screening_and_cons_arm_1][program_id].	radio<br>1	At research clinic<br>2	At another clinic (per participant report)<br>3	Not taking ART
878	[prep_provided]<br><br>Show the field ONLY if:<br>[screening_and_cons_arm_1][program_data_consent] = '1' and ([hiv_status] = '2' OR [hiv_status] = '3') and [hiv_testing] = '2'	PrEP provided this visit or since last visit for [screening_and_cons_arm_1][program_id].	radio<br>1	At research clinic<br>2	At another clinic (per participant report)<br>3	Not taking PrEP
879	[pep_provided]<br><br>Show the field ONLY if:	PEP provided this visit date for [screening_and_cons_arm_1][program_id].	radio<br>1	At research clinic

	[screening_and_cons_arm_1][program_data_consent] = '1' and ([hiv_status] = '2' OR [hiv_status] = '3') and ([hiv_testing] = '2' or [hiv_testing] = '3') and [prep_provided]='3'		2 At another clinic (per patient report)<br>3 Not taking PEP
880	[program_comments]<br><br>Show the field ONLY if:<br>[screening_and_cons_arm_1][program_data_consent] = '1'	Comments about HIV status and prevention or care for [screening_and_cons_arm_1][program_id].	text
881	[clinical_case_report_form_complete]	Section Header: *Form Status*<br><br>Complete?	dropdown<br>0 Incomplete<br>1 Unverified<br>2 Complete
**Instrument: Interim Visit Form (interim_visit_form)**<br>Active languages: None			
882	[id_check_interim]	Section Header: *Participant Identification*<br><br>Please enter participant ID again carefully. This must match the study ID of the record you have opened in order to proceed.	text, Required
883	[id_confirm_interim]	Confirmation that record and entered ID match	calc<br>Calculation: if ([study_id]=[id_check_interim], 1, 0)<br>Field Annotation: @HIDDEN
884	[id_problem_interim]<br><br>Show the field ONLY if:<br>[id_confirm_interim]=0	WARNING: Entered study ID does not match REDCap Record Number. Check study ID carefully and reenter if you made a typo. If the study ID you entered is correct, please return to the Record Status Dashboard and open the correct record.	descriptive
885	[exam_date_iv]	Date of Interim Visit	text (date_dmy), Required
886	[interim_reason]	What is the purpose of the interim visit?<br><br>Check all that apply.	checkbox, Required<br>1 interim_reason___1 STI symptoms<br>2 interim_reason___2 Syphilis treatment<br>3 interim_reason___3 Adverse event
887	[syphilis_treatment]<br><br>Show the field ONLY if:<br>[interim_reason(2)]	Please initial to document receipt of syphilis treatment and update the site's syphilis case log.	text
888	[adverse_event]<br><br>Show the field ONLY if:<br>[interim_reason(3)]	Please briefly describe the adverse event here and complete an adverse event form.	notes
889	[burn_urine_iv]<br><br>Show the field ONLY if:<br>[interim_reason(1)]	Section Header: *STI Symptoms*<br><br>Do you currently have pain or burning with urination?	yesno, Required<br>1 Yes<br>0 No

890	[urethral_discharge_iv]<br>Show the field ONLY if:<br>[interim_reason(1)]	Do you currently have urethral discharge?	yesno, Required<br>1 Yes<br>0 No
891	[rectal_pain_iv]<br>Show the field ONLY if:<br>[interim_reason(1)]	Do you currently have rectal pain?	yesno, Required<br>1 Yes<br>0 No
892	[rectal_discharge_iv]<br>Show the field ONLY if:<br>[interim_reason(1)]	Do you currently have rectal discharge?	yesno, Required<br>1 Yes<br>0 No
893	[ulcer_symptom_iv]<br>Show the field ONLY if:<br>[interim_reason(1)]	Do you currently have a genital ulcer?	yesno, Required<br>1 Yes<br>0 No
894	[side_effects_2_iv]	Section Header: *Symptom Review, including Potential Side Effects of Medication*<br>Since your last visit, have you had any of the following symptoms?	checkbox, Required<br>1 side_effects_2_iv___1 Skin rash or sensitivity<br>2 side_effects_2_iv___2 Headache<br>3 side_effects_2_iv___3 Changes in vision<br>4 side_effects_2_iv___4 Sore throat or pain with swallowing<br>5 side_effects_2_iv___5 Difficulty swallowing<br>6 side_effects_2_iv___6 Diarrhea<br>7 side_effects_2_iv___7 Nausea<br>8 side_effects_2_iv___8 Vomiting<br>9 side_effects_2_iv___9 Abdominal pain<br>10 side_effects_2_iv___10 Other<br>11 side_effects_2_iv___11 None of the above
895	[other_side_effect_iv]<br>Show the field ONLY if:<br>[side_effects_2_iv(10)] = '1'	What other symptom did you experience?	notes
896	[pharynx_eryth_exud_iv]<br>Show the field ONLY if:<br>[interim_reason(1)]	Section Header: *Physical Exam Findings*<br>Pharyngeal erythema or exudate on exam?	radio, Required<br>1 Yes<br>2 No<br>3 Refused
897	[inguinal_adenop_iv]<br>Show the field ONLY if:<br>[interim_reason(1)]	Inguinal adenopathy on exam?	radio, Required<br>1 Yes<br>2 No<br>3 Refused

898	[ulcer_iv]<br><br>Show the field ONLY if:<br>[interim_reason(1)]	Genital or penile ulcer on exam?	radio, Required<br>1 Yes<br>2 No<br>3 Refused
899	[warts_iv]<br><br>Show the field ONLY if:<br>[interim_reason(1)]	Genital or penile warts on exam?	radio, Required<br>1 Yes<br>2 No<br>3 Refused
900	[exam_urethra_discharge_iv]<br><br>Show the field ONLY if:<br>[interim_reason(1)]	Urethral discharge on exam?	radio, Required<br>1 Yes<br>2 No<br>3 Refused
901	[ulcer_anal_iv]<br><br>Show the field ONLY if:<br>[interim_reason(1)]	Perianal ulcer on exam?	radio, Required<br>1 Yes<br>2 No<br>3 Refused
902	[warts_anal_iv]<br><br>Show the field ONLY if:<br>[interim_reason(1)]	Perianal warts on exam?	radio, Required<br>1 Yes<br>2 No<br>3 Refused
903	[exam_rectal_discharge_iv]<br><br>Show the field ONLY if:<br>[interim_reason(1)]	Rectal discharge on exam?	radio, Required<br>1 Yes<br>2 No<br>3 Refused
904	[clinician_swab_iv]<br><br>Show the field ONLY if:<br>[interim_reason(1)]	Section Header: *Specimen Collection*<br>Will participant accept clinician collected swabs?	yesno, Required<br>1 Yes<br>0 No
905	[clinician_collect_type_iv]<br><br>Show the field ONLY if:<br>[interim_reason(1)] and [clinician_swab_iv]=1	Which swab or swabs will the clinician collect? Check all that apply.	checkbox<br>1 clinician_collect_type_iv___1 Throat swab<br>2 clinician_collect_type_iv___2 Rectal swab
906	[self_collect_iv]<br><br>Show the field ONLY if:<br>[interim_reason(1)]	Will patient self-collect swabs?	yesno, Required<br>1 Yes<br>0 No
907	[self_swab_type_iv]<br><br>Show the field ONLY if:<br>[interim_reason(1)] and [self_collect_iv]=1	Which swab or swabs will the clinician collect? Check all that apply.	checkbox<br>1 self_swab_type_iv___1 Throat swab<br>2 self_swab_type_iv___2 Rectal swab
908	[throat_swabs_iv]<br><br>Show the field ONLY if:<br>[interim_reason(1)]	Two throat swabs collected?	radio, Required<br>1 Yes (collected)<br>2 No (refused)

909	[rectal_swabs_iv]<br><br>Show the field ONLY if:<br>[interim_reason(1)]	Two rectal swabs collected?	radio, Required<br>1 Yes (collected)<br>2 No (refused)
910	[urethral_swab_iv]<br><br>Show the field ONLY if:<br>if ([interim_reason(1)] and ([burn_urine_iv]='1' or [urethral_discharge_iv]='1' or [exam_urethra_discharge_iv]='1'), 1, 0)	Urethral swab collected?	radio<br>1 Yes (collected)<br>2 No (refused)
911	[urine_collected_iv]<br><br>Show the field ONLY if:<br>[interim_reason(1)]	Urine specimen collected?	radio, Required<br>1 Yes (collected)<br>2 No (refused)
912	[blood_syphilis_iv]<br><br>Show the field ONLY if:<br>[interim_reason(1)]	Blood for syphilis testing collected?	radio, Required<br>1 Yes (collected)<br>2 No (refused)<br>3 Not indicated (no signs of acute syphilis such as a chancre or rash)
913	[urethritis_iv]	Section Header: *Syndromic Treatment*<br><br>Urethritis present?	calc<br>Calculation: if([burn_urine_iv]='1' or [urethral_discharge_iv]='1' or [exam_urethra_discharge_iv]='1', 1, 0)
914	[proctitis_iv]	Proctitis present?	calc<br>Calculation: if([rectal_pain_iv]='1' or [rectal_discharge_iv]='1' or [exam_rectal_discharge_iv]='1', 1, 0)
915	[days_syndrome_iv]<br><br>Show the field ONLY if:<br>[urethritis_iv]='1' or [proctitis_iv]='1'	For how many days have symptoms been present?	text, Required
916	[syndrome_treatment_iv]<br><br>Show the field ONLY if:<br>[urethritis_iv]='1' or [proctitis_iv]='1'	Syndromic treatment administered?	radio, Required<br>1 Yes (administered)<br>2 No (refused)
917	[staff_syndromic_treat_iv]<br><br>Show the field ONLY if:<br>[syndrome_treatment_iv] = '1'	Staff initials for syndromic treatment administration	text, Required
918	[ept_discussed_iv]<br><br>Show the field ONLY if:<br>[syndrome_treatment_iv] = '1'	Did you discuss expedited partner treatment?	radio, Required<br>1 Yes<br>2 No
919	[ept_partners_iv]<br><br>Show the field ONLY if:<br>[syndrome_treatment_iv] = '1' and [ept_discussed_iv] = '1'	For how many partners did the participant accept to take expedited partner treatment?	text (integer, Min: 0, Max: 10), Required

920	[doxypep_iv]<br><br>Show the field ONLY if:<br>[randomization_arm_1][randomization_group]='1'	You are assigned to the doxyPEP, study arm in Mambo Matatu. I'd like to review with you now how you are using doxyPEP and whether you'd like a refill.	descriptive
921	[doxypep_info_iv]<br><br>Show the field ONLY if:<br>[randomization_arm_1][randomization_group]='1'	Reviewed doxyPEP purpose and instructions with participant?	yesno, Required<br>1 Yes<br>0 No
922	[doxypep_brought_iv]<br><br>Show the field ONLY if:<br>[randomization_arm_1][randomization_group]='1'	Did participant bring their remaining doxyPEP to clinic?	yesno, Required<br>1 Yes<br>0 No
923	[doxypep_count_iv]<br><br>Show the field ONLY if:<br>[randomization_arm_1][randomization_group]='1' and [doxypep_brought_iv]='1'	Please count the number of doxycycline capsules remaining and enter here.	text
924	[doxypep_continue_iv]<br><br>Show the field ONLY if:<br>[randomization_arm_1][randomization_group] = '1'	Participant wants to continue doxyPEP?	yesno, Required<br>1 Yes<br>0 No
925	[doxypep_reason_iv]<br><br>Show the field ONLY if:<br>[randomization_arm_1][randomization_group]='1' and [doxypep_continue_iv]='0'	Why does the participant not want to continue doxyPEP?	notes, Required
926	[doxypep_refill_iv]<br><br>Show the field ONLY if:<br>[randomization_arm_1][randomization_group]='1' and [doxypep_continue_iv]='1'	Provided doxyPEP refill?	yesno, Required<br>1 Yes<br>0 No
927	[staff_doxypep_refill_iv]<br><br>Show the field ONLY if:<br>[randomization_arm_1][randomization_group]='1' and [doxypep_refill_iv]='1'	Staff initials for doxyPEP refill provision	text, Required
928	[comments_doxypep_iv]<br><br>Show the field ONLY if:<br>[randomization_arm_1][randomization_group]='1' and [doxypep_continue_iv]='1'	Comments on doxyPEP refill	notes

929	[treatment_given_iv]	Was any treatment other than syndromic treatment or a doxyPEP refill given at this visit?<br><br>Please check all that apply.	checkbox<br>1 treatment_given_iv___1 syphilis treatment<br>2 treatment_given_iv___2 wart treatment<br>3 treatment_given_iv___3 herpes treatment<br>4 treatment_given_iv___4 other
930	[other_treatment_iv]<br><br>Show the field ONLY if:<br>[treatment_given_iv(4)]	What other treatment was given?	text
931	[interim_visit_form_complete]	Section Header: *Form Status*<br>Complete?	dropdown<br>0 Incomplete<br>1 Unverified<br>2 Complete
