# Supplementary material for: World Health Organization–Recommended Periodic Presumptive Treatment Versus Doxycycline Post-Exposure Prophylaxis for Sexually Transmitted Infection Control Among Men Who Have Sex With Men in Kenya: Protocol for a Randomized Controlled Trial
Source: JMIR Res Protoc. 2026 Jan 6;15:e81113. doi: 10.2196/81113 (PMC12820545; doi:10.2196/81113)
Supplement: Multimedia Appendix 2 [file resprot_v15i1e81113_app2.docx]

**Date _____________ Start time ____________________**

**Study site: ______________ [site] Patient ID: _____________ [pid]**

**Assignment: ____________ [study arm]**

1. **Introduction** *Hello, my name is _______________ and I am _____________________. I’ll be conducting this interview with you today. Thank you for taking the time to meet with me today and share your feedback on this study.*

*As you know, the goal of the Mambo Matatu study is to reduce the burden of bacterial sexually transmitted infections (STI), including gonorrhea, chlamydia, and syphilis among gay and bisexual men and other MSM in Kenya. You were among the first group of people to participate, and the purpose of this exit interview is to learn about your experience with the study arm to which you were assigned. Please be honest about your experiences. We want to know what worked well but also what did not work well or things you did not like. This honest feedback will help understand the potential for the two interventions we studied to be used outside of this study.*

*Do you have any questions before we begin?*

1. **Withdrawal reasons (if applicable)**
   1. Could you please provide your reasons for withdrawing from the Mambo Matatu study?
   2. Would you have liked to continue if those barriers were not present?
   3. We would like to have your reflections on the study overall, if you are willing to share. Is it OK to continue?
2. **Helpfulness of the program**
   1. Overall, how helpful, if at all, was the Mambo Matatu study for you?
      1. In what ways did the study benefit you?
   2. In what ways did the study address your needs?
   3. In what ways did the study NOT address your needs?
3. **Learning about / deciding to enroll in the study**
   1. How did you hear about this study?
   2. What did you first hear about the study?
   3. What made you decide to participate?
   4. Did you have a clear concept of the study when you decided to participate? (Why?)
   5. What concerns did you have about participating in the study?
   6. What helped you overcome those concerns?
4. **Study visits / clinic experiences**Later, we’ll talk a lot about the specific study arm you were assigned to. But first, I’d like to ask you about your study visits at the clinic.
   1. How were your study visits at [site]?
   2. What, if anything, made you feel comfortable during your study visits?
   3. What, if anything, made you feel uncomfortable during your study visits?
   4. What feedback or advice do you have so that we can improve future study visits for others in the community?
5. **Overall feedback on [study arm]**Thank you. Now we’ll talk about the study arm to which your were assigned, that is, [standard care / doxyPEP / periodic presumptive treatment].
   1. Overall, how was your experience with [study arm]?
      1. What, if anything, made your experience with [study arm] better?
      2. What, if anything, made your experience with [study arm] worse?
   2. What did you like the most about [study arm]?
   3. What did you like the least about [study arm]?
   4. Would you recommend [study arm] to others? Why or why not?
6. **Engagement and adherence**
   Thanks for sharing that information. Now, I’d like to get more specific feedback from you about how [study arm] worked for you.

**If [study arm] = standard care**

- 1. During the study, how many times did you have symptoms of an STI, such as burning when you urinate, urethral discharge, rectal pain, or rectal discharge?
  2. Were you able to report these symptoms to the clinic? If yes, how did that go? If no, why not?
  3. Did you receive treatment for an STI during the study? If yes, how did that go?
  4. What are your thoughts on standard care compared to the other interventions we studied (periodic presumptive treatment, doxyPEP)?
  5. Would you have preferred to have one of the other interventions? If so, why? If not, why?

**If [study arm] = periodic presumptive treatment**

1. During the study, how many times did you have symptoms of an STI, such as burning when you urinate, urethral discharge, rectal pain, or rectal discharge?
2. Were you able to report these symptoms to the clinic? If yes, how did that go? If no, why not?
3. Did you receive periodic presumptive treatment during the study? If yes, how did that go?
4. What are your thoughts on periodic presumptive treatment compared to the other arms we studied (standard care, doxyPEP)?
5. Would you have preferred to have one of the other interventions? If so, why? If not, why?

**If [study arm] = doxyPEP**

1. During the study, how many times did you have symptoms of an STI, such as burning when you urinate, urethral discharge, rectal pain, or rectal discharge?
2. Were you able to report these symptoms to the clinic? If yes, how did that go? If no, why not?
3. Did you receive doxyPEP during the study? If yes, how did that go?
4. Were you able to take the tablets as instructed?
5. Did you have any problems with storage of your tablets?
6. What are your thoughts on doxyPEP compared to the other arms we studied (standard care, periodic presumptive treatment)?
7. Would you have preferred to have one of the other interventions? If so, why? If not, why?
8. **Impact on sexual behavior**
   1. In your opinion, how did participating in the study impact your sexual behavior?
   2. Was anything different because of your participation, and if so, how was it different?
   3. How do you think the study arm you were in impacted your sexual behavior?
9. **Safety of the study**
   1. What negative experiences, if any, did you have because of your participation in this study? *[[Probe to get thorough description of the negative experiences.]]*
   2. What were some barriers, if any, that you experienced in participating in the study?
      1. Can you tell me more about that?
10. **Conclusion**Thank you so much for all that you have shared with me today. Before we end, I would like to give you an opportunity to share with me any other thoughts you have about the Mambo Matatu study.
    1. Is there anything else you’d like to tell me?
    2. Do you have any questions before we conclude?
