# Supplementary material for: World Health Organization–Recommended Periodic Presumptive Treatment Versus Doxycycline Post-Exposure Prophylaxis for Sexually Transmitted Infection Control Among Men Who Have Sex With Men in Kenya: Protocol for a Randomized Controlled Trial
Source: JMIR Res Protoc. 2026 Jan 6;15:e81113. doi: 10.2196/81113 (PMC12820545; doi:10.2196/81113)
Supplement: Multimedia Appendix 3 [file resprot_v15i1e81113_app3.docx]

**Date _____________ Start time ____________________**

**Study site: ______________ [site] Staff ID: _____________ [sid]**

1. **Introduction***Hello, my name is _______________ and I am _____________________. I’ll be conducting this interview with you today. Thank you for taking the time to meet with me today and share your feedback on this study.*

*As you know, the goal of the Mambo Matatu study is to reduce the burden of bacterial sexually transmitted infections (STI), including gonorrhea, chlamydia, and syphilis among gay and bisexual men and other MSM in Kenya. We are collecting feedback from staff about what worked well but also what did not work well or things you did not like. This honest feedback will help understand the potential for the two interventions we studied to be used outside of this study.*

*Do you have any questions before we begin?*

1. **Helpfulness of the program**
   1. Overall, how helpful, if at all, was the Mambo Matatu study to participants?
      1. In what ways did the study benefit participants?
   2. In what ways did the study address participant needs?
   3. In what ways did the study NOT address participant needs?
2. **Study visits / clinic experiences**Later, we’ll talk a lot about the specific study arms of the study. But first, I’d like to ask you about participant study visits at the clinic.
   1. How did the study visits go at [site]?
   2. What, if anything, made you feel comfortable conducting study visits?
   3. What, if anything, made you feel uncomfortable conducting study visits?
   4. What feedback or advice do you have so that we can improve future study visits for staff and participants?
3. **Standard Care**
   First, I’d like to get more specific feedback from you about how standard care (i.e., syndromic treatment) worked for staff and participants.
   1. Overall, how was your experience with providing standard care?
      1. What, if anything, made your experience providing standard care better?
      2. What, if anything, made your experience providing standard care worse?
   2. What did you like the most about providing standard care?
   3. What did you like the least about providing standard care?
   4. What are your thoughts on standard care compared to the other interventions we studied (periodic presumptive treatment, doxyPEP)?
4. **Periodic Presumptive Treatment**
   Next, I’d like to get more specific feedback from you about how periodic presumptive treatment worked for staff and participants.
   1. Overall, how was your experience with providing periodic presumptive treatment?
      1. What, if anything, made your experience providing periodic presumptive treatment better?
      2. What, if anything, made your experience providing periodic presumptive treatment worse?
   2. What did you like the most about providing periodic presumptive treatment?
   3. What did you like the least about providing periodic presumptive treatment?
   4. What are your thoughts on periodic presumptive treatment compared to the other interventions we studied (standard care, doxyPEP)?
5. **doxyPEP**
   Finally, I’d like to get more specific feedback from you about how doxyPEP worked for staff and participants.
   1. Overall, how was your experience with providing doxyPEP?
      1. What, if anything, made your experience providing doxyPEP better?
      2. What, if anything, made your experience doxyPEP worse?
   2. What did you like the most about providing doxyPEP?
   3. What did you like the least about providing doxyPEP?
   4. What are your thoughts on doxyPEP compared to the other interventions we studied (standard care, periodic presumptive treatment)?
6. **Safety of the study for staff**
   1. What negative experiences, if any, did you have because of your work on this study? *[[Probe to get thorough description of the negative experiences.]]*
   2. What were some barriers, if any, that you experienced in working on the study?
      1. Can you tell me more about that?
7. **Conclusion**Thank you so much for all that you have shared with me today. Before we end, I would like to give you an opportunity to share with me any other thoughts you have about the Mambo Matatu study.
   1. Is there anything else you’d like to tell me?
   2. Do you have any questions before we conclude?
