# Supplementary material for: World Health Organization–Recommended Periodic Presumptive Treatment Versus Doxycycline Post-Exposure Prophylaxis for Sexually Transmitted Infection Control Among Men Who Have Sex With Men in Kenya: Protocol for a Randomized Controlled Trial
Source: JMIR Res Protoc. 2026 Jan 6;15:e81113. doi: 10.2196/81113 (PMC12820545; doi:10.2196/81113)
Supplement: Multimedia Appendix 4 [file resprot_v15i1e81113_app4.pdf]

SUMMARY STATEMENT

(Privileged Communication)

PROGRAM CONTACT:
ELEANORE Chuang
(240) 747-7858
eleanore.chuang@nih.gov

Release Date: **07/03/2023**
Revised Date:

Application Number: **1R01AI179838-01**

Principal Investigators (Listed Alphabetically):

GRAHAM, SUSAN MARIE (Contact)
SANDERS, EDUARD

Applicant Organization: UNIVERSITY OF WASHINGTON

Review Group: **HSQE**
Health Services: Quality and Effectiveness Study Section

Meeting Date: **06/14/2023**
Council: **OCT 2023**
Requested Start: **09/01/2023**
Opportunity Number: **PA-20-183**
PCC: **M37B**

Project Title: **WHO-recommended Periodic Presumptive Treatment versus Doxycycline Post-Exposure Prophylaxis for STI Control among Cisgender Men Who Have Sex with Men in Kenya**
SRG Action: **Impact Score:22 Percentile:4**
Next Steps: **Visit https://grants.nih.gov/grants/next_steps.htm**
Human Subjects: 48-At time of award, restrictions will apply
Animal Subjects: 10-No live vertebrate animals involved for competing appl.
Gender: 3A-Only men, scientifically acceptable
Minority: 5A-Only foreign subjects, scientifically acceptable
Age: 7A-Only Adults, scientifically acceptable

Project Year	Direct Costs Requested	Estimated Total Cost
1	993,911	1,296,546
2	994,208	1,296,933
3	996,601	1,300,055
4	941,651	1,228,373
5	942,954	1,230,073
TOTAL	4,869,325	6,351,979

ADMINISTRATIVE BUDGET NOTE: The budget shown is the requested budget and has not been adjusted to reflect any recommendations made by reviewers. If an award is planned, the costs will be calculated by Institute grants management staff based on the recommendations outlined below in the COMMITTEE BUDGET RECOMMENDATIONS section.
BIOHAZARD

1R01AI179838-01 Graham, Susan

PROTECTION OF HUMAN SUBJECTS UNACCEPTABLE
BIOHAZARD COMMENT

RESUME AND SUMMARY OF DISCUSSION: This application proposed a clinical trial to compare WHO-recommended periodic presumptive treatment (PPT) or doxycycline post-exposure prophylaxis (doxyPEP) to standard syndromic treatment for *Neisseria gonorrhoeae* (NG) and *Chlamydia trachomatis* (CT) infections among cisgender men who have sex with men in Kenya. The study will also conduct culture-based and molecular analysis of antimicrobial resistance in NG. Panel members agreed that the proposed study was highly significant and innovative because, if successful, it will provide much-needed evidence to inform guidelines about STI control among men who have sex with men in sub-Saharan Africa. In particular, Aims 2 and 3 would still yield useful scientific knowledge even if there were challenges in the execution of the trial. The outstanding investigative team had complementary expertise, was particularly strong in modeling and cost effectiveness analysis, and had history of collaboration. Kenya's National AIDS and STI Control Programme would serve as a consultant. Other strengths of the proposed study included the appealing study design, well-described protocol with thorough description of recruitment and intervention strategies, microcosting approach, and excellent consideration of potential limitations and proposed alternatives. However, panel members also identified some weaknesses. These included: potentially limited generalizability; preliminary studies seemingly tangentially related to the proposed study; insufficient detail for Aim 2 data analyses and Aim 3 evaluation of parameter and structural uncertainty; and potential for participants with strong and extended social networks to share their randomization information. Some panel members found the proposed study to be complicated and ambitious, but others considered it feasible given team expertise and experience. Differences of opinion among panel members about how they weighed these issues largely diminished following the discussion. The panel ultimately concluded that the strengths far outweighed the weaknesses and that the proposed study would have a high impact on the field of STI control for men who have sex with men in sub-Saharan Africa.

DESCRIPTION (provided by applicant): Men who have sex with men (MSM) are at high risk for gonorrhea and chlamydia in Kenya, where nucleic acid amplification testing (NAAT) is not feasible and most infections therefore go undiagnosed. While development of low-cost point-of-care diagnostics is an urgent priority, low-cost and feasible interventions to control sexually transmitted infections (STI) among MSM are needed now, as we await technology advance. In 2011, the WHO recommended periodic presumptive treatment (PPT) of Neisseria gonorrhoeae (NG) and Chlamydia trachomatis (CT) infections for MSM at high risk for HIV acquisition due to condomless anal intercourse with multiple sex partners or a recent STI exposure. More recently, trials in well-resourced settings have demonstrated the efficacy of doxycycline post-exposure prophylaxis (doxyPEP) at reducing NG, CT, and syphilis infections among high-risk MSM. In this R01 application, we propose a rigorous study to evaluate the impact and cost-effectiveness of WHO-recommended PPT versus doxyPEP, compared to standard syndromic treatment, among Kenyan MSM. Our highly productive research team, including collaborators from the University of Washington, Aurum Institute, Nyanza Reproductive Health Society, and Partners for Health and Development in Africa, has expertise in clinical STI care, epidemiology, research with MSM populations, and intervention trials (MPI Graham and Sanders, co-Is Otieno and Kimani), NG and CT diagnosis and testing for antimicrobial resistance (AMR) (co-I Soge), infectious disease modeling (co-I Hamilton), and costing of interventions (co-I Sharma). This study aims to (1) evaluate the effectiveness and impact on AMR in NG of two interventions: WHO-recommended PPT given every 3 months and doxy-PEP taken 24-72 hours after condomless sex, compared to standard syndromic treatment, for reducing STI burden among Kenyan MSM; (2) assess the acceptability, feasibility, and safety of implementing WHO-recommended PPT and doxy-PEP compared to standard

care among providers and patients; and (3) model the health and economic impact of scaling up WHO-recommended STI PPT and doxyPEP compared to standard of care on STI control among MSM and their partners in Kenya. We will conduct an open-label randomized clinical trial with 2900 participants to evaluate these two interventions versus the standard of care assigned in a 2:2:1 ratio, with 18 months of follow-up and rigorous culture-based and molecular analysis of AMR in NG at three MSM-friendly research clinics in Kenya. We will use multidisciplinary science to measure the acceptability, feasibility, and safety of these two interventions, using a conceptual model based on Proctor's Implementation Science Framework. Finally, Aim 1 and 2 results will inform parameters to update a stochastic model of STI transmission and cost- effectiveness analysis to project the impact of scaled-up STI PPT and doxyPEP in Kenya. This work will provide the critical data needed to inform guidelines and improve STI control among these key populations in sub-Saharan Africa and other resource-limited settings.

PUBLIC HEALTH RELEVANCE: Men who have sex with men (MSM) are at high risk for gonorrhea and chlamydia in Kenya, where nucleic acid amplification testing is not feasible and most infections therefore go undiagnosed. We propose an open-label randomized clinical trial with 2900 participants assigned to WHO-recommended periodic presumptive treatment (PPT) or doxycycline post-exposure prophylaxis (doxyPEP), compared to standard syndromic treatment, with 18 months of follow-up and rigorous culture-based and molecular analysis of antimicrobial resistance in Neisseria gonorrhoeae. This work will provide critical data needed to inform guidelines and improve STI control among MSM in sub-Saharan Africa and other resource-limited settings, including modelled estimates of the health and economic impact of scaling up these two interventions on STI control among MSM and their partners in Kenya.

CRITIQUE 1

Significance: 3
Investigator(s): 3
Innovation: 4
Approach: 4
Environment: 2

Overall Impact: The proposal to perform an open-label RCT with a hybrid type 1 implementation-effectiveness component comparing each intervention (i.e., STI PPT, doxyPEP) to a common control in a 2:2:1 ratio addresses a significant clinical issue. This study design is appealing because it will allow evaluation of both interventions in the same trial, and an open-label design allows for optimal evaluation of effectiveness and robust evaluation of acceptability, feasibility, and safety in practice, as well as adherence and sexual behavior among participants who are aware of their assignment. In addition, the investigators propose to model the health and economic impact of scaling up WHO-recommended STI PPT and doxyPEP compared to standard of care on STI control among MSM and their partners in Kenya. The environment is adequate and the team has many years experience conducting research on sexual health for MSM in Kenya and expertise in clinical trial design, mixed methods research, implementation science, modeling, and cost-effectiveness analyses. The study is innovative in that evaluation of a recommended STI control strategy (i.e., STI PPT) for this MSM population in Kenya has never been rigorously evaluated. However, the study is complicated and ambitious, with myriad study components and staff to organize. Aim 2 feels confusing and a bit disorganized. Aim 3 (modeling aim) is useful. The results of this study will be useful for informing treatment strategies which may ultimately reduce STI prevalence.

1. Significance:

Strengths

- The proposal aims to evaluate control strategies for MSM facing disproportionate risk for syndromic treatment of STIs in resource-limited settings, given that the current standard of care in Kenya misses the majority of infections.
- Given that current diagnostic assays are too costly for most resource-limited settings and many infections are asymptomatic or otherwise missed, STI PPT has been proposed as a novel STI prevention strategy for MSM and TGW at risk for bacterial STI.
- Aim 3 seeks to use mathematical models to simulate long-term health and cost outcomes based on study data and to consider clinical outcomes beyond the scope and time horizon of the Aim 1 and Aim 2 work. These results are useful for providing important information to decision makers charged with defining priorities and allocating resources.

Weaknesses

- The potential emergence of AMR is an important concern for both doxyPEP and STI PPT. These are potential drawbacks of each strategy – a major public health concern globally that needs evaluation. A small weakness is that the study may introduce AMR.
- Potentially limited generalizability.

2. Investigator(s):

Strengths

- The investigative team is comprised of individuals from the University of Washington, the Aurum Institute, the Nyanza Reproductive Health Society, and Partners for Health and Development. The study team will be led by MPIs Graham in Seattle and Sanders in Kenya, supported by site PIs Otieno in Kisumu and Kimani in Nairobi. The MPIs Graham and Sanders have collaborated over the past 16 years in HIV prevention and care research in Kenya, thus have relevant experience. The team has many years experience conducting research on sexual health for MSM in Kenya and expertise in clinical trial design, mixed methods research, implementation science, modeling, and cost-effectiveness analyses.
- The MPIs have a well-established track record of research and complementary expertise.

Weaknesses

- The team is very large and will require an ambitious management plan.

3. Innovation:

Strengths

- Evaluation of a recommended STI control strategy (i.e., STI PPT) for this MSM population in Kenya has never been rigorously evaluated.
- Proposal aims to provide key data required to weigh the relative benefits, risks, and costs, by using trial data to model the impact and evaluate the cost-effectiveness of a scaled-up intervention.

Weaknesses

- None.

4. Approach:

Strengths

- The investigative team describes several prior studies in the preliminary data section.
- Figure 1 provides a useful CONSORT follow diagram summarizing eligibility, enrollment, randomization, follow-up, and analysis. The accompanying text provides additional details (e.g., study arms).
- Table 1 provides details on the operational components of Aim 1 such as inclusion/exclusion criteria, sample size, visit schedule, standard of care package.
- Power and sample size calculations assume primary trial outcome is combined prevalence of NG, CT, and early syphilis ascertained by lab diagnosis; requires reduction of 33% in STI prevalence, which appears sufficient (but is also a large percentage).

Weaknesses

- There are several prior studies described in the preliminary data section. However, the preliminary studies seem tangentially related – rather than directly related - to the proposed study. Including formation of a sexual and gender minority health research consortium; assessment of the prevalence of genitourinary and rectal symptoms among HIV-negative and –positive MSM; design and implementation of multiple stochastic, agent-based models of sexual networks; testing of a combined HIV-1 nucleic acid (NA) testing, linkage, treatment, and partner notification intervention.
- The inclusion of "safety" in Aim 2 is confusing and doesn't seem to go with acceptability and feasibility (implementation outcomes). The conceptual model is confusing – unclear what makes effectiveness or AMR rates or safety "service outcomes."
- Table 3 of quantitative data elements is similarly confusing – the organizational structure feels unclear and is difficult for reader to follow.
- The Aim 2 Data Analysis section states: "GEE with appropriate links and robust standard errors will be used to test retention across all study arms and to evaluate associations between study arm and the aim 2 quantitative outcomes." This is difficult to follow – need more detail as a reader (e.g., what links for which outcomes)?

5. Environment:

Strengths

- In the past 15 years, investigative team has conducted numerous studies with MSM in coastal Kenya and Kisumu.

Weaknesses

- None.

Study Timeline:

Strengths

- Mostly adequate.

Weaknesses

- Timeline specifies hiring/training in the first 6 months of study, but worth noting there will be turnover and need for retraining. Timeline specifies dissemination of results in quarter three, which is when all study activities begin (e.g., recruitment, enrollment, follow-up) with the exception of hiring/training.

Protections for Human Subjects:

Acceptable Risks and/or Adequate Protections

- Acceptable.

Data and Safety Monitoring Plan (Applicable for Clinical Trials Only):

Acceptable

Inclusion Plans:

- Sex/Gender: Distribution justified scientifically
- Race/Ethnicity: Distribution justified scientifically
- For NIH-Defined Phase III trials, Plans for valid design and analysis: Scientifically acceptable
- Inclusion/Exclusion Based on Age: Distribution justified scientifically
- Adequate.

Vertebrate Animals:

Not Applicable (No Vertebrate Animals)

- Adequate.

Biohazards:

Unacceptable

- Budget notes biohazard bags, but no other mention of biohazard.

Applications from Foreign Organizations:

Justified

- In collaboration with domestic organization (University of Washington).

Resource Sharing Plans:

Acceptable

Authentication of Key Biological and/or Chemical Resources:

Not Applicable (No Relevant Resources)

Budget and Period of Support:

Recommend as Requested

CRITIQUE 2

Significance: 2
Investigator(s): 2
Innovation: 2
Approach: 3
Environment: 1

Overall Impact: Investigators propose a study to evaluate the impact and cost-effectiveness of WHO-recommended periodic presumptive treatment (PPT) and doxycycline post-exposure prophylaxis (doxyPEP) compared to standard syndromic treatment, among Kenyan MSM. Specific aims include (1) evaluate the effectiveness and impact on AMR in NG of two interventions: WHO-recommended PPT given every 3 months and doxy-PEP taken 24-72 hours after condomless sex, compared to standard syndromic treatment, for reducing STI burden among Kenyan MSM; (2) assess the acceptability, feasibility, and safety of implementing WHO-recommended PPT and doxy-PEP compared to standard care among providers and patients; and (3) model the health and economic impact of scaling up WHO-recommended STI PPT and doxyPEP compared to standard of care on STI control among MSM and their partners in Kenya. This is a complex but well-described and supported study concept to address STIs and the potential for antimicrobial resistance in a LMIC. The excellent study team and highly detailed proposal mitigate concerns arising from the highly ambitious nature of the RCT.

1. Significance:

Strengths

- Complex but well described context and public health challenge – high significance and good consideration of adverse consequences (AMR) integrated into the study.
- Compelling argument to assess doxyPEP in the study setting; strong preliminary data demonstrating effectiveness of the strategy in high-income settings.
- Excellent detail in describing the underlying epidemiology, effectiveness of the proposed intervention and equipoise for the trial. The background was appreciated and very well written.
- Excellent description of the extensive preliminary research done leading up to this proposed study.
- While the RCT is ambitious in its planned recruitment, it's worth noting that Aims 2 and 3 can still yield useful scientific knowledge even if there are challenges in execution of the RCT.

Weaknesses

- None noted by the reviewer.

2. Investigator(s):

Strengths

- Exceptionally strong and well rounded team.
- The modeling and CEA components are particularly strong.

Weaknesses

- None noted by the reviewer.

3. Innovation:

Strengths

- Innovative study design and modeling approach to examine net benefits, including potential adverse consequences (AMR in particular).

Weaknesses

- None noted by the reviewer.

4. Approach:

Strengths

- Thorough description of study design, recruitment and intervention strategies – Figure 1 and table 1 were both extremely useful additions to the proposal.
- The breadth and detail provided for proposed measures throughout the trial was exemplary.
- The modeling approach is strong, building off of an already-developed model with a group of high-caliber investigators.
- The microcosting approach for the proposed interventions is appropriate and well described.
- Excellent consideration of potential limitations and proposed alternatives. This was very well considered.

Weaknesses

- The modeling approach was particularly complex and highly data intensive. While individual-based models can, in theory, more accurately replicate infectious disease dynamics, requirements for timely and representative data on a range of sexual risk behaviors challenge their utility and accuracy. In the absence of such data key parameters are often derived via calibration, which can easily settle on extreme values with limited and/or highly uncertain behavioral and epidemiological data. As such, a more detailed plan to evaluate both parameter and structural uncertainty is necessary. Both investigators and decision-makers need to be convinced that the results of the modeling studies and CEA are not an artifact of the structural assumptions and necessarily uncertain behavioral parameters of the model.

5. Environment:

Strengths

- Exceptionally strong – this multinational collaboration has a good track record of success.

Weaknesses

- None noted by the reviewer.

Study Timeline:

Strengths

- Appropriate, as planned.

Weaknesses

- None noted by the reviewer.

Protections for Human Subjects:
Acceptable Risks and/or Adequate Protections
Data and Safety Monitoring Plan (Applicable for Clinical Trials Only):
Acceptable

Inclusion Plans:
- Sex/Gender: Distribution justified scientifically
- Race/Ethnicity: Distribution justified scientifically
- For NIH-Defined Phase III trials, Plans for valid design and analysis: Scientifically acceptable
- Inclusion/Exclusion Based on Age: Distribution justified scientifically

Vertebrate Animals:
Not Applicable (No Vertebrate Animals)

Biohazards:
Not Applicable (No Biohazards)

Applications from Foreign Organizations:
Justified

Resource Sharing Plans:
Acceptable

Authentication of Key Biological and/or Chemical Resources:
Not Applicable (No Relevant Resources)

Budget and Period of Support:
Recommend as Requested

CRITIQUE 3

Significance: 3
Investigator(s): 2
Innovation: 2
Approach: 2
Environment: 3

Overall Impact: This proposal aims to evaluate the effectiveness of three prescribed interventions to treat STIs among MSM populations in Kenya: (1) standard of care, syndromic treatment, (2) WHO recommended guidelines: periodic presumptive treatment [PPT], and (3) post-exposure prophylaxis [via doxy-PEP]. The strengths of this proposal are the high significance to evaluate and provide clinical recommendations and best practices for STI prevention in resource-limited settings and the approach to compare outcomes using an open-label randomized clinical trial which simulates real-world experimental research.

1. Significance:

Strengths

- This study will significantly advance the field of STI prevention and control in low-resource countries as well as inform the effectiveness of WHO recommendations and policy guidelines for controlling STIs in MSM populations in low-resource settings. This proposal can lead to better informed clinical guidelines/practices and health policies.
- This will be the first study to compare WHO guidelines (periodic presumptive treatment, PPT), doxy-PEP (post-exposure prophylaxis), and the standard of care (syndromic treatment) in Kenya, a low-resource environment where routine STI testing is not affordable/accessible.
- This study will also weigh the costs of PPT and doxy-PEP, namely antimicrobial resistance in gonorrhea/chlamydia, which is a major global health concern.

Weaknesses

- Findings may be limited to Kenya and not generalizable to other settings, especially places without strong academic-community partnerships.

2. Investigator(s):

Strengths

- The investigators have over 15 years of experience collaborating in HIV/STI prevention and treatment in Kenya with locally based academic and community partners. The investigative team has successfully completed numerous NIH grants to study various aspects of HIV/STI prevention and treatment using mixed methods and cost-effectiveness modeling. The investigative team has successfully enrolled hundreds of participants to study HIV/STI prevalence and incidence, antimicrobial resistance, treatment adherence, partner notification, and patient acceptability of provider initiated testing.
- Kenya’s National AIDS and STI Control Programme will serve as a consultant.

Weaknesses

- None noted.

3. Innovation:

Strengths

- This will be one of the first studies to directly evaluate WHO-recommended STI presumptive treatment guidelines.
- Doxy-PEP has been proven to be effective in France and the United States, but not in low-resource settings without routine access to STI testing.

- This proposal will evaluate the differences between provider-based treatment (syndromic and presumptive treatment) versus patient-controlled prevention (doxy-PEP) for highly stigmatized infections.

Weaknesses

- The investigative team has already demonstrated that the WHO recommended guidelines have moderate sensitivity (74.1%) and low specificity (45.8%) for gonorrhea and chlamydia infections.

4. Approach:

Strengths

- The investigators have extensive experience recruiting MSM populations for HIV/STI prevention and treatment research.
- The open-label randomized clinical trial comparing PPT and doxy-PEP to the standard of care (syndromic treatment) is practical and simulates real-world procedures whereby patients know the drug name.
- This clinical trial will recruit both HIV+ and HIV- MSM.
- Aim 2 examines the acceptability and feasibility of various STI prevention guidelines and relies on a mixed methods approach (multilingual ACASI questionnaires, open-ended questions, exit interviews, doxycycline levels for validation, and direct clinical observations).
- Aim 3 will conduct a cost-effectiveness analysis (CEA) and include an NAAT-guided (routine STI assay test) treatment as a comparison which actualizes the global possibilities despite Kenya's resource-limited status. Micro-costing is also applied within the CEA, which is important for diverse and interdisciplinary audiences to understand the tradeoffs in global health research. Aim 3 will estimate the ICER per gonorrhea/chlamydia case treated which will inform best value-based practices moving forward.
- The investigators were careful to test an appropriate PPT regimen that was culturally appropriate with minimal risk of antimicrobial resistance.

Weaknesses

- The proposal does not describe how participants at study sites (with potentially strong and extended social networks) will not share their randomization information especially with open-label cohorts.
- The results will be limited to three study sites in the three largest cities in Kenya.

5. Environment:

Strengths

- The UW collaboration with the University of Nairobi East Africa STI Lab has operated with external quality assurance for more than 10 years.

Weaknesses

- None noted.

Study Timeline:

Strengths

- Appropriate.

Weaknesses

- None.

Protections for Human Subjects:

Unacceptable Risks and/or Inadequate Protections

- The trial will not diagnose and treat infections other than syphilis in real time. Gonorrhea and Chlamydia test results will be held until the study ends.

Data and Safety Monitoring Plan (Applicable for Clinical Trials Only):

Acceptable

Inclusion Plans:

- Sex/Gender: Distribution justified scientifically
- Race/Ethnicity: Distribution justified scientifically
- For NIH-Defined Phase III trials, Plans for valid design and analysis: Not applicable
- Inclusion/Exclusion Based on Age: Distribution justified scientifically

Vertebrate Animals:

Not Applicable

Biohazards:

Not Applicable

Applications from Foreign Organizations:

Not Applicable

Select Agents:

Not Applicable

Resource Sharing Plans:

Not Applicable

Authentication of Key Biological and/or Chemical Resources:

Not Applicable

Budget and Period of Support:

Recommend as Requested

THE FOLLOWING SECTIONS WERE PREPARED BY THE SCIENTIFIC REVIEW OFFICER TO SUMMARIZE THE OUTCOME OF DISCUSSIONS OF THE REVIEW COMMITTEE, OR REVIEWERS' WRITTEN CRITIQUES, ON THE FOLLOWING ISSUES:

PROTECTION OF HUMAN SUBJECTS: UNACCEPTABLE
Please see comment by Reviewer 3 about infection diagnoses not being provided in real time.

INCLUSION OF WOMEN PLAN: ACCEPTABLE

INCLUSION OF MINORITIES PLAN: ACCEPTABLE

INCLUSION ACROSS THE LIFESPAN: ACCEPTABLE

BIOHAZARD COMMENT: Please see comment by Reviewer 1 about biohazard bags.

COMMITTEE BUDGET RECOMMENDATIONS: The budget was recommended as requested.

Footnotes for 1R01AI179838-01; PI Name: Graham, Susan Marie

NIH has modified its policy regarding the receipt of resubmissions (amended applications).See Guide Notice NOT-OD-18-197 at https://grants.nih.gov/grants/guide/notice-files/NOT-OD-18-197.html. The impact/priority score is calculated after discussion of an application by averaging the overall scores (1-9) given by all voting reviewers on the committee and multiplying by 10. The criterion scores are submitted prior to the meeting by the individual reviewers assigned to an application, and are not discussed specifically at the review meeting or calculated into the overall impact score. Some applications also receive a percentile ranking. For details on the review process, see http://grants.nih.gov/grants/peer_review_process.htm#scoring.

MEETING ROSTER

Health Services: Quality and Effectiveness Study Section
Healthcare Delivery and Methodologies Integrated Review Group
CENTER FOR SCIENTIFIC REVIEW
HSQE
06/14/2023 - 06/15/2023

Notice of NIH Policy to All Applicants: Meeting rosters are provided for information purposes only. Applicant investigators and institutional officials must not communicate directly with study section members about an application before or after the review. Failure to observe this policy will create a serious breach of integrity in the peer review process, and may lead to actions outlined in NOT-OD-22-044 at https://grants.nih.gov/grants/guide/notice-files/NOT-OD-22-044.html, including removal of the application from immediate review.

CHAIRPERSON(S)

RICHARDSON, LYNNE D, MD
PROFESSOR AND VICE CHAIR
DEPARTMENT OF EMERGENCY MEDICINE
ICAHN SCHOOL OF MEDICINE AT MOUNT SINAI
NEW YORK, NY 10029

MEMBERS

BANEGAS, MATTHEW P, PHD
ASSOCIATE PROFESSOR
DEPARTMENT OF RADIATION MEDICINE
AND APPLIED SCIENCES
UNIVERSITY OF CALIFORNIA, SAN DIEGO
LA JOLLA, CA 92037

BLAKENEY, ERIN L, PHD *
RESEARCH ASSISTANT PROFESSOR
DEPARTMENT OF BIOBEHAVIORAL NURSING
AND HEALTH INFORMATICS
SCHOOL OF NURSING
UNIVERSITY OF WASHINGTON
SEATTLE, WA 98195-9472

BRAITHWAITE, DEJANA K, PHD *
PROFESSOR
DEPARTMENTS OF SURGERY AND EPIDEMIOLOGY
UNIVERSITY OF FLORIDA
GAINESVILLE, FL 32610

BRIESACHER, BECKY A, PHD
ASSOCIATE PROFESSOR
DEPARTMENT OF PHARMACY AND HEALTH SYSTEMS
SCIENCES
NORTHEASTERN UNIVERSITY
SCHOOL OF PHARMACY
BOSTON, MA 02115

BROOKS CARTHON, J MARGO, PHD *
ASSOCIATE PROFESSOR OF NURSING AND AFRICANA
STUDIES
DEPARTMENT FAMILY AND COMMUNITY HEALTH
SCHOOL OF NURSING
UNIVERSITY OF PENNSYLVANIA
PHILADELPHIA, PA 19104

BUTLER, ANNE M, PHD *
ASSISTANT PROFESSOR
DEPARTMENT OF MEDICINE
SCHOOL OF MEDICINE
WASHINGTON UNIVERSITY
ST. LOUIS, MO 63110

CABANA, MICHAEL D, MD
PROFESSOR
DEPARTMENT OF PEDIATRICS
ALBERT EINSTEIN COLLEGE OF MEDICINE
BRONX, NY 10467

CARPENTER, DELESHA M, PHD *
ASSOCIATE PROFESSOR
DIVISION OF PHARMACEUTICAL OUTCOMES AND POLICY
UNIVERSITY OF NORTH CAROLINA
ASHEVILLE, NC 28804

CHOO, ESTHER K, MD
PROFESSOR
DEPARTMENT OF EMERGENCY MEDICINE
OREGON HEALTH AND SCIENCE UNIVERSITY
PORTLAND, OR 97239

GONZALES, GILBERT, PHD *
ASSISTANT PROFESSOR
THE CENTER FOR MEDICINE, HEALTH & SOCIETY
VANDERBILT UNIVERSITY MEDICAL CENTER
NASHVILLE, TN 37203

HEO, MOONSEONG, PHD *
PROFESSOR
DEPARTMENT OF PUBLIC HEALTH SCIENCES
COLLEGE OF BEHAVIORAL, SOCIAL AND HEALTH SCIENCES
CLEMSON UNIVERSITY
CLEMSON, SC 29634

KENDZOR, DARLA E, PHD
PROFESSOR AND DIRECTOR, HEALTH PROMOTION RESEARCH CENTER
DEPARTMENT OF FAMILY AND PREVENTIVE MEDICINE
UNIVERSITY OF OKLAHOMA HEALTH SCIENCES CENTER
OKLAHOMA CITY, OK 73104

LI, LIANG, PHD
PROFESSOR
DEPARTMENT OF BIOSTATISTICS
THE UNIVERSITY OF TEXAS MD ANDERSON CANCER CENTER
HOUSTON, TX 77030

MAZURENKO, OLENA, MD, PHD
ASSOCIATE PROFESSOR
DEPARTMENT OF HEALTH POLICY AND MANAGEMENT
RICHARD M. FAIRBANKS SCHOOL OF PUBLIC HEALTH
INDIANA UNIVERSITY
INDIANAPOLIS, IN 46202

MEURER, WILLIAM J, MD
PROFESSOR
DEPARTMENT OF EMERGENCY MEDICINE AND NEUROLOGY
UNIVERSITY OF MICHIGAN SCHOOL OF MEDICINE
ANN ARBOR, MI 48109

MILLER, DAVID P, MD
PROFESSOR
DEPARTMENT OF MEDICINE AND PUBLIC HEALTH SCIENCES
WAKE FOREST UNIVERSITY SCHOOL OF MEDICINE
WINSTON-SALEM, NC 27157

NDUMELE, CHIMA, PHD
ASSOCIATE PROFESSOR
DEPARTMENT OF HEALTH POLICY AND MANAGEMENT
SCHOOL OF PUBLIC HEALTH
YALE UNIVERSITY
NEW HAVEN, CT 06520

NOSYK, BOHDAN, PHD
ENDOWED CHAIR AND ASSOCIATE PROFESSOR
FACULTY OF HEALTH SCIENCES
SIMON FRASER UNIVERSITY
VANCOUVER, BC V6Z 1Y6
CANADA

OSAZUWA-PETERS, NOSAYABA, PHD *
DIRECTOR OF CLINICAL RESEARCH TRAINING AND ASSOCIATE PROFESSOR
DEPARTMENT OF HEAD AND NECK SURGERY AND COMMUNICATION SCIENCES
SCHOOL OF MEDICINE
DUKE UNIVERSITY
DURHAM, NC 27710-4000

PERSELL, STEPHEN, MD, MPH *
PROFESSOR OF MEDICINE
DIVISION OF GENERAL INTERNAL MEDICINE
SCHOOL OF MEDICINE
NORTHWESTERN UNIVERSITY
CHICAGO, IL 60611

POPESCU, IOANA, MD
ASSOCIATE PROFESSOR
DEPARTMENT OF MEDICINE
RONALD REAGAN UCLA MEDICAL CENTER
UNIVERSITY OF CALIFORNIA, LOS ANGELES
LOS ANGELES, CA 90095

PRESTON, MICHAEL A, PHD *
ASSISTANT PROFESSOR
DEPARTMENT OF HEALTH BEHAVIOR AND POLICY
SCHOOL OF MEDICINE
VIRIGINA COMMONWEALTH UNIVERSITY
RICHMOND, VA 23298-0149

SAMUS, QUINCY M, PHD
PROFESSOR
DEPARTMENT OF PSYCHIATRY AND BEHAVIORAL SCIENCES
JOHNS HOPKINS UNIVERSITY SCHOOL OF MEDICINE
BALTIMORE, MD 21224

SHAFI, TARIQ, MBBS, MHS
PROFESSOR
HEAD, DIVISION OF KIDNEY DISEASES
HYPERTENSION AND TRANSPLANTATION
DEPARTMENT OF MEDICINE
HOUSTON METHODIST HOSPITAL
HOUSTON, TX 77030

SHAO, HUI, PHD *
ASSOCIATE PROFESSOR
HUBERT DEPARTMENT OF GLOBAL HEALTH
DEPARTMENT OF FAMILY AND PREVENTIVE MEDICINE
ROLLINS SCHOOL OF PUBLIC HEALTH
EMORY UNIVERSITY
ATLANTA, GA 30322

SHERMAN, SCOTT E, MD
PROFESSOR
DEPARTMENT OF POPULATION HEALTH, MEDICINE AND PSYCHIATRY
NEW YORK UNIVERSITY SCHOOL OF MEDICINE
NEW YORK, NY 10016

UNRUH, MARK L, MD, MS *
PROFESSOR AND CHAIR
DEPARTMENT OF INTERNAL MEDICINE
UNIVERSITY OF NEW MEXICO SCHOOL OF MEDICINE
ALBUQUERQUE, NM 87107

VONGPATANASIN, WANPEN, MD
PROFESSOR
DEPARTMENT OF INTERNAL MEDICINE
DIVISION OF CARDIOLOGY
UNIVERSITY OF TEXAS SOUTHWESTERN MEDICAL CENTER
DALLAS, TX 75390

WEI, YU-JUNG, PHD *
ASSOCIATE PROFESSOR
DIVISION OF OUTCOMES AND TRANSLATION SCIENCES
COLLEGE OF PHARMACY
OHIO STATE UNIVERSITY
COLUMBUS, OH 43210

ZARZAUR, BEN L, MD, MPH
ENDOWED CHAIR AND PROFESSOR
DEPARTMENT OF SURGERY
UNIVERSITY OF WISCONSIN SCHOOL OF MEDICINE
MADISON, WI 53704

ZUBKOFF, LISA, PHD *
ASSOCIATE PROFESSOR
DIVISION OF PREVENTIVE MEDICINE
DEPARTMENT OF MEDICINE
UNIVERSITY OF ALABAMA AT BIRMINGHAM
BIRMINGHAM, AL 35233

MAIL REVIEWER(S)

DARROW, LYNDSEY, PHD
PROFESSOR
DEPARTMENT OF EPIDEMIOLOGY
SCHOOL OF COMMUNITY HEALTH SCIENCES
UNIVERSITY OF NEVADA, RENO
RENO, NV 89557

SEELMAN, KRISTIE L, MSW, PHD
ASSOCIATE PROFESSOR
SCHOOL OF SOCIAL WORK
ANDREW YOUNG SCHOOL OF POLICY STUDIES
GEORGIA STATE UNIVERSITY
ATLANTA, GA 30302

SCIENTIFIC REVIEW OFFICER

THRASHER, ANGELA D, PHD
SCIENTIFIC REVIEW OFFICER
CENTER FOR SCIENTIFIC REVIEW
NATIONAL INSTITUTES OF HEALTH
BETHESDA, MD 20892

EXTRAMURAL SUPPORT ASSISTANT

JOHNSON, MELANIE
LEAD EXTRAMURAL SUPPORT ASSISTANT
CENTER FOR SCIENTIFIC REVIEW
NATIONAL INSTITUTES OF HEALTH
BETHESDA, MD 20817

NJOKU, PHILIP C
EXTRAMURAL SUPPORT ASSISTANT
DIVISION OF AIDS, BEHAVORIAL, POPULATION SCIENCES
HEALTHCARE DELIVERY AND METHODOLOGIES (HDM)
NATIONAL INSTITUTES OF HEALTH
BETHESDA, MD 20892

* Temporary Member. For grant applications, temporary members may participate in the entire meeting or may review only selected applications as needed.

Consultants are required to absent themselves from the room during the review of any application if their presence would constitute or appear to constitute a conflict of interest.
